# Global health 2050: the road to halving premature death by mid-century

**DOI:** 10.1016/S0140-6736(24)01439-9

**Published:** 2024-10-14

**Authors:** Dean T Jamison, Lawrence H Summers, Angela Y Chang, Omar Karlsson, Wenhui Mao, Ole F Norheim, Osondu Ogbuoji, Marco Schäferhoff, David Watkins, Olusoji Adeyi, George Alleyne, Ala Alwan, Shuchi Anand, Ruth Nigatu Belachew, Seth Berkley, Stefano Bertozzi, Sarah Bolongaita, Donald Bundy, Flavia Bustreo, Marcia Castro, Simiao Chen, Victoria Y Fan, Ayodamope Fawole, Richard Feachem, Lia Tadesse Gebremedhin, Jayati Ghosh, Sue Goldie, Eduardo Gonzalez-Pier, Yan Guo, Sanjeev Gupta, Prabhat Jha, Felicia Marie Knaul, Margaret Kruk, Christoph Kurowski, Gordon Liu, Saeda Makimoto, Awad Mataria, Rachel Nugent, Hitoshi Oshitani, Ariel Pablos-Mendez, Richard Peto, Neelam Sekhri Feachem, Srinath Reddy, Nisreen Salti, Helen Saxenian, Justina Seyi-Olajide, Agnes Soucat, Stéphane Verguet, Armand Zimmerman, Gavin Yamey

**Affiliations:** Department of Epidemiology & Biostatistics, University of California, San Francisco USA; Mossavar-Rahmani Center for Business and Government, John F. Kennedy School of Government, Harvard University, Cambridge, MA, USA; Department of Public Health, Danish Institute for Advanced Study, University of Southern Denmark, Odense, Denmark; Duke Global Health Institute, Duke University, Durham, NC, USA; Duke Global Health Institute, Duke University, Durham, NC, USA; Bergen Centre for Ethics and Priority Setting, Department of Global Public Health and Primary Care, University of Bergen, Bergen Norway; Duke Global Health Institute, Duke University, Durham, NC, USA; Open Consultants, Berlin, Germany; Department of Global Health, University of Washington, Seattle, WA, USA; Resilient Health Systems, Washington, DC, USA; Pan American Health Organization, Washington, DC, USA; London School of Hygiene & Tropical Medicine, London, England, UK; Center for Tubulointerstitial Kidney Disease, Stanford University, Stanford, CA, USA; Ministry of Health, Addis Ababa, Ethiopia; Pandemic Center, School of Public Health, Brown University, Providence, RI, USA; School of Public Health, University of California, Berkeley, Berkeley, CA, USA; University of Bergen, Bergen, Norway; Department of Population Health, London School of Hygiene & Tropical Medicine, London, England, UK; Fondation Botnar, Basel, Switzerland; Harvard T.H. Chan School of Public Health, Harvard University, Cambridge, MA, USA; Heidelberg Institute of Global Health, Heidelberg University, Heidelberg, Germany; Center for Global Development, Washington, DC, USA; Duke Global Health Institute, Duke University, Durham, NC, USA; Institute for Global Health Sciences, University of California San Francisco, San Francisco, CA, USA; Harvard Ministerial Leadership Program, Division of Policy Translation and Leadership Development, Harvard T.H. Chan School of Public Health, Harvard University, Boston, MA, USA; Department of Economics, College of Social & Behavioral Sciences, University of Massachusetts Amherst, Amherst, MA, USA; Department of Health Policy and Management, Harvard T.H. Chan School of Public Health, Harvard University, Boston, MA, USA; Palladium Group Inc, Washington, DC, USA; Institute for Global Health and Development, School of Public Health, Peking University, Beijing, China; Center for Global Development, Washington, DC, USA; Center for Global Health Research, Dalla Lana School of Public Health, University of Toronto, Toronto, Canada; Institute for Advanced Study of the Americas, Leonard M. Miller School of Medicine, University of Miami, Miami, USA; Harvard T.H. Chan School of Public Health, Harvard University, Cambridge, MA, USA; World Bank, Washington, DC, USA; Institute for Global Health and Development, Peking University, Beijing, China; Ogata Sadako Research Institute for Peace and Development, Japan International Cooperation Agency, Tokyo, Japan; WHO Regional Office for the Eastern Mediterranean, Cairo, Egypt; Department of Global Health, University of Washington, Seattle, WA, USA; Department of Virology, Graduate School of Medicine, Tohoku University, Sendai, Miyagi, Japan; Irving Medical Center, Columbia University, New York, NY, USA; Nuffield Department of Population Health, Oxford University, Oxford, UK; Center for Global Health Diplomacy, Delivery, and Economics, University of California, San Francisco, San Francisco, CA, USA; Public Health Foundation of India, New Delhi, India; Department of Economics, American University of Beirut, Beirut, Lebanon; Results for Development Institute, Washington, DC, USA; Department of Surgery, Lagos State University Teaching Hospital, Lagos, Nigeria; Department of Surgery, Lagos State University Teaching Hospital, Lagos, Nigeria; Harvard H.T. Chan School of Public Health, Harvard University, Boston, MA, USA; Duke Global Health Institute, Duke University, Durham, NC, USA; Duke Global Health Institute, Duke University, Durham, NC, USA

## Abstract

Global health 2050 (GH2050), a new report from the Lancet Commission on Investing in Health, finds that dramatic improvements in human welfare are achievable by mid-century with focused health investments. By 2050, countries that choose to do so can halve their probability of premature death (PPD)—the probability of dying before age 70—from their pre-pandemic level in 2019. We call this goal “50 by 50”: a 50% reduction in PPD by 2050. The interventions for achieving “50 by 50” will also reduce morbidity and disability at all ages.

Historical experience and continued scientific advance indicate that this is a feasible aspiration. Eight of the 30 most populous countries reduced their PPD over the last decade at a rate that would halve PPD before 2050, including countries as diverse as Bangladesh, Iran, Tanzania, and Turkey. These focused gains can be achieved relatively early on the pathway to full universal health coverage (UHC).

The path to achieving “50 by 50” runs through control of a remarkably narrow set of just 15 conditions. For currently high mortality countries, eight infectious diseases and maternal conditions are the highest priority. Seven clusters of noncommunicable diseases and injuries are important everywhere and addressing them will prove central to achieving “50 by 50” in most countries with lower initial levels of mortality.

Focused attention to health system strengthening (HSS) for primary care and first level hospitals will generate capacity to better tackle the 15 priority conditions and will be a critical step on the way to improving capacity to address all the conditions in a UHC package. Packaging interventions into 19 modules (e.g., a childhood immunization module, a module on cardiovascular disease prevention and low-cost, widely available treatment) will address the 15 priority conditions. Adopting this focused approach also invests in key areas of HSS and addresses major morbidities, such as psychiatric illness, not already covered by mortality-reducing interventions. Value for money can be assessed through a two-step process: technical cost effectiveness to assess how best to achieve module-specific goals (e.g., reduction in child mortality, reduction in cardiovascular mortality) and political evaluation of trade-offs in investing in expanding module coverage.

In many countries seeking reform, standard budgetary mechanisms have failed to successfully reorient systems toward priority interventions that improve health. This mechanism of blanket budget transfers from ministries of finance to ministries of health has not been fit to support such reorientation. The Commission concluded that this problem could be addressed by directing a substantial and increasing fraction of budget transfers to making available and affordable the specific drugs, vaccines, diagnostics, and other commodities that are currently available for control of the 15 priority conditions. Drug availability and affordability will typically require four complementary components: (i) redirecting general budget transfers to line item transfers (subsidies) for specific priority drugs; (ii) centralized procurement by government (or perhaps internationally); (iii) procurement in sufficient volumes to ensure availability when needed; and (iv) use and strengthening of existing supply chains, public and private.

Of the many intersectoral policies that governments can adopt to help achieve “50 by 50,” tobacco control is by far the most important, given the number of deaths caused by tobacco and the established and improving capacity of governments to implement tobacco policy. A high level of tobacco taxation is essential, and valuable in the short to medium term for public finance, and should be accompanied by a package of other effective tobacco control policies.

Background research conducted for the Commission points to exceptionally high ongoing levels of mortality risk from pandemics. Country performance against COVID-19 varied greatly, although eventual vaccine availability attenuated, but far from eliminated, this variability by the end of COVID-19’s emergency phase. National implementation of public health fundamentals—early action, isolation of infected individuals, quarantining of those exposed, and social and financial support for people isolating or quarantining—accounted for much of the success of the best-performing nations, such as Japan. In the next pandemic, these fundamentals will help to avert mortality while waiting for vaccine development and deployment.

The conclusions above are primarily aimed at national governments. Our final conclusion is aimed at the development assistance community. We conclude that such assistance should focus on two broad purposes. The first is to provide direct financial and technical support to countries with the least resources—to help develop health systems to better control diseases. The second is to finance global public goods, including strengthening data systems; reducing the development and spread of antimicrobial resistance; preventing and responding to pandemics; fostering global health leadership and advocacy; identifying and spreading best practices; and developing and deploying new health technologies. For both purposes, focusing efforts on the 15 priority conditions would best contribute to “50 by 50.” A decade ago, there were no malaria vaccines and the only available tuberculosis vaccine had low efficacy. Today, two partially successful malaria vaccines have been approved and three promising tuberculosis vaccine candidates are in late stage trials. These successes exemplify the enormous value in funding development of new medicines, vaccines, diagnostics, and operational research against the 15 priority conditions.

The prize of “50 by 50,” with an interim milestone of “30 by 2035” (a 30% reduction in PPD by 2035), remains a prize within reach. The most efficient route is to focus resources against a narrow set of conditions and scale up financing to develop and deploy new health technologies. Our economic analyses have shown that the value of achievable mortality declines remains high and indeed is often a substantial fraction of the value of gains in gross domestic product. Today, the case is better than ever for the value of investing in health for reducing mortality and morbidity, alleviating poverty, growing economies, and improving human welfare.

## Section 1. Introduction

Forty years ago, when the use of economic analysis in improving global health was gaining traction, the World Bank published the influential World Development Report 1993 (WDR1993), “Investing In Health.”^1^ It was the first and only time that the Bank devoted its annual flagship World Development Report, probably the world’s most widely distributed economic publication, to the topic of health improvement. Aimed at finance ministers and international aid donors, the report’s central message was that targeted spending on cost-effective interventions for high-burden diseases could rapidly improve public health outcomes, boost the economy, and improve human welfare.

Twenty years later, in 2013, this core message was re-examined in the first report of the *Lancet* Commission on Investing in Health (CIH).^2^ The report, “Global health 2035: a world converging within a generation” (GH2035), examined long term trends in progress in health. It found that from about 1850 onwards, life expectancy in the best performing countries increased steadily at a rate of about 2.5 years of life expectancy every decade until the time GH2035 was published. GH2035 then pointed to the promise of an ambitious but focused framework for achieving “grand convergence” by 2035. In a grand convergence, mortality from infections and maternal conditions could, in countries that chose to do so, be brought down to levels that would allow their life expectancies to converge toward that of the ‘frontier’ country (the best-performing country).

In 2018, five years after publication of GH2035, on the occasion of the 40th anniversary of the Alma-Ata Declaration, the Lancet invited the CIH to assess progress towards grand convergence and to reflect on the future of the global push for universal health coverage (UHC). The second CIH report (CIH 2.0), “Alma Ata at 40 years: reflections from the Lancet Commission on Investing in Health,” found a partially positive picture on progress towards convergence.^3^ As with GH2035, the CIH 2.0 report departed from mainstream thinking on UHC by stressing the need for selectivity in initial inclusion of interventions in health benefit packages.

### A changed context for investing in health

Rising geopolitical tensions, increasingly manifest climate change, growth in nationalistic populism, dwindling concern for global health, slowed progress towards UHC, and, most significantly, the COVID-19 pandemic, have defined the six years since CIH 2.0. This third report from the CIH (CIH 3.0), “Global health 2050: the road to halving premature death by mid-century” (GH2050), assesses these challenges—as well as opportunities for investment in health in increasingly turbulent times. It extends the time frame under consideration from 2035 to 2050. It also doubles the authorship to 50 authors, with stronger representation of early career researchers and scholars in low- and middle-income countries.

In drafting this CIH 3.0 report, we have learned lessons from the experiences of publishing the GH2035 and CIH 2.0 reports. GH2035 had a demonstrable impact on global health organizations–for example, it informed global women’s and children’s health strategies at the WHO and the Partnership for Maternal, Newborn and Child Health and it helped support the Global Fund’s fourth replenishment replenishment.^4^ Since 2013, there has been impressive progress on HIV, child mortality, and other high priority targets. GH2035 also fed into discussions of the Sustainable Development Goals,^5^ e.g., we briefed the Open Working Group on SDGs multiple times. However, as we discuss later in this report, while many low- and middle-income nations prioritized domestic health spending, others struggled with challenges such as debt and national security and they deprioritized health. In GH2050, we are more realistic when it comes to public spending on health. We hope that our focused approach to achieving mortality reduction and improving health at all ages informs discussions of the current and post-SDGs targets and framework.

Underlying data on economic, social, demographic, and health system indicators include gaps and inaccuracies for all countries but particularly so for those low- and lower middle-income countries where national statistical systems are often severely under-resourced. The World Bank, the United Nations Population Division, and the World Health Organization have made major efforts to construct time series of numbers in ways that are comparable across countries and time. The United Nations (UN) institutions producing these data are forthcoming about underlying weaknesses and explicit about the methodologies they use in assembling their publications. The Commission on Investing in Health uses their results for a wide range of analyses in this report and wishes to explicitly acknowledge its debt to the UN institutions while recognizing that non-UN data sources are available (e.g., recent estimates from the GBD 2021 Demographics Collaborators^6^). At the same time, we too are aware of data shortcomings and will welcome improved data as they become available over time. We encourage readers with better data sources for particular countries or indicators to use those data and point out their availability to us. Rather than repeating our assessment and concerns about data quality throughout this report, we do so at this point only (except when we have had reason to rely on nonstandard sources, as with some statistics on COVID-19).

### Structure of this report

This new report has eight further sections. Section 2 documents progress in global health indicators over the last 50 years, from 1970–2023; such historical experience can give an indication of future trends in mortality decline. In section 3, we explore the feasibility of all nations halving their probability of premature mortality by 2050 (a 50% reduction by 2050, or “50 by 50”). An important milestone on the way to this 2050 goal would be a 30% reduction in premature mortality by 2035, and we examine the feasibility of reaching this milestone. In section 4, we make a case for prioritizing the control of a set of 15 conditions to achieve “50 by 50.” In section 5, we propose a modular approach to strengthening health systems to achieve “50 by 50” and we introduce a new tool, modular cost effectiveness analysis. Section 6 explores ways to finance and deliver the GH2050 agenda. In Section 7, we argue that the world is doing far too little to prepare for the next pandemic and we outline the key steps that could be taken. In section 8, we consider the critical role of intersectoral policies in addressing the high-impact social determinants of health. We focus in particular on smoking, as it is the most important social determinant of morbidity and mortality in most countries and the most actionable, given the overwhelming evidence on the effectiveness of large excise tax increases. Section 9 looks at trends in and priorities for international collective action for health.

Despite the headwinds, we remain convinced, as we were when GH2035 was published, that ever-improving technical capacity combined with focused investment in tackling the 15 priority conditions offer the potential for major health improvements to provide large gains in human welfare. Doubling down on the successful health investments of recent decades promises continued success. Even in a world of seemingly intractable problems, for countries that choose to prioritize health, the prize of “50 by 50” is within reach.

## Section 2. Health in a world of change, 1970–2023

“What’s past is prologue,” says Shakespeare’s Antonio in *The Tempest—*in other words, what has taken place in the past helps to predict future opportunities. Understanding past trends in health progress provides a reality check on what is feasible for global health improvement by 2050.

In this section, we therefore analyze how key health metrics have changed since 1970. First, we examine the fifty-year period from 1970 to 2019, which was defined by steady progress in most countries, with some major exceptions. Next, we focus on the 2020–2023 era, in which the COVID-19 pandemic and major conflicts caused setbacks. Finally, we reflect on key trends today that will likely shape the global health response from 2024 to 2050.

**Figure 1:**
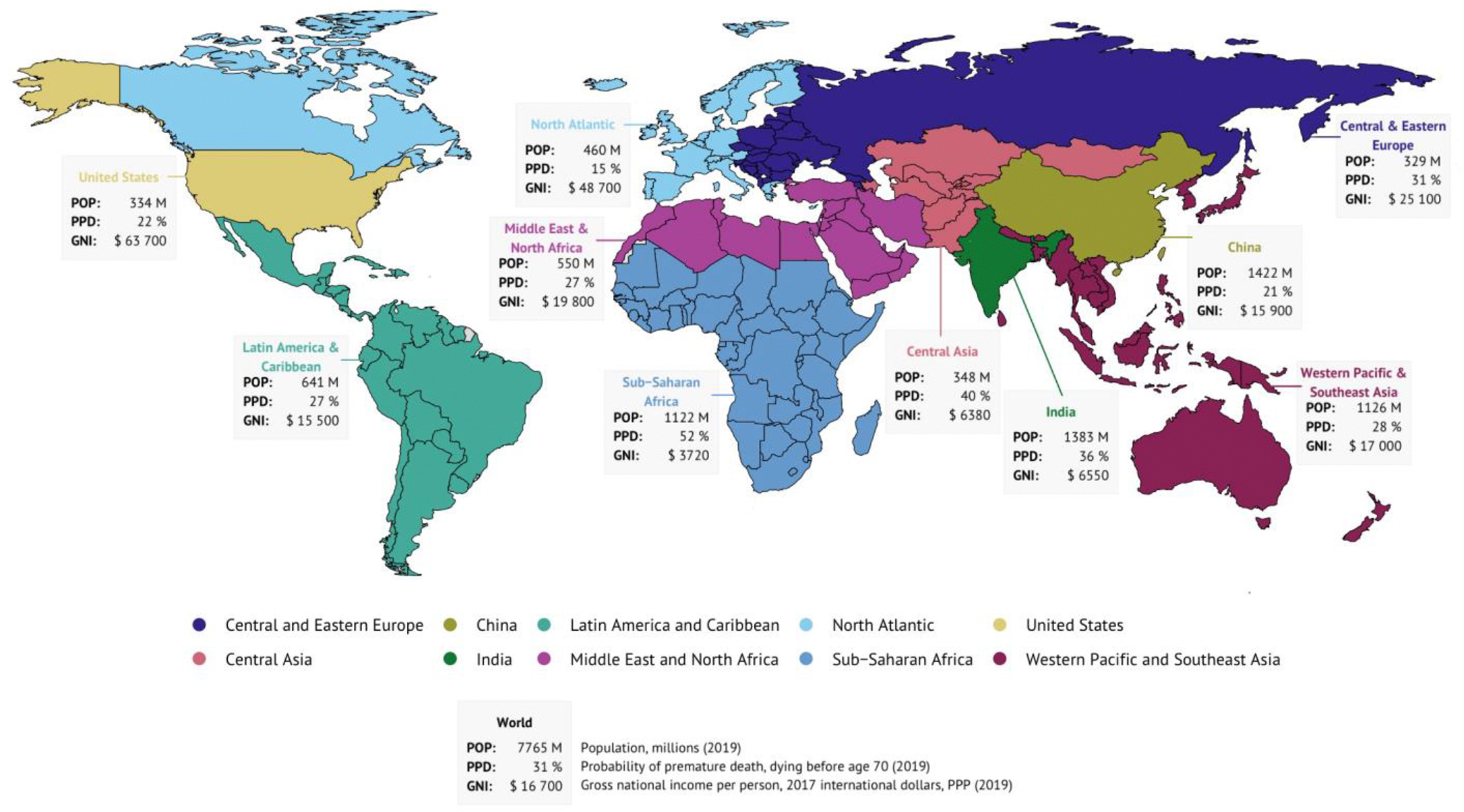
Map of CIH regions with basic statistics

In examining the progress of countries and regions for this CIH 3.0 report, we have used the regional groupings shown in figure 1 (the table on page 3 of the appendix shows a list of countries in each CIH region, and the table on page 6 of the appendix gives basic health, economic, and demographic indicators for each region). The CIH regions differ from the World Bank regional groupings in two ways. First, we separate out China from the East Asia and Pacific region and India from the South Asia region because the statistics of these two nations dominate their regions. Second, we create a CIH region called “North Atlantic” that comprises western European countries and Canada–these are countries with high performance on health indicators. We separate out the United States since (i) its health metrics are so distinct (it does not perform as well as western European countries and Canada), and, (ii) after China and India, the US is the world’s third most populous country.

A key metric that we use throughout this report is the probability of premature death (PPD), defined as the probability of dying before age 70 years under the current age-specific mortality rates (panel 1). PPD also serves as a proxy for progress against mortality after age 70 and morbidity. We use 70 years as the cut-off based on a previous CIH study, by Norheim et al,^7^ which defined premature death “somewhat arbitrarily as death before age 70 years.” The authors noted that: “World life expectancy is now just over 70 years, and most deaths before that age are avoidable.” As Richard Doll says, “In old age death is inevitable, but death before old age is not.”^8^

### Panel 1: Measuring survival progress: shifting from life expectancy at birth to probability of premature death

Life expectancy at birth (LE) is a commonly used measure for monitoring progress in population health. It is often misunderstood—as Wolf says, “People think it means that when they’re reporting life expectancy for 2022 that this is how long a baby who is born in 2022 will live.”^9^ The actual definition of LE is the expected number of years a newborn would live if prevailing patterns of age-specific mortality at the time of birth were to remain throughout its life. Despite such misunderstandings, LE is widely used–including occasionally in this report–due to its ease of communication.^10^

In CIH 3.0, we use the probability of premature death (PPD) as a primary indicator, defined as the probability of dying before age 70 years under the current age-specific mortality rates. As described below, PPD is related to LE, and both LE and PPD are independent of the age structure of the underlying population. We chose PPD as the main indicator in GH2050 for three reasons.

First, PPD encapsulates improvements in survival across all age groups before age 70 more effectively than LE, which is crucial as more deaths shift to older ages in most countries. As of 2019, the global median age of death was 76 years, with projections indicating a rise to 82 years by 2050. The highest and lowest median age of death in 2019 are found in the North Atlantic CIH region (at 84 years) and sub-Saharan Africa CIH region (at 65 years), and they are projected to increase to 89 years and 69 years, respectively.^11^

Second, while LE is influenced by both age-specific death rates and the remaining life years of each age group, PPD is only affected by the former. For example, the same number of deaths reduced in younger ages will have a greater impact on LE than in older ages due to the higher remaining life years in the younger groups. Therefore, a modest decline in LE may mask large reductions in mortality at older ages. In contrast, LE is a more common way to show changes in younger age mortality.

Finally, the differences in reflecting progress in survival between the two measures become more evident as the overall mortality levels fall.^12^ Between 2000 and 2019, in sub-Saharan Africa LE rose from 50.8 to 60.9 years (a 20 percent increase), while PPD dropped from 66% to 52% (a 21 percent decrease) (appendix table 2.6 on page 16 of the appendix). Both LE and PPD show similar levels of relative improvements (20 vs 21 percent). In contrast, in the North Atlantic CIH region, LE saw a modest rise from 78.6 to 82.4 years (a 5 percent increase) against a PPD decline from 21 to 15% (a 28 percent decrease). As seen in this example, changes in PPD are in close agreement with LE in regions with higher mortality, but more sensitively characterize the magnitude of change in countries with lower mortality levels.

#### 1970–2019: a period of steady progress, with major exceptions

**Figure 2:**
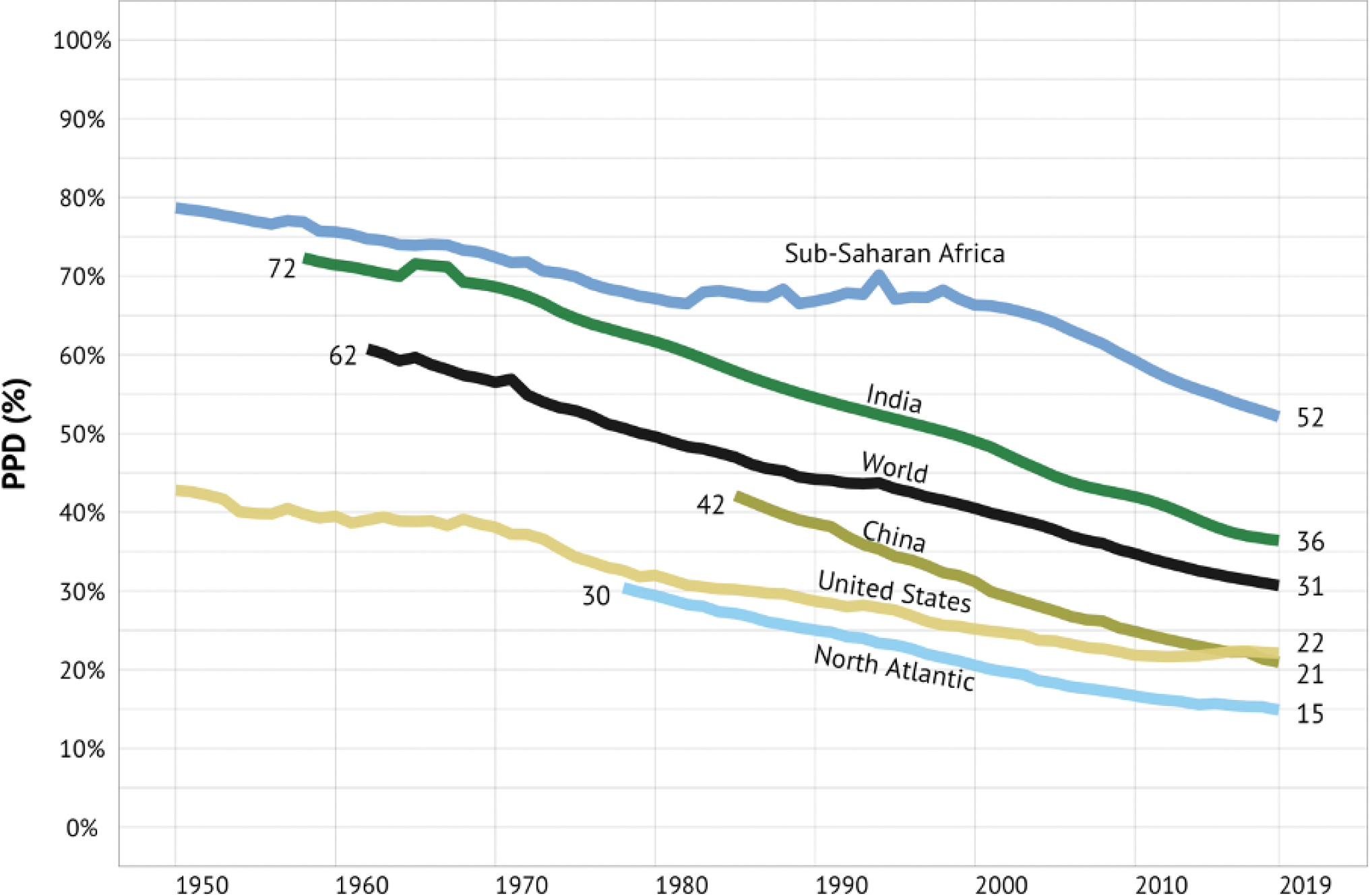
The probability of premature death, 1970–2019: World, China, India, North Atlantic, Sub-Saharan Africa, and the United States Note: The graph highlights the time taken for certain regions—namely the North Atlantic, China, the World, and India—to halve their PPD values to 2019 levels. Data from reference 14 ^14^

From 1970–2019, there was a striking decline in the global PPD, from 57% in 1970 to 31% in 2019 (figure 2). The PPD declined in all CIH regions, with China and the North Atlantic making particularly impressive progress. In contrast, the PPD in the US declined relatively slowly in the period following 1970 and actually rose steadily in the final decade of the 50-year period. The PPD in India in 2019 (36%) was lower than the PPD of the US in 1970 (38%). In 2019, the best performing region was the North Atlantic, with a PPD of 15%, while the region with the highest PPD was sub-Saharan Africa (52%). The HIV/AIDS pandemic was a major setback for mortality in the 1990s in sub-Saharan Africa, when the PPD rose.^13^ However, since 2000, the rate of improvement in mortality was faster in much of sub-Saharan Africa than in any other region.

The findings shown in figure 2 and table 1 are important context for our analysis later in this report on whether it would be feasible for all countries to halve their PPD by 2050 and whether countries worldwide could converge towards a lower PPD by mid-century. Table 1 shows, for the world’s 30 most-populous nations, how many years it would take to halve the PPD assuming the rate of improvement in PPD that these nations achieved from 2010–2019 (appendix tables 2.3 and 2.4 on pages 7–9 of the appendix give basic statistical and health metrics for these 30 countries, including health performance relative to income from 2010–2019). Eight of these countries would halve their PPD by 2050 or earlier, including countries as diverse as Bangladesh, Iran, Tanzania, and Turkey. In other words, for these eight nations, “business as usual” would be enough, though an acceleration in progress would be needed for the other 22 countries. As we discuss in sections 3 and 4, such acceleration can be achieved by a tight focus on a priority set of 15 conditions, combined with scaling up of research and development (R&D) to develop new disease control tools. Appendix table 2.5 on page 11 of the appendix gives information on PPD in 1970 and 2019 in all 123 countries with populations greater than 5 million.

**Figure 3:**
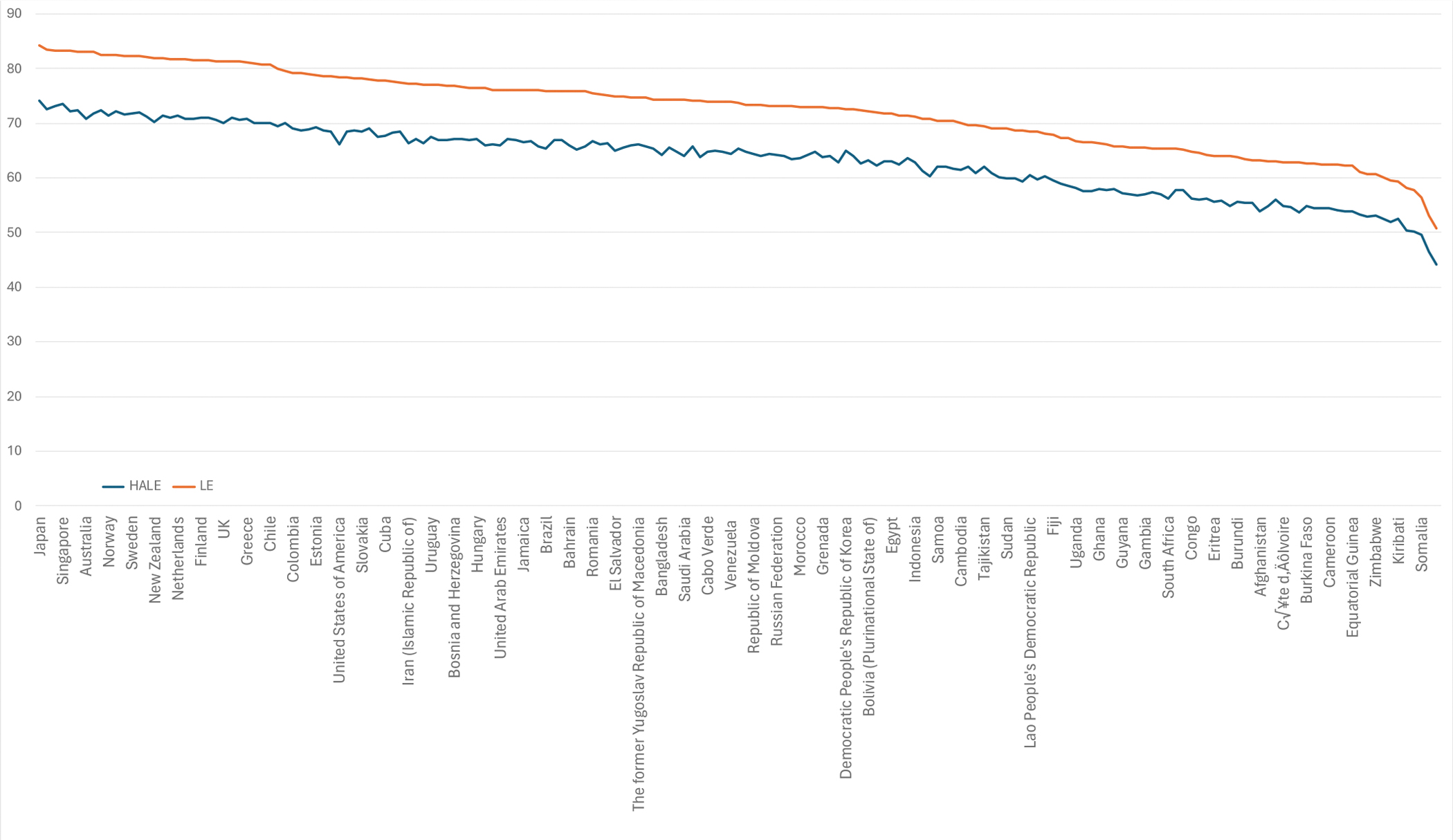
Life expectancy (LE) versus health-adjusted life expectancy (HALE) Source from reference 14 ^14^ Data from reference 15 ^15^

While our focus above has been on mortality improvement, a background paper prepared for CIH 3.0 shows that mortality is highly correlated with morbidity.^14^ Thus, health interventions that are put in place to drive down mortality will also improve morbidity and levels of functioning. Figure 3 shows that life expectancy (LE) is highly correlated with health-adjusted life expectancy (HALE). It also shows that in countries that have achieved a high LE, not all additional years of life are in full health. There are important exceptions to the correlation between mortality and morbidity or loss of functions; these include psychiatric disorders, old age dementias, and failure in normal growth of children and adolescents (discussed further in section 4).

**Figure 4:**
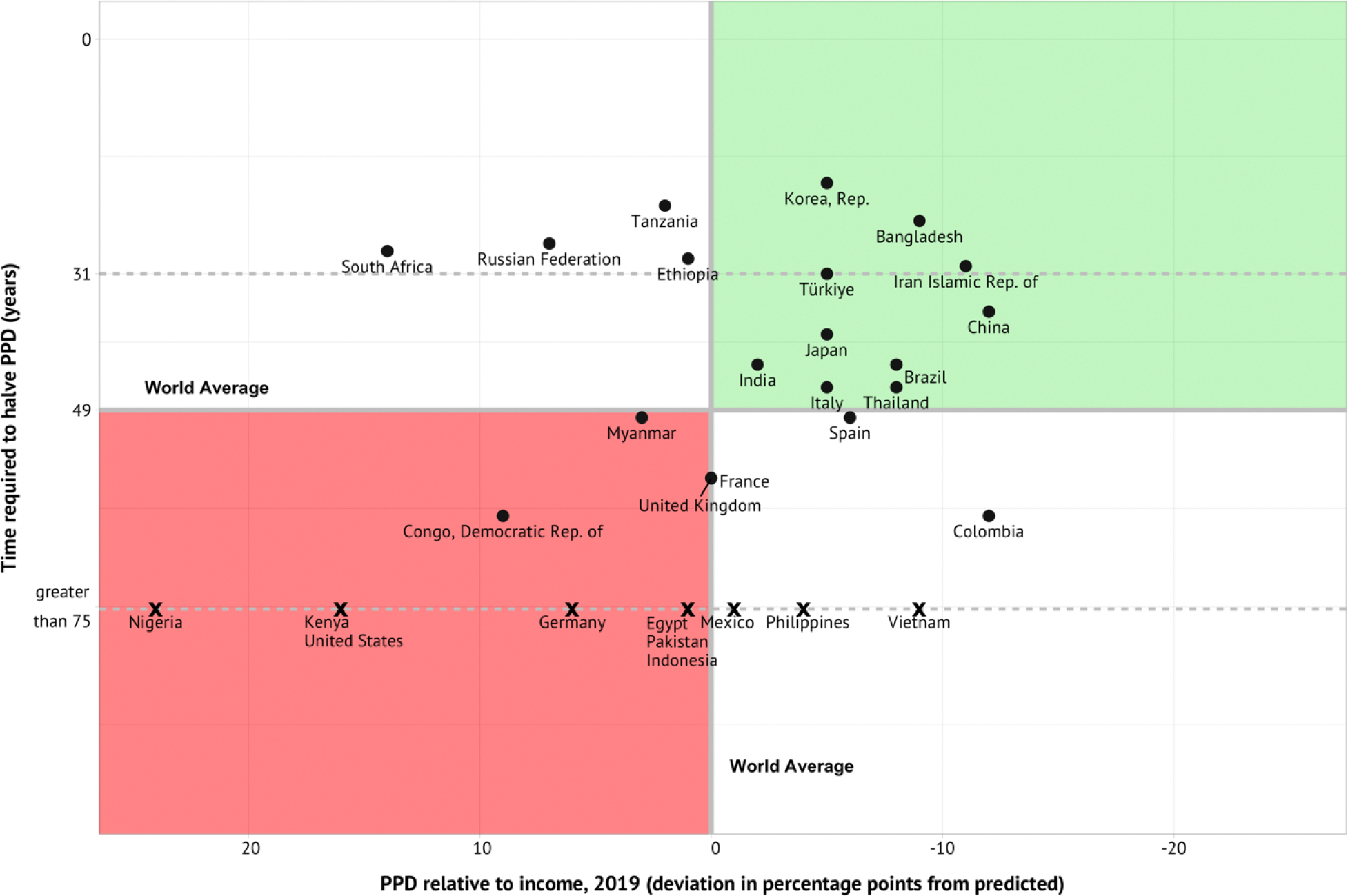
Country performance on the probability of premature death – level relative to per capita income, and halving time, world’s 30 most populous countries, 2019 Notes: 1. PPD is the probability of premature death, i.e., death before age 70. World average PPD was 31% in 2019. 2. The y-axis values show the number of years required to halve a country’s PPD if its rate of improvement in 2010–19 were to continue. The horizontal line drawn at 31 years indicates a halving time just adequate to reduce premature mortality by 50% between 2019 and 2050. Values above 75 years are simply indicated as ‘greater than 75’. 3. The x-axis values show a country’s deviation from the PPD that would be predicted from its income in 2019. The 2019 PPD for Italy, to take an example, was 12 and its value predicted from income was 17. Since predicted is higher than actual, the deviation is a favorable −5.

In figure 4, we show that some of the 30 most-populous nations are out-performing expectations and others are under-performing. On the x-axis, we show PPD in 2019 relative to per capita income for these 30 nations and for the world as a whole—negative values mean a country is doing better (has lower mortality) than expected based on its income. A few countries did exceptionally well (Bangladesh, China, Colombia, Iran), relative to their income, and a few seriously faltered (Kenya, Nigeria, Russia, the US). In 2019, China and the US had roughly the same PPD (figure 2), yet their performance against expectations was very different—China’s performance was better than expected and the US was worse than expected based on income. The y-axis shows how many years it would take to halve the PPD assuming the rate of improvement in PPD that these nations achieved from 2010–2019. Countries showing a high rate of improvement—i.e., relatively short projected times to halve PPD—include Ethiopia, Russia, and Tanzania, as well as, again, Bangladesh, China, and Iran. Countries that are in the green quadrant in the top right are notable in that both their PPD in 2019 was better than expected based on their income and they achieved an impressive rate of improvement in PPD from 2010–2019. In contrast, countries in the red quadrant in the bottom left had a PPD in 2019 worse than expected and they had a slow rate of improvement in PPD from 2010–2019. While multiple reasons underly good country performance, a common element seems to be investment in robust, community-based primary healthcare infrastructure.^16^

GH2035 provided estimates of the value, relative to their income levels, of the mortality declines experienced by countries.^2^ In estimating the economic value of such mortality decline, the report used a metric that it called “full income” valuation. Full income is an inclusive metric that captures two dimensions of the impact of better health on the economy: (i) economic productivity, as measured in a country’s national income accounts (the so-called “instrumental” value of better health, i.e., improved health as an instrument for greater productivity), and (ii) the “intrinsic” value of better health, in and of itself. When valued inclusively with this approach, “the inclusively measured economic benefits of improved health are shown to be decisively greater than when health is valued only by its effect on national income accounts.”^2^ GH2035 estimated the contribution of mortality decline to growth in full income for various regions from 2000–2011. In South Asia, for example, it estimated that the value of mortality decline in 2000–2011 was about 2.9% of average national income per year. This value was almost half as large as the value of increases in income levels during this period. The full income methodology from GH2035 has been used in investment cases for women’s and children’s health and the prevention and treatment of non-communicable diseases, among others.^17^ At the same time, contributions measured in the national income accounts are important. Healthy populations enable higher income levels for countries, faster economic growth, and more rapid poverty reduction, as was well documented in the report of the WHO Commission on Macroeconomics and Health.^18,19^

**Figure 5:**
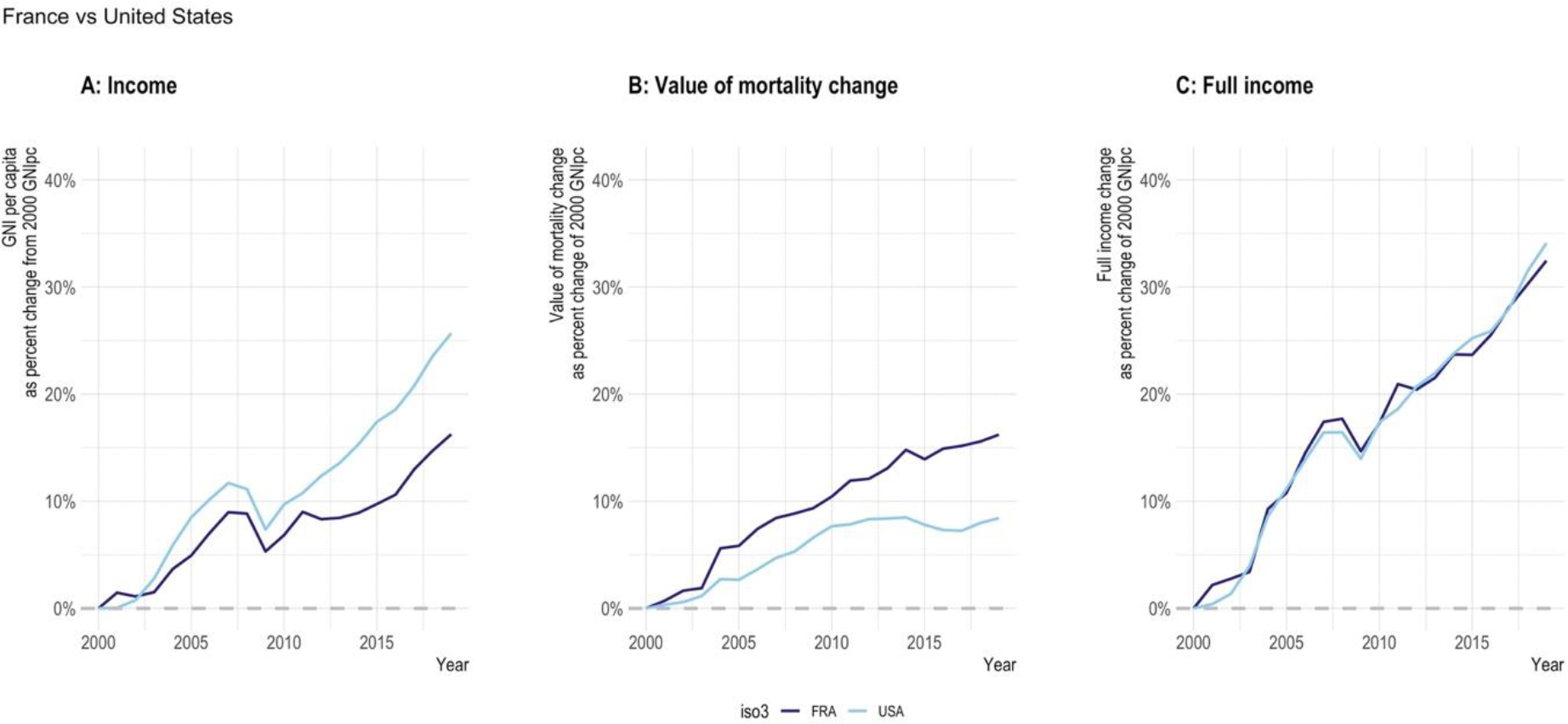
Percent change in income, value of mortality change, full income in France and the United States from 2000–2019 Source from reference 20 ^20^

For GH2050, we have brought these numbers up-to-date.^20^ Table 2 shows the value of income change, mortality change, and change in full income over the period 2010–2019 for the 30 most-populous countries, expressed relative to their 2010 income level. The table confirms the very large contribution of health to economic welfare, which is demonstrated further in figure 5, which compares the US and France over the period 2000–2019. While growth in income in the US exceeded that in France, the value of mortality change in France exceeded that in the US—with the result that the changes in full income were similar in both countries. Consistent with the findings, Chen et al have undertaken analyses concluding that countries underspend on health improvements relative to their value.^21^ The full income approach is one of the several ways to generate compelling evidence to the finance ministries and government planners, and we are in the process of understanding how this evidence is used (or not) and how it could be improved to better meet the needs of the target audience.^22^

#### 2020–23: COVID-19 strikes and tensions between powerful countries amplify

The period from 2020–2023 was marked by the enormous mortality and economic consequences of the COVID-19 pandemic, which have been well described in the literature, and which we discuss further in section 7. During the WHO’s definition of the emergency period of the pandemic—January 2020 through May 4, 2023—we estimate that about 26 million excess deaths occurred, mostly from COVID-19. Our 2021 analysis suggested that the pandemic would be a major setback for achieving global mortality targets, particularly tuberculosis and maternal mortality targets,^24^ though recent evidence suggests we were too pessimistic.

The impacts of the pandemic were compounded by conflicts in Europe, the Middle East, and west and east Africa, driving up direct and indirect civilian deaths, and by continued US-China rivalry, which are markedly altering the global political environment. No end appears in sight for these tensions. Conflicts are also driving increases in the number of refugees and internally displaced people, which are now at record highs, and which represent a challenging cohort for health service delivery. The consequences of these tensions, and from continued fallout from the pandemic, include increases in inflation, energy prices, food prices, and debt servicing; by the end of 2022, external debt of low- and middle-income countries reached about $27 trillion.^25^ Between 2022 and 2023, official non-concessional financial flows to developing countries dropped by almost $40 billion per year to actually become reverse flows (such flows, however, did substantially increase to Ukraine). Concessional flows barely rose, and private flows out of developing countries rose to about $190 billion per year.^26,27^ The International Monetary Fund recently argued that “higher long-term real interest rates, lower growth and higher debt will put pressure on medium-term fiscal trends and financial stability.”^28^

The changed environment will probably also lead to slower economic growth rates, tighter development assistance budgets, and less willingness of powerful countries to collaborate in addressing global challenges, including those to health (we discuss this further in section 9). For example, financing its contributions to the war in Ukraine is leading Germany to terminate strands of research in global health and Norway is reallocating development assistance budgets to Ukraine.

Geopolitical tensions, competition among multiple resources for limited assistance dollars, and political polarization are placing real strains on global health. In the US, for example, the President’s Emergency Plan for AIDS Relief (PEPFAR) is threatened by governmental dysfunction.^29^ Since its launch in 2003 until recently, PEPFAR was always reauthorized for five-year terms with strong bipartisan support. After a bruising partisan battle, in March, 2024, the US congress passed only a 12-month reauthorization bill, and the program’s future is in jeopardy. Rising nationalism presents a challenge to the agenda laid out in our report. For example, in Section 9 we argue for greater international collective action to generate global public goods, including pandemic preparedness and curbing antimicrobial resistance. We recognize that countries may find themselves having to generate these goods themselves–i.e., generate national public goods for health,^30^ or rely on support from regional initiatives such as Africa CDC.

#### Trends that will likely shape the global health response from 2024 to 2050

The global health response is likely to face a number of headwinds. Four of these are conflicts, discussed above, climate change, pandemics, and the demographic pressures on the cost and financing of healthcare. These demographic pressures are two-fold: (i) the inevitable ageing of populations increases demands for health services, and (ii) fertility decline leads to a relative decline in the size of the working age population, with its attendant implications for the capacity to finance and provide health services.

As discussed in panel 2, current estimates show climate change to have highly uncertain but conceivably large consequences for human mortality by 2100, although estimates are much smaller by 2050. The first of several Lancet commissions on climate change and health was published in 2009,^31^ and the Lancet publishes an annual independent countdown report that monitors the impacts of climate change on health (https://www.lancetcountdown.org/).

### Panel 2: Projecting temperature-related mortality impacts from climate change

Quantifying and projecting the health impacts of climate change is important both for planning climate adaptation and evaluating mitigation strategies.^32,33^ However, generating accurate estimates of these impacts is difficult due to three major practical and conceptual challenges.

First, climate change can affect health through a complex web of causal pathways, making it challenging to comprehensively capture the full health burden. Second, there are uncertainties in extrapolating from current climate-health relationships to future ones, including the potential limits of individuals and societies to adaptation.^34^ Third, there are difficulties in quantifying ambiguous risks where there is limited historical precedent and understanding of complex biophysical relationships. These risks include tipping points, defined by the Intergovernmental Panel on Climate Change (IPCC) as “critical thresholds in a system that, when exceeded, can lead to a significant change in the state of the system, often with an understanding that the change is irreversible,”^35^ as well as the potential effects of climate change on war and mass migration.

Despite these difficulties, several multi-disciplinary consortia have recently attempted to robustly quantify the mortality impacts of climate change.^36–38^ They aimed to update the social cost of carbon (SCC)—the net economic impact of one additional tonne of carbon emissions—which is widely used to evaluate climate policies.^39^ Mortality impacts, which have historically represented about 5% of the SCC,^36^ are an order of magnitude higher in these updated studies—for example, around 50% in Rennert and colleagues’ study,^38^ which was specific to the social cost of carbon.

The primary pathway that these recent studies evaluated is the effect of heat on mortality and, conversely, the benefit from reduced cold-induced mortality. Their focus on this particular pathway reflects its strong empirical grounding and perceived significance^37,38^ – in contrast to pathways through extreme weather events that are more uncertain and, to a certain degree, unknowable.^40^ Different pathways differ also in the extent to which compensatory behaviour can reduce mortality impact through that pathway.

These studies broadly involve two steps: first, assessing the current relationship between heat and mortality and, second, projecting this relationship in a hotter world.^36–38,41^ Exposure-response relationships are projected at a national and, increasingly, sub-national scale using climate models and scenarios developed for the Intergovernmental Panel on Climate Change.^36–38,41^ There is a “U-shaped” relationship, indicating an increased relative risk of mortality at low and high.^37,41^ Notably, larger effect sizes are observed in poorer, more densely populated, and warmer regions.^37,41^

**Figure 6:**
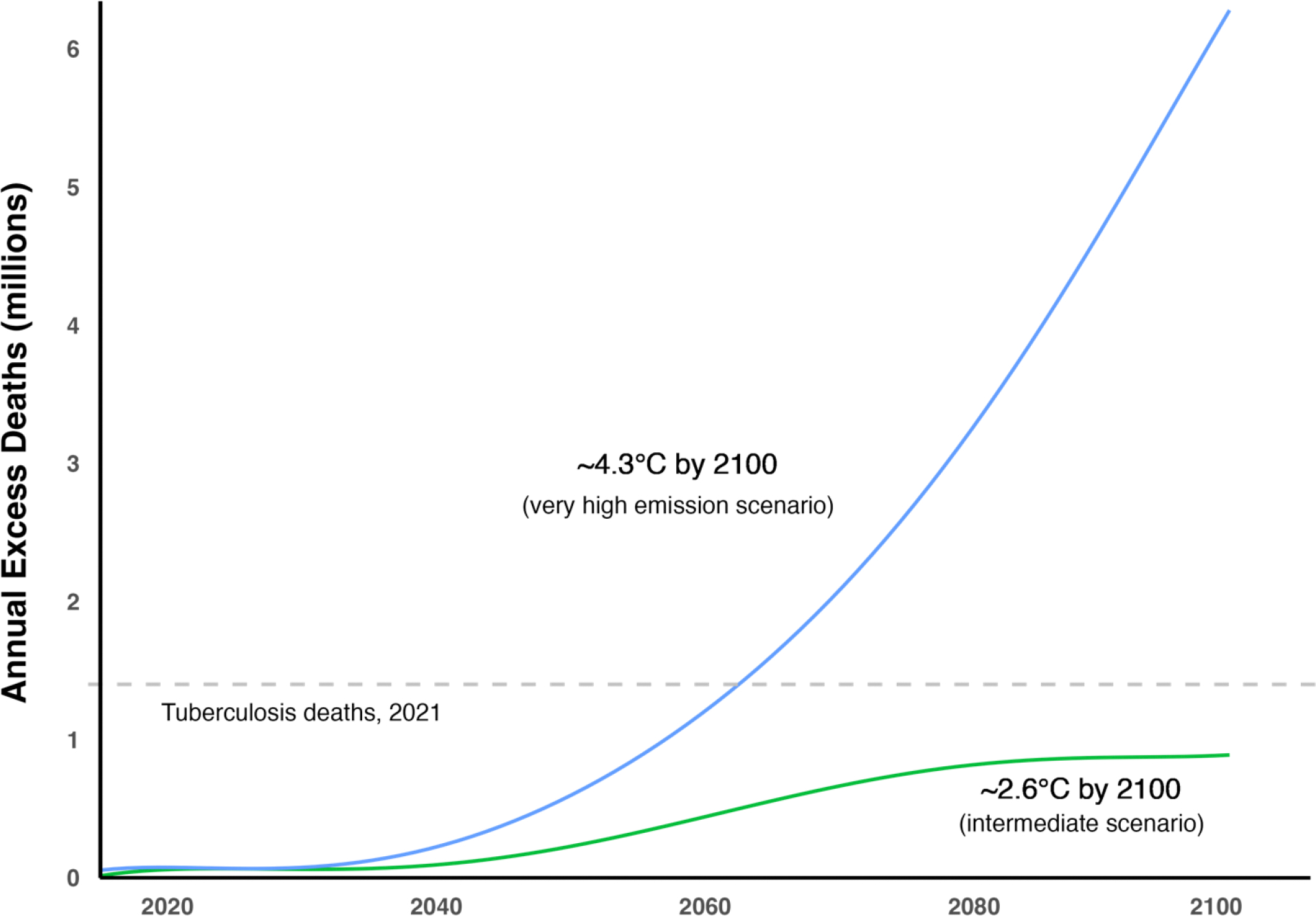
Projected increase in annual heat-related deaths due to climate change compared to the 2015 baseline Note: The graph presents estimates under two Representative Concentration Pathways (RCPs): RCP 4.5, a ~2.4°C increase in global temperatures by 2100 compared to 1850–1900, and RCP 8.5, a ~4.3°C increase, as per the IPCC Fifth Assessment Report from reference 42.^42^ As of 2024, global temperatures have already risen between 1.25°C and 1.5°C from 1850–1900 (Hausfather, 2024). Source from projections adapted from reference 37 ^37^

The IPCC laid out four scenarios for greenhouse gas emissions, called Representative Concentration Pathways: a stringent mitigation scenario (RCP2.6), two intermediate scenarios (RCP4.5 and RCP6.0), and a scenario with very high emissions (RCP8.5). Despite using different datasets and methodological approaches, the projections generated by the new studies under RCP4.5, i.e., a global temperature rise of about 2.5°C by 2100, are similar, with annual excess deaths ranging between 1.1 and 1.7 million in 2100 (Figure 6). Under RCP 8.5, i.e. a global temperature rise of around 4.3°C by 2100, projections in the different studies diverge, ranging between 2.4 and 7.3 million annual excess deaths in 2100. This divergence reflects differences in the studies’ assumptions, including the shape of the relationship between mortality risk and temperature^37,38^ and the inclusion of climate models accounting for low-likelihood, extreme climate outcomes.^37^

While there is significant uncertainty in the impacts, and relatively modest estimated impacts by 2050, these findings add to other arguments for limiting global temperature rise in line with the Paris Agreement—the international treaty to limit temperature rise to below 1.5–2°C, whose success will be critically shaped by decisions taken this decade.^43^ In the nearer term, i.e. up to 2050, many of the recommended measures to improve health systems and public health, such as investing in the health workforce, service delivery, and governance, and lifting people out of poverty, will help to mitigate the effects of heat.^44^ However, the broader impacts of climate change—including morbidity and mortality over the medium to long term—remain deeply uncertain, reflecting complex relationships between highly interdependent biophysical and social systems.^40^ It is therefore of value to continue research to characterise and identify ways of avoiding the remaining health risks, including the impacts of flooding, droughts, migration, and uncompensable heat stress (“the set of environmental conditions under which a healthy human being can no longer maintain a stable core temperature without the assistance of external cooling.”^45,46^ It will also be valuable to identify rapid pathways to net zero emissions globally that impose no unfair burdens on lower-income and recently industrialized countries.

A background analysis conducted for CIH 3.0 and the Disease Control Priorities project gives an assessment of future pandemic risk.^47^ The analysis concluded that the expected annual value of deaths from future pandemics is on the order of 2.5 million deaths per year (this is an average value; in most years the actual value will be zero, whereas in some years, as we saw with COVID-19, the number of deaths will be far higher). Another way to cast our current assessment of risk is that there are about even odds that a new pandemic causing 25 million or more deaths will occur between now and 2050.

Even today’s level of healthcare services will become costlier over time (setting aside the cost of new health technologies and services that will become available in the coming decades). The rising costs are related to increases in population size and average age, combined with the Baumol effect (rising salaries in jobs that see no productivity gains, such as teachers and many health workers, in response to rising salaries in other jobs that did see these gains, like manufacturing). Better paying opportunities outside the health sector and deteriorating work conditions often lead to a large gap between the supply of and demand for the health workforce. International migration compounds the pattern of rising costs in many lower-income countries. Higher wages for physicians and skilled nurses in upper middle-income and high-income countries create a combination of out-migration and upward pressure on domestic wages leading to doubly bad outcomes in many lower-income countries.

Further, while healthcare costs are rising, there has been a deterioration in public finance for health in many countries. Kurowski and colleagues at the World Bank recently noted that “the stark reversal in the priority given to health in government spending does not bode well for global health security and progress toward the health-related Sustainable Development Goals.”^48^ These challenges are compounded by the reverse capital flows described above.^27^

An important tailwind that could accelerate progress in global health and provide an important countervailing pressure to the 4Cs is the impact of new medicines, vaccines, diagnostics, and other health tools. Countries that adopt such new tools see an “accelerator effect” on their mortality decline. GH2035 noted that “historical experience suggests that the adoption of new technologies is associated with a decrease in the under-5 mortality rate of about 2% per year.” A study by Jamison et al^49^ found that around 80% of the decline in the under-5 mortality rate across 95 low-income and middle-income countries can be explained by the diffusion of such technologies. Today, the pipeline of candidate medicines, vaccines, and diagnostics for neglected diseases, emerging infections, and child and maternal health is more robust than ever, and recent product launches are having a transformative impact (panel 3). New research by Schäferhoff and colleagues,^50^ and by Ogbuoji and colleagues,^51^ suggests that the current pipeline is very likely to yield a suite of new tools that could have a dramatic global health impact.

### Panel 3: New tools for prevention and treatment of tuberculosis

Globally, tuberculosis is now the number one infectious disease killer, causing about 1.3 million deaths in 2022;^32^ it is a major cause of premature mortality in high-burden nations. Last year, the *Lancet* Commission on Tuberculosis published its report “Scientific advances and the end of tuberculosis,” which argued that a new set of tuberculosis control technologies provided grounds for optimism in reaching a tuberculosis-free world within a generation.^52^ Such tools, it argued, would need to be adopted wholesale, implemented at scale, and accompanied by sustained investment in development of new tools and in tuberculosis programmes for this vision to be achieved. These tools fall into three categories: diagnostics, therapeutics, and preventive interventions.

#### Diagnostics.

Diagnosis, say Pai and colleagues, is “the weakest aspect of TB care and control”—without diagnosis, the disease cannot be treated.^53^ Prior to the COVID-19 pandemic, around 2.9 million people annually had tuberculosis without being diagnosed, and the annual diagnostic gap grew to 4.2 million during the pandemic. Using new diagnostics along the case finding “cascade”—for those with subclinical tuberculosis, those with symptoms who have not sought care, and those who have sought care but are undiagnosed—could reap large benefits.^52^ New molecular diagnostics are now available that can be used at the point of care (POC) in decentralized settings and the WHO recommends molecular diagnosis over sputum smear microscopy as the preferred frontline testing option. Given the limitations of using sputum, especially in children and people with HIV, efforts are now underway to develop molecular diagnostics based on tongue swabs, urine, blood, and stool, and to develop multi-disease tests. “A simple, non-sputum sample,” say Pai et al,^53^ “combined with an affordable, multi-disease POC molecular technology, deployed in decentralized settings would reach a much larger population, close the case detection gap, and curb TB transmission at the population level.” Screening for tuberculosis and latent tuberculosis have been made more feasible with the advent of interferon-gamma release assays (IGRAs), which have many advantages over tuberculin skin tests, and the C-tb skin test. The C-tb test is now being mass produced by the Serum Institute of India and has a sensitivity and specificity close to that of the IGRA assay at one tenth of the cost.^54^

#### Therapeutics.

There have been striking advances over the last 5 years in the treatment of tuberculosis, including one-month regimens for tuberculosis prevention, a reduction in the duration of treatment of drug-susceptible tuberculosis down to 4 months, and the approval of 6-month treatment regimens for drug-resistant tuberculosis. These advances are now promoted by the 1/4/6×24 (one, four, six by 2024) campaign, launched at the AIDS 2022 conference in Montreal (one month regimen for prevention; four month regimen for sensitive disease; six month regimen for resistant disease). The WHO and the US Centers for Disease Control and Prevention recently approved the 4-month isoniazid–rifapentine–moxifloxacin–pyrazinamide (4HPMZ) regimen for eligible people with drug-susceptible tuberculosis, which “speaks to the momentum of the development of new treatments for drug-susceptible tuberculosis.”^52^ The 2023 TRUNCATE-TB trial suggests that even a two-month regimen for drug-susceptible tuberculosis may be feasible.^55^ For multidrug-resistant and rifampicin-resistant tuberculosis, we are in a “golden age of innovation.”^52^ In particular, in 2022, the WHO codified a six month regimen of bedaquiline–pretomanid–linezolid augmented with moxifloxacin (6BPaLM) in its guidelines as the preferred regimen for adults and adolescents aged 14 years and older. Long-acting injectable drugs (“depot formulations”) are currently being studied for both preventive therapy and treatment of active disease. Such formulations could improve adherence and significantly ease the burdens associated with long-term daily pill intake, both for individual patients and for public health systems.

#### Preventive interventions.

Reducing tuberculosis incidence will require aggressive scale-up of tuberculosis preventive treatment (TPT) to those at highest risk of latent tuberculosis infection, such as people living with HIV and household contacts younger than 5 years. An estimated 1.7 billion have tuberculosis infections that are “latent.”^56^ While latent tuberculosis infection causes no actual harm and cannot be spread, there is a substantial risk that such latent infections can become active. TPT addresses this population. The advent of short-course TPT (e.g., 12 weeks of once-weekly isoniazid and rifapentine [3HP] or 1 month of daily isoniazid and rifapentine [1HP]) makes scale-up more feasible. Last year, India committed to expanding 3HP nationwide; for other countries to follow suit, licensing and cost barriers will need to be addressed. On the preventive vaccine front, progress has been greatly hindered by lack of funding. This lack is a key reason why it took over 19 years for one promising candidate, M72, a fusion protein of two *M tuberculosis* antigens administered with a potent adjuvant, to move from early clinical development studies to a phase 3 trial. Two other vaccine candidates are also now in late stage trials: (i) VPM 1002, a next-generation, genetically modified BCG vaccine, and (ii) MTBVAC, an *M tuberculosis* strain attenuated via two genetic mutations.

## Section 3. Health goals for 2035 and 2050

Health systems serve several important goals. These include preventing and reducing the incidence and severity of disease and improving quality of life at all ages, reducing mortality rates, responding to day-to-day health concerns of the population, and protecting populations from the level and risk of expenditures on health services. Most countries also explicitly value equity in access to services and the attainment of health outcomes.

However, multiplicity of goals can lead, in effect, to absence of actionable goals. To address this problem, GH2050 advances the argument that reduction in the rate of premature mortality—the probability that members of a country’s population die before age 70—serves well as an overarching goal to bring more coherence and focus to these efforts. Panel 1 describes the key metric that we use in this report: PPD. Other goals correlate well with achieving reductions in premature mortality and, of course, focusing most effort on a single goal in no way precludes other efforts, such as improving quality of life and reducing within-country inequalities (Panel 4).

### Panel 4. Inequalities in mortality within countries by sex and socioeconomic status

“Inequality in length of life is the most fundamental of all inequalities; every other type of inequality is conditional upon being alive,” say van Raalte and colleagues.^57^ GH2050 uses national averages of PPD as the key metric, but these may mask within-country inequalities in mortality by sex and socioeconomic status.

#### Sex differences in health outcomes

Sex and gender are important determinants of health outcomes.^58^ GH2035 reported the sharp contrast between faster mortality improvements in adult females than males and discrimination against girls at birth and in under-five mortality in some countries. It took a different approach of looking at sex differences in rates of decline rather than levels of mortality, and concluded that much of the overall improvement in survival was driven by improvements in females. GH2050 returns to this conclusion. The “50 by 50” goal is one for both sexes combined, and, as previously noted, 8 of the 30 most populous countries reduced their PPD during the 2010–2019 period at a rate that would halve their PPD before 2050. Figure 9 shows that, considering females only, 10 of the 30 countries were on track, whereas for males, only 3 countries were on track (Tanzania, Russia, and the Republic of Korea). The three countries with the greatest advantage for females are Bangladesh, Thailand, and Ethiopia whereas Spain, France, and Italy had the greatest advantage for males. Overall, 21 of the 30 most populous countries experienced greater declines for females whereas in only 9 did males do better.

GH2050 finds patterns suggesting discrimination against females in some countries (such as India, Malta, and Nigeria) and against males in others (such as Central and Eastern European countries, Thailand, and Vietnam). Globally in 2019, females had a lower PPD than males in all countries. This gap was the widest in Central and Eastern European countries, where male PPD was up to 2.5 times higher than female PPD. Other countries with male PPD twice that of females include Greece, Japan, Mongolia, Portugal, Republic of Korea, Sri Lanka, Spain, Thailand, Turkey, and Vietnam. Beyond the female biological advantage (accounting for about 25% of the sex difference^59^), the remaining sex differences can be attributed mostly to higher risk exposure among males, most notably smoking; there is growing evidence on ways to reach men with interventions that can reduce such exposure.^60^

Conversely, our analysis found the smallest sex differences in PPD in a mix of countries from sub-Saharan Africa, Western Pacific and Southeast Asia, and the North Atlantic: Nigeria (male PPD only 1 percentage point (pp) higher than females); Bahrain (4pp), Côte d’Ivoire (4 pp), Maldives (4 pp), Netherlands (4 pp), Togo (4 pp), Qatar (4 pp); Guinea (5 pp), Norway (5 pp), and Sweden (5 pp). Our calculations in other age groups found that the male PPD was higher in all age groups everywhere, except for females under 15 years in India and Malta.^14^ Given that females generally have a five-year advantage in life expectancy, smaller survival differences may indicate discrimination against females. A background paper prepared for GH2050 discusses sex disadvantages in more detail.^61^

Although they live longer, females generally have higher rates of disability and poorer health than males, known as the health-survival paradox.^62^ Females also face higher age-specific rates of mental illnesses, dementia, and some of the indicators of failure in child development. However, the lack of sex-disaggregated data on disease prevalence and other morbidity indicators, as well as on access to healthcare and other essential services, severely constrains our understanding of these sex differences. These data gaps also make it harder to design sex- and gender-responsive programmes and policies to reduce these inequalities.

#### Socioeconomic inequality in survival

GH2035 pointed to the health of vulnerable groups in low- and middle-income countries as one key health challenge, and highlighted that avoidable mortality is concentrated disproportionately in poorer communities.

Historically, higher life expectancy was associated with lower lifespan variation, i.e., lower inequality in the length of life lived in a population.^63,64^ However, recent trends in high-income countries show widening gaps between the richest and the poorest individuals. The gap in age at death between the richest and poorest 1% of individuals in the US between 2000–2014 was about 15 years in males and 10 years in females.^65^ Similar findings were reported in Norway.^66^ The gap has widened in some countries, such as the US, UK, and Denmark,^67–69^ and narrowed in others, such as South Korea and many European countries.^70,71^

In low-income and middle-income countries, more attention has been paid to studying inequality of childhood mortality by socioeconomic groups. Chao et al estimated that in 2016 the under-5 mortality rates were twice as high in the poorest households than the richest in low- and middle-income countries (excluding China). ^72^ Despite significant absolute reductions in the gap since 1990, the relative gap remained similar. Key drivers of such inequalities include rural-urban residence, maternal education, sex of the child, and source of drinking water.^73^

In comparison, inequality in adult mortality in low- and middle-income countries has received much less attention. A study in five countries in sub-Saharan Africa showed a 6–10-year difference in life expectancy between the lowest and highest socioeconomic groups between 2003–2016.^74^ In India, an eight-year gap was noted between the richest and poorest quintiles in 2011–12,^75^ and in Indonesia, a four-year difference in life expectancy at age 30 between 2007–2015.^76^ Some studies concluded that the relationship between socioeconomic status and adult mortality in low- and middle-income countries may differ from that of high-income countries, due to different patterns of epidemiological and demographic transitions, including rates of NCD multimorbidity and rates of tobacco and alcohol use.

Taking the global average in the PPD, a person born somewhere in the world in 2019, just before the COVID-19 pandemic, had about a 31% chance of dying before age 70 (assuming continuation of the age-specific mortality rates then prevailing). Figure 2 shows that this 31% in 2019 was about half of what the PPD was in the early 1960s, when it was 62%. Analyses underpinning the GH2050 report conclude that, for most countries, it would be technically and financially feasible to reduce their own current PPD by 50% before the year 2050.^14^ A reasonable long-term goal might then be for countries, or provinces within countries, to reduce the probability of premature mortality from 2019 levels by 50% before 2050. We call this goal “50 by 50.” Achieving the “50 by 50” goal for the world as a whole would imply that a person born somewhere in the world in 2050 would have only a 15% chance of dying before age 70—the level that was seen in the North Atlantic in 2019.

Why did we choose 2019 as a baseline? COVID-19 had a substantial impact on PPD and shows how exogenous shocks like pandemics can threaten “50 by 50.” Although COVID-19 deaths were highly skewed towards the oldest age groups, over one third (36%) of all excess deaths worldwide in 2020–2021 were among those under 65 years.^77^ From 2019 to 2021, the worldwide PPD rose by over 4 percentage points.^11^ However, the COVID-19 pandemic was presumably a temporary setback to mortality declines over the long run: data suggest that in 2023 mortality started falling again, although it was still not down to pre-pandemic levels in many countries.^78^ We used 2019 as the baseline year to avoid these temporary distortions in the overall trend due to the pandemic.

#### Time required to halve premature death

Our assessment began by looking at historical progress in reducing the PPD over the last half century, from 1970–2019. We found remarkable improvements over that period, but also disparities across regions and countries in levels of and trends in PPD.

**Figure 7:**
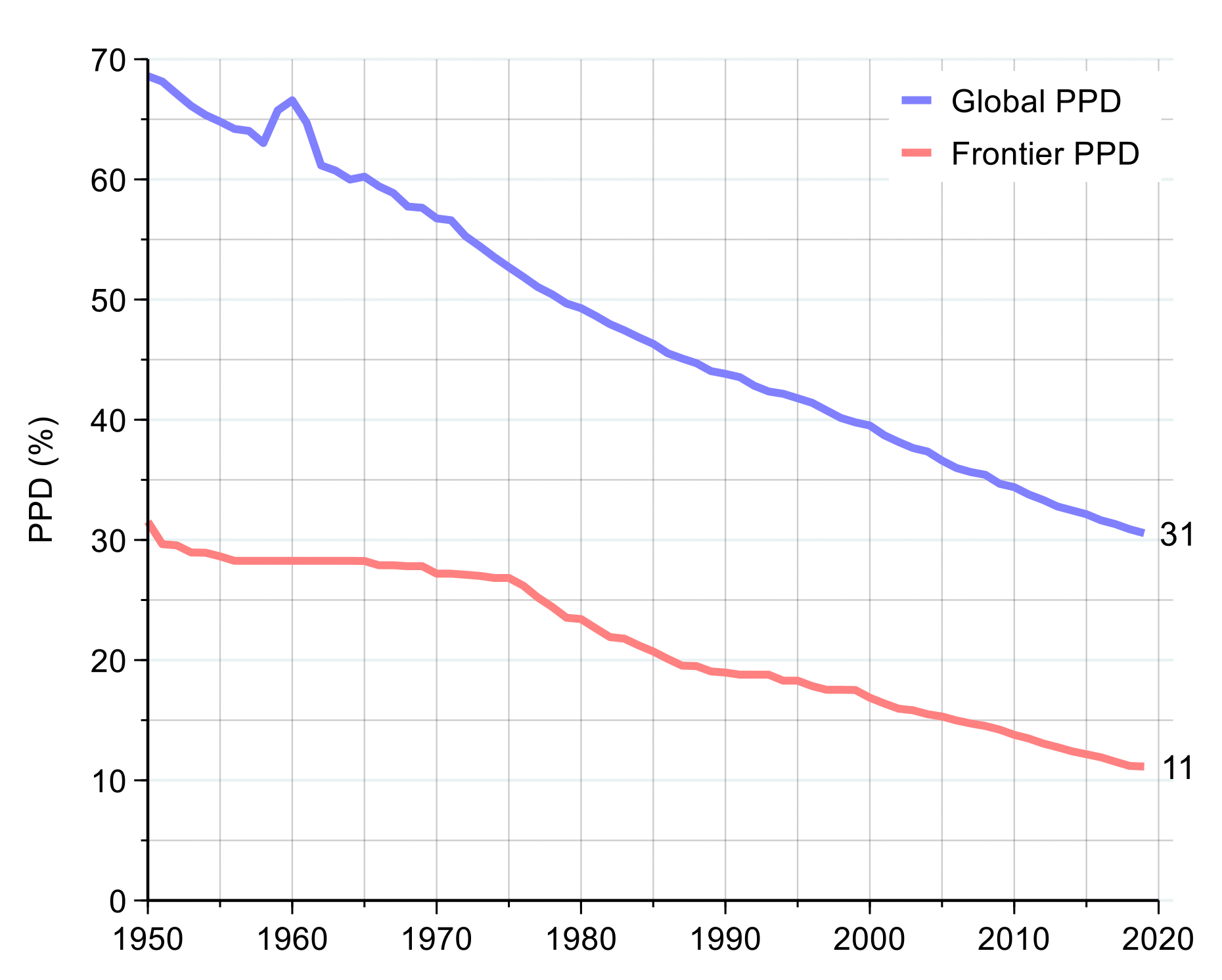
Global and frontier probability of premature death (PPD), 1950–2021 Note: Probability of premature death (PPD) was defined as dying before age 70 years. The frontier is the lowest PPD ever observed. Countries with a population below 3 million in 2019 were not considered for being a frontier. Data source from reference 11 ^11^. Source from reference 79 ^79^

In 2019, PPD ranged from 52% in sub-Saharan Africa to 15% in the North Atlantic, with the other regions falling in between (appendix table 2.2 on page 6 of the appendix). Of particular interest is the relatively high rate of change seen in China (PPD declined at a rate of 1.9% per year) and an actual increase in PPD in the US in the period 2010–2019. Figure 7 plots the “frontier” PPD for the period 1970–2019, as well as the world average. The frontier is defined as the value of PPD in the country with the lowest PPD at that time. The figure shows a clear, if modest, convergence of the world average toward the frontier.

Among the most populous countries, Bangladesh, Republic of Korea, Russia, and Tanzania had the highest rates of decline (table 1). In table 1, the rate of decline in PPD in the period 2010 to 2019 is indicated by how long it would take for PPD to decline by half (the “50 by 50” goal) if the 2000–2019 rate were to continue. In Russia, it would take 26 years to fall by half whereas in the United Kingdom it would take over twice as long (58 years). Importantly, there is no statistically significant correlation between current levels of PPD and rates of change in the last decade (2010–2019).^14^ That is, high rates of decline in PPD are possible regardless of the initial PPD level—for example, the Republic of Korea had the most rapid improvement in PPD from a low initial PPD while Tanzania also had a rapid improvement in PPD despite a high initial level (table 1).

For the world as a whole, changes in PPD since 1970 have largely been driven by improvements in ages 50–69 years.^14^ About 50% of the improvement in PPD was due to reduced mortality in this age group, followed by reduced mortality in ages 0–14 years (about 27%) and ages 15–49 years (about 23%). In the North Atlantic, the proportion of the contribution to the decline in PPD from ages 50–69 years has been about 70% since the 1970s, and even in sub-Saharan Africa this age group contributed the most (40%) to changes in PPD in the period 2010–19. Success in reducing PPD will require success in reducing the burden of the NCDs and injuries that dominate the causes of mortality at middle and older ages.

#### Feasibility of achieving “50 by 50”

**Figure 8:**
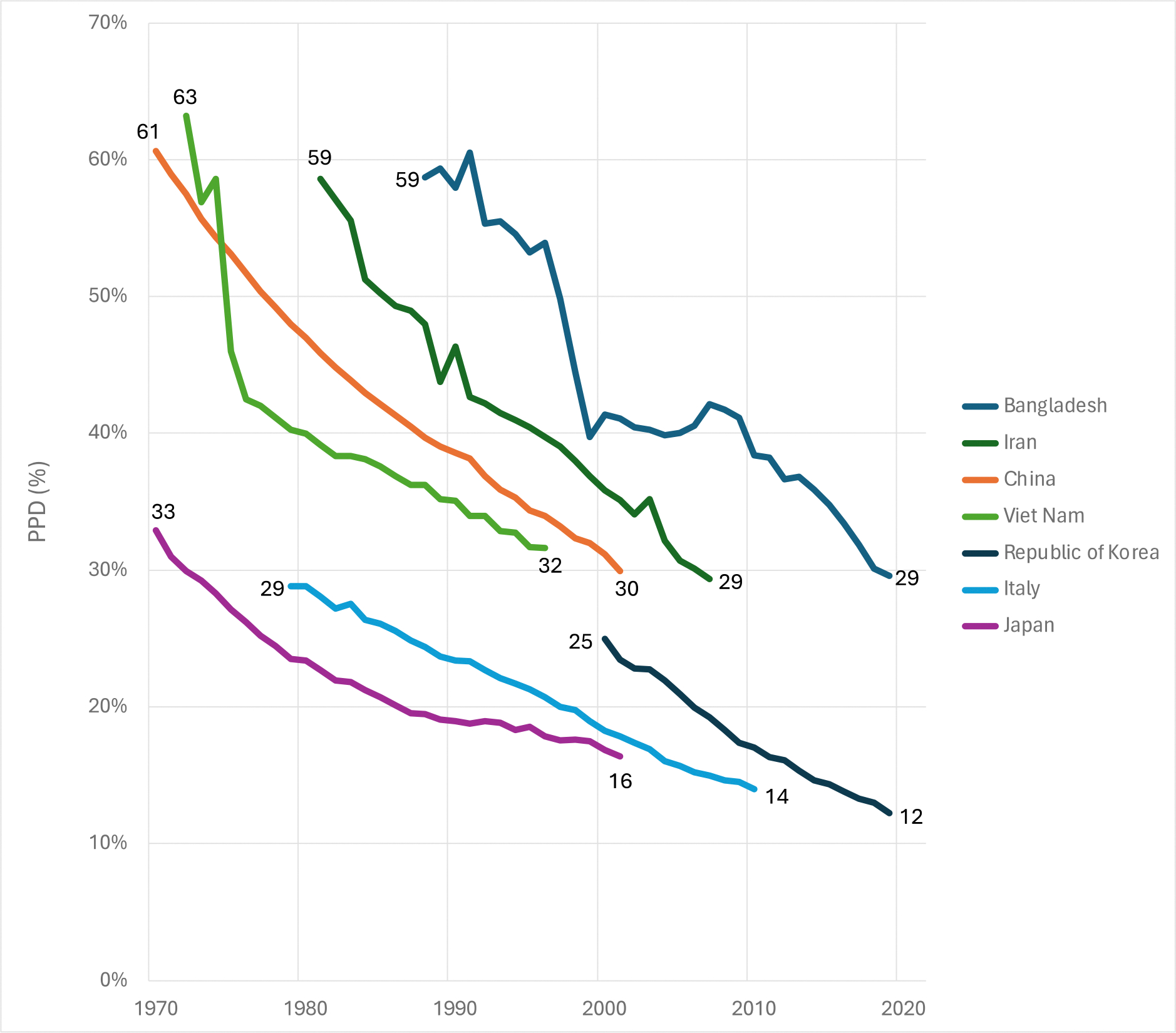
Seven high-population countries that achieved a halving of PPD in the last half century over 31 years or less Notes: PPD = Probability of premature death Source from reference 14 ^14^ Data source from reference 11 ^11^

**Figure 9:**
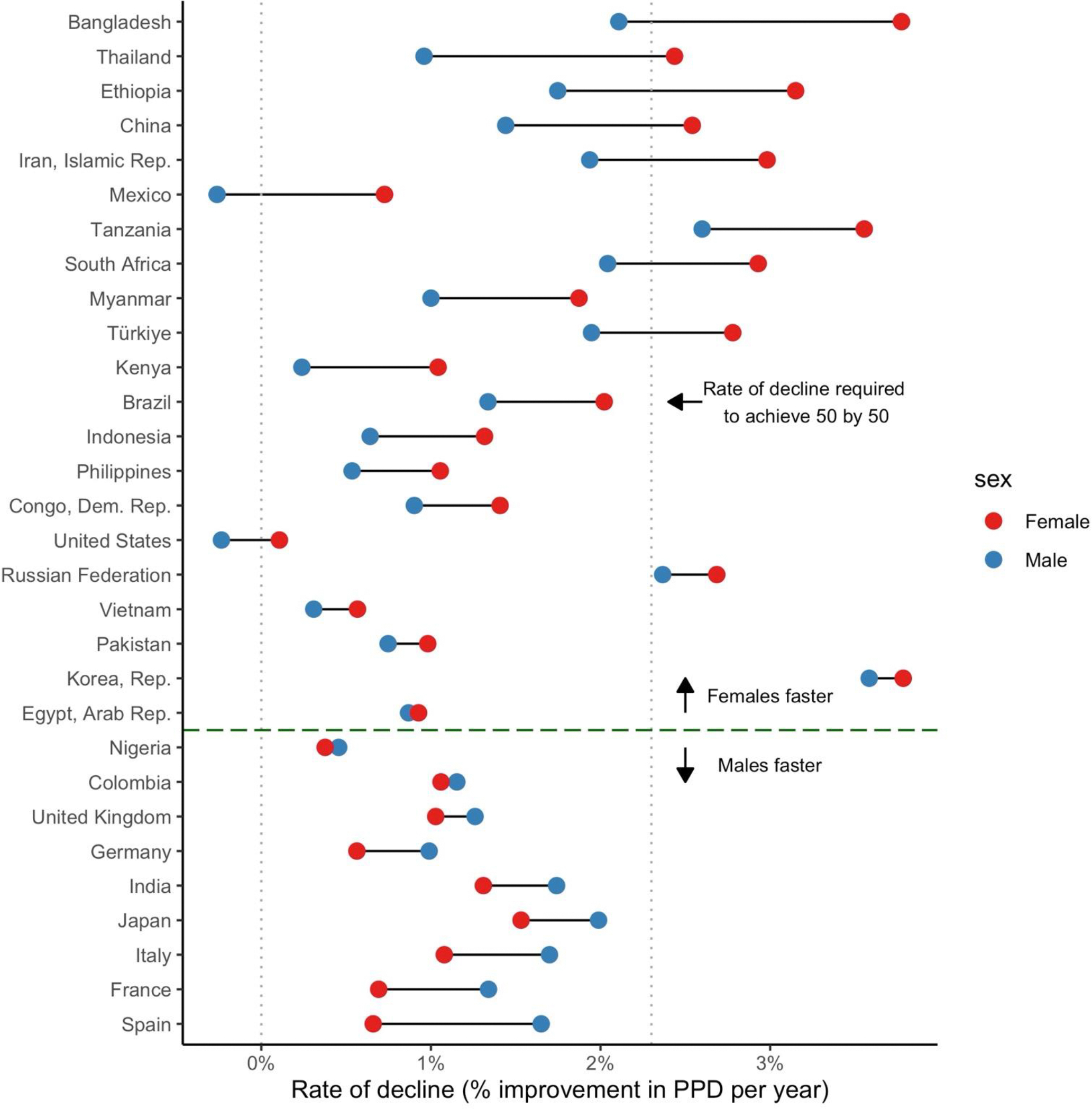
Sex differences in the rate of decline in PPD, 30 most populous countries, 2010–19 Source from UN World Population Prospects 2024

As mentioned earlier, “50 by 50” requires countries to halve their PPD over a 31-year period from 2019 to 2050. Among all countries in the world, 37 countries achieved a halving of PPD in the last half century over 31 years or less (Table 3). Among the 30 most populous countries, seven countries made this remarkable achievement: Bangladesh, China, Iran, Italy, Japan, Republic of Korea, and Viet Nam (Figure 8).

This historical achievement in these countries shows that halving PPD in three decades or less is feasible. Halving occurred from both high starting levels of PPD (e.g., Viet Nam) and from low starting levels (e.g., Italy). With targeted investments in tackling the 15 priority conditions discussed in section 4, together with acceleration in progress from new health technologies, it is realistic for most countries to achieve “50 by 50” if they make the commitment to do so.

##### Countries that are currently on or off track to reach “50 by 50”

In the decade before COVID-19 (2010–2019), globally PPD declined at a rate of 1.4% per year for both sexes combined. The needed annual rate of decline to reach halving of PPD by 2050 is the same for all countries: 2.2%. Globally, 33 countries had an annual rate of decline equal to or better than 2.2% from 2010–2019.^14^ Of the 30 most populous countries, eight countries had an annual rate of decline equal to or better than 2.2% from 2010–2019 (Table 1). This means that in these eight countries, continuing this trend (“business as usual”) would lead to a halving or more in PPD if sustained, though an acceleration in progress would be needed for the other 22 countries. At the other end, 10 out of the 30 most-populous countries had rates of decline in PPD of less than 1% per year. For these countries to achieve “50 by 50,” more than a doubling of the rate of improvement would be needed.

If countries with a medium rate of change (between 2.2 and 1.0%) can achieve the same rate of change as well-performing regional neighbours, halving premature death by 2050 is feasible. This improvement would require scaled up investments in the 15 priority health conditions discussed in section 4 and in rolling out new health tools. As discussed in sections 4 and 6, such countries could achieve these targets by assigning higher priority for health in government spending, including for preventive health interventions, and having governments use subsidies and pooled procurement to ensure access to drugs, vaccines, and commodities that target the 15 priority conditions.

At a rate of decline of 2.2% per year, PPD would fall by 30% by 2035. Thus, a reasonable milestone on the way to the 2050 target would be to reduce PPD from 2019 levels by 30% before 2035 (the goal of “30 by 35”). Table 4 shows the countries that had rates of decline in PPD on track to achieve a 30% reduction by 2035 and a 50% reduction by 2050. For example, the table shows that at its current high rate of improvement, Tanzania would achieve a 30% reduction in PPD by 2030 and a 50% reduction by 2041.

##### Variation in baseline PPD

Countries start with PPDs at baseline (2019) from as low as 12% (Italy, Japan, Korea) to over 50% (the Democratic Republic of the Congo, Nigeria). While “50 by 50” as a goal appears feasible (if aspirational) across the range of starting points, the conditions and age groups that need to be addressed will vary according to starting level.

As we discuss in section 4, most premature deaths are explained by a narrow set of 15 priority conditions, so focused attention to tackling these conditions will have an enormous payoff. The varying importance of each condition across countries can help tailor interventions to achieve “50 by 50”. For example, just eight infections and maternal and child health conditions account for over half the life expectancy gap between the North Atlantic—the region with the highest life expectancy—and sub-Saharan Africa, where lower respiratory infections, tuberculosis, and neonatal conditions are particularly important. Countries that have reached low mortality from these eight infections and maternal and child health conditions can still substantially reduce their premature mortality by carefully focusing on only seven sets of NCDs and injuries. For example, these seven NCDs and injuries account for four-fifths of the life expectancy gap between the North Atlantic and China, with over half that gap accounted for by just three conditions: atherosclerotic cardiovascular diseases (CVDs), hemorrhagic stroke, and tobacco-related NCDs. India is an example of a country that is in between sub-Saharan Africa and China, having almost a third of the life expectancy gap accounted for by the eight infections and maternal and child health conditions (especially neonatal conditions and diarrhea) and almost half by the seven NCDs and injuries (especially atherosclerotic CVDs and tobacco-related NCDs). In all cases, focusing on a limited number of conditions will address a significant fraction of premature mortality.

## Section 4: 15 priority conditions account for most health disparities

To address the medium and long-term goals described in section 3—a 30% reduction in PPD by 2035 and a halving of PPD by 2050—we show in section 4 that just 15 conditions account for a very large fraction of the life expectancy gaps between the highest performing regions and others. Further declines in death rates from these conditions contributed the majority of the life expectancy gains globally between 2000 and 2019.^80^ GH2050 defines them as the 15 priority conditions, comprising eight infections and maternal health conditions (the “I-8,” panel 5) and seven NCDs and injuries (the “NCD-7,”) (panel 5). We propose that countries focus on preventing and treating these 15 conditions as a concrete step towards reaching “50 by 50.”

### Panel 5: 15 priority conditions

This report has proposed that all countries focus on reducing mortality and morbidity from 15 priority conditions, which include eight infections and maternal health conditions (the “I-8”) and seven NCDs and injuries (the “NCD-7”). I-8 is defined using the WHO Global Health Estimates (GHE) categories of country-level causes of death: neonatal conditions, lower respiratory infections, diarrheal diseases, HIV/AIDS, tuberculosis, malaria, childhood cluster diseases, and maternal conditions.^15^ The neonatal conditions comprise the GHE categories of preterm birth complications, birth asphyxia and birth trauma, neonatal sepsis and infections, and “other neonatal conditions” (e.g., hemorrhagic and hematological disorders of the newborn, transitory endocrine and metabolic disorders specific to the newborn, and digestive disorders of the newborn). The GHE category of childhood cluster diseases comprises four vaccine-preventable illnesses: whooping cough, diphtheria, measles, and tetanus.

The NCD-7 includes atherosclerotic cardiovascular diseases (ischemic heart disease and ischemic stroke); hemorrhagic stroke; NCDs strongly linked to infections; NCDs strongly linked to tobacco use; diabetes (diabetes mellitus and chronic kidney disease due to diabetes); road injury; and suicide. The NCDs strongly linked to infections are stomach cancer, liver cancer secondary to hepatitis B, liver cancer secondary to hepatitis C, cervical cancer, rheumatic heart disease, cirrhosis due to hepatitis B, and cirrhosis due to hepatitis C. The NCDs strongly linked to tobacco use are mouth and oropharynx cancers (lip and oral cavity, nasopharynx, other pharynx); trachea, bronchus, and lung cancers; larynx cancer; and chronic obstructive pulmonary disease.

**Figure 10:**
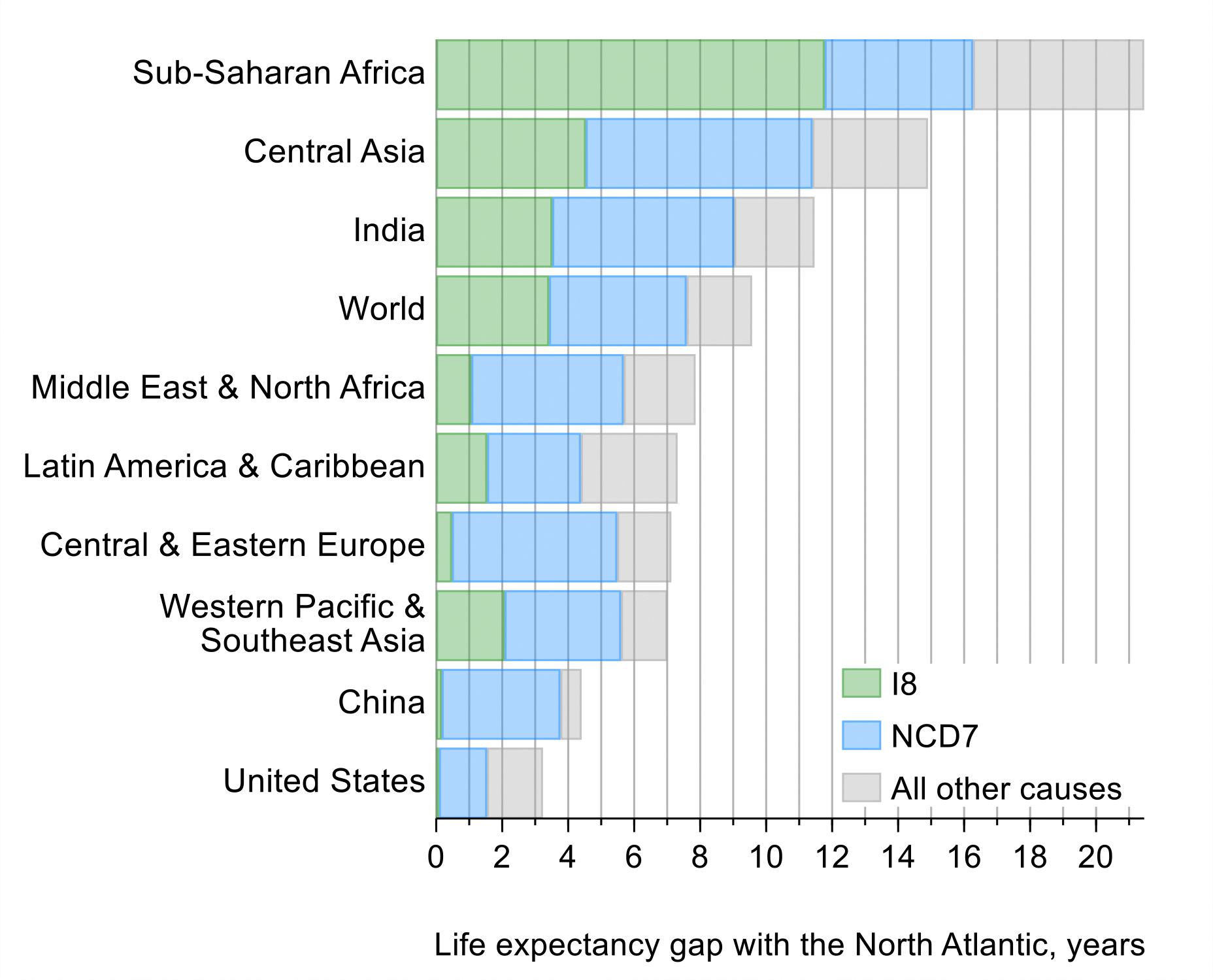
Life expectancy gap compared to the North Atlantic region attributable to priority conditions, 2019 Note: Life expectancy in the North Atlantic was 82 years in 2019. The priority conditions are I-8 plus the NCD-7, as defined below. The priority infections and maternal health conditions (I-8) are neonatal conditions, lower respiratory infections, diarrheal diseases, HIV/AIDS, tuberculosis, malaria, childhood-cluster diseases and maternal conditions. The priority NCDs and injuries (NCD-7) are atherosclerotic cardiovascular diseases, hemorrhagic stroke, NCDs strongly linked to infections, NCDs strongly linked to tobacco use, diabetes, road injury and suicide. Source from reference 80 ^80^ Data from reference 11 and 15.^11,15^

First, we look at the life expectancy gap of each CIH region against the best performing CIH region, which in 2019 was the North Atlantic, with a life expectancy at birth of 82 years and the lowest PPD (15%). The gap in life expectancy between each CIH region and the North Atlantic region in 2019 varied considerably, from 21 years in sub-Saharan Africa to 3 years in the US. Only a tiny fraction of the 17,000 entries in the International Classification of Diseases 11^th^ edition (ICD-11) accounts for the great majority of the health gaps. The 15 priority conditions contribute to about 80% of the life expectancy gap between most regions and the North Atlantic, e.g., 85% of the 4.4-year life expectancy gap between China and the North Atlantic, and 76% of the gap between sub-Saharan Africa and the North Atlantic (see figure 10 and table 5).

Second, we compared life expectancy gains for each region over time. Globally, life expectancy increased by 6.3 years between 2000 and 2019. Changes in the cause-specific mortality rates of the 15 conditions accounted for about 86% of this increase (table 6). In sub-Saharan Africa, these 15 priority conditions contributed to 89% of the 10-year gain in life expectancy during this period (84% was due to reductions in mortality from the I-8 conditions). In India, China, and the North Atlantic, they contributed to 85%, 76%, and 90%, respectively, of the life expectancy gain.

We focus on mortality trends because they are relatively well documented and, more importantly, they correlate highly with morbidity. Interventions against the main causes of mortality have highly significant morbidity reducing consequences. Nevertheless, psychiatric disorders, old-age dementias, and a few other low-mortality but high-morbidity conditions cause immense human suffering and place great demands on health systems. Additionally, in many parts of the world, large numbers of children and adolescents have fallen behind expected norms of physical and cognitive growth as a result of a history of infection and inadequate diet. We discuss these low-mortality problems briefly in this section and intervention modules to address them in section 5.

Below, we examine recent progress that countries have made in tackling the I-8 and the NCD-7 and address the question of whether generally positive trends early in the century continued in the years prior to and continuing into the COVID-19 pandemic years (2020–2023).

#### Progress in reducing mortality from infections and maternal health conditions

A major conclusion of GH2035 was that the high rates of mortality from infections and maternal health conditions could be reduced by 2035 to the low rates seen in the best-performing upper middle-income countries. Our new analysis of the reduction in the life expectancy gap between each region and the North Atlantic from 2000 to 2019 shows that there was significant progress in narrowing the gap, largely due to reducing mortality from infections and maternal health conditions in some regions (appendix table 4.1 on page 17 of the appendix).

Globally, decline in mortality from the I-8 contributed 3.5 years of the total 6.3 year increase in life expectancy 2000–2019 (Table 6). In sub-Saharan Africa, the overall increase in life expectancy was 10 years, of which 8.5 years were accounted for by the I-8, with declines in HIV/AIDS mortality accounting for the largest share, or 2.9 years. The I-8 accounted for 7.4 of the 8.2 year life expectancy increase in India 2000–2019, where decline in mortality from diarrheal disease, neonatal conditions, and tuberculosis were particularly important. Changes in I-8 were an important driver for life expectancy gains for sub-Saharan Africa, India, Central Asia, Western Pacific and Southeast Asia, and Middle East and North Africa. It is important to note that by 2000 much of the world had already experience major gains from the control of I-8 conditions so that a longer perspective would show greater significance even than Table 6 documents. For example, the Expanded Programme on Immunization (EPI) marks its 50th anniversary in 2024 and a retrospective assessment of impact estimated that 40% of the post-1974 decline in infant mortality resulted from the EPI.^81^ Figure 2 and Table 6 suggest limited recent and remaining gains from tackling childhood cluster conditions (vaccine preventable conditions), suggesting substantial earlier gains from immunization.

##### The unfinished I-8 agenda

**Figure 11:**
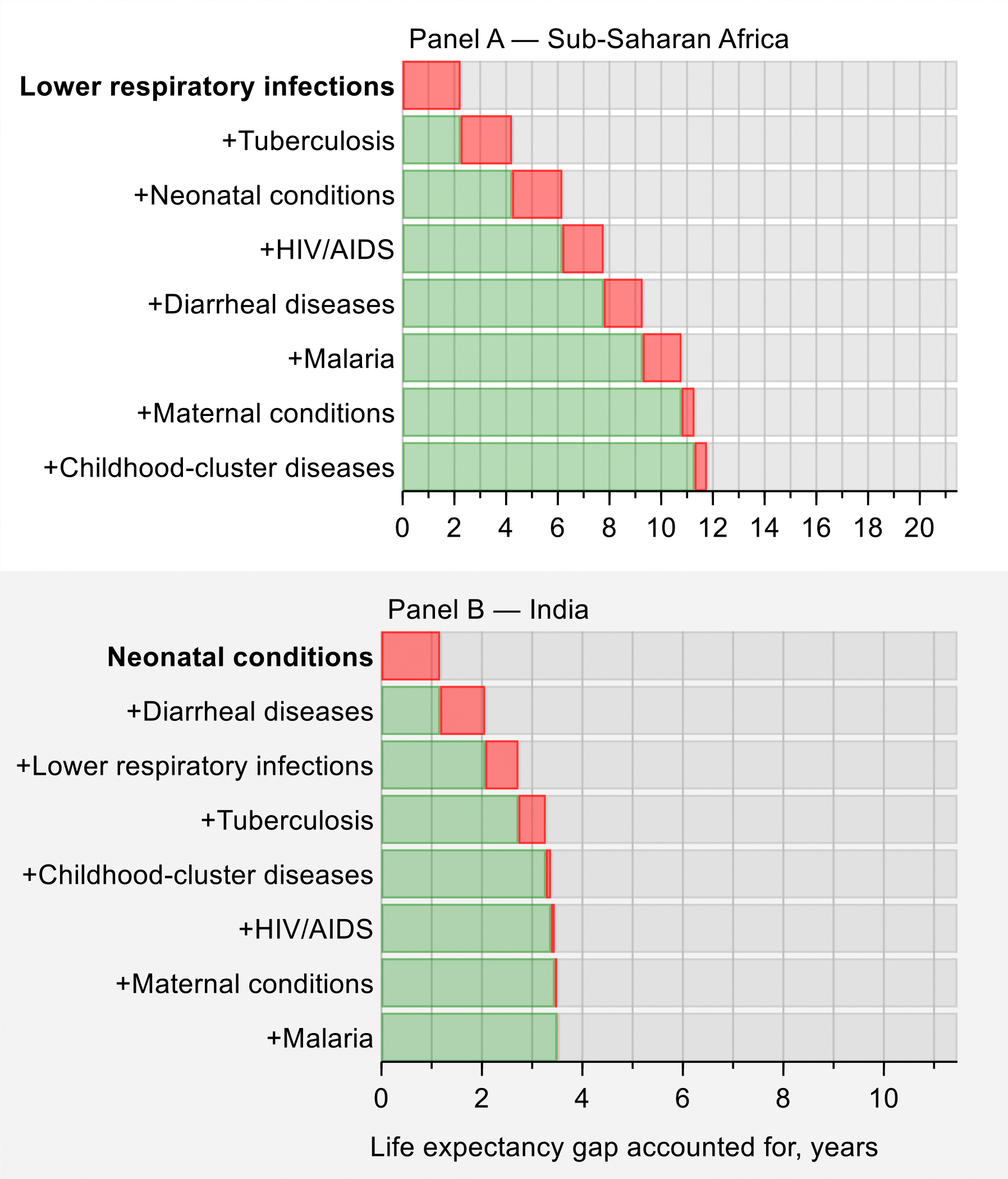
Life expectancy gap compared to the North Atlantic attributable to individual I-8, 2019 Note: Life expectancy in the North Atlantic was 82 years in 2019. The full bars show the total life expectancy gap. Red parts show life expectancy gap accounted for by the cause indicated on the y-axis. Green+red parts show the cumulative contribution of the causes indicated at and above each bar on the y-axis to gap. Gray part shows the proportion not accounted for. Source from reference 80 ^80^ Data from reference 11 and 15 ^11,15^

While acknowledging the significant progress on the I-8 discussed above, these conditions still account for a large share of the life expectancy gap between sub-Saharan Africa, India, Central Asia, and some other regions and the North Atlantic. Figure 11 shows the cumulative contributions of each I-8 condition to the life expectancy gap between the North Atlantic and sub-Saharan Africa and India, respectively. In sub-Saharan Africa, lower respiratory infections, tuberculosis, and neonatal conditions each accounted for about 2 years of the gap, followed by 1.5 years each from HIV/AIDS, diarrheal diseases, and malaria. In India, neonatal conditions, diarrheal diseases, and lower respiratory infections each accounted for about 1 year of the gap. Despite the progress to date, the magnitude of the disparity relative to the North Atlantic remains large.

GH2035 focused on ways to reduce mortality from HIV/AIDS, tuberculosis, malaria, and maternal and child health conditions in all low- and middle-income nations down to the low rates seen in the best performing upper middle-income countries by 2035. Much of under-15 mortality comes from the I-8 conditions of lower respiratory infections, diarrheal diseases, and childhood cluster diseases; some comes from malaria and HIV/AIDS, which we consider separately. We consider I-8 to be a useful aggregate indicator. We now examine the worldwide progress against the I-8 conditions between 2000–2019.

Death rates due to tuberculosis, malaria, maternal mortality, and under-15 mortality have all nearly halved from 2000–2019, while HIV/AIDS death rates have reduced by two-thirds. Three of the eight 2000–2015 millennium development goals focused on child mortality, maternal mortality, and mortality from HIV/AIDS, tuberculosis, and malaria; these goals mobilized attention to and funding for tackling these diseases, including from the Global Fund and Gavi, the Vaccine Alliance. The pace of decline for tuberculosis, HIV/AIDS, and malaria mortality increased between 2010–2019 compared to 2000–2009, while it fell for maternal mortality and under-15 mortality (table 7). From 2010–2019, the HIV/AIDS death rate fell by 7% per year, the tuberculosis death rate fell by 5% per year, and the malaria, maternal, and under-15 death rates all fell by about 3% per year.

Mortality from these conditions is not evenly spread but is concentrated within certain countries. For example, in 2019, 50% of all malaria deaths, 45% of all tuberculosis deaths, and 22% of all HIV/AIDS deaths are concentrated in the top three highest burden countries for each disease. Similarly, 31% of all maternal deaths and 40% of all under 15 deaths are concentrated in the top three highest burden countries (Nigeria, India, and Pakistan).

We calculated the levels and rates of decline in mortality rates for the 30 countries with the highest number of deaths from each condition (appendix tables 4.2–4.5 on pages 20–26 of the appendix). Likewise, we summarize progress against under-15 mortality (appendix table 4.6 on page 28 of the appendix). The world as a whole experienced impressively high rates of decline in mortality from the I-8 conditions between 2000 and 2019, and the decade 2010–2019 was, on average, slightly better than 2000–2010. For under-15 mortality, 13 of the 30 highest burden countries had faster declines in 2010–19 than in 2000–10, while Yemen was the only country that saw an increased under-15 mortality rate in 2010–19. The fastest declines in 2010–19 were in Bangladesh, China, Ethiopia, India, and Uganda (appendix table 4.6 on page 28 of the appendix).

##### The impact of the COVID-19 pandemic

The impact of the COVID-19 pandemic on cause-specific mortality rates is difficult to estimate.^82^ We rely on data from the WHO Global Health Estimates (GHE), which is considered one of the most reliable data sources for up to 2021, but we recognize its limitations. According to the GHE, during the COVID-19 pandemic between 2020 and 2021, slower rates of decline in death rates were seen for HIV/AIDS and under-15 mortality (table 7). Increased death rates were seen for tuberculosis, malaria, and maternal mortality. In contrast to our analysis, which we based on the GHE, a recent Global Burden of Disease (GBD) study reported lower tuberculosis deaths during the pandemic compared to expected deaths.^83^

In our analysis of the top 30 highest burden countries, 23 countries had either slower declines or increased tuberculosis death rates during the pandemic (appendix table 4.2 on page 20 of the appendix). Nevertheless, several sub-Saharan African countries (Democratic Republic of the Congo, Ethiopia, Nigeria, Tanzania, Uganda, and Zambia) recorded an over 6% annual reduction even during the pandemic.

Examining HIV/AIDS death rates, some countries maintained or accelerated their progress, and 11 countries achieved declines of over 10% per year even during the pandemic (appendix table 4.3 on page 22 of the appendix). However, increases in death rates were seen in China, Mozambique, and particularly Pakistan, Kenya, and the Russian Federation where the increase in death rates were 11%, 9%, and 7% per year, respectively.

More than half of the 30 top burden countries for malaria recorded increased malaria deaths. Only three countries (Democratic Republic of the Congo, Liberia, and South Sudan) experienced faster declines in malaria death rates during the pandemic (appendix table 4.4 on page 24 of the appendix).

For maternal mortality, three countries (Bangladesh, Chad, and Kenya) experienced an accelerated decline in maternal mortality, while most countries had increased mortality rates. Countries with the highest increase include Brazil, China, India, Indonesia, Malawi, and the Philippines (appendix table 4.5 on page 26 of the appendix). More than half of the 30 countries experienced a slowing of the decline in the under-15 mortality rate, and Yemen had increased rates during the pandemic (appendix table 4.6 on page 28 of the appendix).

Overall, despite the shock of the pandemic, impressive progress continues in tackling infections. The trends outlined above suggest that a 30% reduction in PPD by 2035, through focused attention on these infections and maternal health conditions, remains feasible. More time is needed to assess the full impact of the pandemic on these trends between 2022 and 2023.

#### Progress in reducing mortality from NCDs and injuries

**Figure 12:**
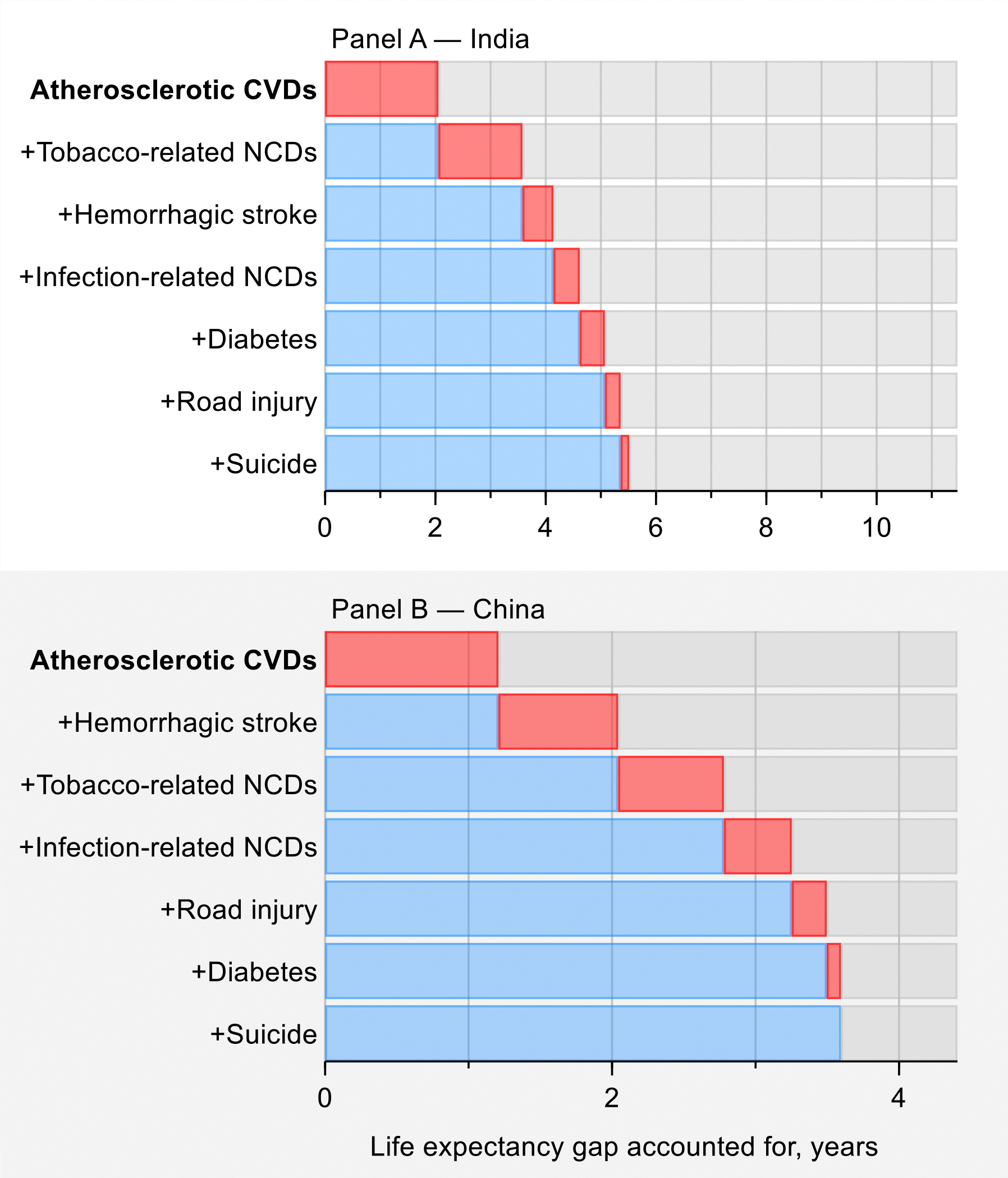
Life expectancy gap compared to the North Atlantic attributable to individual NCD7, 2019 Note: Life expectancy in the North Atlantic was 82 years in 2019. The full bars show the total life expectancy gap. Red parts show life expectancy gap accounted for by the cause indicated on the y-axis. Blue+red parts show the cumulative contribution of the causes indicated at and above each bar on the y-axis to gap. Gray part shows the proportion not accounted for. Source from reference 80 ^80^ Data from references 11 and 35 ^11,15^

Achieving this broader concept of “50 by 50” will require attention to both the I-8, described above, and the NCD-7. Figure 12 illustrates the contribution of the NCD-7 to the life expectancy gap in 2019 between the North Atlantic and India (figure 12 panel A^84^) and between the North Atlantic and China (figure 12 panel B). In India, atherosclerotic CVDs and tobacco-related NCDs each accounted for 2 years of the 5.5-year life expectancy gap attributed to the NCD-7. In China, atherosclerotic CVDs, hemorrhagic stroke, and tobacco-related NCDs each accounted for about 1 year of the 3.6-year gap in life expectancy due to the NCD-7.

Globally, the decrease in NCD-7 death rates accounted for about 1.9 years of the 6.3 year gain in life expectancy between 2000–2019 (Table 6). Improvements in mortality from the NCD-7 did little to raise life expectancy in India and sub-Saharan Africa, both in absolute and relative terms. On the other hand, improvements in the NCD-7 underpinned half of the life expectancy improvements in China and 83% of life expectancy improvements in the North Atlantic from 2000–2019. In the North Atlantic, reduction in atherosclerotic CVD mortality was the largest contributing factor, accounting for 2 years of the 3.8-year increase from 2000–2019. In China, improvements in tobacco-related NCDs, hemorrhagic stroke, and infection-related NCDs accounted for more than half of the 6.1-year gain in life expectancy, while the impact of atherosclerotic CVDs was small, although it increased in importance in 2010–2019 compared to a decade earlier (appendix table 4.7 on page 30 of the appendix).

While progress on mortality from the I-8 from 2000–2019 was relatively favorable, progress on the NCD-7 during this period was mixed (table 8). On the positive side, globally, those two decades saw a general decline in age-specific mortality rates for the NCD-7. For example, for the critical age group 50–69 the mortality rate globally declined by 1.6% per year, with better rates for some conditions and some regions (appendix tables 4.4 and 4.7 on pages 24 and 30 of the appendix).

**Figure 13:**
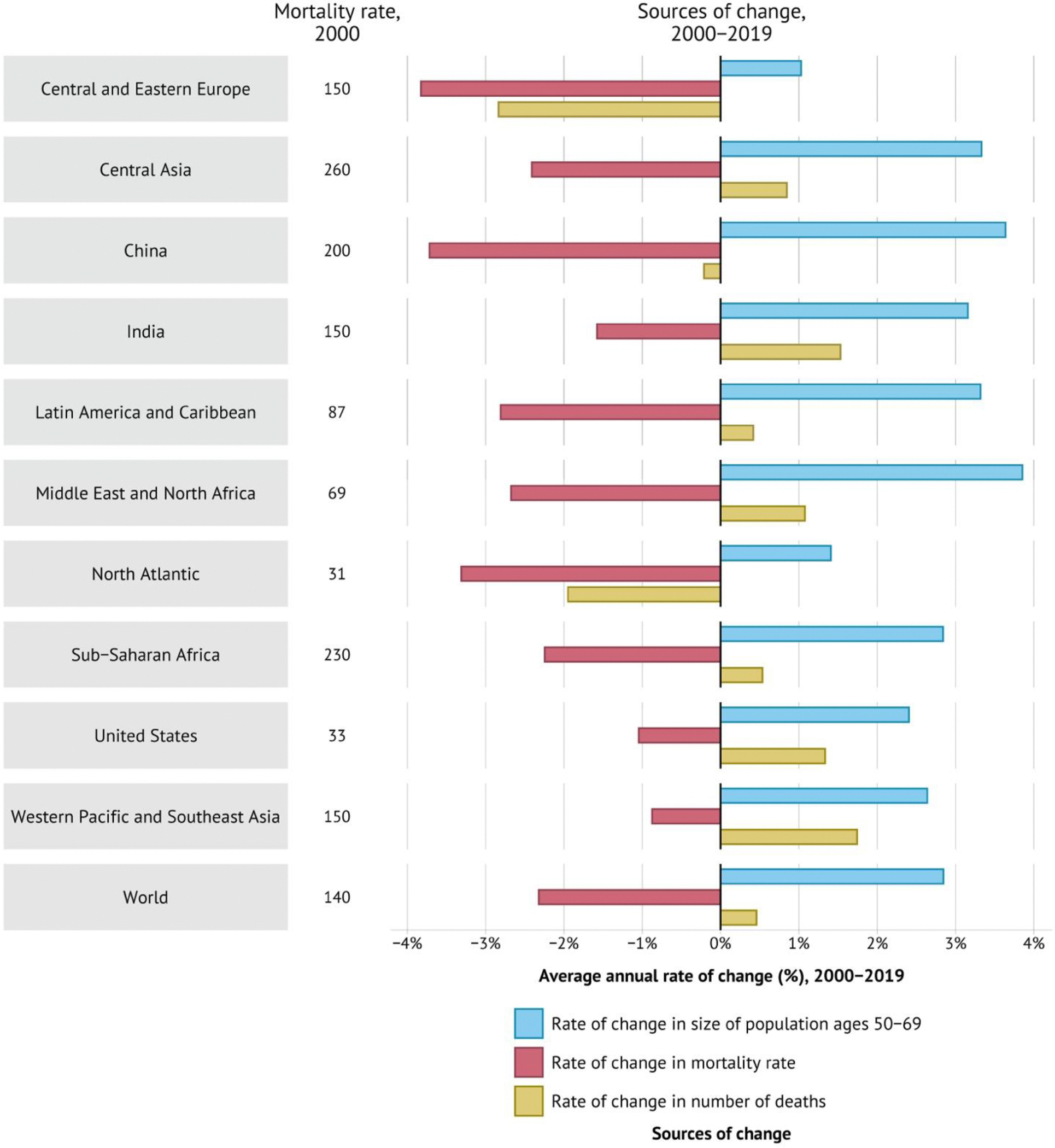
Hemorrhagic stroke, sources of change in deaths from 2000 to 2019, ages 50–69, by CIH region Note: Mortality rate in year 2000 is expressed per 100,000 population per year. Negative rate of change in the figure indicates decline and a positive rate of change indicates increase.

However, improvements in or stabilizing of age-specific death rates represent an incomplete measure of success, for two reasons. First, and most importantly, our analyses show that population growth and aging—which are in part a consequence of past successes in reducing deaths from infections and maternal health conditions—are expected to drive up the number of persons dying from NCD-7s over time by around 1–2% per year^85^ (appendix table 4.8 on page 34 of the appendix). This would represent a close to doubling of NCD-7 deaths by 2050 compared to 2019, accompanied by large growths in incidence and prevalence, leading to a historically unprecedented increase in demand for NCD-7-related healthcare. Figure 13 shows for hemorrhagic stroke, the decomposition of the increase in deaths by region into the part due to demographic change and the part due to reduced mortality rates. High-income countries had most of the 20^th^ century to adjust to epidemiological and demographic shifts and gradually redesign their healthcare systems around prevention and care of NCDs and injuries. Unfortunately, low- and middle-income countries have neither the luxury of time nor the magnitude of investment in the health sector that would be required to replicate the systems that evolved in high-income countries.

Second, data from the WHO’s Global Health Observatory suggest that the risk environment for the NCD-7 has deteriorated over the years. This deterioration has been exacerbated by factors such as greater tobacco affordability in middle-income countries, persistent ambient air pollution, rising harmful alcohol consumption, rapid industrialization fostering a sedentary lifestyle, and the proliferation of unhealthy diets worldwide. As discussed in section 8, obesity rates are rising in many parts of the world, including in children. Without a clear set of policy priorities and the accompanying political courage to implement them (often over the objections of commercial and professional interests), it is possible that rising age-specific incidence of the NCD-7 will place the burden of mortality reduction more firmly onto health systems.

The challenge for control of the NCD-7 then, is twofold: (i) to maintain a focused approach that emphasizes intersectoral action against tobacco (discussed in section 8) and the most cost-effective medical interventions (e.g., CVD prevention), and (ii) to use available resources to innovate on healthcare delivery models that are leaner (i.e., more cost-efficient) and that ideally have similar or better quality of care as has been achieved in high-income countries. Here, the signs are promising. With each passing year, there are more reports from communities of health researchers in the global south demonstrating the effectiveness of technology-supported, locally informed innovations in delivering care for NCDs and injuries.^86–88^ What is needed is greater international financial support for knowledge sharing and cross-country learning.

#### Conditions with high morbidity

The focus of our analyses so far has been on mortality indicators, in part because the evidence base on the prevalence of morbidity is weaker due to large challenges in collecting data and defining morbidity. However, people also care about living healthy lives and so reducing morbidity and improving health-related quality of life are important goals. Health indicators that reflect both mortality and morbidity, such as health-adjusted life expectancy and disease-adjusted life expectancy, correlate highly with life expectancy (figure 3). Most interventions that reduce mortality rates result, therefore, in more healthy lives. In populations with high life expectancy, the proportion of time lived with reduced quality of life tends to increase.^89^

Nevertheless, despite the strong correlation between life expectancy and health-adjusted life expectancy, there are several conditions that cause significant suffering and health burden but do not result in high mortality. We highlight three in particular.

First, mental illnesses, such as affective disorders and schizophrenia, are leading causes of morbidity globally, and cause significant economic loss from presenteeism and absenteeism. Furthermore, national prevalence data for these conditions are not presently produced by the WHO. According to the GBD, the age-standardized rates for mental illnesses have remained relatively stable from 1990 to 2019, which may reflect the challenges in collecting prevalence and severity data over time.^90^ Ambiguity and imprecision in diagnostic criteria undermine epidemiology and other aspects of psychiatric science.^91^ While genome wide assessments of disease or risk offer promise for the future, argues Steven Hyman, their practical development remains immature. The COVID-19 pandemic has exacerbated some mental illnesses, a trend that is likely to intensify with the continuing global climate crisis.^92^ The cost of mental illnesses makes a major contribution in Cutler and Summers’ assessment of the economic consequences of COVID-19 in the US.^93^ Caring for individuals with mental illnesses imposes a large psychological, physical, and financial burden on caregivers.^94,95^ Although lists of deaths by cause show little impact from mental illnesses, some of them (bipolar illness, schizophrenia) are strong risk factors for all-cause mortality, particularly mortality from cardiovascular disease and suicide.^96^ Successful interventions will thus reduce mortality rates as well as morbidity.

The second condition is dementia, which represents a significant public health challenge, particularly in countries with rapidly aging populations. Dementia adversely affects cognitive function and reduces the quality of life for both individuals and their families and is an important cause of death in old age. Dementia was the seventh leading cause of death globally in 2019.^15^ Beyond mortality, the global age-standardized dementia prevalence remained stable between 1990–2019 and is expected to remain stable until 2050.^97^ However, similar to mental illnesses, there are challenges in collecting rigorous prevalence data and so there is wide uncertainty around the prevalence and morbidity estimates from the GBD.^98^ While some high-income countries have reported declines in age-specific incidence rates, the absolute number of individuals affected will continue to rise due to demographic change.^97,99^ Beyond its direct health impact, dementia incurs substantial long-term care needs. Jin et al^100^ projected the need for long-term dementia care in China forward to 2050 and found that meeting this need could cost as much as 6% of gross domestic product (GDP). And, similar to mental illnesses, the psychological as well as time burden on caregivers is high, often disproportionately affecting female family members, exacerbating gender inequalities in health and economic well-being.^101^

Third, in most countries where infections and maternal health conditions dominate, there is widespread failure of child development and a corresponding need to shift from an exclusive focus on child survival to child thriving. Height-for-age and mathematics test scores serve as significant measures of child development and are relatively well measured. Panel 6 highlights newly available data on adolescent height and mathematics skills that show great disparities between even middle-income countries and well-performing ones. Every child deserves not only to survive, but an opportunity to achieve their full potential; exclusively focusing on mortality prevention misses this goal. Poor physical and cognitive growth through adolescence confers significant health and economic disadvantages on children, especially from poor households, over their lifetime.^102^ Interventions exist, that, at least in part, can address these problems. In section 5, we describe a modular approach to health systems strengthening (HSS) that includes interventions to promote children’s health.

### Panel 6: The importance of the “next 7,000 days”

We have demonstrated that the 15 priority conditions account for most of the mortality differentials across regions. These 15 conditions are the most immediate causes of death but have many causal components and associated risk factors: Most premature deaths—from infections as well as NCDs—are determined by exposures occurring repeatedly or over an extended period involving many interlinked factors starting as early as the fetal stage.^103^ Therefore, a life course perspective is needed to fully understand and prevent risk factors leading to premature death. The overwhelming focus of the life-course literature has been the “first 1,000 days” from conception; linking maternal, neonatal, and infant health to mortality, infections, and chronic diseases at subsequent life stages, including adulthood and old age.^104,105^ Therefore, preventing adverse exposures in early life—particularly diarrhea, respiratory infections, and neonatal conditions—will have compounding effects on premature mortality throughout the life course.

However, little attention is paid to human development between the first 1,000 days and adulthood—“the next 7,000 days.” Using the number of global publications to measure research interest, 70% to 90% of research attention on the first 20 years of life has been expended before the subjects reach 5 years of age.^106,107^ Yet the “next 7,000 days,” from about 5 to 19 years, is increasingly recognized as a crucial period for human development. It is a period of dramatic growth and change, including the physical and emotional changes associated with puberty, the adolescent growth spurt, and the rewiring of the late adolescent brain that establishes lifelong mental health and behavioural trajectories.^108^ For example, adolescence is when most smoking initiation occurs.^109^

Education and other human capital acquisition are heavily impacted by events and decisions during adolescence, ^110^ which in turn affects future health and mortality. Estimates suggest that 12 years of schooling reduces the risk of mortality in adulthood by 25%, or almost two percent for each additional year of education.^111^ Suggested pathways are varied, including better income and access to quality care and improved health-related knowledge and healthy behaviours.^112^

A still weak, but rapidly improving evidence base points to enormous inequalities across countries in key aspects of development during adolescence.

#### Global state of the physical and cognitive health of 5–19-year-olds

Global access to education has never been higher. Attendance among children and adolescents of primary and secondary school age (about 6–17 years) is now estimated to be 84% worldwide, and continuing to rise.^113^ The outcomes of education provision are determined in part by the well-being of the learner, yet, while the world carefully monitors education quality and coverage through the UNESCO Institute for Statistics, we do not currently have global systems that monitor the well-being of the learners (6–17 years).

**Figure 14:**
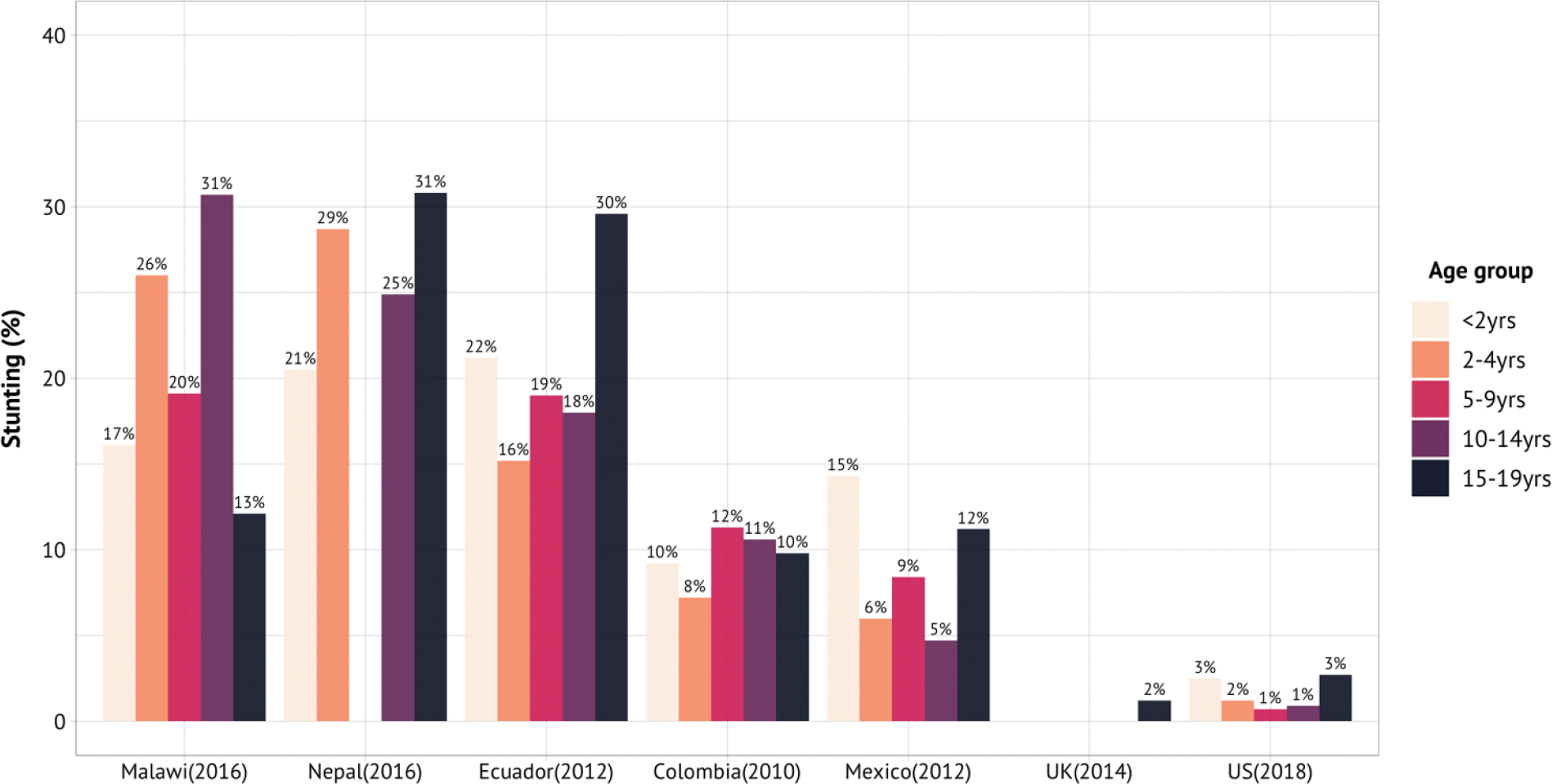
Prevalence of stunting among females in selected countries

Here we use two special data sets to explore two key aspects of such wellbeing. For physical growth, we use data compiled and harmonized by the Biomarkers Reflecting Inflammation and Nutritional Determinants of Anemia (BRINDA) Project to form one of the most comprehensive datasets of school-age child and adolescent nutrition biomarkers. This new and unique data set shows that the prevalence of stunting, which correlates with educational underachievement, is in the range of 10% to 30% in children in low- and middle-income countries (figure 14 for females; for males, see appendix figure 4.1 on page 17 of the appendix). Stunting persists not only during the first 1,000 days, but also throughout the next 5–19 years when we invest most in children’s education. The results suggest that in some countries nearly a third of 17-year-old girls remain at the height of a normally growing 12-year-old girl, while the survey results from high-income countries show that stunting can be largely avoidable at any age. Further, evidence suggests that although early stunting leads to a low growth trajectory if the social determinants remain unchanged, ^114^ the stunting is largely reversible through appropriate interventions.^115^

**Figure 15:**
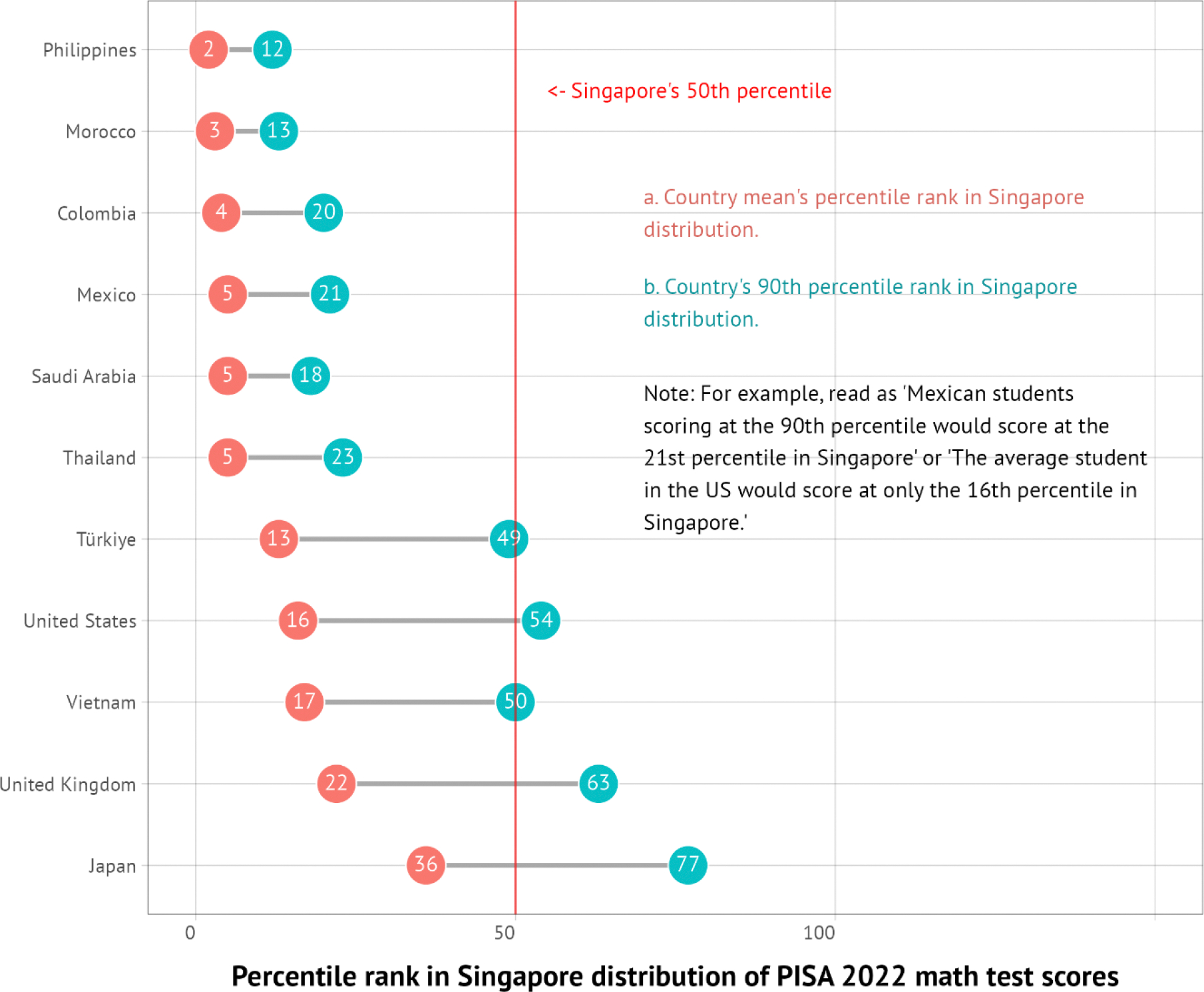
Mathematics test scores relative to Singapore, PISA 2022, selected countries Source from reference 84 ^84^

For cognitive development, we used data from the standardized mathematics test conducted by the OECD as part of their Programme for International Student Assessment.^116^ Comparing the 2022 test results with the achievable results in a “frontier” country, in this case Singapore, figure 15 shows huge cross-country gaps in math test performance, with very substantial and significant under-performance even in middle-income countries. The level of achievement in low-income countries would be expected to be even further away from what is possible, but adolescents are not currently included in any global comparative assessment of education achievement. The huge scale of this difference, comparable in scale to that seen with physical growth, argues for a link between under-achievement in physical growth and cognitive development.

The world invests some USD 2.8 trillion globally (1 trillion in low- and middle-income countries) in the education of young people 5–19 years.^117^ The Disease Control Priorities, Third Edition (DCP3) estimates that in in low- and middle-income countries the investment in the health and wellbeing of this age group is just 2% of the education investment, and only 13% of the health investment in children under 5 years.^108^

#### Key interventions in the “next 7,000” days

There is an established literature on school health and school meals, which has used analysis of cost-effectiveness to identify a school health and nutrition package.^115,118,119^ The essential package for school-age children in DCP3 includes interventions that can be delivered in a school setting to improve cognition and learning, including: the provision of deworming tablets in endemic settings; vision screening and provision of spectacles; promotion of oral health and insecticide-treated bed nets in malaria-endemic areas; and the delivery of dietary education and healthy school meals. In addition, the package protects against vaccine-preventable conditions in adulthood and among future offspring by including tetanus toxoid and HPV vaccinations.^119^ Looking forward, there is growing recognition of the long-term value of early surgical intervention during school age and adolescence^120^ and of the potential damage to mental health of inappropriate social media. The interventions and their benefits are summarized in appendix table 4.9 on page 67 of the appendix.

There is growing global support for school-based interventions, in particular health services and school meals. School-based health services, such as deworming and vision screening, can improve school children’s health and well-being, supporting them to lead a well-educated and healthy life. Increasing coverage of school meals and school health services is now a global movement supported by the School Meals Coalition, with an emphasis on increasing coverage where it is needed the most. In 2022, estimates indicate that 420 million children were reached by school health and nutrition programmes, and that between 80 and 140 million of the most vulnerable children in low- and middle-income countries remained unreached. Expansion of national school health and nutrition programmes to reach those most in need should be a global goal.^121^

## Section 5. A modular approach to health system strengthening

### Bringing focus and specificity to the health systems agenda

The most recent UHC Monitoring Report found that, in the aggregate, the world has made almost no progress on health service coverage since the start of the SDG era, except for continued progress on HIV treatment ^122^ Further, the incidence of catastrophic health expenditure continues to increase rather than decrease. Taken together, these data suggest that the UHC agenda has not been driving progress on health outcomes as much as was expected.

The discourse around UHC suggests that an overly broad vision of UHC, and the general lack of realism on what UHC entails in terms of collective action and fiscal choices, may be contributing to slow progress.^3^ Related, the discourse around “health systems strengthening” has focused largely on how to improve the levels of various health system inputs in resource-poor countries—rather than on how to use limited resources to directly improve population health and build resilient health systems.^123^ The Commission calls for a reset on the UHC and health systems strengthening agendas. We recommend that national governments maintain their focus on public finance of a core set of interventions that are fully prepaid and available to everyone, starting with the poorest (progressive universalism), regardless of location or financing scheme, and with accompanying social protection programmes. This section presents a modular approach to health systems strengthening that would enable building out from an initial focus on “50 by 50” to allow movement towards more comprehensive UHC over time.

What sorts of interventions would help to achieve the “50 by 50” goal and improve the quality of life at all ages? We reviewed recommendations from the WHO and the Disease Control Priorities Project on cost-effective interventions for major health conditions.^124,125^ We sought to identify “core” interventions that were likely to be cost-effective and feasible to implement in countries at all levels of income. In addition to interventions that address the 15 priority conditions, we identified interventions that address other major demands on health systems, e.g., rehabilitation, child and adolescent development, and palliative care. The inclusion of these latter interventions alongside mortality-focused interventions is critical to our proposed “50 by 50” goal, which includes improved quality of life at all ages. These latter interventions are frequently neglected, including by development partners, despite being highly valued among citizens.

Table 9 presents our findings, and a background paper provides more details.^126^ The table is organised by delivery platform and by health topic, with related interventions grouped together. We call these groups “modules.” Each module represents a programme area with a specific set of policies and financing arrangements. We are not advocating for these modules to be “vertical programmes” in the usual sense of the term. We are emphasising that governments can still devote much of their effort and resources into ensuring the effective implementation of specific health conditions and interventions, even within integrated financing and delivery systems. In the next section we point to a specific approach to public finance–improving the availability and affordability of drugs and other key inputs to each module–that would facilitate implementation within an integrated delivery system.

Because table 9 is focused on the foundations of the healthcare system (e.g., treatment of HIV, prevention of cardiovascular disease, family planning), the interventions in the table can be thought of as a checklist for health system development. However, local circumstances will dictate the details, and not every module or intervention will be relevant in every country. We stress that the interventions in our table are not a prescription for each country, but rather a starting point for local deliberation. That said, we expect that a substantial subset of the modules will be relevant and important in most countries.

We contend that focused investments to expand access to service delivery arrangements that deliver these interventions could greatly accelerate progress towards the “50 by 50” target. Previous studies have assessed the mortality impact of interventions like these in diverse country settings.^125,127^ Hence, there is every reason to believe that substantial levels of implementation of country-appropriate modules would enable success with “50 by 50.” Additionally, most of the interventions in table 9 are not being fully implemented, even in wealthier countries. For example, only 31% of Norwegian adults eligible for colorectal cancer screening from 2013–2016 underwent a screening test,^128^ and only 16% of Chinese adults with hypertension had controlled blood pressure in 2019.^129^ High-income countries like the US could benefit from a careful review of our intervention recommendations to identify opportunities for improved implementation.

### A “modular” approach to priority setting

Most countries have some sort of official and broadly defined health benefits package (HBP) or packages that specify the interventions that are guaranteed to be available to all beneficiaries and available at little-to-no out-of-pocket (OOP) cost. However, in many countries, HBPs are often poorly implemented. A recent review of experiences from several low- and middle-income countries found that HBPs largely serve as advocacy documents.^130^ The costs of implementing HBPs are often much higher than the available resources, and HBPs are often not linked to financing or service delivery arrangements, hindering their usefulness.^131^ This is unfortunate, because HBP could be a key policy mechanism for allocating scarce resources efficiently and equitably.

The Commission proposes an approach to doing cost-effectiveness analysis for HBPs that adapts to local policy processes, health system configurations, and financing arrangements, thereby making the HBP more implementable. We call this approach “modular cost-effectiveness analysis” (mCEA), and it underpins the approach we propose for health systems strengthening. An mCEA exercise would involve two stages of work. In the first stage, planners would identify a set of mutually exclusive and collectively exhaustive modules that correspond to different health sector programmes and activities. Depending on the country’s epidemiology, health service architecture, and window of opportunity for policy change, these modules might be organized around the focus areas of technical working groups (e.g., malaria, cardiovascular disease), delivery platforms (e.g., outreach clinics, primary clinical care), payment mechanisms, or other organising principles. The choice of modules would inevitably vary country to country or within a country over time, depending on the policy context. Xishui County in China, for example, is initiating a planning process based on the CIH modular approach but focused almost exclusively on the NCD-7 (panel 7).

#### Panel 7. Using a modular approach to identify key NCD-7 interventions to scale up in Xishui County in China

Xishui county in China has launched a Health and Social Development Project based on the modular approach described in GH2050. The project begins by adopting an indicator—the loss of expected life years due to disease—to accurately pinpoint the major health challenges faced by the region and to prioritize health interventions. The predominant health concerns in China are the NCD-7. These conditions contribute significantly to the life expectancy gap, which is 4.6 years for men and 4 years for women compared to the average in the North Atlantic region. Major contributors to this gap, with the magnitude of their contribution given in parenthesis, are atherosclerotic CVDs (1.4 years for men and 1.7 years for women); haemorrhagic stroke (1.0 years for men and 0.8 years for women); NCDs strongly linked to tobacco (0.9 years for men and 0.7 years for women); and NCDs strongly linked to infection (0.7 years for men and 0.4 years for women).

China’s high cardiovascular mortality can be accounted for by a handful of risk factors including high blood pressure, air pollution, poor dietary habits, and tobacco use; high blood pressure alone accounts for 56% of cardiovascular deaths. Among adults with hypertension, the awareness rate (41%), treatment rate (35%), and control rates (11%) are significantly lower than global averages (54% diagnosed, 42% treated, and 21% controlled), indicating a critical need for enhanced primary and secondary cardiovascular care. Additionally, NCDs strongly linked to tobacco account for 24% of all NCD deaths in China, significantly higher than the global percentage of 15% and twice the global average risk-attributable mortality rate. Finally, China has a high burden of NCDs strongly linked to infection, including from complications of hepatitis B (twice the global average mortality rate).

Applying the Commission’s approach, local teams designed three modules to tackle the NCD-7 in Xishui county. The cardiovascular disease module includes combination drug therapy for people at high CVD risk; glycemic control and monitoring for microvascular complications in people with diabetes; long-term management of chronic kidney disease and heart failure; and secondary prevention of atherosclerotic CVD and rheumatic heart disease in endemic settings. The module for NCDs strongly linked to infection includes hepatitis B vaccination to prevent liver disease and liver cancer.

Overall, the aim of this exercise is to ensure that the intervention modules being proposed for Xishui county are scientifically grounded, culturally acknowledged, publicly accepted, and politically feasible, enhancing their sustainability and effectiveness.

Part of the first stage of mCEA would be to estimate current spending on each module and the budget space for expanding or reducing each module based on the available resources. The allocation of budgets across modules would be based on the national health strategy and other policy and political considerations. Included in table 9 are estimates of the incremental cost (as a share of GDP) of expanding the coverage of our recommended “core” interventions for 19 stylised modules (aligned to the “50 by 50” agenda) to an additional 10% of the population, a realistic increment of expansion within a given policy cycle.^126^ For example, the table shows that the cost of a 10% expansion of the coverage of the tuberculosis module would be 0.87 basis points of GDP, where a basis point is one percent of one percent. The goal of providing policymakers with the distribution of costs across modules is to help structure conversations about where to put (limited) incremental resources to support health system development objectives over time. Our cost estimates inform alternative pathways to expansion of different services and priority conditions over time based on local considerations.

Once planners and politicians have set the general direction for the HBP reform and the budget space for each module, the second stage of mCEA would be a technocratic exercise to optimise value for money within each of the modules given the available budget. Experts assigned to each module would start by mapping candidate interventions to their module and defining one or more relevant outcomes against which to compare costs. For example, a malaria module might focus on the cost of different intervention mixes per child death averted, whereas a cardiovascular disease module might focus on the cost per premature adult death averted. Some modules, such as family planning or palliative care, might not even focus on health outcomes. Importantly, the first and second stages of mCEA would not be one-directional; technical analyses might identify opportunities for greater impact within specific modules that could also influence negotiations around budgetary allocations across modules.

The analytical emphasis for mCEA would be to systematically identify synergies or inefficiencies (in terms of costs or outcomes) that might emerge when multiple related interventions are implemented together. The rank-ordering of interventions by value for money within modules would account for these interdependencies. For example, treatment of diabetes on its own might not be cost-effective, but when delivered alongside primary prevention drugs for cardiovascular disease by the same provider to the same at-risk individual, the bundle of interventions could be cost-effective.^132^ The ranks of different interventions could also be adjusted according to other criteria besides cost-effectiveness, such as equity impact or financial risk protection afforded.^133^

### Implications for health systems strengthening

The modular approach that we propose could advance the health systems strengthening discourse in four important ways. First, it could help shift the focus from health system inputs and functions towards the outputs and outcomes that matter to populations. A policy process organised around a local adaptation of table 9 would foreground the key outcomes for the health system to track and the actions required to achieve those outcomes at a reasonable cost. Equity could be enhanced by prioritising interventions and modules (and related service delivery arrangements) that address the needs of the worst off. Actions to promote and measure health system quality could be readily aligned and embedded within a modular approach.^134^ The range of health needs and outcomes covered in table 5.1 could help health systems better respond to emerging challenges and shocks (i.e., resilience). For example, investments in the “emergency care functions” module–including in critical components like oxygen–could save lives during a pandemic surge.^135^

Second, the health systems strengthening literature is underdeveloped with regard to supply chain strengthening for key commodities like drugs and diagnostics.^123^ A modular approach that maintains a focus on a limited set of interventions could inform drug formularies and procurement system reforms. Section 6 proposes a mechanism that is based on economic principles and could greatly improve access and affordability for high-priority medicines.

Third, a modular approach could guide national and international conversations around the health workforce. Health workforce development plans, including pre-service and in-service training curricula, could be aligned with priority interventions. Our approach could help plan out expansions in the primary healthcare workforce and, as a complement, quantify the need for specialised health workers, e.g., for dental care. Still, health workforce gaps can be attributed in large part to inadequate and inequitable pay, poor working conditions, and low retention and high migration.^136^ To deliver on “50 by 50,” many national governments will need policies and resources in place to ensure fair compensation and regulations that protect both health workers and patients and restore trust in the public system.

Fourth, implementing the modular approach would bring attention to another under-developed aspect of health systems strengthening: health information systems.^123^ By providing a roadmap for health system development, the modular approach would also inform the sorts of key indicators that need to be routinely collected and digitised, including expenditure data, service utilisation data, and clinical outcomes data. An emerging opportunity is to leverage real-world data, such as from the District Health Information System 2 platform, to improve monitoring and implementation of priority interventions.^137^ We do note, however, that many countries–including in sub-Saharan Africa–still lack high-quality demographic and cause-of-death data,^138^ and this hinders any serious attempt to set national priorities using local data. Rectifying this problem is an urgent priority for development assistance for health. Most of the interventions featured in table 9 could be delivered through primary healthcare systems, broadly defined to include community, outpatient, and first-level inpatient care.^3^ Countries that have excelled on premature mortality and indicators for UHC have done so using primary healthcare systems. For example, Thailand’s UHC reforms focused heavily on primary healthcare, and the country has achieved a life expectancy similar to the North Atlantic region but at a fifth of the purchasing power parity-adjusted cost. Successful primary healthcare initiatives tend to have several elements in common, including: (i) empanelment, i.e., assignment of patients to clinics based on geographic proximity, (ii) provision of a manageable set of preventive, chronic, and acute services across the lifespan (as in table 9) at little or no OOP cost, and (iii) use of community outreach workers who are in regular contact with local households to assess priority health needs and connect individuals to services.^16,139^

## Section 6. Health system financing in turbulent times

### Cost implications of the modular approach and “50 by 50”

Achieving a 50% reduction in premature death by 2050 will require countries to devote sufficient resources to the health sector. We view “50 by 50” as a domestic health agenda and universal public finance as its principal financing mechanism. To this end, we estimated the cost required to support full population coverage and prepayment of our recommended interventions for the 15 priority conditions (table 9) in 63 low- and lower-middle-income countries that represent 87% of the total population in these two income groups.^126^ By 2050, the average low-income or lower-middle-income country would need to be spending 2.5% or 4.1% (respectively) of current GDP on these interventions. This is consistent with previous studies that estimated that lower-resource countries would need to spend about 5% of GDP on healthcare to make sufficient progress towards UHC.^140^

The average increase in health spending that would be needed to scale up these interventions to full coverage by 2050 would be an additional 1.1% of current GDP in low-income countries and an additional 2.0% of current GDP in lower-middle-income countries. While this level of incremental spending corresponds to the commitments made by many countries at the 2019 UN HIgh-Level Meeting on UHC to spend an additional 1% of GDP or more for health services,^141^ it implies that government health expenditure will need to at least double, and that nearly all of the additional spending will need to be directed towards the priority conditions and interventions. Of course, some of the poorest countries will not be able to mobilise sufficient domestic resources to double their health spending by 2050 (see below), and continued external assistance will be required. This implies a need to shift the portion of development assistance that goes to direct country support towards these poorest countries to ensure they are not left behind.

The cost estimates for the “50 by 50” interventions are higher than those featured in GH2035, ^2^ mostly because the range of interventions is broader than the I-8 and now includes focused efforts on NCD-7 conditions. However, the estimates are lower than those featured in our 2018 CIH 2.0 report,^3^ which looked at a comprehensive package of services for UHC systems that includes and goes beyond the conditions and interventions featured here. This report’s estimates can be viewed as a minimum required level of spending on health services to address the 15 priority conditions and a highly focused response to emerging threats. However, it assumes that spending will be on existing services and commodities. Healthcare delivery innovations and R&D to develop cheaper drugs and diagnostics could reduce the required costs. Conversely, with continued GDP growth the fraction of GDP spent on these interventions will decrease, unless costs commensurately rise.

Countries that choose to adopt a “50 by 50” target and adapt our general intervention recommendations face three interrelated challenges. First, countries will need to ramp up domestic government health expenditure despite significant macro-fiscal headwinds. Second, progress on “50 by 50”—especially on I-8 conditions and reproductive health—will inevitably accelerate the demographic transition in lower-resource countries, posing longer-term threats to financial sustainability of health spending. Third, many countries could finance the “50 by 50” target at least in part by shifting government funding towards the priority interventions and away from lower-value interventions, but this could be challenging politically. These issues are explored further in the remainder of this section of the report.

### Domestic resource mobilisation in a time of economic headwinds

Health financing trends have undergone some important shifts since 2000 (table 10). The period 2000–2009 is often referred to as the “golden age” of global health spending. Fuelled by economic growth, domestic government health expenditure increased considerably, and domestic spending was complemented by a rapid increase in development assistance for health, largely to support the Millennium Development Goals. Countries like China and Thailand that were early adopters of UHC reforms saw significant reductions in out-of-pocket spending (as a share of total health expenditure). However, economic slowdown following the 2008 global financial crisis led to a deceleration in the growth rate in domestic government health expenditure. Political shifts and austerity measures in many wealthier countries also led to stagnation in development assistance for health. Additionally, progress on reducing OOP spending slowed, albeit with some notable exceptions like China (appendix figure 6.1 on page 69 of the appendix).

During the COVID-19 pandemic most countries increased domestic spending on health, and there was a surge in development assistance, but emerging data suggest this was a deviation from a longer-term trend to which many countries have since reverted.^48^ The biggest challenge facing the health financing agenda is tepid economic growth and the scarring of many economies in the aftermath of the pandemic. The most recent Global Economic Prospects projects that global economic growth will be a bit slower than the 2010–2019 average, including about 4% in emerging economies.^143^ Although inflation has slowed since the pandemic, it remains higher than desired, and rising debt servicing costs in many low- and middle-income countries are hindering their ability to ramp up public spending. In the absence of strong advocacy efforts and clear “asks” to finance ministries, these headwinds will make it more challenging for health services to get the public resources they need to deliver on rapid mortality reductions.

**Figure 16:**
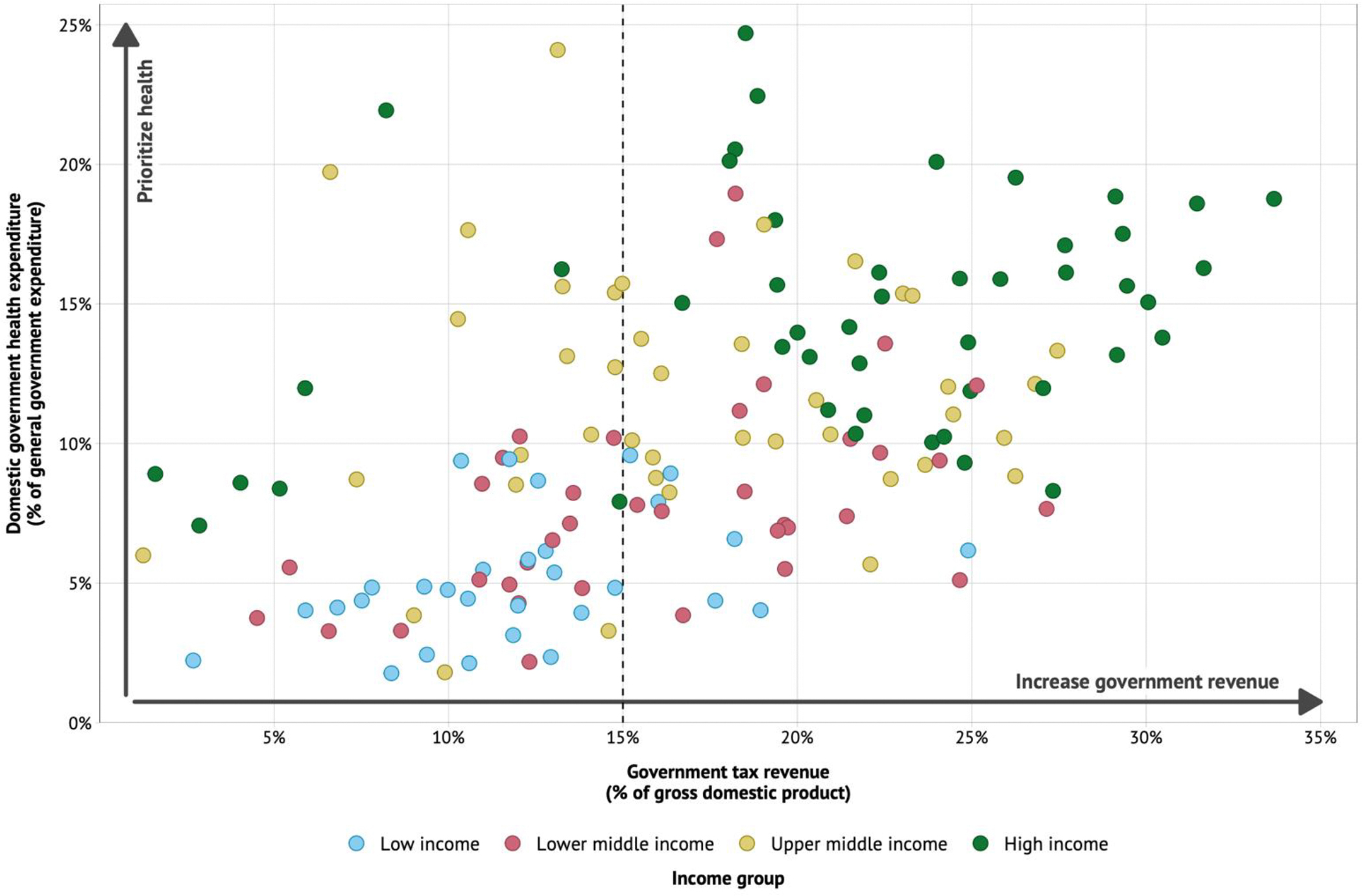
Current levels of government tax revenue and health spending, by country Note: Data are from the reference 145.^145^ The most recent values for each country with complete data (n=126) are plotted here.

Countering these economic headwinds will require action on the part of governments, both executive and legislative branches. As shown in figure 16, the first step in many countries would be to increase general government revenue through increased taxation and improved efficiency of tax collection. This is because, all else equal, more government revenue usually leads to more money for health services. The International Monetary Fund recently estimated that low- and middle-income countries could undertake a series of policy and institutional reforms that could increase their tax-to-GDP share by up to nine percentage points, with a medium-term minimum tax-to-GDP target of 15% of GDP.^144^ Of course, higher tax-to-GDP shares would be required over the longer term to finance an expanding set of goals around the SDGs and the climate transition. Many countries could increase the share of general government expenditure allocated to health. Although we advise against normative targets for the health share of government spending, our cost analyses and country experiences imply that most low- and lower-middle-income countries will need to devote at least 10–15% of general government expenditure to health, even if they have a tax-to-GDP share of 15%. Bids for increased public finance for health budgets will ideally be linked to clear policies and reforms to steward those additional resources well, including by focusing the additional resources on highly cost-effective interventions to reach the unreached with the priority 15 conditions. Of course, it is important to continue pointing out (as we did in GH2035) that healthier populations contribute to greater economic growth.

Given constraints on public sector finance and growing private incomes, many countries will experience rapid growth in private expenditures – both OOP and private voluntary insurance. Such growth has been, historically, highly inefficient, and GH2035 pointed to evidence that it may be more likely to increase rather than relieve pressure on public finance. An alternative—mandatory taxation of users of the services outside the basic package—has shortcomings as well, but can be a viable option.

Many countries also have an opportunity to better steward their existing public sector health resources. We recommend three actions that could improve the efficiency of current spending. First and foremost, some countries could considerably improve public financial management systems. A recent study found that, on average, health ministries in low-income countries return US$ 4 per capita annually that has not been spent; this amount is nearly equivalent to the entire budget for primary healthcare in some countries.^146^ Greater international investment is needed to strengthen and modernise public financial management systems in the poorest countries. Second, procurement of drugs and other commodities is often highly inefficient and duplicative, especially in countries that are heavily aid-dependent and where donors have incentives to set up siloed procurement systems.^147^ Coordination and consolidation of procurement efforts, potentially as part of the broader “one plan, one report, and one budget” agenda ^148^, could free up resources and improve access to a range of commodities. Third, countries could strengthen their priority-setting processes and establish institutions that would guide spending towards interventions that provide more health for a given level of spending.^131^ This report presents two measures that could facilitate spending on priority interventions and programmes: modular cost effectiveness (mCEA, discussed in section 5) and the “Arrow mechanism” (discussed later in this section).

### Domestic resource mobilisation in an aging world

**Figure 17:**
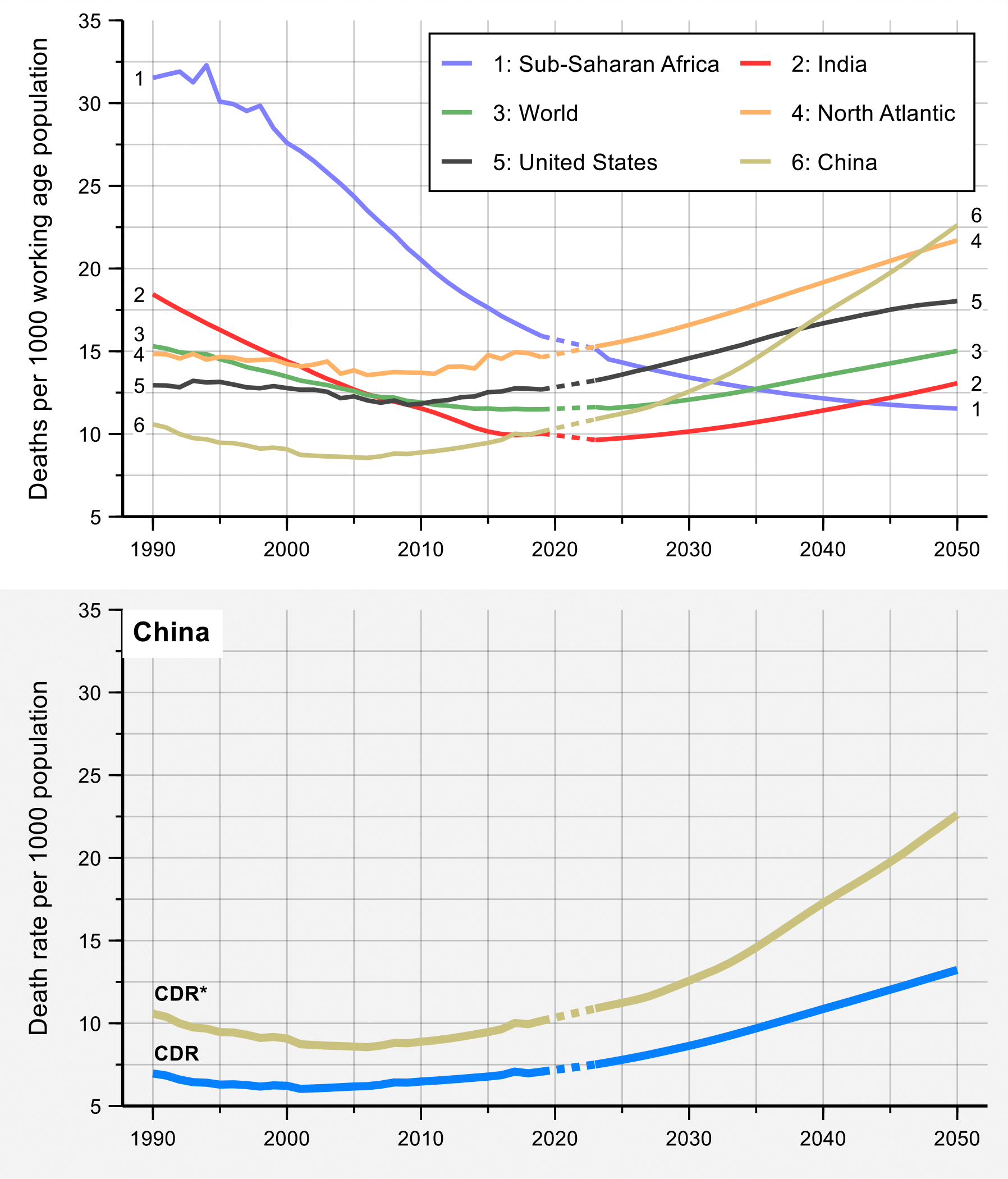
Crude death rate, 1990–2021 and projections to 2050 Note: COVID-19 years (2020–2022) were omitted. Working age is defined as ages 15–64 years. Data Source from reference 11 ^11^

Changes in fertility and mortality rates have dramatically reshaped the demographic makeup of most countries. For the first time in recent history, the crude death rate (CDR) is on the rise in nearly all regions, especially China, the Middle East and North Africa, and the Western Pacific and Southeast Asia (figure 17). If UN Population Division projections for 2050 hold true, the world will experience 1.6 times more deaths in 2050 than in 2019, implying a surge in demand for healthcare driven by a growing older-age population (table 11). While population aging undeniably increases demand on healthcare systems, the broader context is, of course, a potentially highly positive one.^149^

Meanwhile, there will only be moderate growth in the working age populations that are the major contributors to the tax base and a critical source of government revenues for health. In some regions, including China, Central & Eastern Europe, and the North Atlantic, working age populations will decline over the coming decades (table 12). Globally, the old-age dependency ratio is projected to increase from 14% in 2019 to 20% in 2035 and to 26% by 2050. China and the North Atlantic are projected to have old age dependency ratios over 50%, followed by 45% and 39% in Central and Eastern Europe and the United States, respectively. Compared to the UN Population Division projections, full implementation of the recommended interventions to achieve “50 by 50” is expected to accelerate the demographic transition in emerging economies, increasing the size of the population and the median age of the population as well as the old-age dependency ratio. In these regions, domestic resource mobilisation through obligatory social health insurance and social security contributions could fall short of what is needed to provide health and related social services for aging populations. Increased government transfers from taxes will probably be required to ensure stable and adequate funding. Additionally, economic growth has tended to increase the cost of healthcare without a commensurate increase in health sector productivity, a phenomenon known as the Baumol effect (panel 8). Thoughtful adoption of technologies such as clinical support tools based on artificial intelligence (AI) might partially counter the Baumol effect by increasing health sector productivity or reducing costs. One study estimated that AI could realistically reduce healthcare costs by 5–10%.^150^

#### Panel 8: The Baumol effect

In the mid-1960s, the economists William J. Baumol and William G. Bowen were trying to understand the economics of the performing arts.^151^ They were surprised to find that while musicians were not becoming more productive, their wages were rising. A string quartet performing the same piece of music for the same amount of time earned far more in 1965 than in 1865. Their explanation, called the Baumol effect, has profound implications for healthcare costs: salaries of workers in jobs that see no productivity gains, like musicians and the care dimension of healthcare work, rise in response to rising salaries in other jobs that did see such gains, like manufacturing. As Lee explains in his obituary of Baumol: “An arts institution that insisted on paying musicians 1860s wages in a 1960s economy would find their musicians were constantly quitting to take other jobs.”^152^ Just as the string quartet cannot increase its productivity by playing faster, many health workers and school teachers cannot increase their productivity because their human clinical interactions take time and labor. Recorded music and computerised instruction do, of course, increase the reach of musicians and teachers, but demand remains for the in-person experience, for which there are no productivity gains.

In a chapter on rising healthcare costs in Baumol’s book “The Cost Disease: Why Computers Get Cheaper and Healthcare Doesn’t,” ^153^ Pablos-Méndez and colleagues say that too often policymakers blame this rise on aging populations and expensive new health technologies. The Baumol effect, also known as Baumol’s cost disease, is an important driver, they argue.^154^ But they note that this effect is not just caused by “differential productivity levels in different sectors of the economy,” but also by demand for healthcare. If people’s incomes are growing from productivity gains elsewhere in the economy, “people seem willing to pay the increasingly high prices for health services,” which puts an additional upward pressure on the price of such services.

While new technologies in health can indeed raise costs, they can also decrease them. New vaccines against rotavirus infection, for example, cost far less than treating severe diarrhea in a clinic or hospital. GH2035 stressed the importance of the cost-saving (or outcome-improving) impact of new technology in countering demographic and other pressures, including the Baumol effect, that may lead to rising costs. Looking forward to 2050, GH2050 sees demographic pressures to increase costs as dominant, which, combined with the Baumol effect, makes preparing for the fiscal fallout of the demographic transition more necessary and urgent.

More research is needed to understand how health and social care systems can adapt to an aging world. Rapidly aging middle-income countries could benefit from external assistance and greater cross-country collaboration to support this adaptation and build an evidence base for action in the face of limited public resources. Previous work by the World Bank )^155,156^ has identified several key measures that countries could consider. Most of these policies are outside the health sector, such as labour market policies to help parents balance career and family formation goals. Nevertheless, the health sector has an important role to plan in promoting healthy aging, especially through (i) interventions like excise taxes and hypertension treatment that prevent the incidence of disabling and costly NCDs, and (ii) measures to control unproductive cost escalation, like reference pricing and capitation. These were discussed extensively in GH2035.^2^ The WHO Kobe Centre for Health Development has identified several best practices regarding sustainable financing for long-term care services, including the design of benefits and benefits packages for older populations.^157,158^ Heller has drawn lessons from Japan that emphasize early action to address the macroeconomics of aging and avoiding enshrining specific ages for defining entitlements.^159^

### Shifting domestic financing towards drugs for priority interventions

Section 5 of this report proposes mCEA as a means of prioritising interventions and shifting budget allocations towards the delivery systems for these interventions. Yet many countries struggle to fully cover the cost of essential interventions, including those essential medicines and other services listed in HBPs (appendix table 6.1 on page 71 of the appendix). The result is that some or all the cost of these interventions ends up being financed OOP, creating a major barrier to access and a source of financial risk.

The rise in OOP spending worldwide over the past decade is a major concern (appendix figure 17 on page 70 of the appendix). However, we stress that not all OOP spending is of equal concern. OOP spending on interventions that are included in the HBP should be avoided. However, interventions that are not included must be paid for OOP. It is not possible to maximise health and minimise OOP and associated catastrophic and impoverishing health expenditures at the same time with the same set of interventions; there will always be trade-offs depending on how much a population values financial protection compared to health.^160^ Government attempts to provide universal public finance for low value for money health interventions are best thought of with the same rationale (if any) that would underlie subsidy of any consumer good. An area of concern for the Commission is the increasing adoption of high-cost technologies (e.g., chronic haemodialysis, novel cancer drugs) in countries that still have suboptimal implementation and high OOP costs of core interventions for the I-8 and NCD-7. While an HBP may include some high-cost interventions on general subsidy grounds, the opportunity cost (e.g., in terms of excess child or maternal deaths) of funding these interventions will ideally be made explicit.

Irrespective of income levels or financing mechanisms, OOP remains a primary source of financing for essential drugs in many countries. In countries like Canada, Egypt, Mexico, and Nepal, OOP expenses for drugs in recent years have exceeded 1% of GDP (table 13). A study conducted in Brazil reported that OOP spending on drugs accounted for two-thirds of the catastrophic health expenditure in 2016.^161^ Furthermore, in many countries, the disease-specific financial burden resulting from OOP spending on drugs is substantial (appendix table 6.2 on page 73 of the appendix)—for example, an estimated 3 million Americans with diabetes (1 in 10 out of all Americans with diabetes) incurred catastrophic spending on antidiabetic drugs in 2020.^162^

Inadequate access to essential medicines and high OOP costs constitute a major threat to achieving 50×50 (appendix table 6.1 on page 71 of the appendix). The story is similar across many interventions in many countries.^163^ Often, the government makes a promise that interventions (e.g., treatment of drug-sensitive tuberculosis) are free and available at public sector facilities. Indeed, the consultation, if available, may be free, because the health worker’s salary is paid directly from the government, but the drugs (in this case, for tuberculosis) are often not free. Such drugs are commonly stocked out, and the patient is then forced to purchase the drugs themselves OOP at a private retail pharmacy, or simply do without. Diagnostics essential for determining treatment (e.g., nucleic acid amplification tests for tuberculosis) may also be lacking.

The Commission proposes a pragmatic workaround for steering resources to priority interventions and reducing OOP. We call this the “Arrow mechanism,” named after the late Kenneth Arrow, the Nobel Prize winning economist and GH2035 author who developed the mechanism to be applied to malaria drugs (panel 9). The Arrow mechanism involves four key components: (i) redirecting general budget transfers to ministries of health to line item budget transfers for specific priority drugs; (ii) pooled purchasing, quality assurance, and a long-term commitment to manufacturers to ensure a steady supply of medications; (iii) procurement in sufficient volume to ensure availability; and (iv) use and strengthening of existing supply chains, both public and private. Such a mechanism was implemented through the Affordable Medicines Facility-malaria (AMFm), a highly successful development assistance initiative that improved the availability of quality-assured artemisinin combination therapy—in part by leveraging private-sector delivery networks—and reduced the prices of such therapy at the point of use while increasing availability. One elegant feature of the Arrow mechanism is that its implementation does not require sophisticated financing arrangements. It can be effective even in countries that use line-item budgeting, and in these sorts of countries it might also be the quickest and most direct way to increase access to essential medicines. It also engages the private sector in the implementation of the health benefits package, potentially increasing effective coverage.

Panel 9 reflects on how a domestic AMFm-like mechanism could be developed for NCDs, which are often the medicines that are the least available and affordable in many countries. Of course, countries have additional policy options beyond the Arrow mechanism to improve the affordability of, and access to, essential medicines.^164^ As discussed in section 9, a portion of development assistance for health could be allocated to fostering collective action on essential medicines, including but not limited to reestablishment of an Arrow mechanism for critical drugs and commodities.

#### Panel 9: Public subsidies for NCD medicines: lessons from the AMFm?

The Affordable Medicines Facility–malaria (AMFm) was an innovative package of financing and incentives to expand access to affordable artemisinin-based combination therapies (ACTs) and to displace oral artemisinin monotherapies from the market. The purpose of introducing a multidrug combination was to forestall resistance to artemesinin, and to do so in a way that assured availability and affordability of ACTs. Hosted by the Global Fund to Fight AIDS, Tuberculosis, and Malaria, it operated through the private (for profit and not-for-profit) and public sectors. The origins and design features of the AMFm have been described elsewhere in detail.^165,166^

The design of AMFm incorporated three elements: price reductions through negotiations with manufacturers of ACTs; a buyer subsidy, via a co-payment at the top of the global supply chain; and managerial and administrative interventions to promote appropriate use of ACTs. In practical terms, it sought to reduce retail price of ACTs sold in the private sector, from up to US$11 per treatment to the same price as chloroquine or sulfadoxine-pyrimethamine (about $0·50) and to less than the cost of oral artemisinin monotherapy (about $3–7). Patients who received malaria treatment through public-sector clinics and not-for-profit services would also benefit from increased access to free or low-cost ACTs. It was launched in 2009 and lasted through the end of 2012.

An independent evaluation of the effect of AMFm on quality-assured ACT price, availability, and market share was conducted 6–15 months after the delivery of subsidized ACTs in Ghana, Kenya, Madagascar, Niger, Nigeria, Uganda, and Tanzania (including Zanzibar).^167^ In all pilots except Niger and Madagascar, there were large increases in ACT availability (25·8–51·9 percentage points), and market share (15·9–40·3 percentage points), driven mainly by changes in the private for-profit sector. Large falls in median price for ACTs per adult equivalent dose were seen in the private for-profit sector in six pilots, ranging from US$1·28 to $4·82. The market share of oral artemisinin monotherapies decreased in Nigeria and Zanzibar, the two pilots where it was more than 5% at baseline. The evaluators concluded that subsidies combined with supporting interventions could be effective in rapidly improving availability, price, and market share of quality-assured ACTs, particularly in the private for-profit sector. Nevertheless, the Board of the Global Fund subsequently ended the AMFm.

Several studies have reported on post-AMFm trends in access to and the market for ACTs in malaria-endemic countries. A recent study found that following the reduction or termination of subsidies for d ACTs in Uganda and Nigeria, retail prices of these medicines increased and retail prices of non-quality assured ACTs decreased.^168^ This in turn likely contributed to greater availability and increased use of non-quality assured ACTs.

With the epidemiological transition, shifting disease burdens, and pressures on publicly funded health services, could the AMFm experience be relevant to the quest for better access to affordable and quality-assured medicines and other commodities for NCDs? One possibility is to adapt the AMFm’s design for country-level or regional-level subsidies for NCD commodities, with countries’ ministry of finance (not donors) as the purchasers/payers of the subsidy, and with payments going directly to manufacturers. For the Ministry of Finance, it would count as either part of the health budget or additional funding. Since the expenditure would not be managed by the ministry of health, the mechanism would provide the finance ministry with assurances of no capture (at least in the upstream part of the supply chain). For the Ministry of Health, if the subsidy counted as part of its budget, assuming it remained constant after adjusting for inflation, it would constrain the ministry’s room for allocating resources within the publicly-financed health sector—and might be unattractive for that reason. However, if the subsidy were additional, the reduced factory-gate price would effectively increase the health ministry’s purchasing power compared to the status quo.

There are potential objections to this approach. For example, a country-level subsidy would cause major price differences across porous borders, leading to predictable price arbitrage. Unlike the case for communicable diseases, such arbitrage would not be seen as potentially a net positive because the benefits of using those medicines accrue to the individual, with no positive externalities. Second, there are risks of price gouging by middlemen and retailers. However, such price gouging fears proved mostly unfounded during the AMFm because middlemen and retailers appeared satisfied with a change from low-volume and high margins to higher volume and lower margins per unit sold.

## Section 7. Pandemic prevention, preparedness, and response

With globalization, increasing human population, climate change, and other factors, global vulnerabilities to emerging diseases, including pandemics, are growing. In a prescient volume published in 2011, Nathan Wolfe warned that pandemics with devastating effects, such as the 1918 influenza pandemic and COVID-19, may occur frequently in the 21st century.^169^ COVID-19 had major impacts on human health, with estimated excess global deaths of more than 26 million, and caused enormous economic losses and severe societal impacts. In recent decades, the international community invested in pandemic prevention, preparedness, and response (PPR), but the COVID-19 pandemic clearly highlighted significant deficiencies in PPR systems. Zelikow provides a brief but insightful account of COVID-19 response failures in the journal *Foreign Affairs.*^170^

COVID-19 was very different to previous pandemics, and the next pandemic might be very different to COVID-19. Therefore, there can be no “one-size-fits-all-all” approach to PPR. While we certainly learned important lessons from COVID-19, including that outcomes differed markedly across countries due to the different quality of their pandemic responses, we should not learn the wrong lessons. For example, our analyses suggest that expected annual losses from an influenza pandemic to be about twice as high as for coronavirus. If the past is a guide, influenza deaths will occur at much younger ages than for COVID-19, with significant implications for policy.

### The impacts of the COVID-19 pandemic

On May 5, 2023, WHO Director-General Tedros Adhanom Ghebreyesus declared an end to the emergency phase of COVID-19; “I declare an end to the public health emergency of international concern,” he said.^171^ He made it clear, however, that his declaration by no means was an end to the damage caused by the virus whose effects had first been observed in Wuhan at the end of December, 2019, and where its first known death had occurred a week or two later. Rather, May 5, 2023 marked a transition from the emergency phase to a phase of enduring endemicity. Additionally, he noted that morbidity from Long COVID would continue long after the emergency phase ended.

Just as there were “post-mortems” after the 2014–2016 Ebola epidemic in west Africa ^172,173^, there have been multiple examinations of and reports on interim lessons from COVID-19. These include the *Lancet* commission on lessons learned for the future from the COVID-19 pandemic;^174^ a WHO-convened independent panel chaired by Helen Clark and Ellen Johnson Sirleaf;^175^ and Richard Horton’s book “The COVID-19 Catastrophe: What’s Gone Wrong and How to Stop it Happening Again.”^176^ Such retrospective analyses have laid out a broad range of valuable conclusions that we summarize in panel 10. The context for thinking about pandemic preparedness remains, however, an unhopeful one. In a June 2024 update to their Independent Panel report, Clark and Johnson Sirleaf point to the failure of recent negotiations for a pandemic treaty and an atmosphere of ill will and mistrust among countries and conclude that “too many gaps and vulnerabilities remain, and pathogens have an ample opportunity to spill over, slip though and spread fast.”^177^

#### Panel 10: An agenda for pandemic prevention, preparedness and response

##### Prevention:

With the growing risk of pandemics, we need to increase investment in pre-emptive interventions to minimize the risk. Since most pandemics are zoonoses, risk reduction interventions should address the human-animal interface, including improving animal husbandry practices and regulating wild animal trade. It needs to be acknowledged explicitly that human livelihoods now depend on these practices. Both as a matter of aligning incentives and simple justice, it will be important to compensate these individuals’ losses and facilitate their transition to other lines of work. Strengthening biosafety and biosecurity is also critical to prevent the risk of spill-over transmission in laboratories. We need to better understand the pandemic risk from existing microorganisms in domestic and wild animals. Enhancing animal surveillance by applying new technologies such as deep sequencing and environmental surveillance can contribute to mapping pandemic risk. It is especially important to focus on viruses that are crossing species barriers and causing disease in new species, as we have seen recently with H5N1 influenza. These activities should be implemented as part of the One Health approach.^178^

In addition, it is critical to focus surveillance on humans with fevers of unknown origin, and particularly on severe acute respiratory illnesses. Before a zoonotic virus becomes a virus that readily transmits from one person to another, it typically causes occasional infections of humans, acquired from an infected animal (e.g., recent H5N1 human infections from birds and cattle). Those viruses are far more likely to further evolve such that they are also readily transmissible from person to person as compared to viruses that are not yet even capable of infecting people. Thus, we should focus surveillance efforts on people with known zoonotic exposures and fevers of unknown origin. Ideally, strengthened national laboratories would link into global systems that included, for example, aircraft waste water surveillance.

##### Preparedness:

Preparedness involves being ready for infectious disease events, from a small outbreak to a global pandemic, through improved global, national, and local resilience, including updating pandemic preparedness plans. Consider the sequence of events for which a country must be prepared:

- For rapid containment of an outbreak, the country should drive spread to zero if possible, regardless of the mode of transmission. As long as containment is still possible, this must be the very highest priority and the one for which all countries and regions must be prepared. New pathogens can only be contained with non-pathogen-specific tools; pathogen-specific tools may be available for previously described pathogens (e.g., Ebola vaccines). For rapid containment to be possible, detailed plans must be in place for a variety of possible presentations, and staff must be trained and available to respond, if not at the national level then with regional support. Such preparedness must include how to take care of people with a new pathogen, how to isolate them and quarantine their contacts, and how to reduce the probability of ongoing spread (such as through shelter in place orders), as all efforts are made to contain the pathogen.
- For a pandemic that cannot be excluded from the population, rapid epidemiological characterization is essential so that protection efforts can be focused on those most at risk and so that protection can be relaxed for those at minimal risk, thus reducing the secondary harms caused by strict protection measures. Plans should be in place that consider different phases of the pandemic (e.g., the phase before an effective vaccine is available versus the phase after an effective vaccine is available). COVID-19 taught us that many countries failed to adequately protect the most vulnerable (e.g., the poor, the institutionalized elderly, the incarcerated) and excessively protected those at minimal risk (young children) to their significant detriment.
- For outbreaks of known pathogens, regional and global stockpiling of pathogen-specific drugs and vaccines may be critical to the success of early containment efforts.
- Providing adequate capacity for non-specific supportive care in first level hospitals was a significant challenge during the COVID-19 pandemic. Inadequate critical care capacity, such as ICU beds and mechanical ventilators, was a serious issue even in high-income countries. In low- and middle-income countries, lack of access to essential clinical needs such as oxygen was also a significant contributing factor to high death rates. Rose et al draw out the lessons for investing in clinical capacity to reduce pandemic mortality.^135^
- Development of medical countermeasures, including vaccines, therapeutics, and diagnostics, is critical to preparedness. The global system to ensure equitable access to such measures should also be strengthened. Considering a possible global supply chain disruption, stockpiling essential commodities such as PPE is essential to preparedness. Stockpiling of antivirals for influenza may be an asset in case of an influenza pandemic; many high-income countries have such stockpiles, but there are none in many low- and middle-income countries.

##### Response:

There are three strategic objectives for response: containment, suppression, and mitigation. Containment is to interrupt all chains of transmission and usually requires aggressive measures. Suppression is to minimize transmission down to low levels. Mitigation is to slow down the spread so that the peak of incidence becomes lower (“flattening the curve”). A response can be divided into two phases: early and late. Early response starts after detecting a pathogen with pandemic potential and lasts until widespread community transmission. Local or global containment might be a feasible option if early signs of a pandemic are detected. SARS was successfully contained globally within six months after the first recognition of the outbreak. COVID-19 was much more difficult to contain, but containment or suppression was achieved in many Western Asia Pacific countries during the early phase of the pandemic. Various PHSMs were implemented during the COVID-19 pandemic in most countries. Some PHSMs, such as stay-at-home orders and school closures with enormous negative social and economic impacts, were implemented for an extended period in some countries. More research on PHSMs should be conducted to provide science-based guidance, including, importantly, on the extent to which such measures are population-initiated,^179^ and the timing and means for safely ending them. Early response for containment or suppression likely to reply on PHSMs since vaccines will not be available in the early stage of a pandemic. Proper guidance on PHSMs should be developed quickly. Therapeutics may also not be available in the very early stage of the pandemic except for antivirals for an influenza pandemic. Diagnostics should be rapidly developed, validated, and distributed. Early detection and isolation of cases is critical for early containment or suppression, which requires widely available and reliable diagnostics. Mitigation can be done with PHSMs, medical countermeasures, and proper clinical management. Vaccines may play essential roles in mitigating the impact of the pandemic in later phases.

With the end of the emergency phase now more than a year behind us, the statistics are in — in at least in a preliminary way — that allow us to assess the consequences of the pandemic at a country level. Without available peer-reviewed COVID-19 data for 2022 and 2023, we use estimates of excess deaths during the pandemic provided by the *Economist* magazine, available 2020–2023.^180^ We acknowledge the limitations of using a nonstandard data source. However, the *Economist* has a dedicated data science team whose estimates are widely used, with all methodology thoroughly documented and publicly available.^181^ Further, the *Economist* relied on similar data sources as WHO’s published excess death estimates for 2020–2021.^77,182^ Therefore, the estimates for 2020–2021 from the WHO and the *Economist* have a high correlation (>0.98) and only one of the 30 most populous countries had a difference in excess deaths greater than 2% between the two sources (Japan, with an 8% difference). However, the *Economist* data for 2022–2023 may have larger discrepancies: we will update our excess death estimates using mortality data from the United Nations World Population Prospects 2024, available soon.

Table 14 ranks the 30 most populous countries using a reasonable overall metric of their performance in tackling COVID-19, the P-score. Appendix table 7.1 on page 77 of the appendix rank orders all countries with populations over 5 million and appendix table 7.2 on page 80 of the appendix provides our estimates of excess deaths for all countries alphabetically as well as excess deaths per million population and P-score. The P-score is derived from excess deaths during the period of the emergency as a percentage of the number of deaths that would reasonably have been expected had the pandemic not occurred. For the world as a whole, we report an estimate that the emergency period — January 2020 to May 4, 2023 — had just over 26 million excess deaths. As Table 14 shows, this was about 13% of the number of deaths otherwise expected to occur. This 13% is labelled the pandemic’s P-score for all age groups. Excess deaths per million population is a constituent of the P-score, hence it is closely related to it (see appendix table 7.1 on page 77 of the appendix). By taking the baseline number of expected deaths also into account, the P-score potentially adjusts for other factors, such as age distribution, and so it is our preferred measure. As this report goes to press, estimates of losses from COVID-19 continue to evolve, and the numbers we report should be viewed as early—although probably reasonable—approximations.

Japan’s P-score of 3.8% was the best among the 30 most populous countries, and China’s was second best. The lowest performers were Mexico, India, and Ethiopia, all with P-scores of 21%, and Russia at 26%. Only three low-population countries out of 123 countries with populations greater than 5 million had P-scores better than Japan’s, with New Zealand the lowest at under 1%. Ecuador and Peru were the only countries with P-scores higher than Russia’s, with values of 27% and 32%, respectively. Perhaps the single most striking thing about table 14 is the huge range in performance among the most populous countries —a range of almost 7 to 1. Table 14 also shows individual year P-scores for 2020 and 2021, and for the 16 months from January 2022 to the end of the emergency. In 2020, country performance varied much more widely than for the total period. Pablos Mendez et al and Jamison and Wu point to an “East-West divide” in 2020,^183,184^, with a factor of up to 100 to 1 separating countries. Early response in the East, including serious efforts to isolate infectious individuals,^185^ was able to effectively control the original virus. The first academic publication on the pandemic from China appeared in the *Lancet* on January 24, 2020,^186^ and warned of a pandemic risk. China, Thailand, Hong Kong, Taiwan, and Japan had all initiated serious responses by then. In sharp contrast, as Clark and Johnson Sirleaf had observed, February 2020 was a lost month in the West.^175^ Failure to control transmission elsewhere in the world created opportunities for the virus to evolve into far more transmissible variants. The approaches to control that worked well for the original virus appear to have worked less well later in the pandemic.^187^

**Figure 18:**
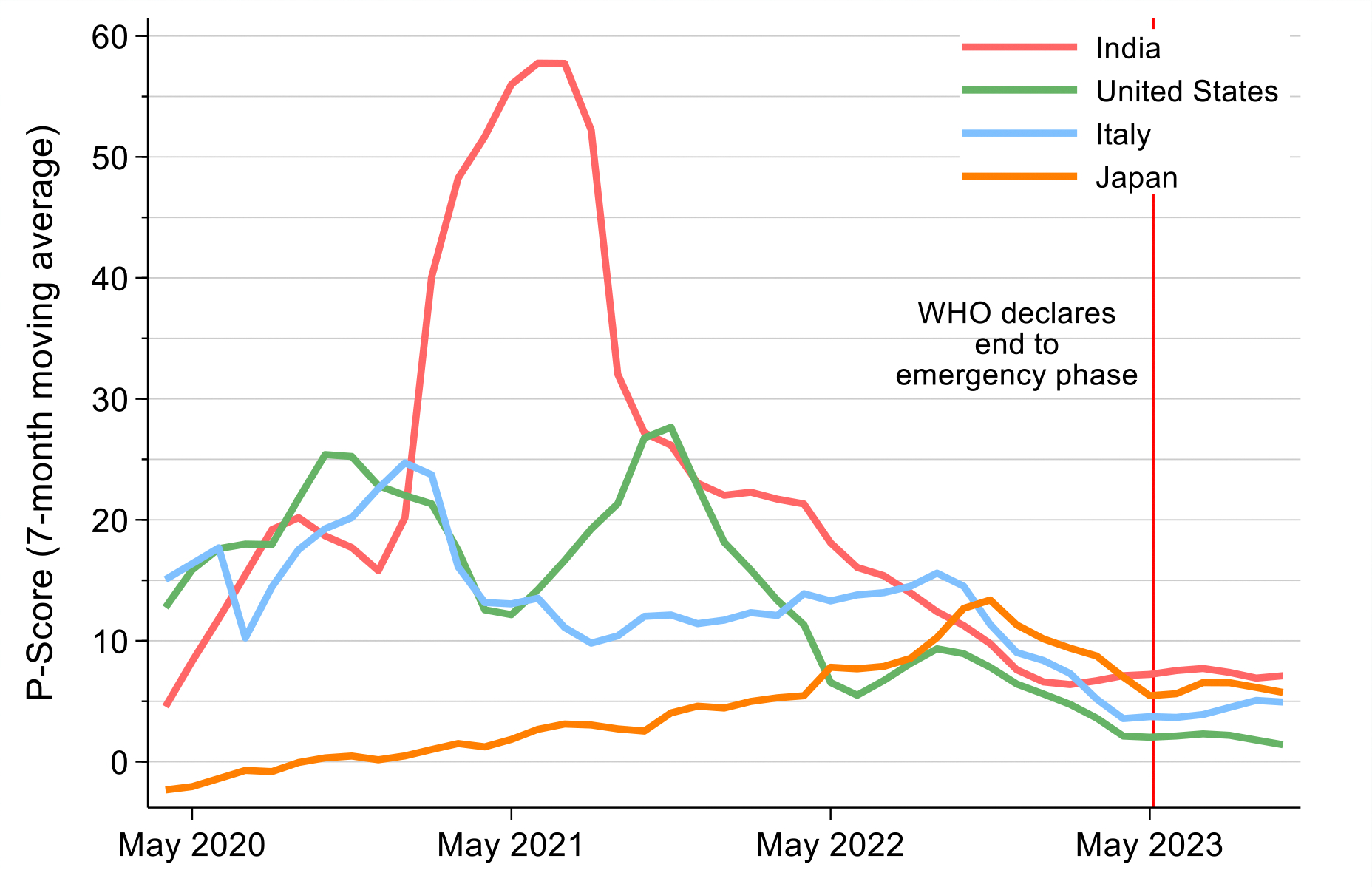
P-score by month: Italy, United States, India and Japan — April 2020 to October 2023 Note: The graph highlights the time taken for certain regions—namely the North Atlantic, China, the World, and India—to halve their PPD values to 2019 levels. Data source: Excess deaths data were downloaded from Our World in Data. Projected deaths were based on deaths in 2019 and the annual change (averaged between 2015–2019) from reference 35 ^11^. Annual projected deaths were divided by 12 (i.e., death baseline were assumed to be uniform across each year). P-Scores were calculated by dividing excess deaths by the projected deaths.

While we believe that the P-score for the emergency period provides a good overall measure of country performance, it incorporates into a single number the different potential values for different time periods and age groups. Figure 18 shows how the P-score was distributed over time in very different ways in Italy, India, Japan, and the US — information that could be highly relevant to understanding different waves or the timing of different response policies. Japan shows remarkably good control early in the pandemic but then a decline in performance as more transmissible variants came to dominate. India shows a major peak mid-way through the pandemic. Figure 18 points to the possibility of more fine-grained assessment to complement the broader picture provided by aggregate P-scores. Likewise, age-disaggregated analyses will likely prove informative.

Economists measure the welfare loss associated with mortality in monetary terms by assessing empirically the value that individuals assign to reducing by small amounts the mortality risks that they might face. We discussed this approach in section 2, when we described the development of a measure of income, called “full income,” that is more comprehensive than GDP per capita. Full income is a measure that incorporates the value of mortality declines. Table 14 reports an estimate of the value of mortality loss — only mortality — associated with the pandemic using this full income metric. For the world as a whole, the value of loss from the pandemic for the period January 2020 through May 4, 2023 reached about 39% of the value of global income in 2019, or on the order of $40 trillion.

Loss of GDP constitutes only part of overall loss in full income, but an important one for the functioning of economies. The IMF provided an early estimate of GDP loss for the world that was as high as $13.8 trillion^188^, and in an early assessment of the economic consequences of the pandemic for the US, Cutler and Summers estimated a loss of $16 trillion over a 10-year period, of which about $7.5 trillion was in GDP loss.^93^ Since the time of its 2022 estimates, the IMF has slightly reduced its estimates of the impact of the pandemic on annual economic output in most parts of the world.^28,189^ The exception was in low-income countries, where the IMF now estimates that 2024 GDP will be more than 7% lower than it otherwise would have been.

### Future pandemic risk

COVID-19 provided compelling evidence that pandemic risk remains very much a feature of the world we live in. Between the time of the great influenza pandemic of 1918 and COVID-19, there were at least four influenza pandemics and two global coronavirus outbreaks. Each of these was deadly, although far less so than COVID-19. Additionally, there were more geographically limited epidemics of viral hemorrhagic fevers (VHF), such as the Ebola virus outbreak in West Africa in 2014–2016. While the VHFs have had limited geographical range and death totals they have nonetheless caused widespread fear and economic disruption. While this section does not deal explicitly with VHFs or similarly geographically limited epidemics, many of our recommendations on preparation and response apply as well to these risks. The message is clear: risks remain very much with us. But, just how big are the risks that the world faces?

As mentioned in section 2, Madhav and colleagues, in an assessment prepared for CIH 3.0 and the Disease Control Priorities Project, have applied the techniques of quantitative disaster modelling to provide insight into the question of the magnitude of risk.^47^ They attempt to quantify probabilities of the sparking of a pandemic — typically the point of transition to humans from another animal host — and to its spread. They use historical and biological data to simulate tens of thousands of possible evolutions of global respiratory pandemics caused by viruses in either the influenza or coronavirus families. Each of these pandemics differs in its transmission and mortality characteristics and in the level of mortality that ensues. For example, COVID-19 was distinctive in the extent to which the elderly were at risk for mortality—and children far less so. However, the next pandemic may show a very different age distribution. Aggregating the simulations provides a picture of the relationship between the potential mortality level of a pandemic and its likelihood — the so-called exceedance probability function.

Table 15 summarizes Madhav and colleagues’ results with four points on the exceedance probability function, typically expressed as annual risks. Their simulations point to an over 6% probability of a pandemic next year involving a million or more deaths and a 2–3% probability of a pandemic involving 25 million or more deaths. Other columns project these risks out over longer time periods. For example, the table reports their finding of a greater than one in five chance over the coming 10 years of a pandemic that kills 25 million or more (equivalent to the number of deaths associated with COVID-19).

In most years, of course, there will be zero pandemic deaths. In some years, there will be a million or many million pandemic deaths. It is useful to think of Madhav and colleagues’ results as conveying that, on a long-term average, there would be 2.5 million deaths per year. Of these, 1.6 million would be expected to be from an influenza pandemic and 0.9 million from viruses in the coronavirus family. To place the 2.5 million number in context, it is about the same as the number of deaths currently occurring annually from HIV/AIDS, tuberculosis, and malaria combined and much higher than the number of annual climate change deaths projected from even very pessimistic scenarios in coming decades.

There is much uncertainty attached to the number of malaria deaths that occurred in any recent year. So, it is no surprise that vastly greater uncertainty would attend the assumptions and modelling efforts to generate estimates of mortality from pandemic risk. Madhav and colleagues stress the multiple sources and magnitude of uncertainty around the results of their simulations. Their results should be ones that broadly position our thinking rather than being held up as more than approximate; this positioning of our thinking points to very high levels indeed of future risk. One element of uncertainty concerns the rate at which pandemic risk is likely to increase in coming years, and most experts judge that risk to be increasing. Madhav and colleagues acknowledge this possibility but nonetheless construct conservative estimates on the basis of non-increasing risk.

Fan et al assessed the expected economic value of losses associated with earlier estimates of pandemic risk.^190^ In table 16, we update those estimates, which convey the pandemic risk results for each of the CIH regions. It conveys those results in terms of expected annual deaths and the implications of those deaths for years lost of life expectancy, for increase in the PPD, and in terms of the value (as a percent of GDP) of expected annual loss.

### Pandemic prevention, preparation and response (PPR)

There have been at least four influenza pandemics since 1918. These influenza pandemics were significantly different from the COVID-19 pandemic. There are many possible scenarios for the next pandemic. One of these possible scenarios is a SARS-like scenario. SARS had a high case fatality ratio (CFR) with a CFR of about 10%. However, containment was a feasible option for SARS due to certain epidemiological characteristics, including no or very little pre-symptomatic transmission. SARS was successfully contained without vaccines or antivirals within six months after first recognition, mainly by public health and social measures (PHSMs), including active case finding and contact tracing. If we had a SARS-like scenario, i.e., high CFR and containment as a feasible option, we should aim for rapid containment with PHSMs and not wait for a vaccine to be developed.

Another possible scenario is one akin to the 1918 influenza pandemic, which lasted from 1918 to 1920, and which was estimated to have killed about 50 million people. There were some significant differences between COVID-19 and the 1918 influenza pandemic, including the age distribution of deaths and the duration of the pandemic. For COVID-19, most deaths have occurred in the elderly. However, the age distribution of fatal cases of the 1918 influenza pandemic was different, as most deaths occurred in young adults and children.

**Figure 19:**
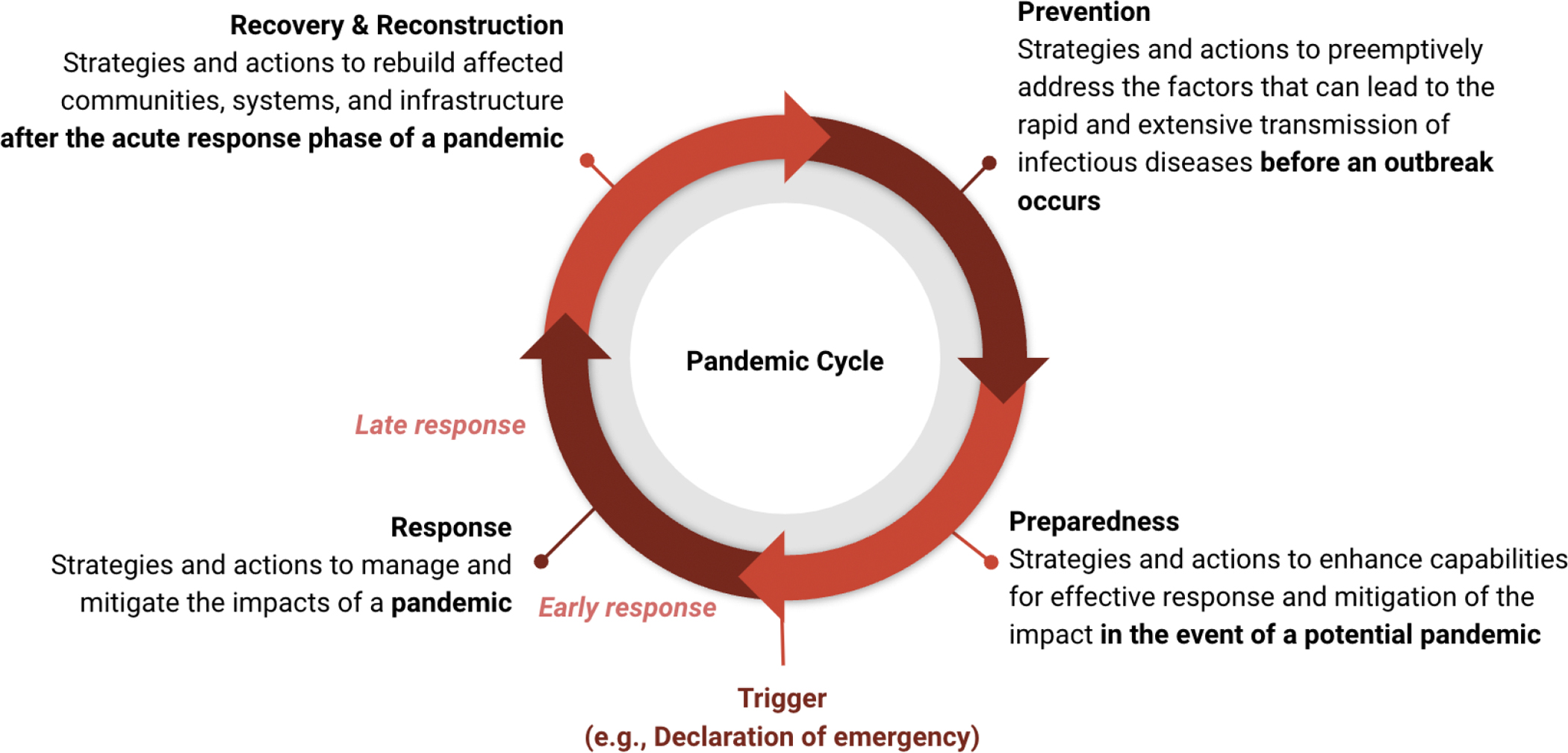
Framework for the phases of the pandemic cycle

Figure 19 shows a framework for depicting the unfolding phase of a pandemic and corresponding points of intervention: prevention, preparedness, response, and recovery and reconstruction. Various capacities and systems are required for each phase.^192^ Panel 10 brings together the substantial list of generally agreed elements for how a country, and the world, could prepare.

Several critical components of PPR, such as national preparedness plans, basic stockpiling of critical drugs and equipment, and surveillance for monitoring, are considered to be national public goods.^30^ However, other components, particularly those necessary for risk reduction and early response, should be regarded as global public goods (GPGs). Most elements depicted in Figure 19 from prevention through early response have elements of GPGs. These GPGs include interventions at the human-animal interface, mapping the pandemic risk, R&D and equitable access to medical countermeasures, surveillance for early warning, and capacity for early response. Centralized stockpiles can be important for VHF preparation. Global financing mechanisms should be sought for these items, including potentially to middle-income countries, for whom there is low priority for official development assistance (ODA). Likewise, most finance ministries, particularly in low-population countries, may reasonably see little benefit in using national resources for GPGs. It is reasonable to expect that if the advanced economies fail to support worthwhile pandemic prevention and surveillance efforts in poorer, low-population countries, then the countries themselves would see little reason to do so—given that most of the benefits lie outside their borders.

In March 2022, in an analysis prepared for the G20 Joint Financing and Health Taskforce, the WHO and the World Bank estimated that the total annual financing need for the PPR system is $31.1 billion.^193^ Their analysis noted that “at least an additional US$ 10.5 billion per year in international financing will be needed to fund a fit-for purpose PPR architecture.” The Independent Panel points to the additional need of over US$ 10 billion per year for agricultural (One Health) measures,^177^ and Glennerster et al point to very high probable benefit to cost ratios from these investments.^194^ At the 2022 Global Pandemic Preparedness Summit, governments committed to investing in PPR, including in the “100 Days Mission”—the mission of developing diagnostics, therapeutics, and vaccines within 100 days of the start of the next pandemic.^195^ However, the limited current investments are heavily focused on vaccine R&D, and there is under-funding of therapeutics and diagnostics R&D. PHSMs are critical, yet there is insufficient investment into strengthening them and studying their effectiveness. The WHO and World Bank also report stressed the importance of surveillance and early warning systems.

While it is understandable that much of the focus of the global community is on pandemic vaccine R&D, we neglect preventive and other aspects of PPR at our peril. Without containment or suppression efforts using PHSMs, most deaths in the next pandemic might occur within the first 3–6 months. Even hitting the 100 Days Mission target for vaccine development may not be fast enough to save a huge number of lives. And while multiple safe, highly effective COVID-19 vaccines were developed in under a year, there is no guarantee that safe, effective vaccines will be developed this quickly—or even at all—in the next pandemic. Vaccine nationalism may also prevent the international system from accessing and procuring vaccines and distributing them equitably worldwide—such vaccine nationalism was a major constraint to COVAX (COVID-19 Vaccines Global Access) in its attempt to achieve global COVID-19 vaccine equity.^170,196^

Essential elements of the vaccine development process include ensuring that protection and sharing of intellectual property reflects both societal needs and the often substantial public investments being made. The WHO Council on the Economics of Health for All,^197^ chaired by Mariana Mazzucato, further develops these points.

#### Preparing for an unprepared world

This section has summarized the enormous human and economic losses from COVID-19. It has presented new assessments showing that the risk remains high for one—or more—major pandemics in the time frames considered in our report, i.e., more than one chance in three of a pandemic killing 10 million or more people by 2035 and as high as one in seven of a pandemic killing 100 million or more by 2050 (table 15). The current responses to this level of risk, however, reflect no sense of urgency.^198^ For example, earlier in 2024, evidence appeared of widespread infection of cattle in the US with H5N1 influenza. Transmission among mammals raises the dangerous prospect of viral evolution into efficient human-to-human transmission. And yet, on April 24, 2024, Zeynep Tufekci, *New York Times* columnist, wrote: “….having spent the past two weeks trying to get answers from our nation’s public health authorities, I’m shocked by how little they seem to know about what’s going on and how little of what they do know is being shared in a timely manner.”^199^ A country that itself commits to being prepared will, it seems, need to plan for dealing with the next pandemic in a world that is not prepared.

## Section 8. Accelerating progress using taxation–especially on tobacco

Earlier in this report, we showed the feasibility of all nations converging on low levels of premature mortality by the middle of the century (section 3) and we made the case that a focus on 15 priority conditions would be key to achieving such transformative gains in global health (section 4). We also examined the health sector interventions that would be needed to reach “50 by 50” and how these could be funded (sections 5 and 6). In this section, we argue that complementary fiscal, regulatory, and information interventions could play a critical role in accelerating progress towards a convergence on low PPD by 2050. The most important of all these interventions is raising taxes on tobacco.

In a chapter on intersectoral policy priorities for health for DCP3, Watkins and colleagues argue that “a range of policies initiated by or in collaboration with other sectors, such as agriculture, energy, and transportation,” can have a large effect in reducing the incidence of disease and injury.^115^ The authors identified a package of 29 intersectoral policies against a wide range of conditions from both the I-8 and NCD-7 (see appendix table 8.1 on page 87 of the appendix). Although these are intersectoral interventions, ministries of health could play key analytic and advocacy roles, fulfilling their mandate across government departments.

Intersectoral interventions make use of four main types of policy instruments. The first is legal instruments—regulations and laws. Examples include halting the use of unprocessed coal and kerosene as household fuels to reduce indoor air pollution and regulating the advertising, promotion, packaging, and availability of tobacco, with enforcement, to help curb tobacco use. The second is engineering instruments to improve the built environment, such as engineering roads to separate vehicles from vulnerable pedestrians, so as to reduce road injuries. The third is public health information in the form of focused information and education, such as providing consumer education to curb excess salt and sugar intake and to reduce the risk of sexually transmitted infections. Ongoing research that generates epidemiological knowledge and that is disseminated via media and social networks can also be considered a key tool of government support for information. The fourth, the focus of section 8, is fiscal instruments, i.e., taxes and subsidies.

Although our discussion focuses on taxation, other instruments will often prove relevant. An important and neglected example is regulation to control lead pollution and its often severe consequences on domains ranging from child cognition to cardiovascular risk.^200,201^ Silverman Bonnifield and colleagues provide an up-to-date overview of the lead problem and of the role of regulatory success in addressing it.^200^

As we did in GH2035, we advocate in particular for the use of economic policies—especially changing the prices of products through taxes and subsidies. These policies are a powerful and enormously underused lever for improving public health. We focus on several of the most important risk factors that are amenable to such policies: smoking, alcohol, ambient air pollution, and possibly diet. Of these, tobacco use is by far the most important in most countries and the most actionable, given a very large amount of data on the effectiveness and feasibility of large excise tax increases. We do not discuss broader social determinants of health, such as income and education, as we previously discussed these in detail in GH2035.

### Tobacco taxation: highly effective and pro-poor

Although it is common to hear that other risk factors are “the new tobacco” or “the new smoking”—for example, “sugar is the new tobacco” ^202^ and “sitting is the new smoking”^203^—we believe that “tobacco is the new tobacco.” Smoking remains the biggest avoidable cause of death in many populations worldwide and NCDs strongly linked to tobacco is one of the most important categories of our NCD-7. Smokers who start early in life and do not quit can expect to lose at least 10–13 years of life compared to otherwise similar never smokers.^204,205^

If current smoking patterns persist, tobacco will kill about one billion people this century.^206^ About 40% of the world’s cigarettes are consumed in China, almost entirely by men, and smoking already causes around 20% of all deaths in middle age in Chinese men.^207^ Across the world, people with low income suffer disproportionately from the health and economic consequences of tobacco,^208^ with smoking accounting for about half of the differences in mortality risk between men of lower and higher social strata.^209,210^

To reduce tobacco-attributed mortality between now and 2050, the key need is cessation by those who smoke; avoidance of initiation will help chiefly in the second half of the century. The benefits of cessation are seen surprisingly quickly. Smokers who quit before the age of 40 years avoid over 90% of the excess mortality risk during their next few decades of life compared to those who continue to smoke.^209^ However, cessation rates are low in several countries in Asia (e.g., China, India, Indonesia), Russia, and Central Europe.^209^

The most important way to promote cessation, prevent initiation of smoking, and drive down tobacco use is excise taxes on tobacco, a policy tool that is still greatly underused despite its effectiveness. In its 2019 report “Health Taxes to Save Lives,” the Taskforce on Fiscal Health Policy, co-chaired by Michael Bloomberg and Lawrence Summers, noted that “raising taxes on tobacco can do more to reduce premature mortality than any other single health policy.”^211^ The Taskforce’s analysis suggested that a steep rise in tobacco prices could avert more than half a million tobacco-related deaths per year, on average, over the next half century. Although taxation may be the most powerful instrument for reducing initiation and use of tobacco products, and encouraging cessation, complementary regulatory and informational measures are also important.

A 100% increase in the price of tobacco in low- and middle-income countries results, on average, in a 40% decline in consumption, which includes about 20% of smokers quitting and 20% reducing their daily use.^212^ Many countries have used large excise taxes to successfully reduce consumption and raise revenues, including Brazil, Colombia, the Philippines, and South.^213^ Study after study has shown that higher tobacco prices can reduce illness and death. For example, increased prices reduce cardiovascular, respiratory, and cancer deaths, severity of childhood asthma, and hospitalization for heart failure failure.^214–216^

One of the arguments that opponents of tobacco taxation make is that taxing tobacco hurts the poor, but this is a myth.^217^ Tobacco taxes are highly progressive (panel 11). People with lower incomes are more price-responsive, so are more likely to reduce their tobacco consumption or to quit when taxes are raised.^218^ As a result, say Pareje et al, “they benefit disproportionately from longer healthier lives, reduced spending on healthcare, fewer lost days of work, and longer working lives.”^218^ In 36 countries, revenues raised from tobacco taxation have been spent on programs that favor the poor,^219^ making the impact of the taxation even more progressive. Cigarette taxation levels are unrelated to smuggling,^212^ and large scale smuggling occurs only with active tobacco industry encouragement; new track and trace technologies are now being used to combat such fraud (panel 12).

#### Panel 11: The pro-poor nature of taxes on tobacco, alcohol, and sugar-sweetened beverages

Effective interventions such as deploying smoke-free environments, banning advertising, and taxing cigarettes have been forcefully implemented under the Framework Convention on Tobacco Control (FCTC).^218^ Ratification of the FCTC, ideally paired with large tax increases, has yielded marked reductions in young adult smoking and has increased cessation. Yet, as of today, tobacco taxes remain the least implemented of the six tobacco control interventions included in the MPOWER package, an intervention package aligned with the FCTC. In 2022, only 40 countries (about 10% of the world’s population) were enforcing taxation levels on a par with the recommended tax rates of 75% or more of cigarette prices.

Opponents of tobacco tax increases, including those from the tobacco industry, argue that such taxes hurt the poor, i.e. they are regressive.^220^ Taxes are considered regressive when the expenditures incurred by the poor represent a greater share of their income as compared to the rich.^221^ In other words, if tobacco taxes were regressive, increased tobacco taxes would lead to a proportionally greater ratio of net cigarette expenditures relative to income among poor versus rich smokers. However, poorer smokers are more sensitive to tobacco price hikes than richer ones. The ensuing reductions in smoking participation and tobacco consumption could thus be far greater among the poor.^222^ With large price increases, the distribution in net cigarette expenditures relative to income could well be progressive.^220,223^

In addition, the classic definition of regressivity solely examines net cigarette consumption relative to income. As such, it overlooks the full array of health and financial consequences of tax hikes. Anticipating the comprehensive impacts of increased taxes among the poor versus rich is therefore of paramount importance. Increased tobacco taxes can be progressive when it comes to health consequences, since they lead to large reductions in premature mortality and morbidity. Through preventing and controlling tobacco-related diseases (e.g., cancers, heart disease, stroke, and pulmonary disease), tobacco taxes eventually crowd out public healthcare costs and out-of-pocket expenditures linked to the treatment of these diseases, and crowd out substantial productivity losses. As a result, they reduce medical impoverishment and deliver financial risk protection, especially for the poor and when preexisting levels of public finance and health insurance are low. Several extended cost-effectiveness analyses have established this: the overall impact of increased tobacco taxes is progressive when accounting for outcomes of health benefits and financial risk protection.^223–229^

While less commonly examined, other health taxes, such as on SSBs or alcohol products, may exhibit similar impacts.^218,230–232^ That is, the progressivity in health benefits (e.g., reductions in mortality and morbidity associated with diabetes or liver cirrhosis) would mimic the pre-tax distribution in risk factors (e.g., obesity, consumption of SSBs/alcohol) across income groups. Likewise, the progressivity in public cost savings and financial risk protection gains would result from the underlying organizational mix of public vs. private finance among the population, among the poor versus rich. Therefore, the overall progressive or regressive nature of such increased health taxes on health benefits and financial protection would greatly depend on a country’s epidemiological and health system context.

#### Panel 12: Secure track and trace technology to fight the illicit trade of cigarettes

In most countries, cigarettes are subject to excise duty, making these products less affordable—the increased prices drive smoking cessation and discourage initiation. Tobacco industry profits were about USD 50 billion in 2010,^205^ creating strong incentives for the industry to keep taxes low so it can maximize its own profit margins. High levels of tobacco taxation have not been linked to smuggling at large scale, which occurs only with the active role played by the tobacco industry.^212^ The tobacco industry smuggles its own products to maintain market share of its brands and to intimidate finance ministries. For example, international tobacco companies organized smuggling in the mid-1990s from the US into Canada, and this led to a short term reduction in tax rates and most notably a large increase of about 30–40 billion excess cigarettes.^233^ The excess cigarettes will eventually cause about 30–40,000 excess deaths from smoking.

Traditional measures to fight fraud consist of having tax inspectors stationed at key points of the supply chain (manufacturing plants and warehouses) and observing the production and movement of goods. However, these controls require strong overall customs and revenue capacity that are resistant to corruption pressures.

Secure track and trace solutions are increasingly being adopted to strengthen controls and complement the work done by inspectors. Such solutions are part of the tracking and tracing obligations under the Protocol to Eliminate Illicit Trade in Tobacco Products, “an international treaty with the objective of eliminating all forms of illicit trade in tobacco products through a package of measures to be taken by countries acting in cooperation with each other.”^234^ An example of such technology is SICPATRACE, which can track and trace tobacco, alcoholic beverages such as spirits and beer (also subject to excise duty), SSBs (in some cases an SSB tax plays a similar role to excise duty), and fuels. This solution applies fiscal markings on each product item, using security inks integrating multiple material-based based security elements that cannot be counterfeited, and a unique ID to enable traceability represented by a 2D barcode.

The use of this technology has had a dramatic effect for many countries. For example, Kenya saw a revenue increase of 45% in the first year of implementation, while Chile saw a 23% increase. Even in high-income countries, such as the US, the implementation of track and trace has been followed by a 37% reduction in seizures of illicit cigarettes. The World Bank and the International Monetary Fund^235,236^ have recognized the effectiveness of these solutions, which must be implemented by all 67 governments that have ratified the Protocol to Eliminate Illicit Trade in Tobacco Products. Other international suppliers of secure track and trace solutions include the UK-based De La Rue and Opsec, and US-based Authentix.

The tobacco industry has responded to this market need by offering their own tracing system, Codentify, which was conceived by the industry and then offered through third-party companies that contributed to its development. However, there are serious concerns about the effectiveness of controls originating from an industry that itself must be controlled (the Protocol clearly defines that obligations assigned to a Party shall not be performed by or delegated to the tobacco industry). Moreover, solutions promoted by the tobacco industry rely entirely on digital track and trace technology, without the use of material-security features, such as tax stamps, that protect and authenticate each duty-paid product. As such, controls can easily be circumvented by the industry, reducing the ability of government authorities to identify gaps and enforce compliance in the market.

There are also 30 countries (45%) that are signatories to the Protocol that do not yet have a track or trace system yet in place. Putting in place state of the art secure and independent track and trace systems in all countries with effective enforcement and increasing taxes is the best strategy to reduce smoking and illicit trade in tobacco. These have the added advantage of providing a reliable source of financing for the countries who are paying the health cost of tobacco consumption. Such secure track and trace systems also have a role in managing counterfeit pharmaceuticals.

While there is ample evidence of excise taxes reducing tobacco consumption, there is more limited, but generally robust, evidence that excise taxes reduce consumption of alcohol and sugar-sweetened beverages (SSBs). In countries with a high prevalence of heavy episodic drinking and low alcohol tax levels, increasing these taxes could generate substantial reductions in death and disability from a range of conditions, including liver disease and cancer, suicide, and gender-based violence. These taxes can also increase general government revenues.

### Increasing prices for fossil fuels and unhealthy foods

In section 2, we argued that one of the headwinds to progress in global health is climate change, and we discussed the conceivably large eventual consequences for human mortality. And just as tobacco taxation is a win-win-win fiscal policy lever—reducing illness and death, raising revenue, and being pro-poor—there is also a win-win-win fiscal policy lever for curbing climate change: the removal of subsidies on fossil fuel production and consumption. Removing such subsidies can slow global warming, reduce ambient air pollution, and improve government finance. Action against coal emissions is the highest priority, given that coal power plants are the largest single source of greenhouse gas emissions.^237^

At the UN Climate Change Conference in Glasgow (COP26) in 2021, nations adopted the Glasgow Climate Pact, which called on all countries to “phase-out … inefficient fossil fuel subsidies, while providing targeted support to the poorest and most vulnerable.”^238^ The Organization for Economic Cooperation and Development and the International Energy Agency estimated that ministries of finance around the world collectively provided $468 billion in 2019 on subsidies for fossil fuels, the bulk of which was on oil products.^239^

In GH2035, we noted that energy subsidies on coal, gasoline, and diesel “encourage excessive energy consumption and production of ambient particulate matter pollution and other pollutants that cause lower respiratory infections in children, and cancers, heart diseases, and chronic obstructive pulmonary disease in adults.” These subsidies also divert public resources away from spending that would be pro-poor, such as on health, education, and social protection. Indeed, many countries spend more public resources on energy subsidies than on health and education combined. Removing fossil fuel subsidies therefore remains an urgent priority for tackling air pollution, climate change, and their impacts on health. We recognize that ending such subsidies may not be politically popular–some countries, such as Chile and France, saw protests and other social unrest when fuel prices rose. However, the value of removing such subsidies is now widely accepted by health and finance ministries and many nations have successfully phased out explicit subsidies, including India, Morocco, Saudi Arabia, and Ukraine.

Obesity is a major determinant of premature adult mortality in many populations, and is likely to become so in many others by 2050 if current trends continue.^240^ The WHO defines overweight as having a body mass index (BMI) of 25-<30 kg/m^2^ and obesity as having a BMI ≥30 kg/m^2^; it estimates that in 2022, 2.5 billion people were overweight, of whom 809 million were obese.^241^ At the same time, underweight and dietary inadequacy remain important in South Asia and parts of Africa.^242^

To examine the relationship between overweight, obesity, and all-cause mortality, the Global BMI Mortality Collaboration conducted a meta-analysis of 239 prospective studies that had individual participant data on 10.6 million people across four continents.^243^ To reduce the possibility of confounding and reverse causality, they restricted the analysis to the 4 million never-smokers without chronic diseases at recruitment. In this group, every 5 kg/m^2^ increase in BMI above 25 kg/m^2^ was associated with 31% higher all-cause mortality and 42% higher cardiovascular mortality. Being obese was more dangerous for men than women—the excess risk of premature death is about three times as big for men with obesity than for women with obesity. Nevertheless, in nearly all countries and at nearly all ages, the prevalence of obesity is higher in women than men.

There is an increasing body of research showing that the relationship between BMI and mortality may be different in different populations. For example, in their study of over 1.1 million people recruited in 19 cohorts in Asia, Zheng and colleagues found an excess risk of death associated with a high BMI in East Asia but not in India or Bangladesh.^244^ A prospective study of 150,000 people in Mexico found that “general, and particularly abdominal, adiposity were strongly associated with mortality.”^240^

Likewise, there is strong evidence that dietary practice affects mortality rates. Walter Willett and his team at Harvard University recently assessed the consequences of adherence to the EAT-Lancet Commission’s 2019 dietary recommendations.^245,246^ Using data from a 34-year cohort study involving around 200,000 health professionals, they found all-cause mortality to be 23% lower in the quintile most closely following a “planetary health diet” (denoted as the highest quintile) compared to the bottom quintile.

Obesity can emerge quickly in a population, over just 30–50 years, as seen for example in Pacific island nations like Nauru and the Cook Islands.^247^ The WHO recommends that member states use targeted fiscal policy to reduce obesity—in particular, taxation of SSBs and energy-dense foods and subsidizing foods that contribute to a healthier diet. Taxation of SSBs, which has been implemented in over 117 countries and territories,^248^ leads to “substantial consistent declines in SSB purchases,”^249^ though the impact on obesity remains unclear.

Taxing energy-dense food has not been widely adopted, though there are some country successes, including in Denmark, Ethiopia, Hungary, Mexico, and Tonga.^250^ Colombia, where 56% of the population is overweight, recently became one of the first countries to introduce a “junk food tax,” i.e. a tax on foods high in salt and saturated fat, to reduce obesity.^251^ Some countries have invested revenues from food and SSB taxation to pro-poor public spending—for example, Malaysia uses revenues from SSB taxes to provide free, healthy breakfasts for primary school children.^252^

Ultra-processed foods (UPFs)—industrially manufactured, prepackaged, ready-to-eat products—have also become a target for taxation (Colombia’s junk food tax includes taxation of UPFs). A recent umbrella review of epidemiological meta-analyses suggested an association of UPFs with obesity,^253^ and ecological studies in sub-Saharan Africa suggest that taxes on UPFs could curb consumption and reduce obesity.^254^

More generally, although the possibility of population reversal in obesity levels is plausible, there are still no examples of even modest success. Indeed, the relation of diet and dietary interventions to obesity and disease is constantly being reassessed and it may be premature to conclude that effect sizes are as large as widely believed. Health systems will thus need to cope with the consequences of high prevalence rates of obesity. Hormonal peptide inhibitors are an important breakthrough in treating obesity,^255^ but practical evidence of population effects over time can only be established when prices fall sufficiently to allow widespread uptake.

Finally, just as removing fossil fuel subsidies would be a win-win-win, ending subsidies on meat and dairy could have multiple benefits—reducing greenhouse gases, curbing the destruction of biodiversity, and assisting the transition away from diets heavy in meat towards plant-based diets. Meat and dairy production, which covers an area as large as the entirety of the Americas (38 million km^2^ of land),^256^ is the primary driver of biodiversity destruction and accounts for about 15% of greenhouse gas emissions.^257^ The IMF notes that in many countries, “large amounts of taxpayers’ money are spent on subsidies that encourage otherwise unprofitable, unsustainable meat and dairy production predicated on the systematic inhumane treatment of farmed animals.” ^257^ Reducing such subsidies or redirecting them towards sustainable farms that produce plant-based protein for human consumption would have favorable health and fiscal consequences. In Poland, ending subsidies in the late 1980s for butter and substitution of vegetable fats from expanded market access was associated with a marked reduction in vascular disease.^258^

## Section 9. International collective action for health

WDR93 pointed to the particular importance of using development assistance for health (DAH) for financing global health research and development (R&D). But it failed to make the more general case that as low-income and middle-income countries grow economically, international resources should, over time, move away from routine support of country health system strengthening and disease control, which are national responsibilities, towards international collective action for health, including R&D, pandemic preparedness and response (PPR), and tackling AMR. GH2035 argued strongly for this transition, an argument that CIH 2.0 developed further. CIH authors have undertaken work that has led to a better empirical knowledge of what fraction of DAH goes to global goods, what the sources of finance are, including from non-traditional sources, and on what those resources are spent.^259,260^

### Investments in international collective action for health

In GH2035, we pointed to the underfunding of the “global functions” of health, which address health challenges that go beyond the boundaries of individual nation states. We categorized global functions into three types: (i) provision of global public goods (GPGs), such as product development for neglected diseases; (ii) management of cross-border externalities, such as PPR; and (iii) fostering leadership and stewardship, such as convening for consensus building. Funding for global functions reaps transnational health benefits, regionally or globally. In contrast, funding for what the CIH calls country-specific functions refers to funding given to an individual country to support health system strengthening and disease control activities, such as supporting the reduction of child and maternal mortality, which will benefit that country alone. Jamison and colleagues defined country-specific functions as activities that tackle “time limited problems within individual countries that justify international collective action because of highly constrained national capacity.”^261^ These problems require solidarity from richer to poorer countries. It is important to be clear that the CIH advocates that expenditures on GPGs need to pass reasonable benefit-cost tests—the same as for any other health development assistance expenditure.^262^ Not all GPGs will pass that test. However, studies show that investments in global health research and development promise substantial public health and economic returns and that these returns would be even larger if the full efficiency potential in the global ecosystem were leveraged (panel 13). In addition, COVID-19 clearly showed that investments in pandemic preparedness pay off, while, at the same time, the costs of inaction are massive.

#### Panel 13: Investments in global health innovations pay off

Studies show that investments in global health product development have substantial returns. For example, a study by Jamison et al found that about 80% of the decline in the under-5 mortality rate from 1970–2000 across 95 low- and middle income countries can be explained by the diffusion of new health technologies .^49^ Policy Cures Research found that 183 new neglected diseases products have been approved by a regulatory agency or prequalified by WHO since 1999, which already have saved over 8 million lives.^263^ With respect to economic benefits, a study by Schäferhoff et al estimated that the returns on investing in late-stage clinical trials and manufacturing in three middle-income countries (India, Kenya, and South Africa) would be as high as about $21-$67 per dollar invested.^264^

New cutting-edge technologies are on the horizon. Ogbuoji et al found that there are currently 1,498 candidate medicines, vaccines, and diagnostics in the product development pipeline for neglected diseases, emerging infectious diseases, and maternal health conditions.^51^ Their study estimated that investing in research and development (R&D) to advance these current candidates in the pipeline would yield 453 product launches from 2023 to 2044 under a conservative base-case scenario. Many of these products target the I-8 conditions (table 18; appendix table 9.1 on page 88 of the appendix and appendix table 9.2 on page 92 of the appendix). With better coordination, an even larger number of products could be launched. The incremental cost, over and above current R&D spending, would be $1.4-$7 billion annually depending on the complexity of the product candidates being launched. Substantial cost savings could be achieved–about $9 billion over the 2023–2044 period–if ecosystem efficiencies, such as AI and smarter clinical trial designs, were to be implemented.

However, the development of these new tools requires additional investments in product development, especially in light of the rising costs of late stage clinical trials and the high trial attrition rates. The decline in funding for neglected disease basic research and product development is therefore a concern. There needs to be sufficient investment to deliver the potential new products from the current pipeline.

In addition, there are multiple and potentially game-changing candidates in the pipeline that address the NCD-7, including for cardiovascular diseases, diabetes, and mental health (table 18; appendix table 9.1 on page 88 of the appendix and appendix table 9.2 on page 93 of the appendix).

Sustained efforts will be required to ensure pricing policies and delivery mechanisms that enable these advances to serve the needs of people in highly resource constrained settings.

To close the funding gap for global functions, GH2035 recommended that a greater proportion of annual DAH should be directed towards these functions. However, available DAH data did not provide evidence on the extent to which resources were targeted at global functions, so in 2015 CIH authors introduced a new method to track aid by function.^259^ The method allowed us to estimate the proportion of DAH directed to the three categories of global functions and to country-specific functions. We also introduced a broader concept of health aid, called “DAH+,” to capture additional public spending on product development for neglected diseases from agencies such as the National Institutes of Health that are usually excluded in studies that track DAH.

**Figure 20:**
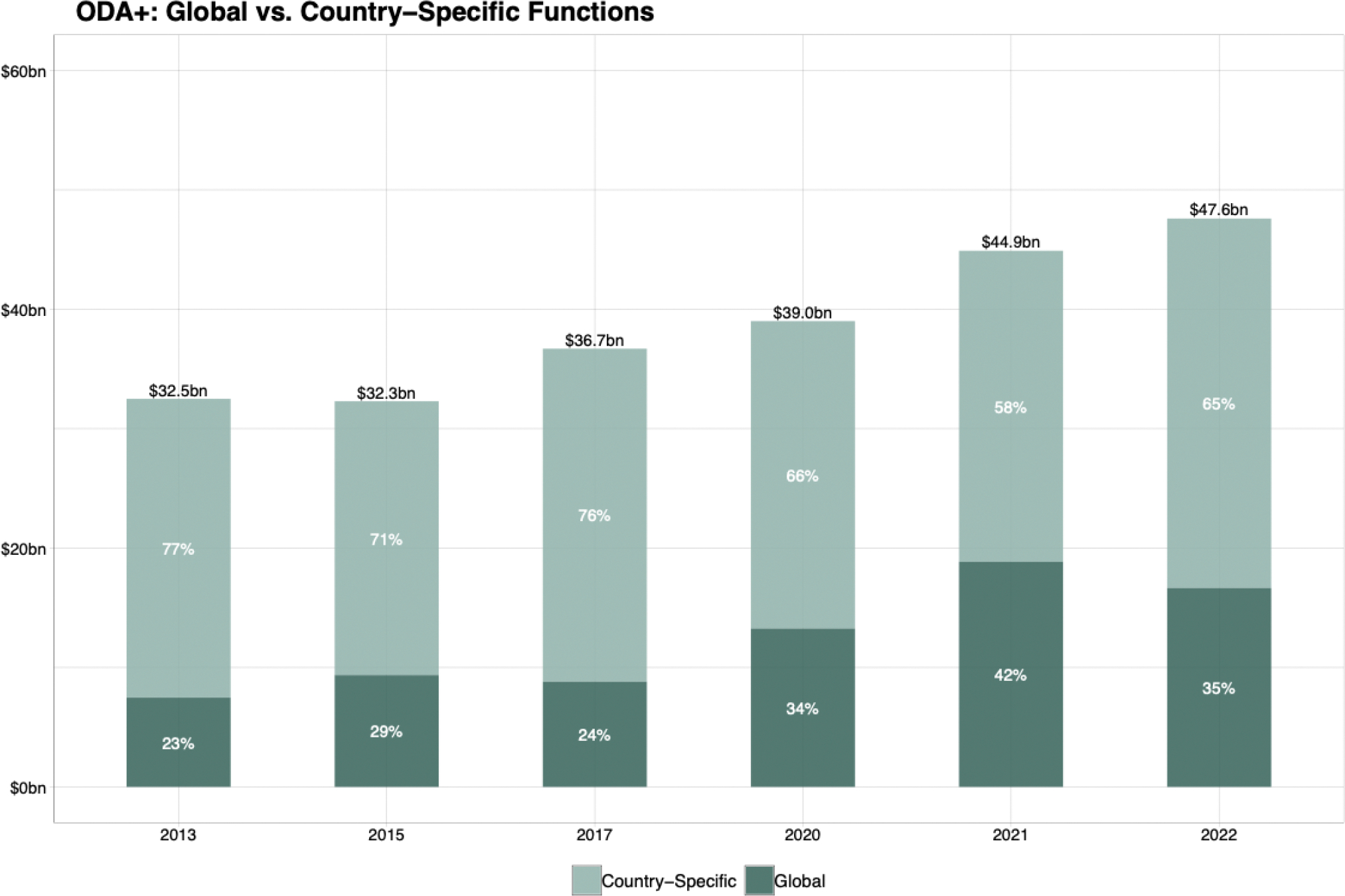
ODA+: Evolution of DAH+ disbursements, 2013–2022 Notes: Gross disbursements. Constant 2021 prices (USD billions). Sources: The CIH uses the term DAH+ to refer to official development assistance (ODA) for health and private (philanthropic) development finance to health as defined by the Organisation for Economic Cooperation and Development (OECD)’s Development Assistance Committee (DAC). DAH+ also includes donor funding for neglected disease product development. Data for our DAH+ analysis comes from two sources: the OECD DAC’s Creditor Reporting System (CRS) database, and the Global Funding for Innovation for Neglected Diseases (G-FINDER) database. See also: reference 50 ^50^

Our 2015 study found that global functions accounted for 23% of the $32.5 billion in DAH+ disbursements in 2013 (in 2021 $), while 77% was allocated towards country-specific activities. A follow-up study by CIH authors confirmed that donors are prone to “cycles of panic and neglect.”^260^ In response to the 2014–2016 Ebola epidemic in West Africa, during the “panic” phase the share of funding for global functions grew to 29% in 2015, driven by a reactive increase in outbreak response funding (figure 20). This was followed by a “neglect” phase—donors did not sustain their preparedness funding after the outbreak, and the share of funding for global functions dropped to 24% in 2017.

Our new analysis for CIH 3.0 has extended these assessments well into the pandemic period.^265^ DAH+ disbursements reached $44.9 billion in 2021 and $47.6 billion in 2022, its highest ever level (figure 20). Despite justified criticism of the behaviour of high-income countries during the pandemic, especially their hoarding of COVID-19 vaccine doses^170,196^, the pandemic led to a substantial increase in DAH+. In addition, the share of DAH+ targeted at global functions grew from about a quarter before the pandemic to over a third during it. The pandemic response clearly drove this increase, but funding for other global functions also contributed to this upward trend. Funding for the control of cross-border disease movement, which includes funding for regional programs and polio eradication, grew compared to previous years (table 17).

However, there are also some concerning trends. First, the share of funding by donor governments channelled through multilateral agencies increased from 23% in 2020 to 30% in 2021, while the share of funding that was directly provided to recipient governments dropped from 38% to 33%. At the same time, DAH+ disbursements to low-income countries remained flat in 2021 compared to 2020 levels, which indicates that the additional funding made available by donors in 2021 did not reach low-income countries.^266^

Second, funding for basic research and product development for neglected diseases fell from $3.8 billion in 2021 to $3.3 billion in 2022. Many of the I-8 conditions are neglected diseases: bacterial pneumonia, diarrheal diseases, HIV/AIDS, malaria, and tuberculosis. This fall comes at a time when there is a pressing need to increase investment in such product development—in order to achieve the “30 by 2035” and “50 by 50” goals described in section 3—and to leverage new approaches to reduce development costs. Given its potential to drive major efficiencies, there is huge interest in applying artificial intelligence (AI) to global health product development, including for neglected diseases and AMR (panel 14). AI has been applied across the whole product development spectrum, including new target identification, drug candidate selection, protein structure prediction, molecular compound design and optimization, and clinical trial design, conduct, and analysis. AI tools can accelerate research, reduce costs, and improve discovery through accelerated and more comprehensive screening, resulting in a greater number of quality candidates to be tested in clinical research.^50^

**Figure 21:**
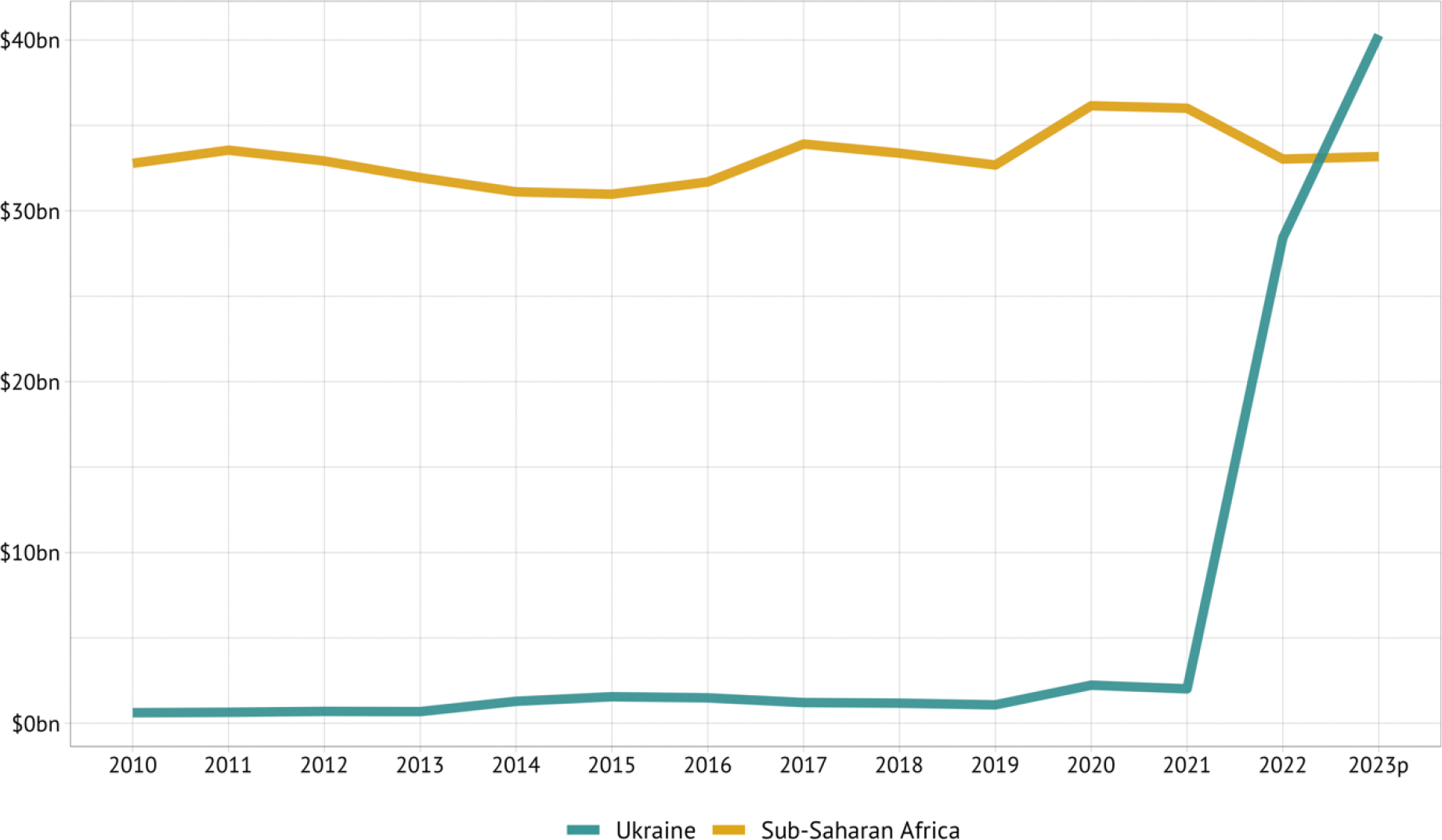
Total ODA disbursements to Ukraine and Sub-Saharan Africa Notes: Disbursements. Constant price in 2022 (USD millions). p= Preliminary figures. Source from reference 267 ^267^

Third, there is pressure on resources within the international system—the health sector must compete with other important priorities. Figure 21 illustrates responses to the Ukraine war alone.

#### Panel 14: Developing antibiotics in the age of AI

AMR continues to be a major threat. In 2019, nearly 1.3 million people died from resistant infections.^268^ By 2050, the death toll of AMR could reach 10 million annually, potentially surpassing cancer as a leading cause of death.^269^ For most of the 20th century, as resistance to specific antibiotics grew, physicians could rely on the discovery and development of new antibiotics to replace them. Now, however, challenging market conditions and limited investment in innovation have led to a decades-long discovery drought and a dwindling antibiotics pipeline.

Recent antibiotic development has focused on making small modifications to existing classes. Though these may create marginal improvements such as increased efficacy or decreased toxicity, in the short term, due to the structural similarities, these antibiotics are ultimately just as susceptible to resistance. Today, some form of resistance has developed against every major class of antibiotics. The AMR crisis will not be solved unless we can quickly develop novel treatments against superbugs and ensure appropriate use. Here, the right use of AI could prove transformational.

AI is uniquely useful for antibiotic discovery for three reasons: (1) it offers unprecedented opportunities to search across vast and unknown chemical spaces for truly novel compounds; (2) the efficacy of AI-generated compounds can be quickly tested in a petri dish, creating a rapid and efficient feedback loop that enables ever-more targeted drugs to be developed; and (3) the speed and potential cost efficiencies of AI-based discovery can help overcome some of the funding challenges stifling early antibiotic development.

For example, Phare Bio is a social venture harnessing the power of AI to tackle the antibiotic pipeline crisis. In collaboration with Professor Jim Collins’ lab at Massachusetts Institute of Technology, Phare Bio’s mission is to use AI and a translational feedback loop to rapidly discover, design, and develop new antibiotic classes against the world’s most urgent bacterial threats, as designated by the WHO and CDC. The Collins lab’s first breakthrough discovery was published in *Cell* in 2020, detailing how the team used machine learning to identify a broad-spectrum antibiotic with potent activity across Gram-negative bacterial pathogens.^270^ Since then, the team has made significant enhancements to the AI platform, including the incorporation of generative AI that is moving beyond identifying antibiotic hits from existing compound libraries to *de novo* design of entirely new compounds, and “explainable” AI that elucidates how the model is learning and making its predictions.^271^ The use of these methods has produced new and promising candidates, including the discovery of a new structural class of compounds active against Gram-positive bacteria such as MRSA.^272^

Recognizing a need to move these discoveries out of academia and through the “valley of death” (the phases of development characterized by high failure rates and lack of financial investment), Phare Bio uses philanthropic and grant funding to build its pipeline of novel, de-risked preclinical candidates, and leverages commercial partnerships for more costly clinical development. With this approach, Phare Bio can file intellectual property for their novel candidates and leverage it for out-licensing agreements with subsequent development partners – further bolstering the financial sustainability of the organization. This blended-finance model addresses the trade-off between profits for privately held intellectual property in these compounds, having prices that can ultimately reach the patients in greatest need, and the philanthropic finance required. The challenge will be for the philanthropists to ensure that their investments do, indeed, result in low prices and widely accessible products rather than high profits.

Aid for health faces an uncertain future. Even after the worst pandemic in a century, donors have lost their appetite to support PPR, a new phase of neglect. This neglect is exemplified by the Pandemic Fund’s struggle to mobilize funding, which seems unrelated to potential shortcomings in its design.^273^ As Michaud and Kates^274^ say, the fund has received “limited donor funding to date and [faces] uncertain future support,” though it could potentially mobilize finance in its upcoming replenishment cycle. Recent international crises have led to major shifts in the global aid landscape and major donors have announced cuts to their aid budgets, which will likely also affect the health sector (panel 15).

#### Panel 15: Official development assistance under pressure

The period from 2020–2023 was marked by major global shocks, including the COVID-19 pandemic and its global economic impacts, rising geopolitical tensions, new and intensifying armed conflicts, and humanitarian crises. These shocks led to substantial shifts in official development assistance (ODA).^265^

In 2022, total ODA disbursements grew by more than a fifth (22%) to a record high of US$277 billion. However, the growth in ODA largely resulted from two factors – support to Ukraine and funding for hosting refugees in donor countries. If these two factors are deducted, ODA increased by only 2.8%. ODA for Ukraine increased sharply, from US$2 billion in 2021 to US$29 billion in 2022, making Ukraine the largest ever recipient of ODA. Preliminary 2023 data indicate that aid to Ukraine grew further to US$40 billion. As such, Ukraine received more aid in 2023 than Sub-Saharan Africa as a region (Figure 9.2). Increases in vitally important humanitarian aid and refugee support went predominantly to Ukraine. The support to Ukraine also contributed to the highest ever level of humanitarian aid – US$37 billion in 2022. The costs for hosting refugees in donor countries have increased substantially since 2020. The 29 member countries of the OECD Development Assistance Committee used 18% of their bilateral ODA for hosting refugees in 2022, compared to 7% in 2020.

The share of ODA for the least developed and other low-income countries dropped from 36% in 2020 to 25% in 2022, leading to an absolute reduction in ODA for least developed and other low-income countries in 2020–2022. The poorest countries are still suffering from the adverse impact of the pandemic: the negative economic effects of the COVID-19 pandemic resulted in the largest surge in extreme poverty globally in decades. A recent World Bank analysis shows that middle-income countries have recovered from the economic setback, but poverty levels in the poorest countries are still worse than before the pandemic.^275^

In addition to these major reallocations of aid, many large aid donors, such as France and Germany, have recently announced cuts to their ODA budgets, threatening overall global levels of ODA.^276–278^

Although very recent data are unavailable, longer term policies and trends support the encouraging observation in GH2035 that China’s rising development assistance may run counter to these otherwise generally adverse trends in DAH.^279,280^ These adverse DAH trends are also to some extent being counterbalanced by the rise of regional agencies. The COVID-19 pandemic fuelled unprecedented regional action, such as the launch of the African Union’s Africa Vaccine Acquisition Trust and the Asian Development Bank’s Asia Pacific Vaccine Access Facility. During COVID-19, these two initiatives complemented COVAX, which helped to achieve the fastest vaccine rollout in history^281^ and provided 74% of all COVID-19 vaccine doses to low-income countries. COVAX was hindered by pharmaceutical companies and high-income nations making bilateral deals^196^, pushing COVAX to the back of the queue, which shows the importance of strong sovereign national and regional buying power.

Adeyi and Nonvignon^282^ and Nonvignon et al^283^ have called for the global health architecture to become more decentralized. Some aspects of their calls were echoed by Anders Nordström, Sweden’s former ambassador for global health.^284^ Using DAH to support regional structures, such as the African Centres for Disease Control or regional public development banks, including for improving access to medicines and vaccines via demand creation, pooled procurement, and delivery, is very much in line with CIH 3.0’s support for global functions.

Furthermore, non-OECD governments are gaining an increasingly important role in development finance. Unlike OECD donors, China does not report on its development finance, although a recent analysis concluded that China uses multilateral processes to inform its DAH priorities.^285^ AidData’s Global Chinese Official Finance dataset estimates that the Chinese government funded more than 13,000 development projects worth $843 billion across 165 countries between 2000 and 2017^286^, and China was a major provider of effective vaccines during the COVID-19 pandemic. About 1% of its pre-pandemic funding was for health projects. China has become an important provider of aid to African and increasingly to Asian countries (though many of these projects are funded through some kind of lending or commercial arrangements), and its influence in these regions will likely continue to grow.

### Investments in priority infections and maternal health conditions

GH2050 continues to argue for the importance of investing in global functions, particularly in PPR and research and product development for the 15 priority conditions we identified. These investments should include support for critically important GPGs that the WHO provides, such as setting global norms and standards, assessing health trends, and developing regulations and conventions.^287^ The WHO Council on the Economics of Health for All^197^ highlighted the WHO’s role in the overarching governance of the multilateral global health system—such governance is another important GPG. A recent investment case points to the high benefits relative to costs of the world’s relatively modest potential expenditures on WHO.^197^

**Figure 22:**
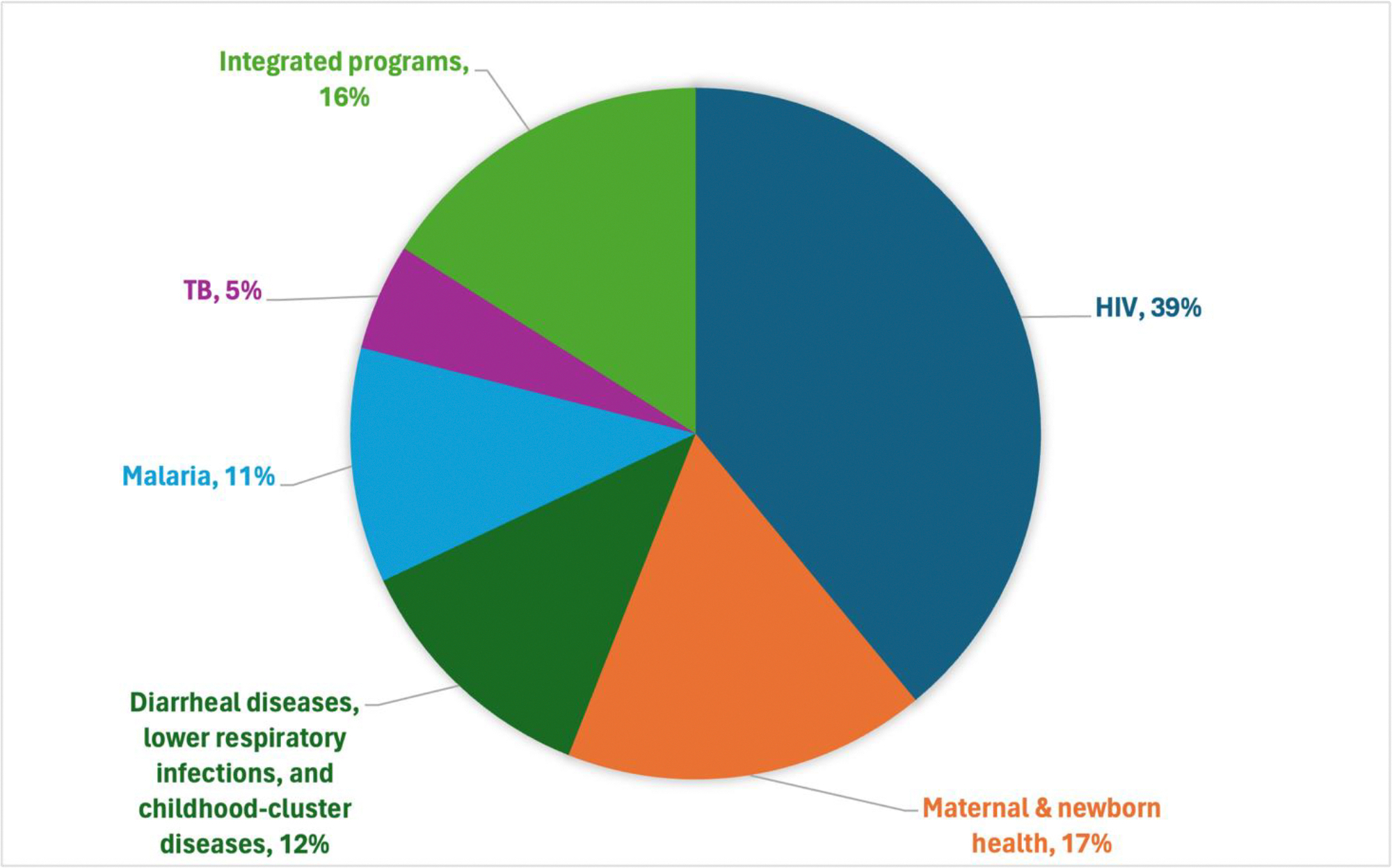
Country-specific funding for the I-8 conditions Notes: Gross disbursements. Constant 2021 prices (USD) Source from reference 50 ^50^

But what, then, should the role of DAH+ be in direct country support, i.e., in the direct support of disease control and HSS in highly resource constrained countries? We make three broad arguments on country-specific aid, two relating to stronger prioritization and one on more efficient use of DAH+. First, funding for country-specific functions should be focused on the priority infections and maternal health conditions (the I-8) to reach “30 by 2035.” Our analysis finds that in 2022, $31.0 billion was targeted at country-specific functions, of which about three-quarters (76% or $23.7 billion) addressed these priority conditions. The remaining share (24%) was mostly for broader health systems support ($7.2 billion), while only a small fraction was for NCDs ($0.2 billion). While, overall, country-specific aid is strongly targeted at the I-8, the funding is distributed unevenly across the eight priority conditions. Between 2020 and 2022, 39% of the I-8 funding was for HIV, with more than half of the HIV funding (51%) targeting middle-income countries. Low-income countries only received 23% of the HIV funding (the remaining 26% was not allocable by income group). Funding for maternal and newborn health accounted for 17%, while malaria and tuberculosis received 11% and 5%, respectively. The remainder was directed to the remaining three priority conditions—diarrhea, childhood-cluster diseases, and lower respiratory infections (12%)—and to integrated service delivery (16%) (figure 22). Our analysis shows that funding for several I-8 areas—notably tuberculosis—is small, while HIV alone accounts for a substantial share of country-specific I-8 funding. Much of the HIV funding is driven by the need to maintain people on antiretroviral drugs. Going forward, major donors to HIV programs, including PEPFAR, have recognized that countries they support—particularly middle-income countries—should finance these programs domestically, as a pathway to sustainability. However, an even greater volume of resources for the priority infections and maternal health conditions will likely be needed to achieve “30 by 2035” in low-income countries.

As highlighted in our previous DAH+ analyses, while the benefits of supporting global functions are transnational, these investments can be made at different levels of the global health system.^260,287^ Examples include funding to individual low- and middle-income countries for pandemic preparedness and response, polio eradication, and responses to AMR that at the same time ensure access to effective treatment.^288^ It is entirely appropriate for DAH+ to also support middle-income countries in these activities, although the instruments used, such as blended financing, should also incentivize (not substitute for) domestic allocations to these areas.

Second, funding for country-specific disease control and HSS should focus on the least-developed countries. Between 2020–2022, 28% of country-specific funding was only channelled to least-developed countries and other low-income countries, while lower-middle-income countries and upper-middle-income countries accounted for 41% and 9%, respectively (the remainder of 22% was not allocable by income group). In the same period (2020–2022), 29% of the I-8 funding was directed to least-developed countries and other low-income countries, 39% to lower-middle-income countries and 8% to upper-middle-income countries (24% were not allocable by income group). These data indicate that funding for the priority infections and maternal health conditions is not well targeted, neglecting countries with the lowest resources, and suggests a potential to shift more of the available funding to resource constrained settings. About half of the I-8 funding went to middle-income countries with adequate potential for domestic public finance of their health system. In addition, there is substantial evidence that donor funding can lead to aid substitution, also known as fungibility. A natural response of finance ministries to country-specific health aid is to reduce domestic public finance for health.

Third, implementation efforts should focus on ensuring affordable drug availability to address the priority infections and maternal health conditions at reasonable costs. One of the best ways that donors can support the “30 by 2035” goal is by bringing down drug prices through market shaping, i.e., by pooling demand and purchasing for multiple countries, and by subsidizing drug prices.^139^ Prices for major childhood vaccines have fallen through Gavi’s market shaping interventions and through UNICEF’s pooled procurement. The Global Fund plays an important role in shaping global markets for medicines and technologies that prevent, diagnose, and treat HIV, tuberculosis, and malaria. About half of the Global Fund’s annual investments—$2 billion per year—are used to procure key medicines and health products, of which about $1.5 billion are purchased through the Global Fund’s pooled procurement mechanism.^289,290^ Through the market-shaping activities of the Global Fund and its partners, such as PEPFAR and the Global Drug Facility, prices for first-line HIV treatment recently dropped to under $45 per person per year, compared to $300 in 2014, a major achievement. In 2023, multidrug-resistant tuberculosis drug prices also fell by more than half (55%) but the price for these drugs remain significantly higher than the price of first-line HIV treatment.^291,292^ A key rationale for donors to fully resource Gavi and the Global Fund at their next replenishments is to ensure that their market-shaping power for priority infections and maternal health conditions can be fully leveraged.

We also favour the use of DAH to support what we are calling the “Arrow mechanism” (see section 6), which was initially implemented through the AMFm (panel 9). The Arrow mechanism goes beyond subsidies and pooled procurement in two significant ways: high volume of supply to ensure widespread availability at affordable prices and reliance on domestic supply chains—public and private. As discussed in section 6, the AMFm subsidized artemisinin-based combination therapies at the factory gate to undercut prices for monotherapies to avoid them becoming resistant to the malaria parasite; it quickly and effectively helped to drive monotherapies from the market in pilot countries.^167^ Due to the continued need for access to affordable medicines, including for NCDs, the Arrow mechanism is more important today than ever. The subsidy can be funded in a variety of ways, not just by donors. It could be placed at the regional or even country level, paid by domestic resources from low- and middle-income countries, and with payments going directly to manufacturers, including potentially to development of domestic or regional manufacturing capacity.

There is also a need to address the growing debt burden of low- and middle-income countries—a challenge central to Brazil’s G20 presidency (the final summit under its presidency takes place in Rio on November 18–19, 2024). Building on the experience of debt swaps in the environmental and climate sectors,^293^ the Global Fund introduced the Debt-to-Health (D2H) initiative in 2007. Under D2H, a creditor country waives its rights to outstanding debt repayments on the condition that the debtor country commits this repayment to domestic health programs.^294,295^ To date, twelve transactions have generated $226 million for ten debtor countries, and $373 million in debt cancelled through the D2H swaps program. The D2H has proven its potential to create fiscal space for increased domestic health investments, though these are small amounts and, quantitatively, this potential remains to be realized. Going forward, debt swaps provide an important opportunity for countries to reduce their debts and strengthen their domestic health investment at the same time.

### Funding for the NCD-7

About $200–300 million per year in donor funding is allocated towards NCDs (table 17). The global health architecture also largely lacks NCD market shaping mechanisms. One exception is the Pan American Health Organization Strategic Fund, a pooled procurement mechanism. Countries in the Americas have been able to use the fund to purchase medicines for NCD products, including for cardiovascular diseases, cancer, and diabetes. Another example is the partnership between the non-profit organization Resolve to Save Lives, multilateral agencies such as the WHO, and country governments to expand access to hypertension medicines, including in India and Latin America.^296^ Due to the growing burden of NCDs, establishing market shaping mechanisms for NCD products, especially for the NCDI-7, will become increasingly important for low- and middle-income countries. It will be crucial to design these mechanisms in a way that allows middle-income countries to benefit from them.

In addition, we argued in GH2035 that more investments are needed for population, policy, and implementation research (PPIR), which involves both the emerging field of implementation science and health policy and systems research. The goal of PPIR is to identify best practices and to facilitate their diffusion across countries; however, individual governments have insufficiently strong incentives to invest in such knowledge generating activities that have value beyond their borders. Given the shifts in the global burden of disease, PPIR will be particularly important for NCDs. Global health donors could fund PPIR for NCDs to identify and facilitate transfer of best practices in addressing the NCD-7.

Both market shaping to reduce drug prices and PPIR provide ways that international resources can directly improve outcomes for the poor in middle-income countries.

### Funding for pandemic prevention, preparedness, and response

As discussed in section 7, a new study prepared as a background paper for GH2050 suggests that there is almost a one in four chance of a pandemic of at least COVID-like severity in the coming decade.^47^ It estimates that the average annualized mortality from future global respiratory pandemics is 2.5 million deaths—1.6 million from influenza and 0.9 million from coronaviruses—which is about the same as the annual number of deaths from HIV, tuberculosis, and malaria combined. Despite this risk, the world remains largely unprepared and is massively underinvesting in preparedness, including in pandemic vaccine development, both a pan-coronavirus vaccine protective against multiple strains ^297^ and a universal influenza vaccine.

Currently, each strain of influenza virus now requires its own vaccine—a new influenza vaccine is introduced each year against the currently circulating seasonal influenza—and so a vaccine against an emergent pandemic influenza strain could take a year or more to get into widespread use. A universal influenza vaccine would be of enormous value for both pandemic and seasonal influenza; multiple, but modest, efforts are now underway to develop such a vaccine. CIH calculations suggest great potential value in a major acceleration of development efforts for such a vaccine. Existing, widely used vaccines against measles, polio, and tuberculosis have shown potential effectiveness against both influenza viruses and coronaviruses.^298,299^ A more complete and up-to-date understanding of this scientific potential would be valuable, including appropriate trials.

Vaccine related investments of only several billion dollars per year promise expected returns in health security of 10 or more times the investment. We agree with Adam Tooze that when it comes to pandemic vaccine R&D, “not only is the cost-benefit ratio unbeatable, but not to undertake this spending is to court disaster.”^300^ Yet this spending is yet to fully materialize. The Coalition for Epidemic Preparedness Innovations (CEPI), for example, asked funders for $3.5 billion in 2021 to prepare for known pandemic threats but was only able to mobilize $2 billion by the end of 2022.^195^ It will also be important to develop the platforms for the next pandemic. Recent advances in AI technology may make it possible to quickly and effectively model potential viral vaccine and drug targets, which is important for pandemic preparedness. CEPI intends to store AI-derived antigen designs in a “vaccine library” to accelerate development of vaccine candidates in the event of an outbreak of a new pathogenic threat. CEPI has funded research to map potential antigenic targets for 10 priority virus families with epidemic or pandemic potential.^301^

Surveillance, early warning, and prevention capacities are important, globally, but there will be little to no justification for the finance ministers of lower income countries to allocate domestic resources to developing these capacities. International resources are required.

DAH will play a critical role in supporting “day zero financing” of the pandemic response.^302^ Such financing refers to pre-committed funding that is made available immediately when the next pandemic hits to support development and equitable deployment of medical countermeasures. In December 2023, Gavi, the Vaccine Alliance’s Board approved a US$500 million investment in a First Response Fund as part of a broader Day Zero Financing Facility.^303^

Overall, a new approach to collective financing of PPR is needed. One such approach is Global Public Investment,^304^ in which all countries contribute through a fair share mechanism, making commitments over time that are more sustainable, equitable, and predictable.

### Manufacturing capacity

In addition to large-scale investments in R&D, global manufacturing capacity needs to be strengthened—the lack of vaccine production capacity was a major barrier during the COVID-19 pandemic. Governments of low- and middle-income countries need to be able to manufacture basic drugs and other material inputs without barriers placed in the way by the currently dominant global manufacturers and high-income country governments. Since GH2035 was published, we have emphasized the importance of building regional manufacturing hubs for vaccines, therapeutics, and diagnostics. The pandemic has led to several new manufacturing initiatives, an important dynamic. However, these initiatives have a strong focus on mRNA vaccine production. While they are important and should continue, diversified manufacturing is needed to enable production of non-mRNA vaccines and vaccines against multiple diseases in low- and middle-income countries. To create sustainable markets, funders need to support local or regional manufacturing when there is reasonable expectation of success. Intrinsic economies of scale and demands on technical and managerial resources require long term commitments to succeed. The increasing unreliability of global supply chains makes investment in national and regional capacity potentially worthwhile even when narrow economic considerations might suggest otherwise. There are encouraging signs on this front—for example, Gavi has committed $1 billion to support African vaccine manufacturing through a new African Vaccine Manufacturing Accelerator.^303^ Adeyi et al have pointed to the significance of the African Union’s goal that 60% of Africa’s vaccine needs are produced on the continent by 2040.^282^ At least important as capacity for vaccines is capacity for priority drugs, diagnostics, and equipment.

It will be important to monitor the extent to which these new initiatives fundamentally transfer technology to emerging firms in low- and middle-income countries beyond fill-and-finish (i.e., beyond just filling vials with vaccine and packaging them for distribution). There are a number of criteria that could be used to assess the strategic and operational value proposition of such initiatives across vaccines, drugs, and diagnostics. For example, one criterion is whether these initiatives fit with the country and regional strategies of low- and middle-income countries. A second is the importance of focusing on drugs and commodities to address the priority 15 conditions. A third concerns the time horizons by which diverse low- and middle-income countries are truly able to develop manufacturing free of IP constraints on products or processes. IP may, for the 15 priority conditions, prove less important than growing a technical workforce.

A high value investment for DAH is to help establish stronger clinical trial networks in low- and middle-income countries that can be used in conjunction with manufacturing capacity. The HIV Prevention Trials Network is a model for the value of such networks. When the COVID-19 pandemic hit, it rapidly pivoted to conducting COVID-19 vaccine trials, and during the 2022 MPOX outbreak it pivoted again to doing MPOX vaccine trials.

### The need for new global financing through strengthening the international system

The Indian G20 Presidency commissioned a report on the future of multilateral development banks (MDBs) from an Independent Expert Group (IEG), co-convened by Nand Kishore Singh, President of the Institute of Economic Growth, and Lawrence Summers. The IEG report concluded that radically reformed and strengthened MDBs are essential to address the global challenges in today’s world; it made three recommendations to leverage the potential of MDBs.^305^ First, the IEG suggested that MDBs should adopt a triple agenda of eliminating extreme poverty, boosting shared prosperity, and contributing to GPGs. Second, the group recommended tripling MDB lending levels by 2030. Overall, the IEG estimated that $500 billion in additional annual official external financing would be needed by 2030. MDBs should provide an incremental $260 billion of the additional annual official financing (of which $160 billion would be concessional lending). Finally, the IEG called for the creation of a Global Challenges Funding mechanism, which would have flexible and innovative arrangements for engaging with investors willing to support elements of the agenda for meeting global challenges.

While the IEG has advanced a constructive agenda, its co-chairs have placed these aspirations into the context of what they view as drastic failure of the global system in 2023. This failure, they argued, resulted in major reverse resource transfers out of the global south, as we noted earlier in this report.^27^ A clear implication is that while low- and middle-income countries and regional institutions might hope for multilateral reform, they would be unwise to plan on it.

That said, regional MDBs significantly stepped up their health financing during the COVID-19 pandemic, and should be used to provide additional concessional and non-concessional funding for health. Their reach could even be further enhanced by expanding the health investments of all public development banks (PDBs). There are at least 330 PDBs that together provide more than $2.3 trillion per year of funding for public investments in developing countries.^306^ During the COVID-19 pandemic, Afreximbank in Africa provided financing for vaccines through the African Vaccine Acquisition Trust mechanism. Corporacion Andina de Fomento funded vaccines for Latin American countries.

We also agree that MDBs, especially the World Bank institutions, should embrace a GPGs agenda. Building on the Evolution Roadmap, the Bank’s Board of Executive Directors recently approved a new Framework for Financial Incentives to promote investments in projects that generate positive cross-border externalities.^307^ Further reform of the financial architecture for health will be required, including to catalyze more domestic finance, a key recommendation of the Future of Global Health Initiatives.^308^ This initiative was a time-bound, multi-stakeholder process, co-chaired by the Kenyan and Norwegian governments, which aimed to accelerate shifts in the global health ecosystem to support country-led trajectories towards UHC. Adeyi and Nonvignon have argued that the initiative should have recommended an even more decisive shift from the status quo.^282^ Important as domestic financial mobilization is, a key test of proposed MDB reforms will be the extent to which they mobilize substantial new resources for concessional lending in low-income countries. Adequate replenishments for the World Bank’s International Development Association are essential.

In addition to the MDBs, international institutions–prominently the WHO–play essential roles in providing international public goods for health. As mentioned earlier, a recent investment case points to some of the domains we have identified as important earlier in this section.^197^ The price is small for the returns realized, and enhanced support is a priority.

Finally, we support the Brazilian G20 presidency’s call for an international agreement on a minimum income tax on billionaires (the final summit of this presidency will be in Rio on November 18–19, 2024).^309^ This tax could generate additional funding for GPGs.

## Conclusions

The Commission reached seven conclusions:

First, dramatic improvements in human welfare are achievable everywhere by 2050 with the right health investments. Countries that choose to do so can halve their PPD by the middle of the century, achieving “50 by 50.” Historical experience and continued scientific advance indicate the feasibility of this goal. The interventions for achieving “50 by 50” will also reduce morbidity and disability at all ages.

Second, rapid, sharp mortality decline and associated decline in morbidity can be achieved relatively early on the pathway to full UHC. The 2035 and 2050 mortality reduction targets described in this report can be reached through tackling a remarkably narrow set of just 15 conditions. Eight of these are infections and maternal health conditions (the I-8) and seven are NCDs and injuries (the NCD-7).

Third, a modular approach to HSS supports an initial tight focus on these 15 priority conditions and a gradual broadening of effort as they become more fully addressed. Adopting this focused approach also addresses major morbidities, such as psychiatric illness, not already covered by mortality-reducing interventions. Value for money can be assessed through a two-step process: technical cost effectiveness to assess how best to achieve module-specific goals and political evaluation of trade-offs in investing in expanding module coverage.

Fourth, publicly financing a short list of drugs and other commodities can steer health systems toward delivering high priority health interventions. It would be valuable for ministries of finance to transfer a substantial and increasing fraction of budget transfers to making available and affordable the specific drugs, vaccines, diagnostics, and other commodities that are currently available for control of the 15 priority conditions. The Arrow mechanism described in our report includes direct subsidy of drugs, pooled purchasing, quality assurance, and a long-term commitment to manufacturers to ensure a steady supply of medications.

Fifth, tobacco control is by far the most important intersectoral policy to help achieve “50 by 50,” given the number of deaths caused by tobacco and the established and improving capacity of governments to implement tobacco policy. As Bloomberg and Summers say, “raising taxes on tobacco can do more to reduce premature mortality than any other single health policy.”^211^ A high level of tobacco taxation is essential, and valuable in the short to medium term for public finance, and should be accompanied by a package of other tobacco control policies.

Sixth, the huge variation across countries in excess deaths during the COVID-19 pandemic, particularly before COVID-19 vaccines were developed, points to lessons from successful countries about public health basics. These include rapid response, isolation of infected individuals, quarantine of those exposed, and social and financial support for those isolating or quarantining. In the next pandemic, these fundamentals will help to avert mortality while waiting for vaccine development and deployment. The six conclusions above are primarily aimed at national governments; our seventh is aimed at the development assistance community. We conclude that ODA should focus on two broad purposes. The first is to provide direct financial and technical support to countries with the least resources—to help control diseases and develop health systems. The second is to finance global public goods, including strengthening data systems; reducing the development and spread of antimicrobial resistance; preventing and responding to pandemics; fostering global health leadership and advocacy; identifying and spreading best practices; and developing and deploying new health technologies. For both purposes, focusing efforts on the 15 priority conditions would best contribute to “50 by 50.”

### Taking stock: from 2024 to 2050

Our report acknowledges the multiple headwinds to global health progress, including rising geopolitical tensions, increasingly manifest climate change, growth in nationalistic populism, slowed progress towards UHC, and rising healthcare costs. Yet despite these challenges, our analysis shows a practical pathway to halving premature death by 2050—the prize of “50 by 50”—is a prize within reach. By focusing resources against a narrow set of conditions and scaling up financing to develop new health technologies, we believe that the global health landscape can be utterly transformed in this way within our lifetimes.

GH2035 provided systematic evidence on the high value of mortality declines in much of the world — often a value that was a substantial fraction of GDP growth. We have updated those findings through 2019 and reiterate the high economic value of actually experienced mortality declines. Today, the case is better than ever for the value of investing in health for reducing mortality and morbidity, alleviating poverty, and improving human welfare.

## Supplementary Material

Appendix (final accepted version)

## Figures and Tables

**Table 1:** Recent health outcome performance, world’s 30-most populous countries

	Probability of premature death in 2019		Time required to halve the probability of premature death (at rate of improvement between 2010 and 2019)		Probability of premature mortality relative to income	
	Rank	%	Rank	Years	Rank	Percentage points
World	-	31%	-	51 years	-	-
Japan	1	12	10	37	9	−5
Italy	1	12	14	46	10	−5
Korea, Rep.	1	12	1	18	11	−5
Spain	4	13	15	50	8	−6
United Kingdom	5	16	17	58	16	0
France	6	16	17	58	17	0
Germany	7	17	22	>75	24	6
China	8	21	9	36	1	−12
Türkiye	9	22	8	30	12	−5
United States	10	22	30	>75	29	16
Colombia	11	23	19	60	2	−12
Iran, Islamic Rep. of	12	23	7	29	3	−11
Thailand	13	25	13	46	7	−8
Brazil	14	26	11	42	6	−8
Vietnam	15	28	28	>75	5	−9
Bangladesh	16	30	3	24	4	−9
Mexico	17	30	29	>75	15	−1
Philippines	18	33	25	>75	13	−4
Russian Federation	19	34	4	26	25	7
India	20	36	12	43	14	−2
Egypt	21	37	21	>75	18	1
Indonesia	22	37	23	>75	20	1
Pakistan	23	41	24	>75	19	1
Ethiopia	24	42	6	29	21	1
Myanmar	25	43	15	50	23	3
Tanzania	25	43	2	22	22	2
South Africa	27	48	5	28	27	14
Congo, Democratic Rep. of	28	51	20	61	26	9
Kenya	29	55	26	>75	28	16
Nigeria	30	63	27	>75	30	24

Notes:

1. The probability of premature death (PPD) in a given year is defined as the probability that a person born in that year would die before age 70 if the age-specific mortality rates in that year continued. PPD values were calculated from World Population Prospects (2022) life tables for the year 2019.

2. The time required to halve PPD provides a summary measure of how rapidly PPD is improving in the period 2010–19. It is calculated on the assumption that the rate of improvement in PPD averaged over the years 2010–19 will continue. If r is the average rate of decline in PPD, expressed in % per year, then: time required to halve (years) = 69.3/r.

3. 2019 Values of PPD relative to income was predicted by a linear regression from 2019 GDP per capita in the 30 most populous countries. Values in the column show predicted (in %) minus actual (in %) and hence negative values indicate better than predicted performance.

**Table 2:** Economic valuation of loss from mortality changes, 2010–2019, 30 most populous countries

	Country ranking		Indicator values	
	Value of change in full income, 2010–2019	Value of income change, 2010–2019^a^	Value of mortality change, 2010–2019 ^a^	Value of full income change, 2010–2019 ^a^
World		2%	2%	4%
Bangladesh	5	6%	2%	8%
Brazil	28	0%	1%	1%
China	3	9%	1%	10%
Congo, Dem. Rep.	7	3%	5%	7%
Colombia	18	3%	1%	4%
Germany	21	2%	1%	3%
Egypt, Arab Rep.	20	1%	1%	3%
Spain	23	1%	1%	2%
Ethiopia	1	8%	5%	14%
France	26	1%	1%	2%
United Kingdom	24	1%	1%	2%
Indonesia	10	5%	1%	6%
India	4	6%	2%	9%
Iran, Islamic Rep.	30	−1%	1%	1%
Italy	29	0%	1%	1%
Japan	22	1%	1%	2%
Kenya	9	4%	2%	6%
Korea, Rep.	17	3%	1%	4%
Mexico	27	1%	0%	1%
Myanmar	2	8%	2%	10%
Nigeria	19	1%	3%	3%
Pakistan	14	3%	2%	5%
Philippines	12	5%	1%	6%
Russian Federation	16	2%	3%	4%
Thailand	15	3%	1%	4%
Türkiye	11	5%	1%	6%
Tanzania	6	3%	5%	8%
United States	25	2%	0%	2%
Vietnam	8	6%	0%	7%
South Africa	13	0%	5%	5%

Notes:

a. Valuation methods used in these calculations follow the suggestions of the Harvard Benefit-Cost Analysis Reference Case Guidelines (Robinson LA, Hammit JK et al. 2019). Numbers expressed relative to level of income in 2010 and expressed as the average annual value of the total change.

Source from reference 20

**Table 3:** Halving premature death in 31 years: 37 country successes in the past half century

	Initial year	Initial level of PPD
**Central and Eastern Europe**		
Armenia	1988	64%
**China** ^a^	1970	61
**Latin America and Caribbean**		
Chile	1970	49
Costa Rica	1970	42
El Salvador	1980	71
Nicaragua	1979	67
Peru	1970	69
**Middle East and North Africa**		
Algeria	1970	76
Bahrain	1988	32
Iran, Islamic Rep. ^a^	1976	58
Israel	1973	33
Lebanon	1975	57
Oman	1970	66
Qatar	1982	33
Saudi Arabia	1970	60
State of Palestine	1973	91
Tunisia	1970	60
United Arab Emirates	1972	50
**North Atlantic**		
Cyprus	1974	64
Iceland	1974	29
Ireland	1978	34
Italy ^a^	1973	32
Luxembourg	1974	38
Malta	1970	38
Norway	1987	25
**Sub-Saharan Africa**		
Cabo Verde	1986	48
Sudan	1988	87
**Western Pacific and Southeast Asia**		
Australia	1970	38
Bangladesh ^a^	1971	95
Cambodia	1975	100
Japan^a^	1970	33
Maldives	1970	71
New Zealand	1977	34
Republic of Korea ^a^	2000	25
Singapore	1970	43
Timor-Leste	1976	94
Vietnam ^a^	1972	63

Notes:

a = Among the 30 most populous countries in the world

PPD = The probability of premature death (defined as the probability that a child born in the indicated year would die before age 70 if the age-specific death rates prevailing at the year of birth were to continue unchanged). The table shows PPD for both sexes combined.

Source from reference 14

**Table 4:** Probability of premature death in 2019 and year to achieve 30% reduction in PPD by 2035 and 50% reduction by 2050, both sexes combined

	PPD, 2019 (%)	Year to achieve target reduction in PPD	
		30%	50%
**Central Asia**			
Azerbaijan	29%	2032	2044
Mongolia	38	2032	2045
Tajikistan	35	2035	2050
**Central and Eastern Europe**			
Estonia	24	2034	2049
Russian Federation^a^	34	2032	2045
Ukraine	32	2034	2047
**Latin America and Caribbean**			
Haiti	47	2026	2032
**Middle East and North Africa**			
Algeria	21	2033	2045
Iran^a^	23	2034	2048
Iraq	33	2031	2042
Kuwait	16	2034	2049
Morocco	28	2033	2046
Oman	20	2035	2050
Qatar	14	2029	2038
Türkiye^a^	22	2035	2049
**North Atlantic**			
Finland	15	2034	2047
Ireland	13	2032	2045
Norway	12	2032	2044
**Sub-Saharan Africa**			
Botswana	48	2033	2046
Eswatini	59	2028	2037
Ethiopia^a^	42	2034	2048
Lesotho	70	2035	2050
Malawi	52	2035	2050
Mozambique	53	2033	2047
Namibia	55	2033	2046
South Africa^a^	48	2033	2047
United Republic of Tanzania^a^	43	2030	2041
Zambia	50	2033	2046
Zimbabwe	57	2030	2041
**Western Pacific and Southeast Asia**			
Bangladesh^a^	38	2031	2043
Dem. People’s Republic of Korea	30	2031	2043
Maldives	30	2034	2049
Republic of Korea^a^	14	2028	2037

Notes:

a = among the 30 most populous countries in the world. Countries projected to achieve a 50% reduction in PPD, based on the rate of decline from 2010 to 2019, by 2050 are categorized by CIH region. Countries that are not on track to meet their targets by 2035 or 2050 have been excluded from the list. This analysis considers only countries with a population exceeding 5 million.

Source from reference 14

**Table 5:** Percent of gap in life expectancy compared to the North Atlantic country accounted for by priority conditions, 2019

		% of gap accounted for by:		
Region	2019 gap (years)	I-8	NCD-7	Priority conditions

Central & Eastern Europe	7.1	7%	70%	77%
Central Asia	15	30%	46%	77%
China	4.4	4%	82%	85%
India	11	31%	48%	79%
Latin America & Caribbean	7.3	21%	39%	60%
Middle East & North Africa	7.9	14%	59%	72%
Sub-Saharan Africa	21	55%	21%	76%
United States	3.2	3%	45%	48%
Western Pacific & Southeast Asia	7.0	30%	50%	80%
World	9.6	36%	44%	79%

Note: Life expectancy in the North Atlantic was 82 years in 2019. The priority conditions are I-8 plus the NCD-7, as defined below.

The priority infections and maternal health conditions (I-8) are neonatal conditions, lower respiratory infections, diarrheal diseases, HIV/AIDS, tuberculosis, malaria, childhood-cluster diseases and maternal conditions.

The priority NCDs and injuries (NCD-7) are atherosclerotic cardiovascular diseases, hemorrhagic stroke, NCDs strongly linked to infections, NCDs strongly linked to tobacco use, diabetes, road injury and suicide.

Source from reference 80

Data from reference 11 and 15

**Table 6:** Changes in life expectancy 2000–2019 attributable to priority conditions

	World	Sub-Saharan Africa	India	China	North Atlantic
	
**Life expectancy in 2000, years**	66.5	50.8	62.7	71.9	78.6
**Total change 2000–2019. years**	6.3	10	8.2	6.1	3.8
**Changes attributable to priority conditions. years (%):**					
**Total I-8**	3.5 (56)	8.4 (84)	7.4 (90)	1.6 (26)	0.27 (7)
Childhood-cluster diseases	0.40 (6)	0.78 (8)	0.80 (10)	0.06 (0.9)	~0 (~0)
Diarrheal diseases	0.59 (9)	0.99 (10)	1.7 (21)	0.13 (2)	−0.02 (−0.5)
HIV/AIDS	0.46 (7)	2.8 (28)	0.52 (6)	~0 (~0)	0.04 (1)
Lower respiratory infections	0.51 (8)	0.72 (7)	0.80 (10)	0.49 (8)	0.22 (6)
Malaria	0.17 (3)	0.90 (9)	0.06 (0.8)	0 (0)	0 (0)
Maternal conditions	0.09 (1)	0.34 (3)	0.25 (3)	0.01 (0.2)	~0 (~0)
Neonatal conditions	0.67 (11)	0.46 (5)	1.5 (18)	0.74 (12)	0.01 (0.4)
Tuberculosis	0.63 (10)	1.4 (14)	1.8 (22)	0.14 (2)	0.01 (0.3)
**Total NCD-7**	1.9 (30)	0.53 (5)	−0.35 (−4)	3.1 (50)	3.1 (83)
Atherosclerotic CVDs	0.71 (11)	0.08 (0.8)	−0.45 (−6)	0.10 (2)	2.0 (53)
Diabetes	−0.03 (−0.5)	−0.02 (−0.2)	−0.18 (−2)	0.06 (0.9)	0.08 (2)
Hemorrhagic stroke	0.34 (5)	0.17 (2)	0.05 (0.6)	0.87 (14)	0.19 (5)
Infection-related NCDs	0.27 (4)	0.15 (1)	0.19 (2)	0.54 (9)	0.18 (5)
Tobacco-related NCDs	0.38 (6)	0.04 (0.4)	−0.16 (−2)	1.1 (19)	0.39 (10)
Road injury	0.13 (2)	0.12 (1)	0.10 (1)	0.17 (3)	0.23 (6)
Suicide	0.10 (2)	~0 (0.1)	0.10 (1)	0.19 (3)	0.05 (1)
**Total other causes**	0.93 (15)	1.1 (11)	1.2 (14)	1.5 (24)	0.37 (10)

Note: Changes in years of life expectancy attributable to specific causes of death are shown, with the percentage of total change attributable to each cause in parentheses. Percentages of total change are negative if the impact of the specific cause was in the opposite direction of the total change (eg, if there was an increase in life expectancy, the increase would have been larger had it not been for rising mortality from that cause).

Source from reference 80

Data from reference 11 and 15

**Table 7:** Worldwide progress against the infections and maternal health conditions, 2000–2010, 2010–2019 and 2019–2021

	Deaths (thousands)			Death rate			Average annual rate of change in death rate, %		
	2000	2019	2021	2000	2019	2021	2000–10	2010–19	2019–21

Tuberculosis	2,600	1,300	1,400	42^a^	17	18	−3.8%	−5.3%	1.4%
HIV/AIDS	1,700	720	650	27^a^	9.3	8.2	−3.9%	−7.1%	−6.0%
Malaria	870	590	620	14^a^	7.6	7.8	−3.2%	−3.3%	1.7%
Maternal deaths	410	240	260	310^b^	170	190	−3.3%	−2.6%	5.1%
Under-15 deaths	12,000	6,500	6,200	88^c^	46	45	−3.5%	−3.1%	−1.6%

Notes:

a Deaths per 100,000 population.

b Deaths per 100,000 births.

c Deaths per 1000 births.

Note: Numbers are rounded. The under-15 mortality rate is an approximate summary of the following priority conditions: neonatal conditions, diarrheal diseases, lower respiratory infections, and childhood-cluster diseases.

Data sources: Under-15 mortality was calculated from UN WPP 2022 life tables. For other outcomes, the number of deaths from each cause and total deaths were obtained from the WHO GHE 2021: The proportion of total deaths from each cause obtained from the WHO GHE was multiplied by the total number of deaths obtained from reference 2. Number of live births and mid-year population were obtained from reference 2.

**Table 8:** Mortality rates from NCD-7 conditions, rates of change from 2000 to 2019, ages 50–69, by CIH region.

	NCD-7	Atheroscler otic CVD	Diabetes	Hemorrhag ic stroke	Infection-associated NCDs	Strongly tobacco-associated NCDs	Road injury	Suicide
World	−1.6%	−1.4%	0.11%	−2.3%	−2.3%	−1.7%	−0.43%	−2.3%
Central Asia	−1.7%	−1.6%	0.74%	−2.4%	−2.3%	−2.4%	−0.71%	−2.5%
Central and Eastern Europe	−2.6%	−2.8%	−0.11%	−3.8%	−2.6%	−1.7%	−3.3%	−3.7%
China	−2.4%	−0.45%	−1.1%	−3.7%	−3.6%	−2.8%	−0.72%	−3.5%
India	−0.76%	−0.31%	0.42%	−1.6%	−1.4%	−1.1%	−0.31%	−1.4%
Latin America and Caribbean	−1.5%	−1.8%	−0.04%	−2.8%	−1.9%	−1.5%	−0.43%	−0.49%
Middle East and North Africa	−1.4%	−1.7%	−0.46%	−2.7%	−1.4%	−0.32%	−0.27%	−0.77%
North Atlantic	−2.4%	−3.8%	−1.8%	−3.3%	−2.3%	−1.1%	−4.0%	−0.56%
Sub-Saharan Africa	−1.5%	−1.4%	−1.1%	−2.2%	−1.7%	−1.5%	−0.04%	−1.9%
United States	−1.2%	−2.0%	−0.02%	−1.0%	0.61%	−1.5%	0.69%	2.3%
Western Pacific and Southeast Asia	−0.75%	−0.18%	1.1%	−0.87%	−2.4%	−1.1%	−0.38%	−2.6%

Note: Table shows average annual rates of change in the mortality rate per 100,000 population per year in the age group 50–69. A negative average annual rate of change indicates a decline in mortality rates.

**Table 9:** A modular approach to health system strengthening

Health area	Module number/name^a^	High-priority interventions within module	Primary outcome metric(s) (secondary outcome metric)	Cost of expanding coverage to an additional 10% of persons in need^b^
*Community-based primary healthcare teams*				
I-8	1. Routine childhood immunization	Most or all antigens recommend by WHO for all countries (n=11)	Child deaths averted (child height for age)	0.22
	2. Treatment of acute childhood illness^c^	Treatment of enteric and lower respiratory infections, malaria, and acute malnutrition	Child deaths averted (child height for age)	2.2
	3. Pregnancy and childbirth services^d^	Antenatal care, safe delivery, management of labor complications, routine care for postpartum women, neonatal care	Maternal deaths averted (Stillbirths and neonatal deaths averted)	2.2
	4. Tuberculosis^d^	Treatment of infected persons, including those with drug-resistant tuberculosis,^d^ and preventive therapies among high-risk contacts	Adult deaths averted	0.87
	5. HIV/AIDS^d^	Long-term antiretroviral drug therapy for infected persons, preventive therapies among high-risk contacts	Adult deaths averted	4.1
NCD-7	6. Basic cardiovascular and respiratory care^d^	Combination drug therapy for persons at high CVD risk^e^, glycemic control and monitoring for microvascular complications in persons with diabetes, management of asthma and COPD	Adult deaths averted	7.1
	7. Mental health care^d^	Combination of drug therapy and psychotherapy for severe mood disorders, schizophrenia, and other serious and commonly occurring conditions^f^	Cases adequately managed for one year (Suicide deaths averted)	3.6
HS	8. Family planning	Contraception services appropriate to setting and patient preference	Unintended pregnancies averted (Couple-years of protection)	0.26
	9. School age child and adolescent development	School-based programs to deliver: deworming, immunization (e.g., HPV), screening for refractive error and oral health; excludes school feeding	Child height-for-age 15-year old math scores (Glasses coverage)	0.67
	10. Custodial and palliative care	Shared responsibility^g^ between health system and household for providing shelter, food, security, dignity and symptom management for conditions not amenable to functional integration (e.g., dementia, spinal cord injury) or treatment (e.g., metastatic ovarian cancer)	Cases adequately managed for one year	1.5
	11. Public health functions	Population-based interventions to improve disease prevention and control, including: case-finding efforts for TB and HIV, vector control efforts for malaria, mass drug administration for selected NTDs, micronutrient supplementation, and measures to identify and isolate infectious individuals during epidemics/pandemics	Child deaths averted Adult deaths averted	0.97
	12. Primary care functions	Integrated approaches to stable, common signs and symptoms in (includes essential diagnostics and supportive care)	N/A; enabling interventions	1.7
*Specialised first-level delivery platforms*				
NCD-7	13. Primary surgical care	Surgical services at first-level hospitals to address common surgical emergencies, focusing on injuries and digestive diseases	Adult deaths averted	3.7
	14.Enhanced cardiovascular and respiratory care	Long-term management of CKD and heart failure, treatment of acute cardiovascular and respiratory complications, secondary prevention of rheumatic heart disease	Adult deaths averted	3.2
HS	15. Rehabilitation	Essential rehabilitation services, focusing on post-acute CVD and injury care	Cases functionally reintegrated within one year	0.95
	16. Dental care	Treatment of infections and caries, dental extraction	DMF (decayed/missing/filled) burden reduced	0.49
	17. Emergency care functions	Integrated approaches to common emergency presentations in community, outpatient, and first-level hospital settings (including prehospital care), includes treatment of acutely ill persons during epidemic/pandemic^h^	N/A; enabling interventions	2.2
*Referral clinics and hospitals*				
NCD-7	18. Basic cancer care	Treatment of pre-cancer and early-stage cervical, breast, colorectal, and oral cancer (with curative intent)	Cases advanced to 10-year survival (Adult deaths averted)	1.2
	19.Enhanced cancer care	Organized screening programs for first-tier cancers, treatment of selected cancers with potential for long-term remission^i^	Cases advanced to 10-year survival (Adult deaths averted)	13

Notes:

a. A modular structure for a country, or for a region in a country, will depend on local epidemiology, system characteristics, and preferences. The CIH table is intended only to serve as an example and a possible starting point.

b. Incremental annual cost of increasing population coverage of all the high-priority interventions in the module by 10%, expressed in basis points of GDP per year. A basis point is one percent of one percent. Note: analysis done only for low- and lower-middle-income countries (n = 82). Source: reference 126

c. In many countries, these interventions will be delivered using the Integrated Management of Childhood Illness approach.

d. Facility-based care is an important delivery modality for many of the interventions that address these conditions. Additionally, a subset of persons with these conditions, dedicated facilities or clinics will be needed for enhanced care, eg, to manage complex cases and provide care to key subpopulations.

e. Includes “secondary prevention” among those with established cardiovascular disease.

f. Conditions include: psychotic disorders, bipolar disorder, depressive disorders, anxiety disorders, and trauma disorders, and opioid use disorder.

g. Many countries struggle to finance a generous package of long-term care services. However, the cost of this caregiving can be a major economic burden on households and falls disproportionately on women and girls. Countries with sufficient resources should consider providing transfer payments to households to offset unpaid care and related expenses.

h. Some of this will be long-term rather than emergency care

i. The cancers in this list will vary considerably by country and as medical care improves; examples include: common childhood leukaemia and lymphoma, prostate cancer, uterine cancer, Hodgkin and selected non-Hodgkin lymphomas in adults, thyroid cancer, and kidney cancer.

**Table 10:** Health financing indicators, rate of change over past decades

	AARC of GDP per person	AARC of CHE per person	AARC of GGHE-D per person	AARC of GGHE-D/CHE	AARC of OOP/CHE
**2000–2010**					
Central and Eastern Europe	4.2%	5.9%	5.6%	0.0%	0.2%
Central Asia	7.5%	5.2%	3.4%	−1.5%	0.3%
China	9.8%	9.1%	18.9%	9.0%	−3.8%
India	5.9%	3.7%	6.1%	2.4%	−0.9%
Latin America and Caribbean	2.0%	3.4%	4.7%	0.8%	−1.4%
Middle East and North Africa	0.1%	1.0%	1.5%	0.8%	−1.2%
North Atlantic	1.0%	2.9%	3.1%	0.1%	−0.9%
Sub-Saharan Africa	2.6%	3.5%	4.1%	−0.6%	−2.0%
United States	0.8%	3.4%	4.4%	1.0%	−2.0%
Western Pacific and Southeast Asia	1.5%	3.0%	3.1%	−0.6%	−0.8%
**World average**	1.3%	2.9%	3.2%	0.0%	−1.1%
					
**2010–2019**					
Central and Eastern Europe	2.6%	2.5%	2.4%	0.2%	−0.3%
Central Asia	2.0%	5.0%	2.4%	−0.8%	0.6%
China	6.0%	8.5%	9.4%	0.8%	−1.5%
India	4.8%	3.7%	6.8%	3.0%	−2.2%
Latin America and Caribbean	1.0%	2.2%	2.9%	0.9%	−1.0%
Middle East and North Africa	1.5%	4.1%	4.5%	0.2%	−1.9%
North Atlantic	1.1%	1.1%	1.0%	−0.1%	−0.1%
Sub-Saharan Africa	0.4%	1.3%	2.1%	0.4%	−0.6%
United States	1.2%	1.5%	2.1%	0.6%	−0.9%
Western Pacific and Southeast Asia	1.5%	2.9%	3.1%	1.0%	−0.9%
**World average**	1.2%	1.8%	1.8%	0.3%	−0.6%
					
**2000–2019**					
Central and Eastern Europe	3.6%	4.4%	4.2%	0.1%	−0.1%
Central Asia	5.0%	5.4%	3.1%	−1.2%	0.4%
China	8.3%	9.3%	14.8%	5.0%	−2.8%
India	5.6%	3.9%	6.8%	2.8%	−1.7%
Latin America and Caribbean	1.6%	2.9%	4.0%	0.8%	−1.2%
Middle East and North Africa	0.8%	2.7%	3.2%	0.5%	−1.6%
North Atlantic	1.1%	2.1%	2.2%	0.0%	−0.5%
Sub-Saharan Africa	1.6%	2.5%	3.3%	−0.1%	−1.4%
United States	1.1%	2.6%	3.5%	0.8%	−1.5%
Western Pacific and Southeast Asia	1.6%	3.1%	3.3%	0.2%	−0.9%
**World average**	1.3%	2.5%	2.6%	0.2%	−0.9%

Notes: AARC = average annual rate of change; CHE = current health expenditure; GGHE-D = general government health expenditure from domestic sources; OOP = out-of-pocket spending. Source from reference 142

**Table 11:** Number of deaths and crude death rate (CDR) per 1000 population

	Number of deaths (thousands)	Deaths as a % of deaths in 2019		CDR per 1000	CDR as a % of CDR in 2019	
	2019	2035	2050	2019	2035	2050
						
World	58,000	126	158	7	110	126
						
Central & Eastern Europe	4,000	101	103	12	107	117
Central Asia	2,000	130	170	7	99	105
China	10,000	135	173	7	137	187
India	9,000	125	158	7	110	130
Latin America & Caribbean	4,000	127	162	7	114	140
Middle East & North Africa	3,000	141	202	5	116	146
North Atlantic	4,000	115	132	9	113	131
Sub-Saharan Africa	10,000	126	162	9	86	83
United States	3,000	126	148	8	117	132
Western Pacific & Southeast Asia	8,000	130	160	7	118	141

Source: from reference 11

**Table 12:** Size of working age population and old age dependency ratios

	Working-age pop. (thousands)	Working-age pop. as a % of 2019		Old-age dependency ratio (%)		
	2019	2035	2050	2019	2035	2050
						
World	5,041,000	114	121	14	20	26
						
Central & Eastern Europe	221,000	91	78	25	33	45
Central Asia	207,000	141	178	7	9	11
China	991,000	94	77	17	34	51
India	926,000	117	121	10	15	22
Latin America & Caribbean	435,000	111	111	13	20	30
Middle East & North Africa	361,000	125	136	8	14	23
North Atlantic	296,000	95	89	31	45	53
Sub-Saharan Africa	612,000	158	224	6	6	8
United States	219,000	102	104	24	35	39
Western Pacific & Southeast Asia	773,000	108	108	15	22	30

Note: Working age is ages 15–64 years. Old age dependency ratio is defined as percentage of the total population that is over 64 divided by the percentage of the total population that is working age. Data source: from reference 11

**Table 13:** Out-of-pocket payment (OOP) for drugs, selected countries, recent years

Country (Year*)	OOP per person for drugs (2021 constant US$)	OOP for drugs as % of GDP	Total drug expenditure as % of health expenditure	% of drug expenditure paid from OOP
Afghanistan (2017)	26	5.1	41	100
Armenia (2021)	193	3.9	32	100
Canada (2021)	504	1.0	13	60
Costa Rica (2021)	78	0.6	8	99
Dominican Republic (2019)	49	0.6	18	80
Egypt (2021)	52	1.3	29	100
Fiji (2019)	20	0.3	8	100
India (2020)	15	0.7	21	100
Malaysia (2021)	32	0.3	7	98
Mexico (2021)	132	1.3	22	100
Nepal (2021)	21	1.8	33	99
North Macedonia (2021)	93	1.4	23	72
Qatar (2017)	40	0.1	8	22
Republic of Moldova (2021)	71	1.3	21	84
Sri Lanka (2017)	17	0.4	13	100
Suriname (2019)	34	0.5	11	56
Uzbekistan (2018)	34	1.8	36	100

Notes: Total drug expenditures include both prescribed drugs and over-the-counter (OTC) drugs. Only countries reporting both total expenditure and OOP for both for prescription OTC drugs are included. Private health expenditure is used to estimate OOP, as there is no OOP reported for drugs.

* We report the latest year that is available. Data sources from reference 142

**Table 14:** Covid 19 – Outcomes for the 30 most populous countries, January 2020 through May 4, 2023

Rank	Country	Excess deaths as a percent of baseline (P-score)^a^				Economic value of welfare loss as a percent of 2019 income ^b^
		January 2020 through May 4, 2023	2020	2021	January 2022 through May 4, 2023	
	World	13%	8.8%	21%	11%	−39%
1	Japan	3.8	−1.0	2.0	8.7	−26
2	China	5.4	−1.1	3.7	11	−11
3	Korea, Rep.	6.4	0	2.4	14	−11
4	Nigeria	6.9	6.0	9.6	5.5	−41
5	France	7.1	9.2	6.5	6.0	−13
6	Germany	8.1	4.8	8.0	11	−20
7	Thailand	9.2	0.1	11	14	−28
8	Spain	11	18	7.7	9.0	−23
9	United Kingdom	12	15	11	10	−25
10	Congo, Democratic Rep. of	12	4.0	19	12	−48
11	Kenya	12	6.7	18	11	−49
12	Italy	13	18	12	11	−34
13	Philippines	13	−1.2	38	5.6	−26
14	Tanzania	14	7.2	24	12	−35
15	United States	14	17	18	8.4	−42
16	Indonesia	15	1.7	31	14	−47
17	Pakistan	16	16	24	9.2	−43
18	Vietnam	16	0.3	21	25	−36
19	Türkiye	17	16	28	8.7	−30
20	Egypt	17	18	31	6.3	−39
21	South Africa	17	11	37	6.9	−58
22	Myanmar	17	8.6	31	13	−57
23	Brazil	18	13	33	11	−45
24	Bangladesh	19	20	29	12	−40
25	Iran, Islamic Rep. of	20	28	32	5.0	−37
26	Colombia	20	20	38	6.7	−39
27	Mexico	21	36	34	1.7	−62
28	India	21	12	40	15	−58
29	Ethiopia	21	9.3	32	22	−58
30	Russian Federation	26	21	40	20	−113

a Excess deaths are estimated for the period starting on January 1, 2020 and concluding on May 4, 2023. (The World Health Organization (WHO) declared the end of the COVID-19 emergency phase on May 5, 2023.) Baseline deaths were based on deaths in 2019 and the annual rate of change (averaged between 2015–2019) from reference 11. P-scores are defined by dividing estimated excess deaths by the projected baseline number of deaths in the same period. Excess deaths are from *The Economist* (downloaded from Our World in Data).

b Numbers expressed relative to level of income in 2019 and represent the loss for the entire pandemic period. Economic value of mortality loss was calculated by first calculating excess death rates for ages below and above 75 years for each country. A value per statistical life (VSL)-to-income ratio of 160 was applied, following the suggestions of the Harvard Benefit-Cost Analysis Reference Case Guidelines from reference 19. The value of excess death rates in the older age group was adjusted based on the ratio of the remaining life expectancy of 80-year-olds to 40-year-olds. Economic value is expressed as GNI per capita in 2017 constant international dollars and adjusted for purchasing power parity (PPP).

**Table 15:** The probabilities of pandemics with greater than one million, 10 million, 25 million, and 100 million deaths ^a^

Deaths	Annual probability	5 Year probability	10 Year probability	25 Year probability
1 million or more	6.3%	28%	48%	80%
10 million or more	4.2	19	35	66
25 million or more ^b^	2.6	12	23	48
100 million or more	0.6	3.0	5.8	14

a Global pandemics refers to respiratory pandemics caused by influenza viruses or coronaviruses.

b During the emergency phase of the Covid-19 pandemic (January 2020 through May 4, 2023) there were an estimated 26 million excess deaths globally, almost entirely attributable, directly or indirectly, to COVID-19.

Source from reference 47

**Table 16:** Expected value of losses from pandemic risk – deaths and income

	Expected value of annual deaths(per 10,000)	Expected value of annual deaths (thousands)	Impact on PPD (percentage points)	Impact on Life expectancy (years)	Expected value of mortality loss (% of GNI)^a^
World	3.2	2,500	1.4	−0.77	5.1%
Central and Eastern	2.5	82	1.4	−0.78	4.0
Europe					
Central Asia	4.4	160	1.2	−0.64	7.0
China	2.4	340	1.7	−0.85	3.8
India	3.2	450	1.3	−0.71	5.1
Latin America and	2.8	180	1.5	−0.79	4.5
Caribbean					
Middle East and North	3.0	160	1.5	−0.77	4.8
Africa					
North Atlantic	2.3	100	1.8	−0.97	3.7
Sub-Saharan Africa	5.0	580	1.0	−0.55	8.0
United States	2.2	71	1.6	−0.90	3.5
Western Pacific and	3.0	340	1.5	−0.82	4.8
Southeast Asia					

Notes: Pandemic risk refers to global coronavirus or influenza pandemics.

a. Economic value of mortality loss was calculated by multiplying the annual death rate with a value per statistical life (VSL)-to-income ratio of 160, following the suggestions of the Harvard Benefit-Cost Analysis Reference Case Guidelines (Robinson et al, 2019). Numbers are expressed relative to level of income in 2019. Economic value is expressed as GNI per capita in 2017 constant international dollars and adjusted for purchasing power parity (PPP)

Data source from reference 191. Life tables were obtained from reference 11. Average annual age specific mortality rate from pandemics was estimated from reference 47 (Table 9).

Source: Source: First two columns, from reference 47. Remaining columns, CIH calculations.

**Table 17:** Funding for global functions by function and sub-function, 2020–2022

Function and Sub-Function	2020	2021	2022
Global public goods	$4700	$4600	$4000
Product development	$3800	$3800	$3300
Development/harmonization of health regulations	$85	$110	$96
Knowledge generation	$750	$710	$620
Intellectual property	$0.00	$0.20	$0.10
Externalities	$8200	$14 000	$12 000
Outbreak preparedness and response COVID-19	$5500 ($4400)	$11 000 ($8800)	$9300 ($5800)
AMR	$230	$320	$160
Responses to marketing of unhealthful products	$110	$130	$100
Control of cross-border disease movement	$2400	$2400	$2600
Leadership and stewardship	$420	$440	$360
Health advocacy	$390	$420	$350
Aid effect/accountability	$30	$24	$14
Country-specific functions	$26 000	$26 000	$31 000
Priority infections and maternal health conditions	$21 000	$20 000	$24 000
NCDs and health systems strengthening Noncommunicable diseases Health system strengthening	$4600 ($250) ($4300)	$5700 ($270) ($5400)	$7400 ($230) ($7200)
Total	$39 000	$45 000	$48 000

Notes: Gross disbursements. Constant 2021 prices (USD millions). All data have been rounded to a maximum of two significant figures. DAH+ refers to ODA, private development finance, and funding for neglected disease product development. Numbers in parentheses are a fraction of the total value of the respective sub-functions.

Source from reference 50

**Table 18:** Notable and potential launches of vaccines, therapeutics, and diagnostics to address I-8 and NCD-7 conditions

Disease	Notable launches between 2000 and 2024*	Potential launches between 2025 and 2040*
**IMH8**		
Childhood cluster diseases	PEDIARIX – a combination vaccine indicated for active immunization against diphtheria, tetanus, pertussis, infection caused by all known subtypes of hepatitis B virus, and poliomyelitis; can be given between six weeks and six years of age. ProQuad – a vaccine indicated for active immunization against measles, mumps, rubella, and varicella in children 12 months through 12 years of age.	
Diarrheal diseases	Rotasiil – the first thermostable rotavirus vaccine capable of long-term storage at ambient temperatures below 25 degrees Celsius.	ZF0901 – a bivalent conjugate vaccine for the prevention *Shigella* infection. The vaccine contains O-specific polysaccharides from *S. flexneri 2a* and *S. sonnei.* CV638 – a live attenuated vaccine for the prevention of *Vibrio cholera* infection which has demonstrated a protective immune response in clinical studies.
HIV/AIDS	Azvudine – a novel nucleoside reverse transcriptase inhibitor with greater effectiveness against drug resistant HIV strains compared to standards of care. HIV 1/2 antibody (anti-HIV Ab) RDT – one of the first rapid diagnostic tests for HIV.	Ad26.Mos4.HIV, Clade C gp140 and Mosaic gp140 HIV bivalent vaccine – a mosaic-based protein developed using adenovirus vector platform and C6 production cell line technology, contains mosaic-based immunogens capable of producing an immune response to a wide verity of HIV-1 subtypes. The vaccine regimen is delivered in four vaccinations in one year, with the first two delivered in a single injection. Islatravir – a nucleoside reverse transcriptase translocation inhibitor which prevents HIV from multiplying by blocking the reverse transcriptase HIV enzyme. Islatravir is being developed and trialed as a fixed-dose combination with doravirine and as a stand-alone therapy.
Lower respiratory infections	Prevenar 13 – a thirteen-valent pneumococcal conjugate vaccine with higher effectiveness against all-cause otitis media compared to other pneumococcal conjugate vaccines. Abrysvo – the first vaccine for pregnant individuals to prevent RSV in infants.	MCDA-LFB – ultra-efficient multiple cross displacement amplification-lateral flow biosensor for serogroup identification of Neisseria meningitidis.
Maternal conditions	Pitocin – a synthetic oxytocin for the treatment of postpartum hemorrhage. NovoSeven – a hemostatic recombinant activated factor VIIa for the treatment of severe blood loss from postpartum hemorrhage.	Enalapril – an angiotensin converting enzyme (ACE) inhibitor for the management of postpartum hypertension and improvement of postpartum cardiovascular function in patients with severe preeclampsia. Metoprolol – a beta-1 blocker for the treatment of high blood pressure in preeclampsia.
Malaria	Mosquirix – the first malaria vaccine approved for public use. Coartem – one of the first artemisinin-based combination therapies for the treatment of malaria.	Artemether/Lumefantrine/Amodiaquine – therapeutic combination has been shown to be a viable alternative to Artemether/Lumefantrine alone for first-line treatment of multidrug-resistant P falciparum malaria.
Neonatal conditions	Albuterol – a bronchodilator for the treatment of bronchospasm in preterm neonates.	Betamethasone – a long-acting corticosteroid with immunosuppressive and anti-inflammatory properties for fetal lung maturation in preterm birth. Pentoxifylline – a synthetic dimethylxanthine derivative for the treatment of impaired fetal growth.
Tuberculosis	Bedaquiline – treatment for multidrug resistant tuberculosis with a unique mechanism of action that inhibits the mycobacterial ATP synthase proton pump. Xpert MTB/RIF Assay – a novel integrated diagnostic device for the diagnosis of tuberculosis and rapid detection of rifampin resistance.	MTBVAC – the first and only live attenuated vaccine derived from a human isolate of *Mycobacterium tuberculosis.* Tubivac – the world's first orally available therapeutic vaccine used as an immune adjunct for tuberculosis chemotherapy. Taken in conjunction with standard tuberculosis medications, it reduces treatment time for the condition to one month.
**NCDI7**		
Atherosclerotic cardiovascular diseases	Colchicine – an alkaloid tablet to reduce the risk of myocardial infarction, stroke, coronary revascularization, and cardiovascular death in patients with atherosclerotic disease or other risk factors for cardiovascular disease. Bempedoic acid – an adenosine triphosphate- citrate lyase inhibitor to reduce risk of myocardial infarction and coronary revascularization in adults with cardiovascular disease who are unable to take statin therapies.	Adult and pediatric hydrochlorothiazide combinations – hydrochlorothiazide is a common treatment for hypertension; patents on hydrochlorothiazide combination therapies are soon to expire thereby increasing affordability and access in low- and middle-income countries.
Diabetes	Afrezza (insulin human) Inhalation Powder – the only inhaled, quick acting insulin commercially available. Insulin degludec (Tresiba) – long-acting insulin analog administered through subcutaneous injection for the treatment of hyperglycemia.	Semaglutide – an antidiabetic medication for type 2 diabetes and long-term weight management; soon to come off patent allowing for the development of cheaper biosimilars that will widen access.
Hemorrhagic stroke	Esmolol – an intravenous cardioselective β-1 adrenergic antagonist for blood pressure management.	Recombinant factor Vila – an intravenously administered blood factor undergoing phase IN trials for the treatment of hemorrhagic stroke.
NCDs strongly linked to infections	PreHevbrio – the first and only 3-antigen Hepatitis B vaccine. Gardasil – the first vaccine for the prevention of human papillomavirus infection.	Lenvervimab – a recombinant human immunoglobulin for the treatment of chronic hepatitis B virus infection. Antaitavir Hasophate and Yiqibuvir – ongoing phase III clinical trials to confirm efficacy and safety for the treatment of chronic hepatitis C in adult patients.
NCDs strongly linked to tobacco use	Repotrectinib – a kinase inhibitor for the treatment of ROS1-positive non-small cell lung cancer and NTRK-positive locally advanced or metastatic solid tumors. Roflumilast – orally administered tablet to reduce risk of COPD exacerbations in patients with severe COPD associated with chronic bronchitis.	MK-7684A – a fixed dose pembrolizumab/vibostolimab co-formulation with chemotherapy as a first line treatment for small cell lung cancer.
Road Injury	Forward collision warning and advanced emergency breaking – advanced driver assistance systems designed to reduce the risk and severity of collisions.	Vehicle-to-vehicle communication – use of wireless ad hoc networks to permit autonomous sharing and processing of data between vehicles thereby mitigating human error and reducing road collisions.
Suicide	Clozapine – the first and only medication with an indication for schizophrenia associated suicide prevention. Esketamine – an oral antidepressant for treatment-resistant depression and depressive symptoms in adults with major depressive disorder who experience suicidal thoughts or behaviors.	NRX-101 – a fixed dose combination oral capsule of D-cycloserine and lurasidone for the maintenance of remission from severe bipolar depression with acute suicidal ideation or behavior.

Note: See appendix table 9.1 on page 88 of the appendix and appendix table 9.2 on page 93 of the appendix
