# Supplementary material for: Global health 2050: the road to halving premature death by mid-century
Source: Lancet. Author manuscript; Available in PMC 2025 Aug 4. (PMC12320876; doi:10.1016/S0140-6736(24)01439-9)
Supplement: Appendix (final accepted version) [file NIHMS2094876-supplement-Appendix__final_accepted_version_.pdf]

GLOBAL HEALTH 2050

APPENDIX

the road to halving premature death by mid-century

THIRD COMMISSION ON INVESTING IN HEALTH

table of contents

TABLES 3

Table A.1 Locations by CIH region 3

Table A.2 Basic statistics for CIH regions, 2023 6

Table A.3 Measures of progress in age-specific mortality rates, life expectancy at birth, and probability of premature death 7

Table A.4 Basic statistics for the 30 most populous countries in 2023 8

Table A.5 The probability of premature death (PPD) relative to income, world's 30-most populous countries 9

Table A.6 The probability of premature death (PPD) in in 1970 and 2019, PPD progress between 2010-2019, and income in 2019 for 105 countries with a population greater than 5 million in 2023. 11

Table A.7 Probability of premature death in 2019 and year to achieve 30% reduction in PPD by 2035 and 50% reduction by 2050, both sexes combined 16

Table A.8 Changes in gap in life expectancy compared to the North Atlantic in 2019 attributable to I-8 18

Table A.9 Changes in gap in life expectancy compared to the North Atlantic in 2019 attributable to NCD-7 19

Table A.10 Progress against tuberculosis in the 30 countries with highest number of deaths, 2000–2010, 2010–2019 and 2019–2021 20

Table A.11 Progress against HIV/AIDS in the 30 countries with highest number of deaths, 2000–2010, 2010–2019 and 2019–2021 21

Table A.12 Progress against malaria in the 30 countries with highest number of deaths, 2000–2010, 2010–2019 and 2019–2021 22

Table A.13 Progress against maternal deaths in the 30 countries with highest number of deaths, 2000–2010, 2010–2019 and 2019–2021 23

Table A.14 Progress against under-15 deaths in the 30 countries with highest number of deaths, 2000–2010, 2010–2019 and 2019–2021 25

Table A.15 Changes in life expectancy attributable to priority conditions: 2000-2010, 2010-2019, 2019-2021 – World, Sub-Saharan Africa, India, China, North Atlantic 27

Table A.16 Changes in deaths from selected NCD-7 conditions decomposed into changes in age group size and age group specific mortality rates, by CIH region, 2000-2010, 2010-2019, and 2000-2019. 30

Table A.17 Health financing indicators, rate of change over past decades 63

Table A.18 Health Benefit Package on Essential Medicines....64

Table A.19 Summary of literature on OOP for drugs....66

Table A.20 P-scores January 31, 2020, to May 4, 2023, and rank for all countries with a population greater than 5 million....68

Table A.21 Excess deaths, excess deaths per million population, and p-scores for all countries January 31, 2020, to May 4, 2023....71

Table A.22 Components of an early intersectoral package of policy instruments....77

Table A.23 Notable and potential launches of vaccines, therapeutics, and diagnostics to address I-8 and NCD-7 conditions....79

Table A.24 Expanded list of notable past and potential number of future launches of vaccines, therapeutics, and diagnostics to address I-8 and NCD-7 conditions....82

Table A.25 Innovative candidate vaccines, therapeutics, and diagnostics that are likely to launch between 2025 and 2040....86

FIGURES....88

Figure A.1 Global and frontier probability of premature death (PPD), 1950–2021....88

Figure A.2 Sex differences in the rate of decline in PPD, 30 most populous countries, 2010-19....89

Figure A.3 Current levels of government tax revenue and health spending, by country....90

Figure A.4 Out-of-pocket payments as share of current health expenditure (%)....91

Figure A.5 Government health expenditure and out-of-pocket payment per person (%)....92

Figure A.6 P-scores by month: United States, Italy, Japan, and China – April 2020 to October 2023 (7-month moving averages)....93

Figure A.7 Correlation between May 4 2023 cumulative p-scores and excess deaths per 1000 population for the 30 most populous countries, January 1, 2020, to May 4, 2023....94

PANELS....95

Panel A.1 Projecting temperature-related mortality impacts from climate change....95

Panel A.1 Figure A Projected increase in annual heat-related deaths due to climate change compared to the 2015 baseline....96

Panel A.2 The importance of the “next 7,000 days”....98

Panel A.2 Figure A Prevalence of stunting among females in selected countries....99

Panel A.2 Figure B Prevalence of stunting among males in selected countries....100

Panel A.2 Figure C Mathematics tests scores relative to Singapore, PISA 2022, selected countries....101

Panel A.2 Table A Essential health services appropriate for children and adolescents as proposed by DCP3....103

Panel A.3 New tools for prevention and treatment of tuberculosis....105

Panel A.4 Developing antibiotics in the age of AI....107

TABLE A.1

Locations by CIH region

**CENTRAL ASIA**		
Afghanistan	Azerbaijan	Kazakhstan
Kyrgyz Republic	Mongolia	Pakistan
Tajikistan	Turkmenistan	Uzbekistan
**CENTRAL AND EASTERN EUROPE**		
Albania	Armenia	Belarus
Bosnia and Herzegovina	Bulgaria	Croatia
Czech Republic	Estonia	Georgia
Hungary	Latvia	Lithuania
Moldova	Montenegro	North Macedonia
Poland	Romania	Russian Federation
Serbia	Slovak Republic	Slovenia
Ukraine		
**CHINA**		
**INDIA**		
**LATIN AMERICA AND CARIBBEAN**		
Argentina	Bahamas, The	Belize
Bolivia	Brazil	Chile
Colombia	Costa Rica	Cuba
Dominican Republic	Ecuador	El Salvador
Guatemala	Guyana	Haiti
Honduras	Jamaica	Mexico
Nicaragua	Panama	Paraguay
Peru	Suriname	Trinidad and Tobago
Uruguay	Venezuela, RB	
**MIDDLE EAST AND NORTH AFRICA**		
Algeria	Bahrain	Egypt, Arab Rep.
Iran, Islamic Rep.	Iraq	Israel
Jordan	Kuwait	Lebanon
Libya	Morocco	Oman
Qatar	Saudi Arabia	Syrian Arab Republic
Tunisia	Türkiye	United Arab Emirates

Yemen, Rep.		
**NORTH ATLANTIC**		
Austria	Belgium	Canada
Cyprus	Denmark	Finland
France	Germany	Greece
Iceland	Ireland	Italy
Luxembourg	Malta	Netherlands
Norway	Portugal	Spain
Sweden	Switzerland	United Kingdom
**SUB-SAHARAN AFRICA**		
Angola	Benin	Botswana
Burkina Faso	Burundi	Cabo Verde
Cameroon	Central African Republic	Chad
Comoros	Congo, Dem. Rep.	Congo, Rep.
Côte d'Ivoire	Djibouti	Equatorial Guinea
Eritrea	Eswatini	Ethiopia
Gabon	Gambia, The	Ghana
Guinea	Guinea-Bissau	Kenya
Lesotho	Liberia	Madagascar
Malawi	Mali	Mauritania
Mauritius	Mozambique	Namibia
Niger	Nigeria	Rwanda
Senegal	Sierra Leone	Somalia
South Africa	South Sudan	Sudan
Tanzania	Togo	Uganda
Zambia	Zimbabwe	
**UNITED STATES**		
**WESTERN PACIFIC AND SOUTHEAST ASIA**		
Australia	Bangladesh	Bhutan
Brunei Darussalam	Cambodia	Fiji
Indonesia	Japan	Korea, Dem. People's Rep.
Korea, Rep.	Lao PDR	Malaysia
Maldives	Myanmar	Nepal
New Zealand	Papua New Guinea	Philippines
Singapore	Solomon Islands	Sri Lanka
Thailand	Timor-Leste	Vanuatu

Vietnam

NOTES:

Countries were included in a CIH region if they were United Nations Member States with populations of at least 300 000 in 2023. Statistics for China are based on jurisdictions under the direct governance of the People's Republic of China. For the CIH World region, if an input dataset contained a World region, those values were used for the CIH World region; if a dataset did not contain a World region, values for the CIH World region were calculated from all locations with available data, regardless of UN Member State status or population size.

TABLE A.2

Basic statistics for CIH regions, 2023

	Population (millions)	Population 70+ (% of total)	Births (thousands)	Deaths (thousands)	TFR[a]	Life expectancy (years)	PPD[b] (%)	GNI[c] per person (2021 int'l $ PPP[d])
Central Asia	384	2.5	10 500	2420	3.6	68.7	37.9	7340
Central and Eastern Europe	320	11.9	2710	3940	1.4	74.9	31.3	35 200
China	1420	8.9	8900	11 700	1.0	78.0	20.9	21 900
India	1440	4.1	23 200	9510	2.0	72.0	34.9	9050
Latin America and Caribbean	653	6.0	9300	4290	1.8	75.6	26.4	18 700
Middle East and North Africa	589	3.7	10 900	2880	2.4	75.2	25.5	22 200
North Atlantic	471	15.5	4050	4730	1.4	82.5	14.5	57 400
Sub-Saharan Africa	1260	1.8	41 800	10 200	4.4	62.2	49.8	4280
United States	343	11.8	3660	2980	1.6	79.3	21.6	74 500
Western Pacific and Southeast Asia	1160	7.2	16 600	8670	1.8	75.3	28.1	19 700
World	8090	6.4	132 000	61 700	2.2	73.2	30.1	20 400

NOTES:

a. Total fertility rate, live births per woman
b. Probability of premature death, dying before age 70
c. Gross national income
d. Purchasing power parity

DATA SOURCE:

Data from United Nations, Department of Economic and Social Affairs, Population Division. World Population Prospects 2024, Online Edition. 2024.

https://population.un.org/wpp/Download/Standard/MostUsed/(accessed Aug 26, 2024), World Development Indicators | DataBank [Internet].

Available from: https://databank.worldbank.org/source/world-development-indicators (accessed August 26, 2024).

TABLE A.3

Measures of progress in age-specific mortality rates, life expectancy at birth, and probability of premature death

Region	Year	Mortality rate		Life expectancy at birth (LE)		Probability of premature death (PPD)		Ratio of relative difference in PPD and LE
		Age-weighted mortality rate (per 10,000)[a]	Absolute and relative difference[b]	LE (year)	Absolute and relative difference[b]	PPD (%)	Absolute and relative difference[b]	
Sub-Saharan Africa	2000	143	-55 (-39%)	51.2	9.5 (18%)	66	-13 (-20%)	1.1
	2019	88		60.7		52		
India	2000	86	-20 (-23%)	62.7	8.0 (13%)	49	-12 (-24%)	1.9
	2019	67		70.7		37		
World	2000	85	-9.9 (-12%)	66.4	6.2 (9%)	41	- 10 (-24%)	2.6
	2019	75		72.6		31		
North Atlantic	2000	95	+0.8 (+1%)	78.6	3.6 (5%)	21	-6 (-27%)	5.9
	2019	96		82.4		15		

NOTES:

a. Weighted average of the age-specific mortality rates where the weights are the proportions of population in each age group.
b. Relative difference expressed as percentage of initial level.

SOURCE:

United Nations, Department of Economic and Social Affairs, Population Division. World Population Prospects 2024, Online Edition. 2024. https://population.un.org/wpp/Download/Standard/MostUsed/
Accessed July 14, 2024

TABLE A.4

Basic statistics for the 30 most populous countries in 2023

	Population (millions)	Population 70+ (% of total)	Births (thousands)	Deaths (thousands)	TFRa	Life expectancy (years	PPDb (%)	GNI per personc (2021 international $, PPP)
Bangladesh	171	3.9	3490	859	2.2	74.7	27.9	8500
Brazil	211	6.7	2600	1490	1.6	75.8	25.6	18 000
China	1420	8.9	8900	11 700	1.0	78.0	20.9	21 900
Colombia	52	5.8	705	282	1.6	77.7	20.2	18 700
Congo, Dem. Rep.	106	1.8	4370	902	6.0	61.9	48.7	1430
Egypt, Arab Rep.	115	2.7	2410	625	2.8	71.6	35.0	16 200
Ethiopia	129	1.9	4110	767	4.0	67.3	39.7	2800
France	66	15.9	639	616	1.6	83.3	14.8	55 400
Germany	84	16.6	719	1030	1.4	81.4	16.5	64 100
India	1440	4.1	23 200	9510	2.0	72.0	34.9	9050
Indonesia	281	4.1	4480	2120	2.1	71.1	35.8	13 700
Iran, Islamic Rep.	91	4.9	1170	423	1.7	77.7	18.5	16 100
Italy	60	18.1	385	663	1.2	83.7	11.6	52 400
Japan	124	23.6	750	1520	1.2	84.7	11.6	47 800
Kenya	55	1.7	1500	399	3.2	63.6	53.3	5610
Korea, Rep.	52	12.0	236	346	0.7	84.3	10.2	49 700
Mexico	130	5.1	2040	799	1.9	75.1	28.6	21 800
Myanmar	54	4.0	904	495	2.1	66.9	42.9	5230
Nigeria	228	1.7	7510	2680	4.5	54.5	61.1	5580
Pakistan	248	2.4	6880	1600	3.6	67.6	39.2	5500
Philippines	115	2.9	1840	716	1.9	69.8	38.3	10 700
Russian Federation	145	10.6	1300	1790	1.4	73.2	35.3	39 200
South Africa	63	3.9	1190	584	2.2	66.1	47.5	13 700
Sudan	50	1.7	1680	320	4.3	66.3	41.2	2810
Tanzania	67	1.8	2350	386	4.6	67.0	45.4	3520
Thailand	72	9.5	591	637	1.2	76.4	27.2	20 400
Türkiye	87	6.3	1070	552	1.6	77.2	22.6	33 900
United Kingdom	69	14.0	688	654	1.6	81.3	16.3	54 400
United States	343	11.8	3660	2980	1.6	79.3	21.6	74 500
Vietnam	100	5.1	1390	660	1.9	74.6	27.1	13 000

NOTES:

a. Total fertility rate, live births per woman
b. Probability of premature death, dying before age 70
c. Gross national income per person, constant 2021 international dollars at purchasing power parity (PPP)

Data source: United Nations, Population Division "World Population Prospects 2024" (population, population 70+, births, deaths, TFR, life expectancy); authors' calculations using United Nations, Population Division "World Population Prospects 2024" (PPD); World Bank "World Development Indicators", 2024 (GNI per person).

TABLE A.5

The probability of premature death (PPD) relative to income, world's 30-most populous countries

	Country Rank			Level of Indicator	
	PPD (2019)	PPD relative to income (2019)	PPD AARC (2010-2019)	PPD (2019)	PPD AARC (2010-2019)
World	-	-	-	31	1.3
Bangladesh	16	6	2	32	2.7
Brazil	13	5	11	26	1.6
China	8	3	10	21	1.8
Colombia	9	2	13	22	1.5
Congo, Democratic Rep. of	28	26	19	51	1.1
Egypt	17	14	12	36	1.6
Ethiopia	23	18	5	42	2.3
France	4	15	16	16	1.2
Germany	6	24	23	17	0.9
India	19	13	15	37	1.3
Indonesia	19	19	20	37	1
Iran, Islamic Rep. of	7	1	6	20	2.3
Italy	1	10	14	12	1.4
Japan	1	9	8	12	1.8
Kenya	29	28	25	55	0.6
Korea, Rep.	1	8	1	12	3.9
Mexico	15	12	27	29	0.4
Myanmar	25	22	17	44	1.2
Nigeria	30	30	29	63	0.3
Pakistan	22	17	22	41	0.9
Philippines	21	20	26	39	0.5
Russian Federation	17	25	3	36	2.6
South Africa	27	27	4	49	2.4
Sudan	23	21	18	42	1.2
Tanzania	26	23	9	47	1.8
Thailand	12	7	24	26	0.8
Türkiye	9	11	7	22	2.3
United Kingdom	4	16	21	16	1
United States	9	29	30	22	-0.1
Vietnam	14	4	28	28	0.4

NOTES:

PPD: The probability of premature death (PPD), defined as dying before the age of 70 at age-specific mortality rates for a baby born in the indicated year. PPD values were calculated from World Population Prospects (2024) life tables for the year 2019.

PPD to income: These results stem from a linear model relating PPD and 2019 gross national income (GNI) per capita in constant 2017 international dollars. The deviation from prediction indicates the disparity between the actual values and those predicted by the model.

PPD AARC: The average annual rate of change in PPD between 2010 and 2019.

COVID-19 P-score: P-scores are defined by dividing estimated excess deaths by the projected normal number of deaths in the same period. Excess deaths are from The Economist (downloaded from Our World in Data).

* Cumulative deaths for the period starting on January 1, 2020, and concludes on May 4, 2023. The World Health Organization (WHO) declared the end to the emergency phase of the COVID-19 pandemic on May 5, 2023.

Projected deaths were based on deaths in 2019 and the annual rate of change (averaged between 2015–2019) from United Nations, Department of Economic and Social Affairs, Population Division. World Population Prospects 2024, Online Edition. 2024. https://population.un.org/wpp/Download/Standard/MostUsed/ (accessed July 14, 2024).

TABLE A.6

The probability of premature death (PPD) in in 1970 and 2019, PPD progress between 2010-2019, and income in 2019 for 105 countries with a population greater than 5 million in 2023.

	PPD		Time required to halve PPDa	Income[b]
	1970 (%)	(2019) (%)	(years)	2019 (2021 international $, PPP)
World	56	31	55	19 000
Afghanistan	82	49	>75	3000 c
Algeria	74	23	50	15 000
Angola	77	49	42	7500
Argentina	43	25	>75	26 000
Australia	38	13	74	54 000
Austria	37	15	38	66 000
Azerbaijan	57	30	36	20 000 c
Bangladesh	72	32	26	7100
Belarus	32	33	26	26 000
Belgium	36	16	35	63 000
Benin	72	52	>75	3400
Bolivia	67	41	>75	9700
Brazil	56	26	43	17 000
Bulgaria	34	30	>75	27 000
Burkina Faso	76	51	64	2300
Burundi	72	49	44	890
Cambodia	83	35	52	4500
Cameroon	66	51	47	4700
Canada	33	15	>75	54 000
Central African Republic	72	93	>75	1200
Chad	75	63	>75	1900
Chile	50	19	53	27 000
China	60	21	38	18 000
Colombia	50	22	45	17 000
Congo, Dem. Rep.	73	51	62	1300
Congo, Rep.	63	52	>75	6300
Costa Rica	39	18	>75	22 000
Cuba	39	25	>75	-

TABLE A.6

The probability of premature death (PPD) in in 1970 and 2019, PPD progress between 2010-2019, and income in 2019 for 105 countries with a population greater than 5 million in 2023.

	PPD		Time required to halve PPDa	Income[b]
	1970 (%)	(2019) (%)	(years)	2019 (2021 international $, PPP)
Czech Republic	40	20	40	
Côte d'Ivoire	72	57	47	6200
Denmark	31	16	32	68 000
Dominican Republic	52	31	57	20 000
Ecuador	52	23	54	13 000
Egypt, Arab Rep.	63	36	43	15 000
El Salvador	67	35	>75	45 000
Ethiopia	75	42	30	2500
Finland	38	15	29	58 000
France	34	16	56	56 000
Germany	36	17	>75	65 000
Ghana	67	46	64	6200
Greece	30	16	>75	33 000
Guatemala	70	33	>75	12 000
Guinea	75	51	>75	3500
Haiti	67	47	13	3400
Honduras	61	34	45	5800
Hungary	38	28	50	37 000
India	68	37	54	7900
Indonesia	61	37	70	13 000
Iran, Islamic Rep.	65	20	30	14 000
Iraq	50	34	35	15 000
Ireland	37	14	32	76 000
Israel	36	13	54	44 000
Italy	33	12	48	51 000
Japan	33	12	38	47 000
Jordan	54	22	24	9200 c
Kazakhstan	53	32	20	30 000
Kenya	60	55	>75	5100
Korea, Dem. People's Rep.	50	30	31	-
Korea, Rep.	52	12	18	47 000

TABLE A.6

The probability of premature death (PPD) in in 1970 and 2019, PPD progress between 2010-2019, and income in 2019 for 105 countries with a population greater than 5 million in 2023.

	PPD		Time required to halve PPDa	Income[b]
	1970 (%)	(2019) (%)	(years)	2019 (2021 international $, PPP)
Kyrgyz Republic	56	37	29	5700
Lao PDR	74	40	37	7600 c
Lebanon	43	19	>75	15 000
Liberia	76	53	>75	1500 c
Libya	57	32	>75	18 000
Madagascar	69	46	>75	1700
Malawi	77	49	27	1700 c
Malaysia	53	27	>75	31 000
Mali	82	52	>75	2400
Mauritania	64	41	>75	6200
Mexico	56	29	>75	22 000
Morocco	68	28	28	8600
Mozambique	72	53	32	1500
Myanmar	66	44	58	6 400 c
Nepal	73	38	59	4600
Netherlands	30	14	42	66 000
New Zealand	37	15	>75	46 000
Nicaragua	60	30	49	6300
Niger	81	49	65	1600 c
Nigeria	76	63	>75	5700 c
Norway	29	12	26	84 000
Oman	69	16	20	36 000
Pakistan	60	41	>75	5400
Papua New Guinea	66	47	>75	4100 c
Paraguay	50	30	>75	15 000
Peru	68	25	25	15 000
Philippines	52	39	>75	10 000
Poland	37	25	62	37 000
Portugal	38	16	46	39 000
Romania	38	29	>75	36 000
Russian Federation	40	36	26	37 000

TABLE A.6

The probability of premature death (PPD) in in 1970 and 2019, PPD progress between 2010-2019, and income in 2019 for 105 countries with a population greater than 5 million in 2023.

	PPD		Time required to halve PPDa	Income[b]
	1970 (%)	(2019) (%)	(years)	2019 (2021 international $, PPP)
Rwanda	67	44	49	2600
Saudi Arabia	61	20	20	48 000 c
Senegal	75	39	47	4100
Serbia	37	27	64	20 000
Sierra Leone	75	52	31	1600
Singapore	43	12	25	100 000 c
Slovak Republic	38	24	42	37 000
Somalia	70	55	33	1500 c
South Africa	60	49	29	14 000
South Sudan	93	54	>75	-
Spain	31	13	51	46 000
Sri Lanka	46	24	38	14 000
Sudan	62	42	59	3600
Sweden	27	12	36	63 000
Switzerland	30	12	33	78 000
Syrian Arab Republic	51	34	>75	2900 c
Tajikistan	54	35	41	4400
Tanzania	69	47	38	3400
Thailand	61	26	>75	21 000 c
Togo	68	51	55	2600
Tunisia	60	24	>75	13 000
Turkmenistan	58	38	>75	-
Türkiye	53	22	30	28 000 c
Uganda	65	48	32	2600
Ukraine	36	33	34	18 000
United Arab Emirates	51	10	>75	71 000 c
United Kingdom	35	16	72	55 000 c
United States	38	22	>75	70 000
Uzbekistan	51	35	64	7800 c
Venezuela, RB	46	31	>75	-
Vietnam	60	28	>75	11 000

TABLE A.6

The probability of premature death (PPD) in in 1970 and 2019, PPD progress between 2010-2019, and income in 2019 for 105 countries with a population greater than 5 million in 2023.

	PPD		Time required to halve PPDa	Income[b]
	1970 (%)	(2019) (%)	(years)	2019 (2021 international $, PPP)
Rwanda	74	42	>75	-
Saudi Arabia	57	50	28	3300 [c]
Senegal	55	57	26	3400

NOTES:

a Time required to halve the probability of premature death at the rate of improvement between 2010 and 2019.

b Income is expressed as gross national income (GNI) per person in constant 2021 international dollars at purchasing power parity (PPP).

c Extrapolated using annual percent change of gross domestic product (GDP) per person.

- Data unavailable

DATA SOURCE:

United Nations, Population Division “World Population Prospects 2024” (GNI per person); authors’ calculations using United Nations, Population Division “World Population Prospects 2024” (PPD, halving time).

TABLE A.7

Probability of premature death in 2019 and year to achieve 30% reduction in PPD by 2035 and 50% reduction by 2050, both sexes combined

	PPD, 2019 (%)	Year to achieve target reduction in PPD	
		30%	50%
**CENTRAL ASIA**			
Kazakhstan	32%	2029	2038
Kyrgyzstan	37	2034	2048
**CENTRAL AND EASTERN EUROPE**			
Belarus	33	2032	2044
Estonia	23	2034	2049
Lithuania	28	2033	2047
Russian Federationa	36	2032	2045
**LATIN AMERICA AND CARIBBEAN**			
Haiti	47	2025	2032
Peru	25	2032	2044
**MIDDLE EAST AND NORTH AFRICA**			
Irana	20	2034	2048
Jordan	22	2031	2043
Kuwait	11	2025	2030
Morocco	28	2033	2046
Oman	16	2029	2038
Qatar	10	2026	2032
Saudi Arabia	20	2029	2038
State of Palestine	24	2035	2050
Türkiyea	22	2034	2048
**NORTH ATLANTIC**			
Finland	15	2034	2048
Norway	12	2032	2044
**SUB-SAHARAN AFRICA**			
Botswana	44	2029	2038
Eswatini	61	2030	2040
Ethiopiaa	42	2034	2048
Lesotho	69	2034	2049
Malawi	49	2033	2046
Sierra Leone	52	2035	2049
South Africaa	49	2033	2047
Zambia	50	2033	2046

TABLE A.7

Probability of premature death in 2019 and year to achieve 30% reduction in PPD by 2035 and 50% reduction by 2050, both sexes combined

	PPD, 2019 (%)	Year to achieve target reduction in PPD	
		30%	50%
Zimbabwe	57	2032	2044
Botswana	44	2029	2038
**WESTERN PACIFIC AND SOUTHEAST ASIA**			
Bangladesha	32	2032	2044
Dem. People's Republic of Korea	30	2035	2049
Maldives	15	2029	2037
Republic of Koreaa	12	2028	2036

NOTES:

a = among the 30 most populous countries in the world. Countries projected to achieve a 50% reduction in PPD, based on the rate of decline from 2010 to 2019, by 2050 are categorized by CIH region. Countries that are not on track to meet their targets by 2035 or 2050 have been excluded from the list. This analysis considers only countries with a population exceeding 5 million.

Source from Norheim OF. Halving premature death and improving the quality of life at all ages. Background paper for CIH 3.0 [Internet]. 2024. Available from: https://www.uib.no/sites/w3.uib.no/files/attachments/norheim_et_al_2024_cih3.pdf (accessed September 19, 2024).

TABLE A.8

Changes in gap in life expectancy compared to the North Atlantic in 2019 attributable to I-8

Region	(1) Gap in 2000 (years)	(2) Amount of gap in 2000 due to I-8 (years)	(3) Amount of gap due toI-8 eliminated 2000–2019 (years)	(4) Amount of gap remaining in 2019 due to I-8 (years)	(5) Percent of gap remaining in 2019 due to I-8
World	16	7.2	3.8	3.3	35%
Central & Eastern Europe	14	1.1	0.6	0.5	7.0%
Central Asia	21	8.4	4.2	4.2	29%
China	10.0	1.7	1.6	0.2	3.9%
India	20	10.8	7.4	3.4	29%
Latin America & Caribbean	11	2.5	1.1	1.4	20%
Middle East & North Africa	13	2.6	1.6	1.0	13%
North Atlantic	3.6	0.2	0.2	0.0	
Sub-Saharan Africa	31	20.7	9.8	10.9	50%
United States	5.4	0.3	0.2	0.1	3.2%
Western Pacific & Southeast Asia	13	5.3	3.2	2.1	28%

NOTES:

Life expectancy in the North Atlantic was 82 years in 2019. Pollard's decomposition method was used (see Pollard JH. On the decomposition of changes in expectation of life and differentials in life expectancy. Demography 1988; 25: 265–76).

The priority infections and maternal health conditions (I-8) are neonatal conditions, lower respiratory infections, diarrheal diseases, HIV/AIDS, tuberculosis, malaria, childhood-cluster diseases, and maternal conditions.

Adapted from CIH background paper Karlsson O, Chang AY, Norheim OF, Mao W, Jamison DT. Priority health conditions and life expectancy disparities. Available from: https://www.uib.no/sites/w3.uib.no/files/attachments/karlsson_et_al._priority_health_conditions.pdf (September 19, 2024)

Data from United Nations, Department of Economic and Social Affairs, Population Division. World Population Prospects 2024, Online Edition. 2024. https://population.un.org/wpp/Download/Standard/MostUsed/ (accessed July 14, 2024) and World Health Organization. Global Health Estimates, 2000-2021. Geneva: Department of Data and Analytics (DNA) Division of Data, Analytics and Delivery for Impact (DDI) WHO, 2024.

TABLE A.9

Changes in gap in life expectancy compared to the North Atlantic in 2019 attributable to NCD-7

Region	(1) Gap in 2000 (years)	(2) Amount of gap in 2000 due to I-8 (years)	(3) Amount of gap due toI-8 eliminated 2000–2019 (years)	(4) Amount of gap remaining in 2019 due to I-8 (years)	(5) Percent of gap remaining in 2019 due to I-8
World	16	5.7	1.5	4.2	43%
Central & Eastern Europe	14	9.2	4.0	5.2	68%
Central Asia	21	8.1	1.0	7.1	48%
China	10.0	6.3	2.8	3.5	82%
India	20	5.2	-0.4	5.6	49%
Latin America & Caribbean	11	4.8	2.0	2.8	40%
Middle East & North Africa	13	7.1	2.5	4.6	60%
North Atlantic	3.6	2.5	2.5	0.0	
Sub-Saharan Africa	31	4.4	-0.6	5.0	23%
United States	5.4	3.6	2.1	1.5	44%
Western Pacific & Southeast Asia	13	5.0	1.3	3.7	50%

NOTES:

Life expectancy in the North Atlantic was 82 years in 2019. Pollard's decomposition method was used (see Pollard JH. On the decomposition of changes in expectation of life and differentials in life expectancy. Demography 1988; 25: 265–76).

The priority infections and maternal health conditions (I-8) are neonatal conditions, lower respiratory infections, diarrheal diseases, HIV/AIDS, tuberculosis, malaria, childhood-cluster diseases, and maternal conditions.

Adapted from CIH background paper Karlsson O, Chang AY, Norheim OF, Mao W, Jamison DT. Priority health conditions and life expectancy disparities. Available from: https://www.uib.no/sites/w3.uib.no/files/attachments/karlsson_et_al._priority_health_conditions.pdf (September 19, 2024)

Data from United Nations, Department of Economic and Social Affairs, Population Division. World Population Prospects 2024, Online Edition. 2024. https://population.un.org/wpp/Download/Standard/MostUsed/ (accessed July 14, 2024) and World Health Organization. Global Health Estimates, 2000-2021. Geneva: Department of Data and Analytics (DNA) Division of Data, Analytics and Delivery for Impact (DDI) WHO, 2024.

TABLE A.10

Progress against tuberculosis in the 30 countries with highest number of deaths, 2000–2010, 2010–2019 and 2019–2021

	Deaths (thousands)			Death rate (per 100,000 population)			Average annual rate of change in death rate, %		
	2000	2019	2021	2000	2019	2021	2000–10	2010–19	2019–21
World	2,500	1,300	1,400	41	17	18	-3.9%	-5.2%	1.6%
Afghanistan	14	9.9	10	68	26	25	-4.4%	-5.5%	-1.8%
Angola	14	20	21	89	63	61	2.1%	-6.0%	-1.8%
Bangladesh	93	39	43	69	24	26	-3.0%	-8.2%	4.1%
Brazil	8.5	7.0	11	4.9	3.4	5.1	-1.6%	-2.3%	23%
Cameroon	25	12	12	170	48	44	-6.6%	-6.1%	-3.8%
Central African Rep.	11	7.6	8.3	300	150	160	-2.3%	-4.7%	2.9%
China	110	38	36	8.4	2.7	2.5	-7.0%	-4.6%	-2.6%
Congo DR	57	57	51	110	61	52	-3.0%	-3.4%	-8.0%
Côte d’Ivoire	24	8.7	8.5	140	31	29	-11%	-4.0%	-3.9%
Ethiopia	110	25	21	160	22	17	-11%	-9.5%	-10%
Ghana	18	16	16	91	51	49	-2.9%	-3.1%	-2.4%
India	910	330	360	86	24	25	-6.0%	-7.0%	2.9%
Indonesia	120	96	140	56	35	49	-1.3%	-3.5%	18%
Kenya	49	32	32	160	62	60	-0.89%	-9.1%	-1.6%
Madagascar	14	13	13	84	45	45	-3.8%	-2.7%	-0.056%
Mozambique	24	11	13	130	37	42	-1.5%	-12%	6.4%
Myanmar	88	22	37	200	42	69	-5.5%	-10%	29%
Nepal	27	17	18	110	59	61	-2.8%	-3.7%	1.6%
Nigeria	130	160	130	100	76	58	-1.5%	-1.5%	-13%
North Korea	38	19	23	160	71	86	-6.2%	-1.8%	9.9%
Pakistan	58	46	53	37	20	22	-3.3%	-3.1%	5.2%
Philippines	29	28	39	36	25	34	-3.1%	-0.40%	16%
Russia	32	9.6	10	22	6.6	7.1	-2.6%	-9.9%	4.4%
Somalia	8.4	11	12	95	67	67	-0.45%	-3.3%	0.036%
South Africa	100	60	59	220	100	95	0.11%	-8.3%	-3.0%
Tanzania	58	32	25	170	54	41	-2.4%	-9.5%	-13%
Thailand	40	11	10	64	15	14	-9.6%	-4.5%	-3.3%
Uganda	27	17	14	110	39	30	-5.7%	-4.9%	-12%
Vietnam	30	11	11	39	11	11	-4.9%	-8.1%	-0.67%
Zambia	22	15	7.9	220	80	40	-5.2%	-5.1%	-29%

NOTES:

Numbers are rounded. Data from World Health Organization. Global Health Estimates, 2000-2021. Geneva: Department of Data and Analytics (DNA) Division of Data, Analytics and Delivery for Impact (DDI) WHO, 2024.

TABLE A.11

Progress against HIV/AIDS in the 30 countries with highest number of deaths, 2000–2010, 2010–2019 and 2019–2021

	Deaths (thousands)			Death rate (per 100,000 population)			Average annual rate of change in death rate, %		
	2000	2019	2021	2000	2019	2021	2000–10	2010–19	2019–21
World	1,600	720	650	27	9.2	8.1	-3.9%	-7.1%	-5.8%
Angola	8.0	17	14	49	52	42	3.2%	-2.9%	-10%
Brazil	13	12	12	7.6	5.6	5.9	-0.81%	-2.4%	2.1%
Cameroon	22	15	11	150	58	43	-1.3%	-8.7%	-14%
Central African Rep.	12	5.1	4.4	330	100	86	-4.6%	-7.3%	-8.4%
China	28	37	38	2.2	2.6	2.7	6.2%	-4.6%	0.97%
Congo DR	53	21	14	110	22	14	-4.7%	-11%	-20%
Congo, Rep.	6.1	7.2	7.6	200	130	130	-5.1%	1.1%	0.96%
Côte d'Ivoire	55	14	12	310	50	39	-6.9%	-12%	-12%
Ethiopia	70	15	12	100	13	10	-12%	-9.0%	-11%
Ghana	21	16	11	110	50	33	-3.3%	-4.8%	-18%
India	220	45	45	21	3.2	3.2	-4.3%	-15%	-0.89%
Indonesia	1.2	28	24	0.56	10	8.6	28%	4.9%	-7.9%
Kenya	110	20	21	370	38	40	-11%	-12%	2.5%
Malawi	71	14	13	630	73	63	-9.7%	-12%	-7.2%
Mali	7.8	5.6	5.1	67	27	23	-5.8%	-3.6%	-7.3%
Mexico	4.4	5.3	4.6	4.4	4.2	3.6	-0.14%	-0.42%	-7.0%
Mozambique	49	47	48	270	160	150	1.3%	-7.0%	-1.7%
Myanmar	5.8	7.0	5.9	13	13	11	5.7%	-5.6%	-9.0%
Nigeria	100	56	43	80	27	20	-6.7%	-4.3%	-14%
Pakistan	0.02	8.9	12	0.01	3.8	4.8	58%	16%	12%
Russia	8.7	28	31	5.9	19	21	4.8%	8.0%	6.4%
South Africa	140	53	48	300	89	78	-0.57%	-12%	-6.3%
South Sudan	6.7	9.1	8.3	110	87	76	2.0%	-4.8%	-6.6%
Tanzania	99	30	24	290	50	39	-7.2%	-11%	-13%
Thailand	54	15	12	86	21	17	-6.2%	-8.2%	-10%
Uganda	83	19	17	350	44	38	-8.1%	-13%	-6.7%
United States	15	5.2	5.1	5.4	1.6	1.5	-6.5%	-6.2%	-1.5%
Vietnam	5.0	5.0	4.3	6.5	5.1	4.4	0.65%	-3.2%	-7.9%
Zambia	58	25	20	580	130	100	-9.4%	-5.2%	-12%
Zimbabwe	100	23	21	880	150	130	-6.8%	-11%	-7.8%

NOTES:

Numbers are rounded. Data from World Health Organization. Global Health Estimates, 2000-2021. Geneva: Department of Data and Analytics (DNA) Division of Data, Analytics and Delivery for Impact (DDI) WHO, 2024.

TABLE A.12

Progress against malaria in the 30 countries with highest number of deaths, 2000–2010, 2010–2019 and 2019–2021

	Deaths (thousands)			Death rate (per 100,000 population)			Average annual rate of change in death rate, %		
	2000	2019	2021	2000	2019	2021	2000–10	2010–19	2019–21
World	870	580	600	14	7.4	7.6	-3.2%	-3.5%	1.3%
Angola	22	16	19	140	48	56	-9.1%	-0.86%	8.0%
Benin	6.7	11	11	92	86	83	0.73%	-1.7%	-1.7%
Burkina Faso	38	16	16	320	77	73	-2.9%	-12%	-2.6%
Burundi	12	6.6	7.4	190	54	57	-12%	0.67%	2.7%
Cameroon	18	11	13	120	44	47	-6.2%	-3.9%	3.2%
Central African Rep.	4.0	3.9	5.0	100	79	98	1.4%	-4.5%	11%
Chad	10	14	14	120	82	79	-0.59%	-3.4%	-1.5%
Congo DR	98	78	73	190	84	74	-4.1%	-4.6%	-6.3%
Côte d'Ivoire	30	12	12	170	44	41	-1.8%	-12%	-4.0%
Ethiopia	25	5.8	8.2	37	5.0	6.7	-5.3%	-15%	16%
Ghana	20	11	11	100	36	35	-4.1%	-6.7%	-0.84%
Guinea	15	11	11	170	85	77	-2.6%	-5.0%	-4.7%
India	30	7.7	8.4	2.8	0.56	0.59	-1.3%	-15%	3.1%
Kenya	12	11	12	39	22	22	-6.4%	0.80%	1.1%
Liberia	6.0	4.5	4.1	210	89	78	-9.8%	2.1%	-6.2%
Madagascar	2.3	5.1	13	14	18	44	-3.1%	6.7%	56%
Malawi	19	6.9	7.5	170	36	37	-8.4%	-7.2%	1.5%
Mali	23	20	20	200	96	89	-4.4%	-3.2%	-4.1%
Mozambique	40	19	22	220	63	71	-7.3%	-5.3%	5.7%
Niger	23	29	30	200	130	120	-2.6%	-2.2%	-1.6%
Nigeria	240	180	180	190	87	84	-3.9%	-4.1%	-1.4%
Rwanda	6.3	3.2	3.4	77	25	25	-9.2%	-1.6%	-0.21%
Sierra Leone	12	7.7	8.2	280	100	100	-2.2%	-8.6%	0.78%
South Sudan	11	7.4	6.7	190	71	61	-9.1%	-0.019%	-7.1%
Sudan	5.6	6.9	8.1	20	15	17	-8.9%	7.5%	5.4%
Tanzania	41	24	26	120	40	41	-9.6%	-0.96%	0.82%
Togo	5.0	3.4	3.6	96	41	40	-4.5%	-4.4%	-0.64%
Uganda	42	16	18	170	37	39	-7.6%	-8.1%	1.6%
Yemen	2.8	2.4	2.8	14	6.8	7.6	-2.1%	-5.7%	5.9%
Zambia	15	7.9	8.8	150	43	45	-11%	-0.46%	2.4%

NOTES:

Numbers are rounded. Data from World Health Organization. Global Health Estimates, 2000-2021. Geneva: Department of Data and Analytics (DNA) Division of Data, Analytics and Delivery for Impact (DDI) WHO, 2024.

TABLE A.13

Progress against malaria in the 30 countries with highest number of deaths, 2000–2010, 2010–2019 and 2019–2021

	Deaths (thousands)			Death rate (per 100,000 population)			Average annual rate of change in death rate, %		
	2000	2019	2021	2000	2019	2021	2000–10	2010–19	2019–21
World	410	240	260	300	170	190	-3.3%	-2.4%	5.8%
Afghanistan	9.5	11	12	910	790	820	0.73%	-2.4%	2.1%
Angola	4.1	3.1	3.4	540	240	260	-5.5%	-2.6%	3.0%
Bangladesh	14	4.3	3.9	350	130	120	-4.5%	-5.8%	-5.4%
Brazil	1.8	1.8	3.1	53	66	120	1.2%	1.1%	32%
Cameroon	4.1	3.5	3.7	670	380	400	-2.4%	-3.6%	1.7%
Chad	4.0	5.4	5.2	910	720	670	-1.4%	-0.90%	-3.7%
China	9.7	2.6	2.5	55	18	24	-5.7%	-5.7%	14%
Congo DR	15	15	16	670	390	400	-2.1%	-3.6%	0.64%
Côte d’Ivoire	3.9	2.5	2.5	500	260	260	-0.95%	-6.0%	-0.046%
Ethiopia	29	10.0	9.5	930	260	240	-5.9%	-7.3%	-3.7%
Guinea	2.7	2.2	2.3	760	460	470	-1.4%	-3.7%	0.65%
India	110	26	30	380	110	130	-7.1%	-5.8%	9.0%
Indonesia	13	5.8	7.4	280	130	160	-4.3%	-4.1%	13%
Kenya	8.8	4.5	3.5	710	310	240	-3.7%	-4.8%	-13%
Madagascar	3.2	3.7	3.9	460	390	400	-0.75%	-1.2%	1.3%
Malawi	3.4	2.6	3.1	670	420	480	-3.1%	-1.6%	7.4%
Mali	3.6	3.4	3.5	660	390	390	-2.7%	-2.6%	-0.63%
Mozambique	3.3	2.8	2.9	410	240	240	-1.2%	-4.5%	-1.0%
Niger	4.0	4.4	4.8	660	440	470	-2.0%	-2.2%	3.2%
Nigeria	45	45	49	810	620	670	-1.6%	-1.2%	4.0%
Pakistan	24	15	15	420	230	230	-3.3%	-3.0%	-0.76%
Philippines	2.5	2.4	3.0	110	120	160	-0.77%	2.1%	16%
Somalia	4.2	3.8	4.6	940	530	600	-1.1%	-5.0%	6.5%
South Africa	1.7	1.7	2.1	160	140	170	4.7%	-6.4%	11%
South Sudan	2.3	2.6	2.7	780	860	850	-0.52%	1.7%	-0.52%
Sudan	5.6	4.4	5.0	490	270	310	-4.0%	-1.9%	6.2%
Tanzania	11	6.8	6.4	770	310	280	-5.0%	-4.2%	-4.5%
Uganda	6.1	3.8	4.3	530	240	250	-4.7%	-3.3%	3.0%
Yemen	2.2	1.9	2.1	270	150	160	-4.2%	-1.6%	2.1%
Zimbabwe	1.7	2.1	2.2	400	430	440	3.0%	-2.2%	0.94%

NOTES:

Numbers are rounded. Data on deaths from World Health Organization. Global Health Estimates, 2000-2021. Geneva: Department of Data and Analytics (DNA) Division of Data, Analytics and Delivery for Impact (DDI) WHO,

2024 and live births from WPP24 United Nations, Department of Economic and Social Affairs, Population Division. World Population Prospects 2024, Online Edition. 2024. https://population.un.org/wpp/Download/Standard/MostUsed/ (accessed July 14, 2024).

TABLE A.14

Progress against under-15 deaths in the 30 countries with highest number of deaths, 2000–2010, 2010–2019 and 2019–2021

	Deaths (thousands)			Death rate (per 100,000 population)			Average annual rate of change in death rate, %		
	2000	2019	2021	2000	2019	2021	2000–10	2010–19	2019–21
World	12,000	6,700	6,300	88	47	46	-3.5%	-3.0%	-1.8%
Afghanistan	150	100	100	150	75	72	-3.7%	-3.7%	-1.8%
Angola	170	110	100	240	87	80	-5.4%	-5.2%	-3.7%
Bangladesh	400	130	120	100	39	35	-5.1%	-4.8%	-5.2%
Benin	47	51	50	160	120	110	-1.8%	-1.8%	-2.2%
Burkina Faso	110	78	72	210	110	100	-3.4%	-3.4%	-3.4%
Cameroon	99	81	78	170	92	86	-2.6%	-3.9%	-3.4%
Chad	94	110	110	240	160	150	-1.6%	-2.2%	-3.1%
China	830	180	130	44	11	9.7	-6.9%	-7.1%	-6.7%
Congo DR	420	400	400	200	110	100	-2.9%	-3.1%	-3.4%
Côte d'Ivoire	120	84	80	160	89	83	-3.1%	-3.3%	-3.3%
Egypt, Arab Rep.	100	59	54	53	24	22	-4.5%	-3.5%	-3.8%
Ethiopia	550	260	240	190	70	65	-5.2%	-5.1%	-4.0%
Ghana	87	60	57	130	70	66	-2.9%	-3.6%	-3.1%
Guinea	68	58	57	200	130	120	-2.6%	-2.0%	-2.4%
India	3,000	970	840	110	39	35	-4.5%	-5.7%	-5.6%
Indonesia	290	130	120	61	29	27	-4.0%	-3.7%	-3.4%
Kenya	130	75	85	110	52	59	-5.4%	-2.4%	6.0%
Madagascar	89	78	81	140	88	87	-3.6%	-0.87%	-0.20%
Mali	110	100	100	220	120	120	-3.1%	-2.7%	-2.9%
Mozambique	150	95	92	200	85	79	-4.9%	-4.0%	-3.4%
Niger	140	130	130	250	140	130	-4.6%	-1.7%	-1.3%
Nigeria	1,200	1,200	1,100	240	170	160	-2.4%	-1.3%	-2.3%
Pakistan	650	490	470	120	74	69	-2.1%	-2.8%	-3.0%
Philippines	100	71	65	44	33	33	-2.1%	-0.74%	-0.60%
Somalia	87	98	97	210	150	140	0.75%	-4.9%	-3.4%
South Africa	83	49	50	78	42	42	-1.9%	-4.6%	-0.092%
Sudan	140	110	110	130	74	69	-3.0%	-2.9%	-3.2%
Tanzania	200	110	110	150	54	50	-6.0%	-4.6%	-3.8%
Uganda	190	82	80	170	53	50	-6.2%	-5.7%	-3.4%
Yemen	80	64	62	100	54	49	-4.7%	-1.8%	-4.3%

NOTES:

Numbers are rounded. The under-15 mortality rate is an approximate summary of the following priority conditions: neonatal conditions, diarrheal diseases, lower respiratory infections, and childhood-cluster diseases.

TABLE A.14

Progress against under-15 deaths in the 30 countries with highest number of deaths, 2000–2010, 2010–2019 and 2019–2021

Data from United Nations, Department of Economic and Social Affairs, Population Division. World Population Prospects 2024, Online Edition. 2024. https://population.un.org/wpp/Download/Standard/MostUsed/ (accessed July 14, 2024).

TABLE A.15

Changes in life expectancy attributable to priority conditions: 2000-2010, 2010-2019, 2019-2021 – World, Sub-Saharan Africa, India, China, North Atlantic

	WORLD			SUB-SAHARAN AFRICA			INDIA			CHINA			NORTH ATLANTIC		
	2000-2010	2010-2019	2019-2021	2000-2010	2010-2019	2019-2021	2000-2010	2010-2019	2019-2021	2000-2010	2010-2019	2019-2021	2000-2010	2010-2019	2019-2021
**Life expectancy in the earlier year**	66.4	70.1	72.6	51.2	56.7	60.7	62.7	67.2	70.7	72.3	75.7	77.9	78.6	81.0	82.2
**Total change, years**	3.7	2.5	-1.7	5.5	4.0	-0.42	4.4	3.6	-3.5	3.4	2.3	0.18	2.4	1.3	-0.39
**CHANGES ATTRIBUTABLE TO PRIORITY CONDITIONS, YEARS (%):**															
**TOTAL I-8**	2.2 (61)	1.4 (56)	0.19 (-11)	5.0 (90)	3.7 (94)	0.84 (-202)	3.9 (87)	3.0 (82)	0.33 (-10)	1.2 (36)	0.45 (20)	0.07 (38)	0.20 (9)	0.03 (3)	0.06 (-16)
Childhood-cluster diseases	0.33 (9)	0.10 (4)	0.07 (-4)	0.85 (15)	0.04 (1)	0.17 (-42)	0.37 (8)	0.28 (8)	0.06 (-2)	0.06 (2)	0.02 (1.0)	~0 (~0)	~0 (~0)	~0 (~0)	~0 (~0)
Diarrheal diseases	0.38 (10)	0.22 (9)	0.03 (-2)	0.57 (10)	0.43 (11)	0.08 (-18)	0.88 (20)	0.66 (18)	0.08 (-2)	0.12 (4)	0.02 (1)	~0 (2)	-0.01 (-0.4)	~0 (~0)	~0 (-1)
HIV/AIDS	0.23 (6)	0.24 (10)	0.03 (-2)	1.6 (29)	1.4 (35)	0.24 (-58)	0.19 (4)	0.25 (7)	~0 (-0.1)	-0.03 (-0.9)	0.03 (1)	~0 (-0.6)	0.02 (0.9)	0.02 (1)	~0 (-0.3)
Lower respiratory infections	0.33 (9)	0.19 (8)	0.05 (-3)	0.38 (7)	0.33 (8)	0.10 (-24)	0.39 (9)	0.38 (10)	0.07 (-2)	0.42 (12)	0.10 (4)	0.02 (10)	0.15 (6)	0.01 (0.7)	0.05 (-13)
Malaria	0.10 (3)	0.07 (3)	-0.02 (1)	0.62 (11)	0.32 (8)	-0.01 (3)	0.01 (0.1)	0.05 (1)	~0 (~0)	~0 (~0)	~0 (~0)	0 (0)	0 (0)	0 (0)	0 (0)
Maternal conditions	0.06 (2)	0.03 (1)	-0.01 (0.3)	0.16 (3)	0.18 (4)	0.01 (-2)	0.15 (3)	0.05 (1)	~0 (0.1)	0.01 (0.3)	0.01 (0.2)	~0 (0.1)	~0 (~0)	~0 (~0)	~0 (~0)
Neonatal conditions	0.47 (13)	0.29 (11)	0.03 (-2)	0.31 (6)	0.24 (6)	0.07 (-17)	0.86 (19)	0.68 (19)	0.13 (-4)	0.54 (16)	0.23 (10)	0.04 (24)	0.03 (1)	~0 (0.4)	0.01 (-2)

TABLE A.15

Changes in life expectancy attributable to priority conditions: 2000-2010, 2010-2019, 2019-2021 – World, Sub-Saharan Africa, India, China, North Atlantic

	WORLD			SUB-SAHARAN AFRICA			INDIA			CHINA			NORTH ATLANTIC		
	2000-2010	2010-2019	2019-2021	2000-2010	2010-2019	2019-2021	2000-2010	2010-2019	2019-2021	2000-2010	2010-2019	2019-2021	2000-2010	2010-2019	2019-2021
Tuberculosis	0.35 (9)	0.28 (11)	~0 (0.2)	0.51 (9)	0.82 (21)	0.19 (-46)	1.0 (23)	0.59 (16)	-0.01 (0.2)	0.11 (3)	0.03 (1)	~0 (2)	0.01 (0.3)	~0 (0.3)	~0 (~0)
**TOTAL NCD-7**	0.88 (24)	0.77 (30)	0.16 (-9)	0.01 (0.2)	0.04 (1)	0.03 (-7)	-0.05 (-1)	0.14 (4)	0.21 (-6)	1.3 (38)	1.2 (54)	0.09 (48)	1.7 (72)	1.0 (82)	0.19 (-47)
Atherosclerotic CVDs	0.30 (8)	0.30 (12)	0.05 (-3)	-0.03 (-0.5)	-0.01 (-0.2)	-0.02 (5)	-0.12 (-3)	-0.08 (-2)	0.08 (-2)	-0.23 (-7)	0.15 (7)	0.02 (9)	1.1 (45)	0.63 (50)	0.08 (-21)
Diabetes	-0.01 (-0.3)	-0.03 (-1)	-0.01 (0.5)	-0.04 (-0.8)	-0.02 (-0.5)	-0.02 (5)	-0.05 (-1)	-0.08 (-2)	~0 (-0.1)	0.02 (0.5)	0.02 (0.7)	~0 (0.6)	0.04 (2)	0.04 (3)	~0 (0.7)
Hemorrhagic stroke	0.16 (4)	0.15 (6)	0.02 (-1)	0.04 (0.6)	0.04 (1)	-0.01 (3)	0.04 (0.9)	0.02 (0.7)	0.02 (-0.6)	0.37 (11)	0.37 (16)	0.03 (16)	0.10 (4)	0.07 (6)	0.01 (-2)
Infection-related NCDs	0.13 (4)	0.11 (4)	0.02 (-1)	0.02 (0.4)	0.03 (0.6)	0.05 (-11)	0.07 (2)	0.11 (3)	~0 (-0.1)	0.26 (8)	0.19 (8)	0.02 (12)	0.10 (4)	0.06 (5)	0.01 (-2)
Road injury	0.05 (1)	0.07 (3)	0.02 (-1)	0.04 (0.8)	0.02 (0.4)	0.02 (-6)	0.02 (0.4)	0.07 (2)	0.03 (-1.0)	0.11 (3)	0.11 (5)	0.02 (9)	0.18 (7)	0.05 (4)	0.01 (-3)
Suicide	0.05 (1)	0.03 (1)	0.01 (-0.7)	-0.02 (-0.3)	-0.01 (-0.3)	~0 (1.0)	0.02 (0.4)	0.06 (2)	0.05 (-1)	0.14 (4)	0.05 (2)	~0 (2)	0.04 (2)	0.01 (1.0)	0.01 (-2)
Tobacco-related NCDs	0.20 (6)	0.13 (5)	0.04 (-2)	~0 (~0)	~0 (~0)	0.02 (-5)	-0.03 (-0.6)	0.04 (1)	0.01 (-0.4)	0.62 (18)	0.33 (14)	~0 (0.1)	0.18 (8)	0.17 (13)	0.07 (-18)
**TOTAL OTHER CAUSES**	0.55 (15)	0.34 (13)	-2.1 (120)	0.52 (9)	0.19 (5)	-1.3 (309)	0.62 (14)	0.52 (14)	-4.0 (116)	0.88 (26)	0.60 (27)	0.02 (14)	0.46 (19)	0.19 (15)	-0.64 (164)
COVID-19	0 (0)	0 (0)	-1.8 (103)	0 (0)	0 (0)	-1.1 (255)	0 (0)	0 (0)	-3.4 (98)	0 (0)	0 (0)	~0 (-0.5)	0 (0)	0 (0)	-0.79 (201)

TABLE A.15

Changes in life expectancy attributable to priority conditions: 2000-2010, 2010-2019, 2019-2021 – World, Sub-Saharan Africa, India, China, North Atlantic

	WORLD			SUB-SAHARAN AFRICA			INDIA			CHINA			NORTH ATLANTIC		
	2000-2010	2010-2019	2019-2021	2000-2010	2010-2019	2019-2021	2000-2010	2010-2019	2019-2021	2000-2010	2010-2019	2019-2021	2000-2010	2010-2019	2019-2021
Other COVID-19 pandemic-related outcomes	0 (0)	0 (0)	-0.39 (22)	0 (0)	0 (0)	-0.42 (100)	0 (0)	0 (0)	-0.77 (22)	0 (0)	0 (0)	-0.01 (-7)	0 (0)	0 (0)	~0 (0.1)

NOTES:

Changes in years of life expectancy attributable to specific causes of death are shown, with the percentage of total change attributable to each cause in parentheses below. Percentages of total change are negative if the impact of the specific cause was in the opposite direction of the total change (eg, if there was an increase in life expectancy, the increase would have been larger had it not been for rising mortality from that cause). Total impact of other causes includes all other causes of death while only the specific impact of COVID-19 related mortality is shown. Pollard's decomposition method was used (see POLLARD Pollard JH. On the decomposition of changes in expectation of life and differentials in life expectancy. Demography 1988; 25: 265–76.).

Data from United Nations, Department of Economic and Social Affairs, Population Division. World Population Prospects 2024, Online Edition. 2024. https://population.un.org/wpp/Download/Standard/MostUsed/ (accessed July 14, 2024) and World Health Organization. Global Health Estimates, 2000-2021. Geneva: Department of Data and Analytics (DNA) Division of Data, Analytics and Delivery for Impact (DDI) WHO, 2024.

TABLE A.16

Changes in deaths from selected NCD-7 conditions decomposed into changes in age group size and age group specific mortality rates, by CIH region, 2000-2010, 2010-2019, and 2000-2019.

Panel A: NCD-7

Panel B: Atherosclerotic CVD

Panel C: Hemorrhagic stroke

Panel A1: NCD-7 – World	Age group			
	15-49	50-69	70+	All ages
**POPULATION SIZE (MILLIONS)**				
2000	3 210	808	269	6 160
2010	3 670	1 080	355	7 010
2019	3 930	1 380	451	7 800
Rate of change in population size (% per year)				
2000-2010	1.3	2.9	2.8	1.3
2010-2019	0.8	2.8	2.7	1.2
2000-2019	1.1	2.9	2.8	1.2
**DEATHS (THOUSANDS)**				
2000	2 960	6 790	11 900	21 900
2010	3 060	7 340	14 300	24 900
2019	2 880	8 790	16 000	27 900
Rate of change in deaths (% per year)				
2000-2010	0.3	0.8	1.8	1.3
2010-2019	-0.7	2.0	1.3	1.3
2000-2019	-0.1	1.4	1.6	1.3
**DEATH RATE (PER 100 000 POPULATION PER YEAR)**				
2000	92.1	840	4 430	356
2010	83.4	683	4 030	355
2019	73.3	635	3 560	358
Rate of change in death rate (% per year)				
2000-2010	-1.0	-2.1	-0.9	0.0
2010-2019	-1.4	-0.8	-1.4	0.1
2000-2019	-1.2	-1.5	-1.1	0.0

DATA SOURCE:
World Health Organization (WHO). Global Health Estimates, 2000-2021. Geneva: Department of Data and Analytics (DNA) Division of Data, Analytics and Delivery for Impact (DDI), WHO, 2024.

NOTE:
Rates of change between any two years are assumed to be constant. Hence the rate of change in the number of deaths will be the sum of the rate of change in the population size and the rate of change in the death rate, except for rounding.

TABLE A.16

Changes in deaths from selected NCD-7 conditions decomposed into changes in age group size and age group specific mortality rates, by CIH region, 2000-2010, 2010-2019, and 2000-2019.

Panel A2: NCD-7 – Central Asia	Age group			
	15-49	50-69	70+	All ages
**POPULATION SIZE (MILLIONS)**				
2000	115	21.7	5.4	242
2010	153	29.3	7.3	303
2019	177	39.7	8.8	357
Rate of change in population size (% per year)				
2000-2010	2.9	3.0	3.1	2.3
2010-2019	1.6	3.4	2.1	1.8
2000-2019	2.3	3.2	2.6	2.1
**DEATHS (THOUSANDS)**				
2000	142	314	324	791
2010	165	335	423	934
2019	169	386	446	1 010
Rate of change in deaths (% per year)				
2000-2010	1.5	0.7	2.7	1.7
2010-2019	0.3	1.6	0.6	0.9
2000-2019	0.9	1.1	1.7	1.3
**DEATH RATE (PER 100 000 POPULATION PER YEAR)**				
2000	124	1 450	5 990	327
2010	108	1 140	5 780	308
2019	95.1	972	5 050	283
Rate of change in death rate (% per year)				
2000-2010	-1.4	-2.3	-0.4	-0.6
2010-2019	-1.4	-1.8	-1.5	-0.9
2000-2019	-1.4	-2.1	-0.9	-0.8

DATA SOURCE:

World Health Organization (WHO). Global Health Estimates, 2000-2021. Geneva: Department of Data and Analytics (DNA) Division of Data, Analytics and Delivery for Impact (DDI), WHO, 2024.

NOTE:

Rates of change between any two years are assumed to be constant. Hence the rate of change in the number of deaths will be the sum of the rate of change in the population size and the rate of change in the death rate, except for rounding.

TABLE A.16

Changes in deaths from selected NCD-7 conditions decomposed into changes in age group size and age group specific mortality rates, by CIH region, 2000-2010, 2010-2019, and 2000-2019.

Panel A3: NCD-7 – Central and Eastern Europe	Age group			
	15-49	50-69	70+	All ages
**POPULATION SIZE (MILLIONS)**				
2000	181	70.2	29.2	343
2010	169	78.6	34.2	332
2019	153	85.8	35.2	329
Rate of change in population size (% per year)				
2000-2010	-0.7	1.1	1.6	-0.3
2010-2019	-1.0	1.0	0.3	-0.1
2000-2019	-0.9	1.1	1.0	-0.2
**DEATHS (THOUSANDS)**				
2000	297	944	1 850	3 090
2010	204	735	1 900	2 840
2019	140	688	1 680	2 510
Rate of change in deaths (% per year)				
2000-2010	-3.7	-2.5	0.3	-0.9
2010-2019	-4.0	-0.7	-1.4	-1.4
2000-2019	-3.9	-1.6	-0.5	-1.1
**DEATH RATE (PER 100 000 POPULATION PER YEAR)**				
2000	164	1 340	6 340	901
2010	121	934	5 560	855
2019	91.6	802	4 770	762
Rate of change in death rate (% per year)				
2000-2010	-3.0	-3.6	-1.3	-0.5
2010-2019	-3.0	-1.7	-1.7	-1.3
2000-2019	-3.0	-2.7	-1.5	-0.9

DATA SOURCE:

World Health Organization (WHO). Global Health Estimates, 2000-2021. Geneva: Department of Data and Analytics (DNA) Division of Data, Analytics and Delivery for Impact (DDI), WHO, 2024.

NOTE:

Rates of change between any two years are assumed to be constant. Hence the rate of change in the number of deaths will be the sum of the rate of change in the population size and the rate of change in the death rate, except for rounding.

TABLE A.16

Changes in deaths from selected NCD-7 conditions decomposed into changes in age group size and age group specific mortality rates, by CIH region, 2000-2010, 2010-2019, and 2000-2019.

Panel A3: NCD-7 – China	Age group			
	15-49	50-69	70+	All ages
**POPULATION SIZE (MILLIONS)**				
2000	723	180	54.9	1 270
2010	767	257	77.6	1 350
2019	708	354	103	1 420
Rate of change in population size (% per year)				
2000-2010	0.6	3.6	3.5	0.6
2010-2019	-0.9	3.6	3.1	0.6
2000-2019	-0.1	3.6	3.3	0.6
**DEATHS (THOUSANDS)**				
2000	726	1 750	3 610	6 110
2010	658	1 800	4 710	7 190
2019	514	2 060	5 150	7 730
Rate of change in deaths (% per year)				
2000-2010	-1.0	0.3	2.7	1.6
2010-2019	-2.7	1.5	1.0	0.8
2000-2019	-1.8	0.9	1.9	1.2
**DEATH RATE (PER 100 000 POPULATION PER YEAR)**				
2000	100	971	6 570	482
2010	85.8	703	6 070	532
2019	72.5	583	5 020	543
Rate of change in death rate (% per year)				
2000-2010	-1.6	-3.2	-0.8	1.0
2010-2019	-1.8	-2.1	-2.1	0.2
2000-2019	-1.7	-2.6	-1.4	0.6

DATA SOURCE:

World Health Organization (WHO). Global Health Estimates, 2000-2021. Geneva: Department of Data and Analytics (DNA) Division of Data, Analytics and Delivery for Impact (DDI), WHO, 2024.

NOTE:

Rates of change between any two years are assumed to be constant. Hence the rate of change in the number of deaths will be the sum of the rate of change in the population size and the rate of change in the death rate, except for rounding.

TABLE A.16

Changes in deaths from selected NCD-7 conditions decomposed into changes in age group size and age group specific mortality rates, by CIH region, 2000-2010, 2010-2019, and 2000-2019.

Panel A5: NCD-7 - India	Age group			
	15-49	50-69	70+	All ages
**POPULATION SIZE (MILLIONS)**				
2000	543	111	27.6	1 060
2010	659	157	37.8	1 240
2019	757	209	50.5	1 390
Rate of change in population size (% per year)				
2000-2010	1.9	3.5	3.2	1.6
2010-2019	1.6	3.2	3.3	1.2
2000-2019	1.8	3.4	3.2	1.4
**DEATHS (THOUSANDS)**				
2000	557	987	918	2 510
2010	698	1 320	1 420	3 470
2019	699	1 960	2 100	4 770
Rate of change in deaths (% per year)				
2000-2010	2.3	2.9	4.5	3.3
2010-2019	0.0	4.5	4.4	3.6
2000-2019	1.2	3.7	4.4	3.4
**DEATH RATE (PER 100 000 POPULATION PER YEAR)**				
2000	103	891	3 330	238
2010	106	838	3 770	279
2019	92.3	939	4 150	344
Rate of change in death rate (% per year)				
2000-2010	0.3	-0.6	1.2	1.6
2010-2019	-1.5	1.3	1.1	2.3
2000-2019	-0.6	0.3	1.2	2.0

DATA SOURCE:

World Health Organization (WHO). Global Health Estimates, 2000-2021. Geneva: Department of Data and Analytics (DNA) Division of Data, Analytics and Delivery for Impact (DDI), WHO, 2024.

NOTE:

Rates of change between any two years are assumed to be constant. Hence the rate of change in the number of deaths will be the sum of the rate of change in the population size and the rate of change in the death rate, except for rounding.

TABLE A.16

Changes in deaths from selected NCD-7 conditions decomposed into changes in age group size and age group specific mortality rates, by CIH region, 2000-2010, 2010-2019, and 2000-2019.

Panel A6: NCD-7 – Latin America and Caribbean	Age group			
	15-49	50-69	70+	All ages
**POPULATION SIZE (MILLIONS)**				
2000	272	59.4	17.7	515
2010	312	83.7	25.4	583
2019	337	110	34.6	636
Rate of change in population size (% per year)				
2000-2010	1.4	3.5	3.7	1.2
2010-2019	0.8	3.1	3.5	1.0
2000-2019	1.1	3.3	3.6	1.1
**DEATHS (THOUSANDS)**				
2000	179	360	584	1 140
2010	196	420	764	1 390
2019	196	501	913	1 620
Rate of change in deaths (% per year)				
2000-2010	0.9	1.6	2.7	2.0
2010-2019	0.0	2.0	2.0	1.7
2000-2019	0.5	1.8	2.4	1.9
**DEATH RATE (PER 100 000 POPULATION PER YEAR)**				
2000	66.0	606	3 300	221
2010	62.7	501	3 010	238
2019	58.2	454	2 640	254
Rate of change in death rate (% per year)				
2000-2010	-0.5	-1.9	-0.9	0.8
2010-2019	-0.8	-1.1	-1.5	0.7
2000-2019	-0.7	-1.5	-1.2	0.7

DATA SOURCE:

World Health Organization (WHO). Global Health Estimates, 2000-2021. Geneva: Department of Data and Analytics (DNA) Division of Data, Analytics and Delivery for Impact (DDI), WHO, 2024.

NOTE:

Rates of change between any two years are assumed to be constant. Hence the rate of change in the number of deaths will be the sum of the rate of change in the population size and the rate of change in the death rate, except for rounding.

TABLE A.16

Changes in deaths from selected NCD-7 conditions decomposed into changes in age group size and age group specific mortality rates, by CIH region, 2000-2010, 2010-2019, and 2000-2019.

Panel A7: NCD-7 – Middle East and North Africa	Age group			
	15-49	50-69	70+	All ages
**POPULATION SIZE (MILLIONS)**				
2000	200	36.9	9.6	381
2010	259	53.6	13.8	467
2019	296	76.1	18.3	553
Rate of change in population size (% per year)				
2000-2010	2.6	3.8	3.7	2.0
2010-2019	1.5	4.0	3.2	1.9
2000-2019	2.1	3.9	3.5	2.0
**DEATHS (THOUSANDS)**				
2000	148	316	397	884
2010	173	386	549	1 130
2019	169	492	722	1 400
Rate of change in deaths (% per year)				
2000-2010	1.5	2.0	3.3	2.4
2010-2019	-0.2	2.7	3.1	2.4
2000-2019	0.7	2.4	3.2	2.4
**DEATH RATE (PER 100 000 POPULATION PER YEAR)**				
2000	74.3	857	4 130	232
2010	66.6	721	3 970	241
2019	57.1	646	3 940	253
Rate of change in death rate (% per year)				
2000-2010	-1.1	-1.7	-0.4	0.4
2010-2019	-1.7	-1.2	-0.1	0.5
2000-2019	-1.4	-1.5	-0.3	0.5

DATA SOURCE:

World Health Organization (WHO). Global Health Estimates, 2000-2021. Geneva: Department of Data and Analytics (DNA) Division of Data, Analytics and Delivery for Impact (DDI), WHO, 2024.

NOTE:

Rates of change between any two years are assumed to be constant. Hence the rate of change in the number of deaths will be the sum of the rate of change in the population size and the rate of change in the death rate, except for rounding.

TABLE A.16

Changes in deaths from selected NCD-7 conditions decomposed into changes in age group size and age group specific mortality rates, by CIH region, 2000-2010, 2010-2019, and 2000-2019.

Panel A8: NCD-7 – North Atlantic	Age group			
	15-49	50-69	70+	All ages
**POPULATION SIZE (MILLIONS)**				
2000	209	93.2	47.7	422
2010	211	107	57.5	446
2019	202	122	67.8	464
Rate of change in population size (% per year)				
2000-2010	0.1	1.4	1.9	0.6
2010-2019	-0.5	1.5	1.8	0.4
2000-2019	-0.2	1.4	1.9	0.5
**DEATHS (THOUSANDS)**				
2000	113	399	1 560	2 080
2010	81.8	335	1 400	1 820
2019	60.3	323	1 310	1 700
Rate of change in deaths (% per year)				
2000-2010	-3.2	-1.7	-1.1	-1.3
2010-2019	-3.3	-0.4	-0.7	-0.8
2000-2019	-3.2	-1.1	-0.9	-1.1
**DEATH RATE (PER 100 000 POPULATION PER YEAR)**				
2000	54.1	428	3 280	493
2010	38.8	314	2 440	408
2019	29.8	266	1 940	366
Rate of change in death rate (% per year)				
2000-2010	-3.3	-3.0	-2.9	-1.9
2010-2019	-2.9	-1.8	-2.5	-1.2
2000-2019	-3.1	-2.5	-2.7	-1.6

DATA SOURCE:

World Health Organization (WHO). Global Health Estimates, 2000-2021. Geneva: Department of Data and Analytics (DNA) Division of Data, Analytics and Delivery for Impact (DDI), WHO, 2024.

NOTE:

Rates of change between any two years are assumed to be constant. Hence the rate of change in the number of deaths will be the sum of the rate of change in the population size and the rate of change in the death rate, except for rounding.

TABLE A.16

Changes in deaths from selected NCD-7 conditions decomposed into changes in age group size and age group specific mortality rates, by CIH region, 2000-2010, 2010-2019, and 2000-2019.

Panel A9: NCD-7 – Sub-Saharan Africa	Age group			
	15-49	50-69	70+	All ages
**POPULATION SIZE (MILLIONS)**				
2000	313	52.5	11.8	682
2010	418	69.0	15.6	895
2019	544	93.4	20.4	1 140
Rate of change in population size (% per year)				
2000-2010	2.9	2.8	2.8	2.8
2010-2019	3.0	3.4	3.0	2.7
2000-2019	3.0	3.1	2.9	2.7
**DEATHS (THOUSANDS)**				
2000	232	460	424	1 200
2010	303	549	548	1 470
2019	370	676	694	1 810
Rate of change in deaths (% per year)				
2000-2010	2.7	1.8	2.6	2.1
2010-2019	2.2	2.3	2.6	2.3
2000-2019	2.5	2.0	2.6	2.2
**DEATH RATE (PER 100 000 POPULATION PER YEAR)**				
2000	74.2	876	3 600	175
2010	72.5	796	3 520	164
2019	67.9	723	3 410	159
Rate of change in death rate (% per year)				
2000-2010	-0.2	-1.0	-0.2	-0.7
2010-2019	-0.7	-1.1	-0.4	-0.4
2000-2019	-0.5	-1.0	-0.3	-0.5

DATA SOURCE:

World Health Organization (WHO). Global Health Estimates, 2000-2021. Geneva: Department of Data and Analytics (DNA) Division of Data, Analytics and Delivery for Impact (DDI), WHO, 2024.

NOTE:

Rates of change between any two years are assumed to be constant. Hence the rate of change in the number of deaths will be the sum of the rate of change in the population size and the rate of change in the death rate, except for rounding.

TABLE A.16

Changes in deaths from selected NCD-7 conditions decomposed into changes in age group size and age group specific mortality rates, by CIH region, 2000-2010, 2010-2019, and 2000-2019.

Panel A10: NCD-7 – United States	Age group			
	15-49	50-69	70+	All ages
**POPULATION SIZE (MILLIONS)**				
2000	146	51.4	24.9	281
2010	151	71.3	27.5	311
2019	157	82.2	35.5	338
Rate of change in population size (% per year)				
2000-2010	0.4	3.3	1.0	1.0
2010-2019	0.5	1.6	2.9	0.9
2000-2019	0.4	2.5	1.9	1.0
**DEATHS (THOUSANDS)**				
2000	97.6	274	925	1 300
2010	86.9	300	752	1 140
2019	86.2	330	793	1 210
Rate of change in deaths (% per year)				
2000-2010	-1.2	0.9	-2.1	-1.3
2010-2019	-0.1	1.1	0.6	0.7
2000-2019	-0.7	1.0	-0.8	-0.4
**DEATH RATE (PER 100 000 POPULATION PER YEAR)**				
2000	67.0	534	3 720	462
2010	57.6	420	2 730	366
2019	54.8	401	2 240	359
Rate of change in death rate (% per year)				
2000-2010	-1.5	-2.4	-3.0	-2.3
2010-2019	-0.5	-0.5	-2.2	-0.2
2000-2019	-1.0	-1.5	-2.6	-1.3

DATA SOURCE:

World Health Organization (WHO). Global Health Estimates, 2000-2021. Geneva: Department of Data and Analytics (DNA) Division of Data, Analytics and Delivery for Impact (DDI), WHO, 2024.

NOTE:

Rates of change between any two years are assumed to be constant. Hence the rate of change in the number of deaths will be the sum of the rate of change in the population size and the rate of change in the death rate, except for rounding.

TABLE A.16

Changes in deaths from selected NCD-7 conditions decomposed into changes in age group size and age group specific mortality rates, by CIH region, 2000-2010, 2010-2019, and 2000-2019.

Panel A11: NCD-7 – Western Pacific and Southeast Asia	Age group			
	15-49	50-69	70+	All ages
**POPULATION SIZE (MILLIONS)**				
2000	492	127	38.9	934
2010	549	163	56.0	1 040
2019	582	205	74.4	1 130
Rate of change in population size (% per year)				
2000-2010	1.1	2.5	3.7	1.1
2010-2019	0.6	2.6	3.2	0.9
2000-2019	0.9	2.5	3.5	1.0
**DEATHS (THOUSANDS)**				
2000	451	958	1 290	2 730
2010	480	1 130	1 790	3 430
2019	468	1 340	2 180	4 000
Rate of change in deaths (% per year)				
2000-2010	0.6	1.7	3.3	2.3
2010-2019	-0.3	1.9	2.2	1.7
2000-2019	0.2	1.8	2.8	2.0
**DEATH RATE (PER 100 000 POPULATION PER YEAR)**				
2000	91.6	753	3 320	293
2010	87.5	695	3 200	329
2019	80.5	655	2 920	354
Rate of change in death rate (% per year)				
2000-2010	-0.5	-0.8	-0.4	1.2
2010-2019	-0.9	-0.7	-1.0	0.8
2000-2019	-0.7	-0.7	-0.7	1.0

DATA SOURCE:

World Health Organization (WHO). Global Health Estimates, 2000-2021. Geneva: Department of Data and Analytics (DNA) Division of Data, Analytics and Delivery for Impact (DDI), WHO, 2024.

NOTE:

Rates of change between any two years are assumed to be constant. Hence the rate of change in the number of deaths will be the sum of the rate of change in the population size and the rate of change in the death rate, except for rounding.

TABLE A.16

Changes in deaths from selected NCD-7 conditions decomposed into changes in age group size and age group specific mortality rates, by CIH region, 2000-2010, 2010-2019, and 2000-2019.

Panel B1: Atherosclerotic CVD – World	Age group			
	15-49	50-69	70+	All ages
**POPULATION SIZE (MILLIONS)**				
2000	3 210	808	269	6 160
2010	3 670	1 080	355	7 010
2019	3 930	1 380	451	7 800
Rate of change in population size (% per year)				
2000-2010	1.3	2.9	2.8	1.3
2010-2019	0.8	2.8	2.7	1.2
2000-2019	1.1	2.9	2.8	1.2
**DEATHS (THOUSANDS)**				
2000	565	2 350	6 070	8 990
2010	633	2 580	7 380	10 600
2019	639	3 190	8 230	12 100
Rate of change in deaths (% per year)				
2000-2010	1.1	0.9	2.0	1.6
2010-2019	0.1	2.4	1.2	1.5
2000-2019	0.7	1.6	1.6	1.6
**DEATH RATE (PER 100 000 POPULATION PER YEAR)**				
2000	17.6	292	2 250	146
2010	17.3	240	2 080	151
2019	16.3	231	1 820	155
Rate of change in death rate (% per year)				
2000-2010	-0.2	-1.9	-0.8	0.3
2010-2019	-0.7	-0.4	-1.4	0.3
2000-2019	-0.4	-1.2	-1.1	0.3

DATA SOURCE:

World Health Organization (WHO). Global Health Estimates, 2000-2021. Geneva: Department of Data and Analytics (DNA) Division of Data, Analytics and Delivery for Impact (DDI), WHO, 2024.

NOTE:

Rates of change between any two years are assumed to be constant. Hence the rate of change in the number of deaths will be the sum of the rate of change in the population size and the rate of change in the death rate, except for rounding.

TABLE A.16

Changes in deaths from selected NCD-7 conditions decomposed into changes in age group size and age group specific mortality rates, by CIH region, 2000-2010, 2010-2019, and 2000-2019.

Panel B2: Atherosclerotic CVD – Central Asia	Age group			
	15-49	50-69	70+	All ages
**POPULATION SIZE (MILLIONS)**				
2000	115	21.7	5.4	242
2010	153	29.3	7.3	303
2019	177	39.7	8.8	357
Rate of change in population size (% per year)				
2000-2010	2.9	3.0	3.1	2.3
2010-2019	1.6	3.4	2.1	1.8
2000-2019	2.3	3.2	2.6	2.1
**DEATHS (THOUSANDS)**				
2000	39.5	144	193	377
2010	44.8	150	259	454
2019	47.5	178	271	497
Rate of change in deaths (% per year)				
2000-2010	1.3	0.5	3.0	1.9
2010-2019	0.7	1.9	0.5	1.0
2000-2019	1.0	1.1	1.8	1.5
**DEATH RATE (PER 100 000 POPULATION PER YEAR)**				
2000	34.4	661	3 570	156
2010	29.3	513	3 540	150
2019	26.8	449	3 070	139
Rate of change in death rate (% per year)				
2000-2010	-1.6	-2.5	-0.1	-0.4
2010-2019	-1.0	-1.5	-1.6	-0.8
2000-2019	-1.3	-2.0	-0.8	-0.6

DATA SOURCE:

World Health Organization (WHO). Global Health Estimates, 2000-2021. Geneva: Department of Data and Analytics (DNA) Division of Data, Analytics and Delivery for Impact (DDI), WHO, 2024.

NOTE:

Rates of change between any two years are assumed to be constant. Hence the rate of change in the number of deaths will be the sum of the rate of change in the population size and the rate of change in the death rate, except for rounding.

TABLE A.16

Changes in deaths from selected NCD-7 conditions decomposed into changes in age group size and age group specific mortality rates, by CIH region, 2000-2010, 2010-2019, and 2000-2019.

Panel B3: Atherosclerotic CVD – Central and Eastern Europe	Age group			
	15-49	50-69	70+	All ages
**POPULATION SIZE (MILLIONS)**				
2000	181	70.2	29.2	343
2010	169	78.6	34.2	332
2019	153	85.8	35.2	329
Rate of change in population size (% per year)				
2000-2010	-0.7	1.1	1.6	-0.3
2010-2019	-1.0	1.0	0.3	-0.1
2000-2019	-0.9	1.1	1.0	-0.2
**DEATHS (THOUSANDS)**				
2000	87.9	536	1 500	2 120
2010	58.3	420	1 570	2 050
2019	38.0	379	1 350	1 770
Rate of change in deaths (% per year)				
2000-2010	-4.0	-2.4	0.5	-0.3
2010-2019	-4.6	-1.1	-1.6	-1.6
2000-2019	-4.3	-1.8	-0.5	-1.0
**DEATH RATE (PER 100 000 POPULATION PER YEAR)**				
2000	48.7	764	5 140	618
2010	34.5	534	4 600	618
2019	24.7	442	3 840	538
Rate of change in death rate (% per year)				
2000-2010	-3.4	-3.5	-1.1	0.0
2010-2019	-3.6	-2.1	-2.0	-1.5
2000-2019	-3.5	-2.8	-1.5	-0.7

DATA SOURCE:

World Health Organization (WHO). Global Health Estimates, 2000-2021. Geneva: Department of Data and Analytics (DNA) Division of Data, Analytics and Delivery for Impact (DDI), WHO, 2024.

NOTE:

Rates of change between any two years are assumed to be constant. Hence the rate of change in the number of deaths will be the sum of the rate of change in the population size and the rate of change in the death rate, except for rounding.

TABLE A.16

Changes in deaths from selected NCD-7 conditions decomposed into changes in age group size and age group specific mortality rates, by CIH region, 2000-2010, 2010-2019, and 2000-2019.

Panel B4: Atherosclerotic CVD – China	Age group			
	15-49	50-69	70+	All ages
**POPULATION SIZE (MILLIONS)**				
2000	723	180	54.9	1 270
2010	767	257	77.6	1 350
2019	708	354	103	1 420
Rate of change in population size (% per year)				
2000-2010	0.6	3.6	3.5	0.6
2010-2019	-0.9	3.6	3.1	0.6
2000-2019	-0.1	3.6	3.3	0.6
**DEATHS (THOUSANDS)**				
2000	90.4	357	1 030	1 480
2010	111	464	1 860	2 430
2019	94.8	587	2 260	2 940
Rate of change in deaths (% per year)				
2000-2010	2.0	2.7	6.1	5.1
2010-2019	-1.7	2.6	2.2	2.1
2000-2019	0.3	2.6	4.2	3.7
**DEATH RATE (PER 100 000 POPULATION PER YEAR)**				
2000	12.5	198	1 880	117
2010	14.4	181	2 400	180
2019	13.4	166	2 210	207
Rate of change in death rate (% per year)				
2000-2010	1.4	-0.9	2.5	4.4
2010-2019	-0.8	-0.9	-0.9	1.6
2000-2019	0.4	-0.9	0.8	3.1

DATA SOURCE:

World Health Organization (WHO). Global Health Estimates, 2000-2021. Geneva: Department of Data and Analytics (DNA) Division of Data, Analytics and Delivery for Impact (DDI), WHO, 2024.

NOTE:

Rates of change between any two years are assumed to be constant. Hence the rate of change in the number of deaths will be the sum of the rate of change in the population size and the rate of change in the death rate, except for rounding.

TABLE A.16

Changes in deaths from selected NCD-7 conditions decomposed into changes in age group size and age group specific mortality rates, by CIH region, 2000-2010, 2010-2019, and 2000-2019.

Panel B5: Atherosclerotic CVD – India	Age group			
	15-49	50-69	70+	All ages
**POPULATION SIZE (MILLIONS)**				
2000	543	111	27.6	1 060
2010	659	157	37.8	1 240
2019	757	209	50.5	1 390
Rate of change in population size (% per year)				
2000-2010	1.9	3.5	3.2	1.6
2010-2019	1.6	3.2	3.3	1.2
2000-2019	1.8	3.4	3.2	1.4
**DEATHS (THOUSANDS)**				
2000	118	360	371	850
2010	168	506	586	1 260
2019	194	786	888	1 870
Rate of change in deaths (% per year)				
2000-2010	3.6	3.5	4.7	4.0
2010-2019	1.6	5.0	4.7	4.5
2000-2019	2.6	4.2	4.7	4.2
**DEATH RATE (PER 100 000 POPULATION PER YEAR)**				
2000	21.7	325	1 350	80.3
2010	25.6	322	1 550	101
2019	25.7	377	1 760	135
Rate of change in death rate (% per year)				
2000-2010	1.6	-0.1	1.4	2.4
2010-2019	0.0	1.8	1.4	3.2
2000-2019	0.9	0.8	1.4	2.8

DATA SOURCE:

World Health Organization (WHO). Global Health Estimates, 2000-2021. Geneva: Department of Data and Analytics (DNA) Division of Data, Analytics and Delivery for Impact (DDI), WHO, 2024.

NOTE:

Rates of change between any two years are assumed to be constant. Hence the rate of change in the number of deaths will be the sum of the rate of change in the population size and the rate of change in the death rate, except for rounding.

TABLE A.16

Changes in deaths from selected NCD-7 conditions decomposed into changes in age group size and age group specific mortality rates, by CIH region, 2000-2010, 2010-2019, and 2000-2019.

Panel B6: Atherosclerotic CVD – Latin America and Caribbean	Age group			
	15-49	50-69	70+	All ages
**POPULATION SIZE (MILLIONS)**				
2000	272	59.4	17.7	515
2010	312	83.7	25.4	583
2019	337	110	34.6	636
Rate of change in population size (% per year)				
2000-2010	1.4	3.5	3.7	1.2
2010-2019	0.8	3.1	3.5	1.0
2000-2019	1.1	3.3	3.6	1.1
**DEATHS (THOUSANDS)**				
2000	32.4	129	316	478
2010	32.7	144	401	578
2019	35.7	176	477	689
Rate of change in deaths (% per year)				
2000-2010	0.1	1.0	2.4	1.9
2010-2019	1.0	2.3	2.0	2.0
2000-2019	0.5	1.6	2.2	2.0
**DEATH RATE (PER 100 000 POPULATION PER YEAR)**				
2000	11.9	218	1 790	92.7
2010	10.5	171	1 580	99.1
2019	10.6	160	1 380	108
Rate of change in death rate (% per year)				
2000-2010	-1.3	-2.4	-1.2	0.7
2010-2019	0.1	-0.8	-1.5	1.0
2000-2019	-0.6	-1.6	-1.4	0.8

DATA SOURCE:

World Health Organization (WHO). Global Health Estimates, 2000-2021. Geneva: Department of Data and Analytics (DNA) Division of Data, Analytics and Delivery for Impact (DDI), WHO, 2024.

NOTE:

Rates of change between any two years are assumed to be constant. Hence the rate of change in the number of deaths will be the sum of the rate of change in the population size and the rate of change in the death rate, except for rounding.

TABLE A.16

Changes in deaths from selected NCD-7 conditions decomposed into changes in age group size and age group specific mortality rates, by CIH region, 2000-2010, 2010-2019, and 2000-2019.

Panel B7: Atherosclerotic CVD – Middle East and North Africa	Age group			
	15-49	50-69	70+	All ages
**POPULATION SIZE (MILLIONS)**				
2000	200	36.9	9.6	381
2010	259	53.6	13.8	467
2019	296	76.1	18.3	553
Rate of change in population size (% per year)				
2000-2010	2.6	3.8	3.7	2.0
2010-2019	1.5	4.0	3.2	1.9
2000-2019	2.1	3.9	3.5	2.0
**DEATHS (THOUSANDS)**				
2000	49.0	166	259	475
2010	53.4	194	355	604
2019	56.0	249	471	776
Rate of change in deaths (% per year)				
2000-2010	0.9	1.6	3.2	2.4
2010-2019	0.5	2.8	3.2	2.8
2000-2019	0.7	2.1	3.2	2.6
**DEATH RATE (PER 100 000 POPULATION PER YEAR)**				
2000	24.5	451	2 690	125
2010	20.6	363	2 570	129
2019	18.9	327	2 570	140
Rate of change in death rate (% per year)				
2000-2010	-1.7	-2.1	-0.5	0.4
2010-2019	-1.0	-1.1	0.0	0.9
2000-2019	-1.4	-1.7	-0.2	0.6

DATA SOURCE:

World Health Organization (WHO). Global Health Estimates, 2000-2021. Geneva: Department of Data and Analytics (DNA) Division of Data, Analytics and Delivery for Impact (DDI), WHO, 2024.

NOTE:

Rates of change between any two years are assumed to be constant. Hence the rate of change in the number of deaths will be the sum of the rate of change in the population size and the rate of change in the death rate, except for rounding.

TABLE A.16

Changes in deaths from selected NCD-7 conditions decomposed into changes in age group size and age group specific mortality rates, by CIH region, 2000-2010, 2010-2019, and 2000-2019.

Panel B8: Atherosclerotic CVD – North Atlantic	Age group			
	15-49	50-69	70+	All ages
**POPULATION SIZE (MILLIONS)**				
2000	209	93.2	47.7	422
2010	211	107	57.5	446
2019	202	122	67.8	464
Rate of change in population size (% per year)				
2000-2010	0.1	1.4	1.9	0.6
2010-2019	-0.5	1.5	1.8	0.4
2000-2019	-0.2	1.4	1.9	0.5
**DEATHS (THOUSANDS)**				
2000	20.7	156	1 010	1 190
2010	15.8	106	824	946
2019	10.3	94.9	708	813
Rate of change in deaths (% per year)				
2000-2010	-2.7	-3.8	-2.0	-2.3
2010-2019	-4.6	-1.2	-1.7	-1.7
2000-2019	-3.6	-2.6	-1.9	-2.0
**DEATH RATE (PER 100 000 POPULATION PER YEAR)**				
2000	9.9	167	2 120	282
2010	7.5	99.1	1 430	212
2019	5.1	78.0	1 040	175
Rate of change in death rate (% per year)				
2000-2010	-2.8	-5.1	-3.8	-2.8
2010-2019	-4.2	-2.6	-3.5	-2.1
2000-2019	-3.4	-3.9	-3.7	-2.5

DATA SOURCE:

World Health Organization (WHO). Global Health Estimates, 2000-2021. Geneva: Department of Data and Analytics (DNA) Division of Data, Analytics and Delivery for Impact (DDI), WHO, 2024.

NOTE:

Rates of change between any two years are assumed to be constant. Hence the rate of change in the number of deaths will be the sum of the rate of change in the population size and the rate of change in the death rate, except for rounding.

TABLE A.16

Changes in deaths from selected NCD-7 conditions decomposed into changes in age group size and age group specific mortality rates, by CIH region, 2000-2010, 2010-2019, and 2000-2019.

Panel B9: Atherosclerotic CVD – Sub-Saharan Africa	Age group			
	15-49	50-69	70+	All ages
**POPULATION SIZE (MILLIONS)**				
2000	313	52.5	11.8	682
2010	418	69.0	15.6	895
2019	544	93.4	20.4	1 140
Rate of change in population size (% per year)				
2000-2010	2.9	2.8	2.8	2.8
2010-2019	3.0	3.4	3.0	2.7
2000-2019	3.0	3.1	2.9	2.7
**DEATHS (THOUSANDS)**				
2000	27.1	125	188	341
2010	37.8	153	249	441
2019	47.4	193	321	563
Rate of change in deaths (% per year)				
2000-2010	3.4	2.1	2.8	2.6
2010-2019	2.5	2.6	2.9	2.8
2000-2019	3.0	2.3	2.8	2.7
**DEATH RATE (PER 100 000 POPULATION PER YEAR)**				
2000	8.7	237	1 600	50.0
2010	9.0	221	1 600	49.3
2019	8.7	207	1 580	49.4
Rate of change in death rate (% per year)				
2000-2010	0.4	-0.7	0.0	-0.2
2010-2019	-0.4	-0.8	-0.1	0.0
2000-2019	0.0	-0.7	-0.1	-0.1

DATA SOURCE:

World Health Organization (WHO). Global Health Estimates, 2000-2021. Geneva: Department of Data and Analytics (DNA) Division of Data, Analytics and Delivery for Impact (DDI), WHO, 2024.

NOTE:

Rates of change between any two years are assumed to be constant. Hence the rate of change in the number of deaths will be the sum of the rate of change in the population size and the rate of change in the death rate, except for rounding.

TABLE A.16

Changes in deaths from selected NCD-7 conditions decomposed into changes in age group size and age group specific mortality rates, by CIH region, 2000-2010, 2010-2019, and 2000-2019.

Panel B10: Atherosclerotic CVD – United States	Age group			
	15-49	50-69	70+	All ages
**POPULATION SIZE (MILLIONS)**				
2000	146	51.4	24.9	281
2010	151	71.3	27.5	311
2019	157	82.2	35.5	338
Rate of change in population size (% per year)				
2000-2010	0.4	3.3	1.0	1.0
2010-2019	0.5	1.6	2.9	0.9
2000-2019	0.4	2.5	1.9	1.0
**DEATHS (THOUSANDS)**				
2000	21.6	116	598	736
2010	18.3	110	427	555
2019	14.8	122	430	567
Rate of change in deaths (% per year)				
2000-2010	-1.6	-0.6	-3.3	-2.8
2010-2019	-2.3	1.2	0.1	0.2
2000-2019	-2.0	0.3	-1.7	-1.4
**DEATH RATE (PER 100 000 POPULATION PER YEAR)**				
2000	14.8	226	2 400	261
2010	12.1	154	1 550	179
2019	9.4	149	1 210	168
Rate of change in death rate (% per year)				
2000-2010	-2.0	-3.8	-4.3	-3.7
2010-2019	-2.8	-0.4	-2.7	-0.7
2000-2019	-2.4	-2.2	-3.5	-2.3

DATA SOURCE:

World Health Organization (WHO). Global Health Estimates, 2000-2021. Geneva: Department of Data and Analytics (DNA) Division of Data, Analytics and Delivery for Impact (DDI), WHO, 2024.

NOTE:

Rates of change between any two years are assumed to be constant. Hence the rate of change in the number of deaths will be the sum of the rate of change in the population size and the rate of change in the death rate, except for rounding.

TABLE A.16

Changes in deaths from selected NCD-7 conditions decomposed into changes in age group size and age group specific mortality rates, by CIH region, 2000-2010, 2010-2019, and 2000-2019.

Panel B11: Atherosclerotic CVD – Western Pacific and Southeast Asia	Age group			
	15-49	50-69	70+	All ages
**POPULATION SIZE (MILLIONS)**				
2000	492	127	38.9	934
2010	549	163	56.0	1 040
2019	582	205	74.4	1 130
Rate of change in population size (% per year)				
2000-2010	1.1	2.5	3.7	1.1
2010-2019	0.6	2.6	3.2	0.9
2000-2019	0.9	2.5	3.5	1.0
**DEATHS (THOUSANDS)**				
2000	76.5	260	582	919
2010	91.7	327	821	1 240
2019	98.9	421	1 020	1 540
Rate of change in deaths (% per year)				
2000-2010	1.8	2.3	3.5	3.0
2010-2019	0.9	2.9	2.4	2.4
2000-2019	1.4	2.6	3.0	2.8
**DEATH RATE (PER 100 000 POPULATION PER YEAR)**				
2000	15.6	204	1 500	98.4
2010	16.7	201	1 470	119
2019	17.0	206	1 370	136
Rate of change in death rate (% per year)				
2000-2010	0.7	-0.2	-0.2	1.9
2010-2019	0.2	0.3	-0.7	1.5
2000-2019	0.5	0.0	-0.5	1.7

DATA SOURCE:

World Health Organization (WHO). Global Health Estimates, 2000-2021. Geneva: Department of Data and Analytics (DNA) Division of Data, Analytics and Delivery for Impact (DDI), WHO, 2024.

NOTE:

Rates of change between any two years are assumed to be constant. Hence the rate of change in the number of deaths will be the sum of the rate of change in the population size and the rate of change in the death rate, except for rounding.

TABLE A.16

Changes in deaths from selected NCD-7 conditions decomposed into changes in age group size and age group specific mortality rates, by CIH region, 2000-2010, 2010-2019, and 2000-2019.

Panel C1: Hemorrhagic stroke - World	Age group			
	15-49	50-69	70+	All ages
**POPULATION SIZE (MILLIONS)**				
2000	3 210	808	269	6 160
2010	3 670	1 080	355	7 010
2019	3 930	1 380	451	7 800
Rate of change in population size (% per year)				
2000-2010	1.3	2.9	2.8	1.3
2010-2019	0.8	2.8	2.7	1.2
2000-2019	1.1	2.9	2.8	1.2
**DEATHS (THOUSANDS)**				
2000	324	1 170	1 540	3 060
2010	337	1 200	1 770	3 330
2019	309	1 350	1 810	3 490
Rate of change in deaths (% per year)				
2000-2010	0.4	0.2	1.4	0.8
2010-2019	-0.9	1.3	0.3	0.5
2000-2019	-0.2	0.7	0.9	0.7
**DEATH RATE (PER 100 000 POPULATION PER YEAR)**				
2000	10.1	145	570	49.8
2010	9.2	112	497	47.5
2019	7.9	97.2	402	44.7
Rate of change in death rate (% per year)				
2000-2010	-0.9	-2.6	-1.4	-0.5
2010-2019	-1.7	-1.5	-2.3	-0.7
2000-2019	-1.3	-2.1	-1.8	-0.6

DATA SOURCE:

World Health Organization (WHO). Global Health Estimates, 2000-2021. Geneva: Department of Data and Analytics (DNA) Division of Data, Analytics and Delivery for Impact (DDI), WHO, 2024.

NOTE:

Rates of change between any two years are assumed to be constant. Hence the rate of change in the number of deaths will be the sum of the rate of change in the population size and the rate of change in the death rate, except for rounding.

TABLE A.16

Changes in deaths from selected NCD-7 conditions decomposed into changes in age group size and age group specific mortality rates, by CIH region, 2000-2010, 2010-2019, and 2000-2019.

Panel C2: Hemorrhagic stroke – Central Asia	Age group			
	15-49	50-69	70+	All ages
**POPULATION SIZE (MILLIONS)**				
2000	115	21.7	5.4	242
2010	153	29.3	7.3	303
2019	177	39.7	8.8	357
Rate of change in population size (% per year)				
2000-2010	2.9	3.0	3.1	2.3
2010-2019	1.6	3.4	2.1	1.8
2000-2019	2.3	3.2	2.6	2.1
**DEATHS (THOUSANDS)**				
2000	14.1	50.6	37.0	104
2010	15.1	50.8	45.2	113
2019	14.8	53.1	41.5	111
Rate of change in deaths (% per year)				
2000-2010	0.7	0.0	2.0	0.8
2010-2019	-0.2	0.5	-0.9	-0.2
2000-2019	0.2	0.3	0.6	0.4
**DEATH RATE (PER 100 000 POPULATION PER YEAR)**				
2000	12.3	233	684	42.8
2010	9.8	173	617	37.1
2019	8.3	134	470	31.1
Rate of change in death rate (% per year)				
2000-2010	-2.2	-2.9	-1.0	-1.4
2010-2019	-1.8	-2.8	-3.0	-2.0
2000-2019	-2.0	-2.9	-2.0	-1.7

DATA SOURCE:

World Health Organization (WHO). Global Health Estimates, 2000-2021. Geneva: Department of Data and Analytics (DNA) Division of Data, Analytics and Delivery for Impact (DDI), WHO, 2024.

NOTE:

Rates of change between any two years are assumed to be constant. Hence the rate of change in the number of deaths will be the sum of the rate of change in the population size and the rate of change in the death rate, except for rounding.

TABLE A.16

Changes in deaths from selected NCD-7 conditions decomposed into changes in age group size and age group specific mortality rates, by CIH region, 2000-2010, 2010-2019, and 2000-2019.

Panel C3: Hemorrhagic stroke – Central and Eastern Europe	Age group			
	15-49	50-69	70+	All ages
**POPULATION SIZE (MILLIONS)**				
2000	181	70.2	29.2	343
2010	169	78.6	34.2	332
2019	153	85.8	35.2	329
Rate of change in population size (% per year)				
2000-2010	-0.7	1.1	1.6	-0.3
2010-2019	-1.0	1.0	0.3	-0.1
2000-2019	-0.9	1.1	1.0	-0.2
**DEATHS (THOUSANDS)**				
2000	25.2	104	115	244
2010	16.8	67.9	94.1	179
2019	13.1	56.0	81.7	151
Rate of change in deaths (% per year)				
2000-2010	-4.0	-4.2	-2.0	-3.1
2010-2019	-2.8	-2.1	-1.6	-1.9
2000-2019	-3.4	-3.2	-1.8	-2.5
**DEATH RATE (PER 100 000 POPULATION PER YEAR)**				
2000	14.0	148	392	71.1
2010	10.0	86.3	275	53.9
2019	8.5	65.2	232	45.8
Rate of change in death rate (% per year)				
2000-2010	-3.3	-5.3	-3.5	-2.7
2010-2019	-1.7	-3.1	-1.9	-1.8
2000-2019	-2.6	-4.2	-2.7	-2.3

DATA SOURCE:

World Health Organization (WHO). Global Health Estimates, 2000-2021. Geneva: Department of Data and Analytics (DNA) Division of Data, Analytics and Delivery for Impact (DDI), WHO, 2024.

NOTE:

Rates of change between any two years are assumed to be constant. Hence the rate of change in the number of deaths will be the sum of the rate of change in the population size and the rate of change in the death rate, except for rounding.

TABLE A.16

Changes in deaths from selected NCD-7 conditions decomposed into changes in age group size and age group specific mortality rates, by CIH region, 2000-2010, 2010-2019, and 2000-2019.

Panel C4: Hemorrhagic stroke – China	Age group			
	15-49	50-69	70+	All ages
**POPULATION SIZE (MILLIONS)**				
2000	723	180	54.9	1 270
2010	767	257	77.6	1 350
2019	708	354	103	1 420
Rate of change in population size (% per year)				
2000-2010	0.6	3.6	3.5	0.6
2010-2019	-0.9	3.6	3.1	0.6
2000-2019	-0.1	3.6	3.3	0.6
**DEATHS (THOUSANDS)**				
2000	101	415	788	1 310
2010	99.9	395	902	1 400
2019	72.9	400	863	1 340
Rate of change in deaths (% per year)				
2000-2010	-0.1	-0.5	1.4	0.7
2010-2019	-3.4	0.1	-0.5	-0.5
2000-2019	-1.7	-0.2	0.5	0.1
**DEATH RATE (PER 100 000 POPULATION PER YEAR)**				
2000	14.0	230	1 440	103
2010	13.0	154	1 160	103
2019	10.3	113	841	93.9
Rate of change in death rate (% per year)				
2000-2010	-0.7	-4.0	-2.1	0.0
2010-2019	-2.6	-3.4	-3.5	-1.1
2000-2019	-1.6	-3.7	-2.8	-0.5

DATA SOURCE:

World Health Organization (WHO). Global Health Estimates, 2000-2021. Geneva: Department of Data and Analytics (DNA) Division of Data, Analytics and Delivery for Impact (DDI), WHO, 2024.

NOTE:

Rates of change between any two years are assumed to be constant. Hence the rate of change in the number of deaths will be the sum of the rate of change in the population size and the rate of change in the death rate, except for rounding.

TABLE A.16

Changes in deaths from selected NCD-7 conditions decomposed into changes in age group size and age group specific mortality rates, by CIH region, 2000-2010, 2010-2019, and 2000-2019.

Panel C5: Hemorrhagic stroke – India	Age group			
	15-49	50-69	70+	All ages
**POPULATION SIZE (MILLIONS)**				
2000	543	111	27.6	1 060
2010	659	157	37.8	1 240
2019	757	209	50.5	1 390
Rate of change in population size (% per year)				
2000-2010	1.9	3.5	3.2	1.6
2010-2019	1.6	3.2	3.3	1.2
2000-2019	1.8	3.4	3.2	1.4
**DEATHS (THOUSANDS)**				
2000	35.2	144	87.6	273
2010	46.1	179	117	346
2019	48.9	258	155	463
Rate of change in deaths (% per year)				
2000-2010	2.7	2.2	3.0	2.4
2010-2019	0.7	4.1	3.1	3.3
2000-2019	1.7	3.1	3.0	2.8
**DEATH RATE (PER 100 000 POPULATION PER YEAR)**				
2000	6.5	130	318	25.8
2010	7.0	114	310	27.8
2019	6.4	124	307	33.3
Rate of change in death rate (% per year)				
2000-2010	0.8	-1.3	-0.2	0.8
2010-2019	-0.9	0.9	-0.1	2.0
2000-2019	0.0	-0.3	-0.2	1.4

DATA SOURCE:

World Health Organization (WHO). Global Health Estimates, 2000-2021. Geneva: Department of Data and Analytics (DNA) Division of Data, Analytics and Delivery for Impact (DDI), WHO, 2024.

NOTE:

Rates of change between any two years are assumed to be constant. Hence the rate of change in the number of deaths will be the sum of the rate of change in the population size and the rate of change in the death rate, except for rounding.

TABLE A.16

Changes in deaths from selected NCD-7 conditions decomposed into changes in age group size and age group specific mortality rates, by CIH region, 2000-2010, 2010-2019, and 2000-2019.

Panel C6: Hemorrhagic stroke – Latin America and Caribbean	Age group			
	15-49	50-69	70+	All ages
**POPULATION SIZE (MILLIONS)**				
2000	272	59.4	17.7	515
2010	312	83.7	25.4	583
2019	337	110	34.6	636
Rate of change in population size (% per year)				
2000-2010	1.4	3.5	3.7	1.2
2010-2019	0.8	3.1	3.5	1.0
2000-2019	1.1	3.3	3.6	1.1
**DEATHS (THOUSANDS)**				
2000	22.8	52.6	47.0	124
2010	19.3	52.9	55.5	129
2019	17.9	56.5	62.7	138
Rate of change in deaths (% per year)				
2000-2010	-1.7	0.1	1.7	0.4
2010-2019	-0.8	0.7	1.4	0.8
2000-2019	-1.3	0.4	1.5	0.6
**DEATH RATE (PER 100 000 POPULATION PER YEAR)**				
2000	8.4	88.6	266	24.1
2010	6.2	63.2	219	22.1
2019	5.3	51.2	181	21.7
Rate of change in death rate (% per year)				
2000-2010	-3.0	-3.3	-1.9	-0.8
2010-2019	-1.7	-2.3	-2.1	-0.2
2000-2019	-2.4	-2.8	-2.0	-0.5

DATA SOURCE:

World Health Organization (WHO). Global Health Estimates, 2000-2021. Geneva: Department of Data and Analytics (DNA) Division of Data, Analytics and Delivery for Impact (DDI), WHO, 2024.

NOTE:

Rates of change between any two years are assumed to be constant. Hence the rate of change in the number of deaths will be the sum of the rate of change in the population size and the rate of change in the death rate, except for rounding.

TABLE A.16

Changes in deaths from selected NCD-7 conditions decomposed into changes in age group size and age group specific mortality rates, by CIH region, 2000-2010, 2010-2019, and 2000-2019.

Panel C7: Hemorrhagic stroke – Middle East and North Africa	Age group			
	15-49	50-69	70+	All ages
**POPULATION SIZE (MILLIONS)**				
2000	200	36.9	9.6	381
2010	259	53.6	13.8	467
2019	296	76.1	18.3	553
Rate of change in population size (% per year)				
2000-2010	2.6	3.8	3.7	2.0
2010-2019	1.5	4.0	3.2	1.9
2000-2019	2.1	3.9	3.5	2.0
**DEATHS (THOUSANDS)**				
2000	12.9	29.1	28.8	75.6
2010	11.7	27.6	32.4	73.9
2019	11.6	32.7	39.1	84.9
Rate of change in deaths (% per year)				
2000-2010	-1.0	-0.5	1.2	-0.2
2010-2019	-0.1	1.9	2.1	1.6
2000-2019	-0.6	0.6	1.6	0.6
**DEATH RATE (PER 100 000 POPULATION PER YEAR)**				
2000	6.5	79.0	300	19.8
2010	4.5	51.5	235	15.8
2019	3.9	43.0	213	15.3
Rate of change in death rate (% per year)				
2000-2010	-3.5	-4.2	-2.4	-2.2
2010-2019	-1.6	-2.0	-1.1	-0.3
2000-2019	-2.6	-3.1	-1.8	-1.3

DATA SOURCE:

World Health Organization (WHO). Global Health Estimates, 2000-2021. Geneva: Department of Data and Analytics (DNA) Division of Data, Analytics and Delivery for Impact (DDI), WHO, 2024.

NOTE:

Rates of change between any two years are assumed to be constant. Hence the rate of change in the number of deaths will be the sum of the rate of change in the population size and the rate of change in the death rate, except for rounding.

TABLE A.16

Changes in deaths from selected NCD-7 conditions decomposed into changes in age group size and age group specific mortality rates, by CIH region, 2000-2010, 2010-2019, and 2000-2019.

Panel C8: Hemorrhagic stroke – North Atlantic	Age group			
	15-49	50-69	70+	All ages
**POPULATION SIZE (MILLIONS)**				
2000	209	93.2	47.7	422
2010	211	107	57.5	446
2019	202	122	67.8	464
Rate of change in population size (% per year)				
2000-2010	0.1	1.4	1.9	0.6
2010-2019	-0.5	1.5	1.8	0.4
2000-2019	-0.2	1.4	1.9	0.5
**DEATHS (THOUSANDS)**				
2000	7.0	29.7	89.0	126
2010	5.2	21.9	88.6	116
2019	3.8	20.2	83.1	107
Rate of change in deaths (% per year)				
2000-2010	-3.0	-3.0	0.0	-0.8
2010-2019	-3.2	-0.9	-0.7	-0.9
2000-2019	-3.1	-2.0	-0.4	-0.8
**DEATH RATE (PER 100 000 POPULATION PER YEAR)**				
2000	3.4	31.8	187	29.8
2010	2.4	20.6	154	26.0
2019	1.9	16.6	123	23.1
Rate of change in death rate (% per year)				
2000-2010	-3.1	-4.3	-1.9	-1.4
2010-2019	-2.8	-2.4	-2.5	-1.3
2000-2019	-3.0	-3.4	-2.2	-1.3

DATA SOURCE:

World Health Organization (WHO). Global Health Estimates, 2000-2021. Geneva: Department of Data and Analytics (DNA) Division of Data, Analytics and Delivery for Impact (DDI), WHO, 2024.

NOTE:

Rates of change between any two years are assumed to be constant. Hence the rate of change in the number of deaths will be the sum of the rate of change in the population size and the rate of change in the death rate, except for rounding.

TABLE A.16

Changes in deaths from selected NCD-7 conditions decomposed into changes in age group size and age group specific mortality rates, by CIH region, 2000-2010, 2010-2019, and 2000-2019.

Panel C9: Hemorrhagic stroke – Sub-Saharan Africa	Age group			
	15-49	50-69	70+	All ages
**POPULATION SIZE (MILLIONS)**				
2000	313	52.5	11.8	682
2010	418	69.0	15.6	895
2019	544	93.4	20.4	1 140
Rate of change in population size (% per year)				
2000-2010	2.9	2.8	2.8	2.8
2010-2019	3.0	3.4	3.0	2.7
2000-2019	3.0	3.1	2.9	2.7
**DEATHS (THOUSANDS)**				
2000	30.0	106	78.9	223
2010	37.5	120	92.8	258
2019	44.0	141	111	302
Rate of change in deaths (% per year)				
2000-2010	2.3	1.3	1.6	1.5
2010-2019	1.8	1.8	2.0	1.8
2000-2019	2.0	1.5	1.8	1.6
**DEATH RATE (PER 100 000 POPULATION PER YEAR)**				
2000	9.6	201	668	32.6
2010	9.0	174	596	28.8
2019	8.1	151	543	26.5
Rate of change in death rate (% per year)				
2000-2010	-0.6	-1.4	-1.1	-1.2
2010-2019	-1.2	-1.6	-1.0	-0.9
2000-2019	-0.9	-1.5	-1.1	-1.1

DATA SOURCE:

World Health Organization (WHO). Global Health Estimates, 2000-2021. Geneva: Department of Data and Analytics (DNA) Division of Data, Analytics and Delivery for Impact (DDI), WHO, 2024.

NOTE:

Rates of change between any two years are assumed to be constant. Hence the rate of change in the number of deaths will be the sum of the rate of change in the population size and the rate of change in the death rate, except for rounding.

TABLE A.16

Changes in deaths from selected NCD-7 conditions decomposed into changes in age group size and age group specific mortality rates, by CIH region, 2000-2010, 2010-2019, and 2000-2019.

Panel C10: Hemorrhagic stroke – United States	Age group			
	15-49	50-69	70+	All ages
**POPULATION SIZE (MILLIONS)**				
2000	146	51.4	24.9	281
2010	151	71.3	27.5	311
2019	157	82.2	35.5	338
Rate of change in population size (% per year)				
2000-2010	0.4	3.3	1.0	1.0
2010-2019	0.5	1.6	2.9	0.9
2000-2019	0.4	2.5	1.9	1.0
**DEATHS (THOUSANDS)**				
2000	5.8	17.1	40.1	63.2
2010	4.8	18.7	36.3	60.0
2019	4.3	20.9	39.8	65.2
Rate of change in deaths (% per year)				
2000-2010	-2.0	0.9	-1.0	-0.5
2010-2019	-1.1	1.3	1.0	0.9
2000-2019	-1.6	1.1	0.0	0.2
**DEATH RATE (PER 100 000 POPULATION PER YEAR)**				
2000	4.0	33.3	161	22.4
2010	3.2	26.2	132	19.3
2019	2.7	25.5	112	19.3
Rate of change in death rate (% per year)				
2000-2010	-2.3	-2.4	-2.0	-1.5
2010-2019	-1.6	-0.3	-1.8	0.0
2000-2019	-2.0	-1.4	-1.9	-0.8

DATA SOURCE:
World Health Organization (WHO). Global Health Estimates, 2000-2021. Geneva: Department of Data and Analytics (DNA) Division of Data, Analytics and Delivery for Impact (DDI), WHO, 2024.

NOTE:
Rates of change between any two years are assumed to be constant. Hence the rate of change in the number of deaths will be the sum of the rate of change in the population size and the rate of change in the death rate, except for rounding.

TABLE A.16

Changes in deaths from selected NCD-7 conditions decomposed into changes in age group size and age group specific mortality rates, by CIH region, 2000-2010, 2010-2019, and 2000-2019.

Panel C11: Hemorrhagic stroke – Western Pacific and Southeast Asia	Age group			
	15-49	50-69	70+	All ages
**POPULATION SIZE (MILLIONS)**				
2000	492	127	38.9	934
2010	549	163	56.0	1 040
2019	582	205	74.4	1 130
Rate of change in population size (% per year)				
2000-2010	1.1	2.5	3.7	1.1
2010-2019	0.6	2.6	3.2	0.9
2000-2019	0.9	2.5	3.5	1.0
**DEATHS (THOUSANDS)**				
2000	68.2	222	220	515
2010	79.3	266	297	645
2019	77.0	305	333	719
Rate of change in deaths (% per year)				
2000-2010	1.5	1.8	3.0	2.3
2010-2019	-0.3	1.5	1.3	1.2
2000-2019	0.6	1.7	2.2	1.8
**DEATH RATE (PER 100 000 POPULATION PER YEAR)**				
2000	13.9	175	565	55.2
2010	14.4	163	531	61.9
2019	13.2	149	448	63.4
Rate of change in death rate (% per year)				
2000-2010	0.4	-0.7	-0.6	1.1
2010-2019	-1.0	-1.0	-1.8	0.3
2000-2019	-0.2	-0.8	-1.2	0.7

DATA SOURCE:
World Health Organization (WHO). Global Health Estimates, 2000-2021. Geneva: Department of Data and Analytics (DNA) Division of Data, Analytics and Delivery for Impact (DDI), WHO, 2024.

NOTE:
Rates of change between any two years are assumed to be constant. Hence the rate of change in the number of deaths will be the sum of the rate of change in the population size and the rate of change in the death rate, except for rounding.

TABLE A.17

Health financing indicators, rate of change over past decades

	AARC of GDP per person	AARC of CHE per person	AARC of GGHE-D per person	AARC of GGHE-D/CHE	AARC of OOP/ CHE
**2000-2010**					
**World average**					
Central and Eastern Europe					
Central Asia					
China					
India					
Latin America and Caribbean					
Middle East and North Africa					
North Atlantic					
Sub-Saharan Africa					
United States					
Western Pacific and Southeast Asia					
World average					

NOTES:
AARC = average annual rate of change; CHE = current health expenditure; GGHE-D = general government health expenditure from domestic sources; OOP = out-of-pocket spending. Source from WHO. Global Health Expenditure Database [Internet]. Available from: https://apps.who.int/nha/database (accessed August 26, 2024).

TABLE A.18

Health Benefit Package on Essential Medicines

NOTES:

Source: WHO's Health Benefit Package Survey (2020-2021). 97 countries provided at least one answer to the essential medicine related questions and have been included for analysis. Details about the survey methods can be found at: https://www.who.int/teams/health-financing-and-economics/economic-analysis/health-technology-

assessment-and-benefit-package-design/survey-homepage. In addition to “full coverage”, “partial coverage” and “no coverage”, some countries also reported “uncertain” or unanswered the relevant question(s). Therefore, the two columns not necessarily add up to 100%.

Several limitations need to be considered at the interpretation of the findings:

1) the survey only covers the largest (in terms of population coverage) publicly financed mechanism for each country. In other words, information may not be comparable across countries, especially when some countries reported a scheme covering the general population while some other countries reported a vertical program covering specific population only ;

2) coverage of essential medicine was reported by the survey respondents and information has not be verified through other channels.

TABLE A.19

Summary of literature on OOP for drugs

PUBLICATION DETAILS	DRUG EXPENDITURE AND FINANCIAL BURDEN
Liu et al, 2023<br>• China<br>• Essential medicine	The median affordability was equal to 0.88 (IQR: 2.58) days' wage of the lowest paid unskilled government worker, but steadily rose from 2006 to 2019. Subgroup analysis showed that the affordability in the western region (1.40, IQR: 2.88), urban area (0.95, IQR: 2.80), private sector (0.90, IQR: 2.30), of originator brands (OB) (2.90, IQR: 6.68), and antineoplastic and immunomodulating agents (5.68, IQR: 56.47) were worse than their counterparts.
Luiza et al, 2016<br>• Brazil	In about one of every 17 households (5.3%) catastrophic health expenditure was reported and, in 3.2%, the medicines were reported as one of the items responsible for this situation. The prevalence of households with catastrophic health expenditure and on medicines in relation to the total of households showed a regressive tendency for economic classes.
Haakenstad et al, 2022<br>• India	36.3% (95% uncertainty interval: 32.7–40.1) of explained variation in CHE is attributed to whether a private sector pharmacy was used and the number of drugs obtained. Of all outpatient visits, 13% are with a private sector chemist, a similar rate as public primary providers (15%). Insurance was used in just 6% of hospitalizations and its use explained just 0.2% (0.1–0.4) of CHE overall. Eighty-six percent of users of outpatient care obtained drugs from the private sector. We estimate that eliminating spending on private drugs would reduce CHE by 56% in Odisha.
Mekuria et al, 2023<br>• Ethiopia	Medicines accounted for the majority of healthcare spending (> 65%) across the surveys. From 2010 to 2016, the total percentage of households facing catastrophic medicine payments decreased from 1% to 0.73%. However, the actual number of people expected to have experienced catastrophic medicine payments increased from 399,174 to 401,519 people. Payment for medicines pushed 11,132 households into poverty in 2015/16. The majority of disparities were explained by economic status, place of residence, and type of health services.
Meririe et al, 2023<br>• Ethiopia<br>• Vaccine-preventable diseases	Households incurred drug expenditures contributed about 52% (42% to 60% depending on condition) of total expenditures.
Zarei et al, 2022<br>• Iran<br>• Type 2 Diabetes	The results show that expenditure on diabetes medication therapies in the form of mono-dual therapy and some cases triple oral therapies were not catastrophic even for rural households. Insulin puts patients at risk of catastrophic pharmaceutical expenditures when added to the treatment schedules, and lack of financial protection intensifies it. In general, the poorer households and those resistant to first-line treatments were at increased risk of catastrophic pharmaceutical expenditures.
Devine et al, 2023<br>• US<br>• Diabetes	An estimated 3 million Americans (10.3%) experienced out-of-pocket spending for antidiabetic drugs that reached catastrophic spending thresholds in 2020
Sengar et al, 2022<br>• Iran<br>• Cancer medicines	Risk of catastrophic expenditure was reported by 58%-67% of oncologists for rituximab and trastuzumab. Risks of financial toxicity were substantially higher within the private health system compared with the public system
Li et al, 2022<br>• US<br>• Cancer medicines	From 2011 to 2016, the uptake of targeted oral anticancer medicines (TOAMs) among our study population increased from 3.6% to 8.9%. The percentage of non-low-income subsidy TOAM users who reached catastrophic coverage increased from 54.6% to 60.3%. Among those who reached the catastrophic phase, mean total gross spending on TOAMs in the catastrophic phase increased from $16,074 (USD) to $64,233 (USD) and mean patient out-of-pocket spending from $596 (USD) to $2,549 (USD). The mean 30-day total spending increased from $4,011 (USD) to $8,857 (USD), and the mean 30-day out-of-pocket spending from $154 (USD) to $328 (USD).

NOTES:

1) Search was performed on PubMed with the following search terms: catastrophic health expenditure & drug; catastrophic pharmaceutical expenditure; household out-of-pocket & drug; financial burden & drug;

2) Selected studies: study reporting financial burden/out-of-pocket payment/catastrophic health expenditure that is directly attributed to drug (can be a specific drug or drugs in general) has been selected.

REFERENCES:

Haakenstad A, Kalita A, Bose B, Cooper JE, Yip W. Catastrophic health expenditure on private sector pharmaceuticals: a cross-sectional analysis from the state of Odisha, India. Health Policy Plan. 2022 Aug 3;37(7):872-884. doi: 10.1093/heapol/czac035.

Devine JW, Lim D, Lugo A, Farley JF. Prevalence and patterns of catastrophic spending for antidiabetic medication in 2020. J Manag Care Spec Pharm. 2023 Oct;29(10):1158-1164.

Li M, Liao K, Pan IW, Shih YT. Growing Financial Burden From High-Cost Targeted Oral Anticancer Medicines Among Medicare Beneficiaries With Cancer. JCO Oncol Pract. 2022 Nov;18(11):e1739-e1749.

Liu Z, Zou K, Liu D, Zhang M, Shi Y, Chen Z, Lang B, Cheng X, Li H, Zeng L, Tang Y, Zhao S, Choonara I, Jiang Y, Zhang L. The price and affordability of essential medicines, progress and regional distribution in China: a systematic review. Front Pharmacol. 2023 May 5;14:1153972.

Luiza VL, Tavares NU, Oliveira MA, Arrais PS, Ramos LR, Pizzol TD, Mengue SS, Farias MR, Bertoldi AD. Catastrophic expenditure on medicines in Brazil. Rev Saude Publica. 2016 Dec;50(suppl 2):15s.

Mekuria GA, Ali EE. The financial burden of out of pocket payments on medicines among households in Ethiopia: analysis of trends and contributing factors. BMC Public Health. 2023 May 3;23(1):808.

Memirie ST, Tolla MT, Rumpler E, Sato R, Bolongaita S, Tefera YL, Tesfaye L, Tadesse MZ, Getnet F, Mengistu T, Verguet S. Out-of-pocket expenditures and financial risks associated with treatment of vaccine-preventable diseases in Ethiopia: A cross-sectional costing analysis. PLoS Med. 2023 Mar 10;20(3):e1004198.

Sengar M, Fundytus A, Hopman W, Pramesh CS, Radhakrishnan V, Ganesan P, Mathew A, Lombe D, Jalink M, Gyawali B, Trapani D, Roitberg F, De

Vries EGE, Moja L, Ilbawi A, Sullivan R, Booth CM. Cancer Medicines: What Is Essential and Affordable in India? JCO Glob Oncol. 2022 Jul;8:e2200060.

Zarei L, Moradi N, Peiravian F, Hatami-Mazinani N, Mahi-Birjand M, Arabloo J, Babar ZU. Catastrophic pharmaceutical expenditure in patients with type 2 diabetes in Iran. Int J Equity Health. 2022 Dec 29;21(1):188

TABLE A.20

P-scores January 31, 2020, to May 4, 2023, and rank for all countries with a population greater than 5 million

Rank		P-score (%)
	World	12%
1	New Zealand	0.36
2	Central African Rep.	1.6
3	Dominican Rep.	2.9
4	Denmark	3.7
5	Taiwan	4.1
6	Japan	4.2
7	Guinea	4.2
8	Liberia	4.3
9	Côte d'Ivoire	4.3
10	Chad	4.6
11	Benin	4.7
12	Sierra Leone	4.7
13	South Sudan	4.7
14	Somalia	4.8
15	China	5.0
16	Australia	5.1
17	Nigeria	5.1
18	Congo	5.2
19	Niger	5.5
20	Togo	5.6
21	Angola	5.7
22	Mali	5.8
23	Burkina Faso	5.9
24	Norway	6.1
25	Turkmenistan	6.1
26	Sweden	6.4
27	Haiti	6.4
28	South Korea	6.5
29	Canada	6.6
30	Papua New Guinea	6.8
31	Mauritania	6.9
32	Madagascar	7.0
33	Malaysia	7.2
34	Burundi	7.2
35	Senegal	7.3
36	Singapore	7.3
37	France	7.3
38	Cameroon	7.4
39	Uzbekistan	7.4
40	Ghana	7.6
41	Germany	7.8
42	Finland	7.8
43	Congo DR	8.2
44	Yemen	8.3

TABLE A.20

P-scores January 31, 2020, to May 4, 2023, and rank for all countries with a population greater than 5 million

Rank		P-score (%)
45	Syria	8.3
46	Ireland	8.5
47	Kyrgyzstan	8.6
48	Belgium	8.9
49	Thailand	9.0
50	Costa Rica	9.1
51	Israel	9.4
52	Switzerland	10
53	China, Hong Kong	10
54	Greece	10
55	Venezuela	10
56	Portugal	10
57	Indonesia	10
58	Malawi	10
59	Netherlands	10
60	Austria	10
61	Palestine	11
62	Kenya	11
63	Rwanda	11
64	Mozambique	11
65	Sri Lanka	11
66	Spain	11
67	Tunisia	11
68	Myanmar	11
69	United Kingdom	11
70	Hungary	12
71	Oman	12
72	Ukraine	13
73	Zimbabwe	13
74	Tajikistan	13
75	Philippines	13
76	Lebanon	13
77	Tanzania	13
78	Czechia	13
79	Saudi Arabia	13
80	Italy	13
81	Morocco	14
82	Jordan	14
83	United States	14
84	Romania	14
85	Sudan	14
86	Pakistan	14
87	Poland	14
88	Azerbaijan	14
89	Ethiopia	15
90	Uganda	15
91	Zambia	15
92	Afghanistan	15

TABLE A.20

P-scores January 31, 2020, to May 4, 2023, and rank for all countries with a population greater than 5 million

Rank		P-score (%)
93	Chile	15
94	Cuba	15
95	South Africa	16
96	El Salvador	16
97	Nicaragua	16
98	Egypt	17
99	Viet Nam	17
100	Kazakhstan	18
101	Algeria	18
102	India	18
103	Slovakia	18
104	Türkiye	18
105	Guatemala	18
106	Brazil	18
107	Cambodia	18
108	Argentina	19
109	Serbia	19
110	Lao	19
111	Colombia	20
112	Bulgaria	20
113	Bolivia	20
114	Belarus	20
115	Iran	21
116	Nepal	21
117	Honduras	22
118	Paraguay	22
119	Russia	24
120	Bangladesh	25
121	Mexico	25
122	Iraq	27
123	Ecuador	28
124	Libya	30
125	United Arab Emirates	34
126	Peru	35

NOTES:

Estimated for the period starting on January 1, 2020, and concluding on May 4, 2023. (The World Health Organization (WHO) declared the end of the COVID-19 emergency phase on May 5, 2023.) P-scores are excess deaths divided by expected deaths in the same period.

Estimates for excess deaths are from The Economist – processed by Our World in Data. "Central estimate" Estimated excess mortality from The Economist. 2023. https://ourworldindata.org/excess-mortality-covid#estimated-excess-mortality-from-the-economist (accessed July 18, 2024). Estimates for total deaths are from United Nations, Department of Economic and Social Affairs, Population Division. World Population Prospects 2024, OnlineEdition.2024. https://population.un.org/wpp/Download/Standard/MostUsed/. We calculate the expected deaths used for the denominator as total deaths minus excess deaths (since The Economist – processed by Our World in Data. "Central estimate" Estimated excess mortality from The Economist. 2023. https://ourworldindata.org/excess-mortality-covid#estimated-excess-mortality-from-the-economist did not include expected deaths for all countries).

TABLE A.21

Excess deaths, excess deaths per million population, and p-scores for all countries January 31, 2020, to May 4, 2023

	Excess deaths (thousands)	Excess deaths per million population	P-score (%)
World	23,000	2,900	12%
Afghanistan	120	3,100	15
Albania	16	5,800	23
Algeria	120	2,600	18
American Samoa	0.08	1,500	7.6
Andorra	0.16	2,000	10
Angola	47	1,300	5.7
Anguilla	0.05	3,400	21
Antigua and Barbuda	-0.08	-910	-3.9
Argentina	210	4,600	19
Armenia	13	4,600	13
Aruba	0.47	4,300	15
Australia	29	1,100	5.1
Austria	28	3,100	10
Azerbaijan	33	3,300	14
Bahamas	1.5	3,700	13
Bahrain	3.1	2,000	33
Bangladesh	640	3,800	25
Barbados	0.6	2,000	6.2
Belarus	77	8,400	20
Belgium	32	2,800	8.9
Belize	0.7	1,900	11
Benin	18	1,400	4.7
Bermuda	0.32	4,900	20
Bhutan	-0.7	-960	-4.4
Bolivia	66	5,600	20
Bonaire, Sint Eustatius and Saba	0.18	6,400	31
Bosnia and Herzegovina	25	7,800	18
Botswana	4.9	2,100	10
Brazil	830	4,000	18
British Virgin Islands	0.09	2,300	13
Brunei Darussalam	0.43	950	5.4
Bulgaria	71	10,000	20
Burkina Faso	34	1,600	5.9
Burundi	21	1,600	7.2
Cabo Verde	0.9	1,800	11
Cambodia	57	3,300	18
Cameroon	49	1,800	7.4
Canada	66	1,700	6.6
Cayman Islands	0.15	2,100	15
Central African Rep.	7.6	1,500	1.6
Chad	31	1,800	4.6
Chile	64	3,300	15

TABLE A.21

Excess deaths, excess deaths per million population, and p-scores for all countries January 31, 2020, to May 4, 2023

	Excess deaths (thousands)	Excess deaths per million population	P-score (%)
China	1,700	1,200	5.0
China, Hong Kong	17	2,300	10
China, Macao SAR	1.4	2,000	14
Taiwan	25	1,100	4.1
Colombia	190	3,700	20
Comoros	1.5	1,800	7.7
Congo	6.5	1,100	5.2
Congo DR	230	2,300	8.2
Cook Islands	0.01	710	2.6
Costa Rica	8.5	1,700	9.1
Croatia	26	6,700	16
Cuba	58	5,200	15
Curaçao	0.7	4,000	14
Cyprus	2.1	1,600	7.2
Czechia	49	4,700	13
Côte d'Ivoire	33	1,100	4.3
Denmark	6.8	1,200	3.7
Djibouti	1.2	1,100	4.2
Dominica	0.10	1,500	3.5
Dominican Rep.	6.7	600	2.9
Ecuador	83	4,700	28
Egypt	340	3,100	17
El Salvador	23	3,700	16
Equatorial Guinea	1.5	860	3.2
Eritrea	6.3	1,900	10
Estonia	5.3	4,000	11
Eswatini	2.5	2,100	7.2
Ethiopia	330	2,700	15
Falkland Islands (Malvinas)	0.00	1,000	7.3
Faroe Islands	0.05	1,000	2.9
Fiji	0.8	870	2.7
Finland	14	2,600	7.8
France	150	2,300	7.3
French Guiana	1.3	4,300	34
French Polynesia	0.7	2,400	21
Gabon	2.7	1,100	5.3
Gambia	3.7	1,500	6.9
Georgia	25	6,700	18
Germany	240	2,900	7.8
Ghana	56	1,700	7.6
Gibraltar	0.09	2,500	12
Greece	40	3,700	10
Greenland	-0.11	-2,000	-5.9
Grenada	0.16	1,400	4.8
Guadeloupe	2.1	5,200	18
Guam	0.9	5,300	29

TABLE A.21

Excess deaths, excess deaths per million population, and p-scores for all countries January 31, 2020, to May 4, 2023

	Excess deaths (thousands)	Excess deaths per million population	P-score (%)
Guatemala	53	3,000	18
Guernsey	0.27	4,300	16
Guinea	18	1,300	4.2
Guinea-Bissau	3.2	1,500	6.4
Guyana	3.2	3,900	16
Haiti	19	1,700	6.4
Holy See	0.00	3,500	8.0
Honduras	31	3,000	22
Hungary	50	5,100	12
Iceland	0.35	940	4.4
India	5,400	3,800	18
Indonesia	730	2,600	10
Iran	290	3,300	21
Iraq	140	3,300	27
Ireland	9.1	1,800	8.5
Isle of Man	0.23	2,700	8.4
Israel	14	1,600	9.4
Italy	280	4,700	13
Jamaica	5.6	2,000	7.5
Japan	200	1,600	4.2
Jersey	0.6	5,400	17
Jordan	16	1,400	14
Kazakhstan	81	4,100	18
Kenya	130	2,500	11
Kiribati	0.10	750	3.0
Kosovo (under UNSC res. 1244)	4.9	2,800	14
Kuwait	7.4	1,700	24
Kyrgyzstan	12	1,800	8.6
Lao	25	3,400	19
Latvia	11	5,900	12
Lebanon	15	2,600	13
Lesotho	4.1	1,800	4.9
Liberia	6.0	1,100	4.3
Libya	30	4,300	30
Liechtenstein	0.04	1,100	4.6
Lithuania	26	9,200	21
Luxembourg	0.14	220	0.9
Madagascar	51	1,700	7.0
Malawi	37	1,800	10
Malaysia	42	1,200	7.2
Maldives	0.7	1,400	19
Mali	38	1,700	5.8
Malta	0.9	1,800	7.6
Marshall Islands	0.05	1,300	5.7
Martinique	2.2	6,400	19
Mauritania	6.2	1,300	6.9

TABLE A.21

Excess deaths, excess deaths per million population, and p-scores for all countries January 31, 2020, to May 4, 2023

	Excess deaths (thousands)	Excess deaths per million population	P-score (%)
Mauritius	2.3	1,800	6.1
Mayotte	0.9	3,200	42
Mexico	670	5,300	25
Micronesia (Fed. States of)	0.24	2,200	9.0
Monaco	0.27	6,900	10
Mongolia	1.2	350	1.6
Montenegro	3.9	6,400	17
Montserrat	0.01	1,200	3.0
Morocco	91	2,500	14
Mozambique	82	2,600	11
Myanmar	170	3,200	11
Namibia	3.8	1,300	5.4
Nauru	0.00	240	1.1
Nepal	130	4,200	21
Netherlands	52	2,900	10
New Caledonia	0.29	1,000	4.5
New Zealand	0.44	86	0.36
Nicaragua	18	2,700	16
Niger	40	1,600	5.5
Nigeria	430	2,000	5.1
Niue	0.00	600	1.2
North Macedonia	17	9,300	27
Northern Mariana Islands	0.16	3,400	32
Norway	8.2	1,500	6.1
Oman	4.1	910	12
Pakistan	690	2,900	14
Palau	0.00	110	0.30
Palestine	6.3	1,200	11
Panama	10	2,200	14
Papua New Guinea	15	1,500	6.8
Paraguay	28	4,200	22
Peru	220	6,500	35
Philippines	290	2,500	13
Poland	190	5,000	14
Portugal	37	3,500	10
Puerto Rico	19	5,700	19
Qatar	1.5	540	19
Republic of Moldova	19	6,300	15
Romania	120	6,300	14
Russia	1,400	9,500	24
Rwanda	26	2,000	11
Réunion	4.6	5,300	37
Saint Helena	-0.00	-110	-0.22
Saint Kitts and Nevis	0.21	4,500	14
Saint Lucia	0.47	2,700	9.2
Saint Martin (French part)	0.11	3,700	12

TABLE A.21

Excess deaths, excess deaths per million population, and p-scores for all countries January 31, 2020, to May 4, 2023

	Excess deaths (thousands)	Excess deaths per million population	P-score (%)
Saint Pierre and Miquelon	0.00	840	1.9
Saint Vincent and the Grenadines	0.22	2,100	5.5
Samoa	0.06	260	1.3
San Marino	0.15	4,300	17
Sao Tome and Principe	0.24	1,100	5.6
Saudi Arabia	31	1,000	13
Senegal	24	1,400	7.3
Serbia	61	8,900	19
Seychelles	-0.03	-240	-0.9
Sierra Leone	11	1,300	4.7
Singapore	6.0	1,100	7.3
Sint Maarten (Dutch part)	0.18	4,400	20
Slovakia	32	5,900	18
Slovenia	6.6	3,100	9.5
Solomon Islands	0.9	1,100	6.5
Somalia	30	1,800	4.8
South Africa	280	4,600	16
South Korea	70	1,400	6.5
South Sudan	16	1,500	4.7
Spain	160	3,400	11
Sri Lanka	53	2,300	11
Sudan	130	2,800	14
Suriname	2.0	3,200	15
Sweden	19	1,800	6.4
Switzerland	22	2,500	10
Syria	28	1,300	8.3
Tajikistan	20	2,000	13
Tanzania	150	2,300	13
Thailand	160	2,300	9.0
Timor-Leste	1.8	1,300	5.6
Togo	13	1,400	5.6
Tokelau	-0.00	-88	-3.3
Tonga	0.02	220	1.0
Trinidad and Tobago	5.1	3,500	13
Tunisia	28	2,400	11
Turkmenistan	8.2	1,200	6.1
Turks and Caicos Islands	0.11	2,400	11
Tuvalu	0.01	1,100	3.4
Türkiye	300	3,400	18
Uganda	100	2,200	15
Ukraine	240	5,400	13
United Arab Emirates	10	980	34
United Kingdom	230	3,400	11
United States	1,300	3,900	14
United States Virgin Islands	0.5	6,200	13
Uruguay	7.9	2,300	7.1

TABLE A.21

Excess deaths, excess deaths per million population, and p-scores for all countries January 31, 2020, to May 4, 2023

	Excess deaths (thousands)	Excess deaths per million population	P-score (%)
Uzbekistan	50	1,400	7.4
Vanuatu	-0.13	-430	-2.3
Venezuela	63	2,200	10
Viet Nam	310	3,100	17
Wallis and Futuna Islands	0.01	500	2.1
Western Sahara	0.7	1,200	6.1
Yemen	53	1,400	8.3
Zambia	52	2,600	15
Zimbabwe	48	3,100	13

NOTES:

Excess deaths are estimated for the period starting on January 1, 2020, and concluding on May 4, 2023. (The World Health Organization (WHO) declared the end of the COVID-19 emergency phase on May 5, 2023.) P-scores are excess deaths divided by expected deaths in the same period.

Estimates for excess deaths are from The Economist – processed by Our World in Data. "Central estimate" Estimated excess mortality from The Economist. 2023. https://ourworldindata.org/excess-mortality-covid#estimated-excess-mortality-from-the-economist (accessed July 18, 2024). Estimates for total deaths are from WPP24 United Nations, Department of Economic and Social Affairs, Population Division. World Population Prospects 2024,OnlineEdition.2024.https://population.un.org/wpp/Download/Standard/MostUsed/.

We calculate the expected deaths used for the denominator for the p-scores as total deaths minus excess deaths (since The Economist – processed by Our World in Data. "Central estimate" Estimated excess mortality from The Economist. 2023. https://ourworldindata.org/excess-mortality-covid#estimated-excess-mortality-from-the-economist did not include expected deaths for all countries). Population data from United Nations, Department of Economic and Social Affairs, Population Division. World Population Prospects 2024, OnlineEdition.2024. https://population.un.org/wpp/Download/Standard/MostUsed/ (accessed July 14, 2024).

TABLE A.22

Components of an early intersectoral package of policy instruments

Key health risk	Policy	Instrument
**AIR POLLUTION**	1. Indoor air pollution: subsidize other clean household energy sources, including liquid propane gas (LPG), for the poor and other key populations.	Fiscal
	2. Indoor air pollution: halt the use of unprocessed coal and kerosene as a household fuel.	Regulatory
	3. Indoor air pollution: promote the use of low-emission household devices.	Information and education
	4. Emissions: tax emissions and/or auction off transferable emission permits.	Fiscal
	5. Emissions: regulate transport, industrial, and power generation emissions.	Regulatory
	6. Fossil fuel subsidies: dismantle subsidies for and increase taxation of fossil fuels (except LPG).	Fiscal
	7. Public transportation: build and strengthen affordable public transportation systems in urban areas.	Built environment
**ADDICTIVE SUBSTANCE USE**	8. Substance use: impose large excise taxes on tobacco, alcohol, and other addictive substances.	Fiscal
	9. Substance use: impose strict regulation of advertising, promotion, packaging, and availability of tobacco, alcohol, and other addictive substances, with enforcement.	Regulatory
	10. Smoking in public places: ban smoking in public places.	Regulatory
**INADEQUATE NUTRIENT INTAKE**	11. School feeding: finance school feeding for all schools and students in selected geographical areas.	Fiscal
	12. Food quality: ensure that subsidized foods and school feeding programs have adequate nutritional quality.	Regulatory
	13. Iron and folic acid: fortify food.	Regulatory
	14. Iodine: fortify salt.	Regulatory
**EXCESSIVE NUTRIENT INTAKE**	15. Trans fats: ban and replace with polyunsaturated fats.	Regulatory
	16. Salt: impose regulations to reduce salt in manufactured food products.	Regulatory
	17. Sugar sweetened beverages: tax to discourage use.	Fiscal
	18. Salt and sugar: provide consumer education against excess use, including product labeling.	Information and education
**ROAD TRAFFIC INJURIES**	19. Vehicle safety: enact legislation and enforcement of personal transport safety measures, including seatbelts in vehicles and helmets for motorcycle users.	Regulatory
	20. Traffic safety: set and enforce speed limits on roads.	Regulatory
	21. Traffic safety: include traffic calming mechanisms into road construction.	Built environment
**OTHER RISKS**	22. Pesticides: enact strict control and move to selective bans on highly hazardous pesticides.	Regulatory

TABLE A.22

Components of an early intersectoral package of policy instruments

Key health risk	Policy	Instrument
	23. Water and sanitation: enact national standards for safe drinking water, sanitation, and hygenic behavior within and outside households and institutions.	Regulatory
	24. Hazardous waste: enact legislation and enforcement of standards for hazardous waste disposal.	Regulatory
	25. Lead exposure: take actions to reduce human exposure to lead, including bans on leaded fuels and on lead in paint, cookware, water pipes, cosmetics, drugs, and food supplements.	Regulatory
	26. Agricultural antibiotic use: reduce and eventually phase out subtherapeutic antibiotic use in agriculture.	Regulatory
	27. Emergency response: create and exercise multisectoral responses and supply stockpiles to respond to pandemics and other emergencies.	Regulatory
	28. Safe sex: remove duties and taxes on condoms, then introduce subsidies in brothels and for key at-risk populations.	Fiscal
	29. Exercise: take initial steps to develop infrastructure enabling safe walking and cycling.	Built environment

NOTES:

The table show an early intersectoral package which draws on policy interventions that have been determined to have the strongest evidence and the highest likely magnitude of health effect.

SOURCE:

Disease Control Priorities: Improving Health and Reducing Poverty. 3rd edition.

TABLE A.23

Notable and potential launches of vaccines, therapeutics, and diagnostics to address I-8 and NCD-7 conditions

Disease	Notable launches between 2000 and 2024*	Potential launches between 2025 and 2040*
**IMH8**		
Childhood cluster diseases	PEDIARIX – a combination vaccine indicated for active immunization against diphtheria, tetanus, pertussis, infection caused by all known subtypes of hepatitis B virus, and poliomyelitis; can be given between six weeks and six years of age.	ProQuad – a vaccine indicated for active immunization against measles, mumps, rubella, and varicella in children 12 months through 12 years of age.
Diarrheal diseases	Rotasiil – the first thermostable rotavirus vaccine capable of long-term storage at ambient temperatures below 25 degrees Celsius.	ZF0901 – a bivalent conjugate vaccine for the prevention Shigella infection. The vaccine contains O-specific polysaccharides from S. flexneri 2a and S. sonnei.<br><br>CV638 – a live attenuated vaccine for the prevention of Vibrio cholera infection which has demonstrated a protective immune response in clinical studies.
HIV/AIDS	Azvudine – a novel nucleoside reverse transcriptase inhibitor with greater effectiveness against drug resistant HIV strains compared to standards of care.<br><br>HIV 1/2 antibody (anti-HIV Ab) RDT – one of the first rapid diagnostic tests for HIV.	Ad26.Mos4.HIV, Clade C gp140 and Mosaic gp140 HIV bivalent vaccine – a mosaic-based protein developed using adenovirus vector platform and C6 production cell line technology, contains mosaic-based immunogens capable of producing an immune response to a wide verity of HIV-1 subtypes. The vaccine regimen is delivered in four vaccinations in one year, with the first two delivered in a single injection.<br><br>Islatravir – a nucleoside reverse transcriptase translocation inhibitor which prevents HIV from multiplying by blocking the reverse transcriptase HIV enzyme. Islatravir is being developed and trialed as a fixed-dose combination with doravirine and as a stand-alone therapy.
Lower respiratory infections	Prevenar 13 – a thirteen-valent pneumococcal conjugate vaccine with higher effectiveness against all-cause otitis media compared to other pneumococcal conjugate vaccines.<br><br>Abrysvo – the first vaccine for pregnant individuals to prevent RSV in infants.	MCDA-LFB – ultra-efficient multiple cross displacement amplification-lateral flow biosensor for serogroup identification of Neisseria meningitidis.
Maternal conditions	Pitocin – a synthetic oxytocin for the treatment of postpartum hemorrhage.<br><br>NovoSeven – a hemostatic recombinant activated factor VIIa for the treatment of severe blood loss from postpartum hemorrhage.	Enalapril – an angiotensin converting enzyme (ACE) inhibitor for the management of postpartum hypertension and improvement of postpartum cardiovascular function in patients with severe preeclampsia.<br><br>Metoprolol – a beta-1 blocker for the treatment of high blood pressure in preeclampsia.
Malaria	Mosquirix – the first malaria vaccine approved for public use.<br><br>Coartem – one of the first artemisinin-based combination therapies for the treatment of malaria.	Artemether/Lumefantrine/Amodiaquine – therapeutic combination has been shown to be a viable alternative to Artemether/Lumefantrine alone for first-line treatment of multidrug-resistant P falciparum malaria.

TABLE A.23

Notable and potential launches of vaccines, therapeutics, and diagnostics to address I-8 and NCD-7 conditions

Disease	Notable launches between 2000 and 2024*	Potential launches between 2025 and 2040*
Neonatal conditions	Albuterol – a bronchodilator for the treatment of bronchospasm in preterm neonates.	Betamethasone – a long-acting corticosteroid with immunosuppressive and anti-inflammatory properties for fetal lung maturation in preterm birth.<br>Pentoxifylline – a synthetic dimethylxanthine derivative for the treatment of impaired fetal growth.
Tuberculosis	Bedaquiline – treatment for multidrug resistant tuberculosis with a unique mechanism of action that inhibits the mycobacterial ATP synthase proton pump.<br><br>Xpert MTB/RIF Assay – a novel integrated diagnostic device for the diagnosis of tuberculosis and rapid detection of rifampin resistance.	MTBVAC – the first and only live attenuated vaccine derived from a human isolate of Mycobacterium tuberculosis.<br><br>Tubivac – the world's first orally available therapeutic vaccine used as an immune adjunct for tuberculosis chemotherapy. Taken in conjunction with standard tuberculosis medications, it reduces treatment time for the condition to one month.
**NCDI7**		
Atherosclerotic cardiovascular diseases	Colchicine – an alkaloid tablet to reduce the risk of myocardial infarction, stroke, coronary revascularization, and cardiovascular death in patients with atherosclerotic disease or other risk factors for cardiovascular disease.<br><br>Bempedoic acid – an adenosine triphosphate-citrate lyase inhibitor to reduce risk of myocardial infarction and coronary revascularization in adults with cardiovascular disease who are unable to take statin therapies.	Adult and pediatric hydrochlorothiazide combinations – hydrochlorothiazide is a common treatment for hypertension; patents on hydrochlorothiazide combination therapies are soon to expire thereby increasing affordability and access in low- and middle-income countries.
Diabetes	Afrezza (insulin human) Inhalation Powder – the only inhaled, quick acting insulin commercially available.<br><br>Insulin degludec (Tresiba) – long-acting insulin analog administered through subcutaneous injection for the treatment of hyperglycemia.	Semaglutide – an antidiabetic medication for type 2 diabetes and long-term weight management; soon to come off patent allowing for the development of cheaper biosimilars that will widen access.
Hemorrhagic stroke	Esmolol – an intravenous cardioselective β-1 adrenergic antagonist for blood pressure management.	Recombinant factor VIIa – an intravenously administered blood factor undergoing phase III trials for the treatment of hemorrhagic stroke.
NCDs strongly linked to infections	PreHevbrio – the first and only 3-antigen Hepatitis B vaccine.<br><br>Gardasil – the first vaccine for the prevention of human papillomavirus infection.	Lenvervimab – a recombinant human immunoglobulin for the treatment of chronic hepatitis B virus infection.<br><br>Antaitavir Hasophate and Yiqibuvir – ongoing phase III clinical trials to confirm efficacy and safety for the treatment of chronic hepatitis C in adult patients.
NCDs strongly linked to tobacco use	Repotrectinib – a kinase inhibitor for the treatment of ROS1-positive non-small cell lung cancer and NTRK-positive locally advanced or metastatic solid tumors.<br><br>Roflumilast – orally administered tablet to reduce risk of COPD exacerbations in patients with severe COPD associated with chronic bronchitis.	MK-7684A – a fixed dose pembrolizumab/vibostolimab co-formulation with chemotherapy as a first line treatment for small cell lung cancer.
Road Injury	Forward collision warning and advanced emergency breaking – advanced driver assistance systems designed to reduce the risk and severity of collisions.	Vehicle-to-vehicle communication – use of wireless ad hoc networks to permit autonomous sharing and processing of data between vehicles thereby mitigating human error and reducing road collisions.

TABLE A.23

Notable and potential launches of vaccines, therapeutics, and diagnostics to address I-8 and NCD-7 conditions

Disease	Notable launches between 2000 and 2024*	Potential launches between 2025 and 2040*
Suicide	Clozapine – the first and only medication with an indication for schizophrenia associated suicide prevention.<br><br>Esketamine – an oral antidepressant for treatment-resistant depression and depressive symptoms in adults with major depressive disorder who experience suicidal thoughts or behaviors.	NRX-101 – a fixed dose combination oral capsule of D-cycloserine and lurasidone for the maintenance of remission from severe bipolar depression with acute suicidal ideation or behavior.

TABLE A.24

Expanded list of notable past and potential number of future launches of vaccines, therapeutics, and diagnostics to address I-8 and NCD-7 conditions.

Disease	Notable launches between 2000 and 2024	Potential launches between 2025 and 2040
**IMH8**		
Childhood cluster diseases	Diphtheria & Tetanus Toxoids & Acellular Pertussis Vaccine Adsorbed (DAPTACEL)[1]<br>Diphtheria & Tetanus Toxoids & Acellular Pertussis Vaccine Adsorbed, Hepatitis B (recombinant) and Inactivated Poliovirus Vaccine Combined (Pediarix) [1]<br>Diphtheria and Tetanus Toxoids and Acellular Pertussis Adsorbed and Inactivated Poliovirus Vaccine (KINRIX) [1]<br>Diphtheria and Tetanus Toxoids and Acellular Pertussis Adsorbed and Inactivated Poliovirus Vaccine (Quadracel) [1]<br>Diphtheria and Tetanus Toxoids and Acellular Pertussis Adsorbed, Inactivated Poliovirus, Haemophilus b Conjugate [Meningococcal Protein Conjugate] and Hepatitis B [Recombinant] Vaccine (VAXELIS) [1]<br>Diphtheria and Tetanus Toxoids and Acellular Pertussis Adsorbed, Inactivated Poliovirus and Haemophilus b Conjugate (Tetanus Toxoid Conjugate) Vaccine (Pentacel) [1]<br>Measles, Mumps and Rubella Vaccine, Live (PRIORIX) [1]<br>Measles, Mumps, and Rubella Virus Vaccine, Live (M-M-R II) [1]<br>Measles, Mumps, Rubella and Varicella Virus Vaccine Live (ProQuad) [1]	
Diarrheal diseases	Four vaccines for cholera and four vaccines for rotavirus, one therapeutic for cryptosporidiosis, two diagnostics for cholera, two diagnostics for cryptosporidiosis, four diagnostics for giardiasis, and three diagnostics for multiple diarrheal diseases.[2]<br>Examples: Rotasiil – first thermostable rotavirus vaccine capable of long-term storage at ambient temperatures below 25 degrees Celsius.[2]<br>Nitazoxanide – a broad spectrum antiparasitic for treatment of cryptosporidiosis.2 Cholera RT-PCR – allows for more rapid and reliable detection of Vibrio cholerae.[2]	Between 2025 and 2030 we expect two simple vaccines for rotavirus, zero therapeutics, two diagnostic assays for cholera, one diagnostic assay for multiple diarrheal diseases, and one diagnostic platform technology for shigella.[12] Between 2031 and 2040 we expect zero vaccines, zero therapeutics, and zero diagnostics.[12]<br>Examples: ZF0901 – a bivalent conjugate vaccine for the prevention Shigella infection. The vaccine contains O-specific polysaccharides from S. flexneri 2a and S. sonnei.[12] CV638 – a live attenuated vaccine for the prevention of Vibrio cholera infection which has demonstrated a protective immune response in clinical studies.[12]
HIV/AIDS	Two therapeutics for HIV/AIDS and ten diagnostics for HIV/AIDS.[2]<br>Examples: Azvudine – a novel nucleoside reverse transcriptase inhibitor with greater effectiveness against drug resistant strains compared to other	Between 2025 and 2030 we expect zero vaccines, two simple repurposed drugs, and eight diagnostic assays.[12] Between 2031 and 2040 we expect three complex vaccines, two complex biologics, and zero diagnostics.[12]

TABLE A.24

Expanded list of notable past and potential number of future launches of vaccines, therapeutics, and diagnostics to address I-8 and NCD-7 conditions.

	standards of care.[2] HIV 1/2 antibody (anti-HIV Ab) RDT – first rapid diagnostic test for HIV.[2]	Examples: Ad26.Mos4.HIV, Clade C gp140 and Mosaic gp140 HIV bivalent vaccine – a mosaic-based protein developed using adenovirus vector platform and C6 production cell line technology, contains mosaic-based immunogens capable of producing an immune response to a wide verity of HIV-1 subtypes. The vaccine regimen is delivered in four vaccinations in one year, with the first two delivered in a single injection.[12] Islatravir – a nucleoside reverse transcriptase translocation inhibitor which prevents HIV from multiplying by blocking the reverse transcriptase HIV enzyme. Islatravir is being developed and trialed as a fixed-dose combination with doravirine and as a stand-alone therapy.[12]
Lower respiratory infections	Two vaccines for pneumonia3, seventeen vaccines for influenza[4], and two vaccines for respiratory syncytial virus.[5]<br><br>Examples: Prevenar 13 – first thirteen-valent pneumococcal conjugate vaccine; higher effectiveness against all-cause otitis media compared to other pneumococcal conjugate vaccines.[3] FluMist – a live attenuated influenza vaccine in the form of a nasal spray.[4] Abrysvo – first vaccine for pregnant individuals to prevent RSV in infants.[5]	Between 2025 and 2030 we expect three simple vaccines for pneumonia, zero therapeutics, and four diagnostic assays for pneumonia.[12] Between 2031 and 2040 we expect zero vaccines, zero therapeutics, and zero diagnostics.[12]<br><br>Examples: MCDA-LFB – ultra-efficient multiple cross displacement amplification-lateral flow biosensor for serogroup identification of Neisseria meningitidis.[12]
Maternal conditions	Pitocin – a synthetic oxytocin for the treatment of postpartum hemorrhage.[6]<br><br>NovoSeven – a hemostatic recombinant activated factor VIIa for the treatment of severe blood loss from postpartum hemorrhage.[6]	Between 2025 and 2030 we expect zero vaccines, five simple repurposed drugs for intrauterine growth restriction, five simple repurposed drugs for maternal enteric microbiome, sixteen simple repurposed drugs for preeclampsia/eclampsia, twelve simple repurposed drugs for preterm birth, fifteen diagnostic assays for intrauterine growth restriction, 29 diagnostic assays for preeclampsia/eclampsia, and twenty-two diagnostic assays for preterm birth.[12] Between 2031 and 2040 we expect zero vaccines, one simple new chemical entity for preeclampsia/eclampsia, and one simple new chemical entity for preterm birth.[12]<br><br>Enalapril – an angiotensin converting enzyme (ACE) inhibitor for the management of postpartum hypertension and improvement of postpartum cardiovascular function in patients with severe preeclampsia.12 Metoprolol – a beta-1 blocker for the treatment of high blood pressure in preeclampsia.[12]
Malaria	Two vaccines for malaria, eighteen therapeutics for malaria, and four diagnostics for malaria.[2]<br><br>Examples: Mosquirix – first malaria vaccine approved for public use.[2] Coartem – one of the first artemisinin-based combination therapies for treatment of malaria.2 Plasmodium spp. multiple antigens test.[2]	Between 2025 and 2030 we expect zero vaccines, zero therapeutics, six diagnostic assays for multiple malaria strains, five diagnostic platform technologies for multiple malaria strains, five diagnostic assays for P. falciparum, and five diagnostic assays for P. vivax.[12] Between 2031 and 2040 we expect two complex vaccines for P. falciparum, one simple new chemical entity for multiple malaria strains, and zero diagnostics.[12]<br><br>Examples: Artemether/Lumefantrine/Amodiaquine – therapeutic combination has been shown to be a viable alternative to Artemether/Lumefantrine

TABLE A.24

Expanded list of notable past and potential number of future launches of vaccines, therapeutics, and diagnostics to address I-8 and NCD-7 conditions.

		alone for first-line treatment of multidrug-resistant P falciparum malaria.[12]
Neonatal conditions	Albuterol – a bronchodilator for the treatment of bronchospasm in preterm neonates.[7]	Between 2025 and 2030 we expect zero vaccines, twelve simple repurposed drugs for preterm birth, and twenty-two diagnostic assays for preterm birth.[12] Between 2031 and 2040 we expect zero vaccines, one simple new chemical entity for preterm birth, and zero diagnostics.[12]<br>Examples: Betamethasone – a long-acting corticosteroid with immunosuppressive and anti-inflammatory properties for fetal lung maturation in preterm birth.[12] Pentoxifylline – a synthetic dimethylxanthine derivative for the treatment of impaired fetal growth.[12]
Tuberculosis	Seven therapeutics for tuberculosis and eleven diagnostics for tuberculosis.[2]<br>Examples: Bedaquiline – treatment for multidrug resistant tuberculosis with a unique mechanism of action that inhibits the mycobacterial ATP synthase proton pump.2 Xpert MTB/RIF Assay – novel integrated diagnostic device for the diagnosis of tuberculosis and rapid detection of rifampin resistance.[2]	Between 2025 and 2030 we expect two complex vaccines, seven simple repurposed drugs, twenty-six diagnostic assays, and six diagnostic platform technologies.[12] Between 2031 and 2040 we expect zero vaccines, zero therapeutics, and zero diagnostics.[12]<br>Examples: MTBVAC – the first and only live attenuated vaccine derived from a human isolate of Mycobacterium tuberculosis.[12] Tubivac – the world's first orally available therapeutic vaccine used as an immune adjunct for tuberculosis chemotherapy. Taken in conjunction with standard tuberculosis medications, it reduces treatment time for the condition to one month.[12]
**NCDI7**		
Atherosclerotic cardiovascular diseases	Colchicine – an alkaloid tablet to reduce the risk of myocardial infarction, stroke, coronary revascularization, and cardiovascular death in patients with atherosclerotic disease or other risk factors for cardiovascular disease.[8]<br>Bempedoic acid – an adenosine triphosphate-citrate lyase inhibitor to reduce risk of myocardial infarction and coronary revascularization in adults with cardiovascular disease who are unable to take statin therapies.[8]	Adult and pediatric hydrochlorothiazide combinations – hydrochlorothiazide is a common treatment for hypertension; patents on hydrochlorothiazide combination therapies are soon to expire thereby increasing affordability and access in low- and middle-income countries.[13]
Diabetes	Afrezza (insulin human) Inhalation Powder[8]<br>Lantidra (donislecel-jujn)[8]<br>Lantus (insulin glargine) injection[8]<br>Lyumjev (insulin lispro-aabc injection, 100 units/mL and 200 units/mL)[8]<br>Novolog MIX 70/30 (insulin aspart protamine and insulin aspart)[8]<br>Symlin (pramlintide)[8]<br>Tresiba (insulin degludec injection)[8]<br>Tzield (teplizumab-mzwv)[8]	Semaglutide – an antidiabetic medication for type 2 diabetes and long-term weight management; soon to come off patent allowing for the development of cheaper biosimilars that will widen access.[14]
Hemorrhagic stroke	Esmolol – an intravenous cardioselective β-1 adrenergic antagonist for blood pressure management.[9]	Recombinant factor VIIa – an intravenously administered blood factor undergoing phase III trials for the treatment of hemorrhagic stroke.[15]

TABLE A.24

Expanded list of notable past and potential number of future launches of vaccines, therapeutics, and diagnostics to address I-8 and NCD-7 conditions.

NCDs strongly linked to infections	Two vaccines for Hepatitis B1, thirteen therapeutics for gastric cancer[8], eleven therapeutics for liver cancer[8], and five therapeutics for cervical cancer.[8]<br><br>Examples: PreHevbrio – first and only 3-antigen Hepatitis B vaccine.1 Everolimus – an antineoplastic agent that inhibits the mammalian target of rapamycin.8 Pembrolizumab – a programmed death receptor-1 blocking antibody.8 Bevacizumab – a vascular endothelial growth factor inhibitor.[8]	Between 2025 and 2030 we expect zero vaccines, one simple new chemical entity for Hepatitis C, and seven diagnostic assays for Hepatitis C.[12] Between 2031 and 2040 we expect zero vaccines, two complex biologics for Hepatitis B, and zero diagnostics.[12]<br><br>Examples: Lenvervimab – a recombinant human immunoglobulin for the treatment of chronic hepatitis B virus infection.12 Antaitavir Hasophate and Yiqibuvir – ongoing phase III clinical trials to confirm efficacy and safety for the treatment of chronic hepatitis C in adult patients.[12]
NCDs strongly linked to tobacco use	Twenty-eight therapeutics for lung cancer, and fifteen therapeutics for chronic obstructive pulmonary disorder.[8]<br><br>Examples: Repotrectinib – a kinase inhibitor for the treatment of ROS1-positive non-small cell lung cancer and NTRK-positive locally advanced or metastatic solid tumors.[8] Roflumilast – orally administered tablet to reduce risk of COPD exacerbations in patients with severe COPD associated with chronic bronchitis.[8]	MK-7684A – a fixed dose pembrolizumab/vibostolimab co-formulation with chemotherapy as a first line treatment for small cell lung cancer.[16]
Road Injury	Forward collision warning and advanced emergency breaking – advanced driver assistance systems designed to reduce the risk and severity of collisions.[10]	Vehicle-to-vehicle communication – use of wireless ad hoc networks to permit autonomous sharing and processing of data between vehicles thereby mitigating human error and reducing road collisions.[17]
Suicide	Clozapine – the first and only medication with an indication for schizophrenia associated suicide prevention.[11]<br><br>Esketamine – an oral antidepressant for treatment-resistant depression and depressive symptoms in adults with major depressive disorder who experience suicidal thoughts or behaviors.[11]	NRX-101 – a fixed dose combination oral capsule of D-cycloserine and lurasidone for the maintenance of remission from severe bipolar depression with acute suicidal ideation or behavior.[16]

REFERENCES:

1. https://www.fda.gov/vaccines-blood-biologics/vaccines/vaccines-licensed-use-united-states
2. https://impact.policycuresresearch.org/
3. https://www.ncbi.nlm.nih.gov/pmc/articles/PMC4778694/
4. https://www.ncbi.nlm.nih.gov/pmc/articles/PMC7223957/
5. https://www.ncbi.nlm.nih.gov/pmc/articles/PMC10609699/
6. https://www.ncbi.nlm.nih.gov/books/NBK294453
7. https://www.ncbi.nlm.nih.gov/pmc/articles/PMC8867519
8. https://www.centerwatch.com/directories/1067-fda-approved-drugs
9. https://www.ncbi.nlm.nih.gov/books/NBK294453
10. https://www.ncbi.nlm.nih.gov/pmc/articles/PMC8431415/
11. https://www.ncbi.nlm.nih.gov/pmc/articles/PMC10172553/
12. https://centerforpolicyimpact.org/our-work/research-and-development/
13. https://www.ahajournals.org/doi/full/10.1161/CIRCULATIONAHA.115.008722
14. https://www.nature.com/articles/d41586-024-02044-x
15. https://www.ncbi.nlm.nih.gov/pmc/articles/PMC9933458/
16. https://clinicaltrials.gov/
17. https://www.ncbi.nlm.nih.gov/pmc/articles/PMC7864337/

TABLE A.25

Innovative candidate vaccines, therapeutics, and diagnostics that are likely to launch between 2025 and 2040

Disease condition	Number of products	Product type	Selected Phase 3 candidates	Source
HIV/AIDS	The HIV/AIDS pipeline contains over 50 products with 13 candidates in the preclinical phase, 33 in phase 1, 8 in phase 2, and 1 product in phase 3	VACCINES	Ad26.Mos4.HIV, Clade C gp140 and Mosaic gp140 HIV bivalent vaccine - a mosaic-based protein developed using adenovirus vector platform and C6 production cell line technology, contains mosaic-based immunogens capable of producing an immune response to a wide verity of HIV-1 subtypes. The vaccine regimen is delivered in four vaccinations in one year, with the first two delivered in a single injection.	https://www.jnj.com/media-center/press-releases/johnson-johnson-announces-new-clinical-data-on-mosaic-based-hiv-preventive-vaccine-regimen
		DRUGS	(1) Islatravir, a nucleoside reverse transcriptase translocation inhibitor which prevents HIV from multiplying by blocking the reverse transcriptase HIV enzyme. Islatravir is being developed trialed as a fixed-dose combination with doravirine and as a stand-alone therapy. Phase 2b results showed treatment regimens containing islatravir and doravirine showed antiviral efficacy (2) VM1500A, a non-nucleoside reverse transcriptase inhibitor also being evaluated in for HIV treatment in combination with other drugs. (3) Semzuvolimab, a CD4 attachment inhibitors being trialed for HIV management in patients who are resistant to routine anti-HIV drugs.	https://www.thelancet.com/journals/lanhiv/article/PIIS2352-3018(21)00021-7/abstract, https://clinicalinfo.hiv.gov/en/drugs/ub-421/patient, https://www.viriom.com/news/2020/5/4/viriom-announces-initiation-of-phase-2-study-investigating-efficacy-of-intramuscular-long-acting-injectable-nanoformulation-of-vm1500a-in-hiv-infected-patients
		DIAGNOSTICS	NA	
Tuberculosis	The tuberculosis pipeline contains over 100 candidates with 25 in preclinical phase, 10 in phase 1, 40 in phase 2, and 33 in phase 3 across the product range	VACCINES	(1) MTBVAC stands as the first and only live attenuated vaccine derived from a human isolate of Mycobacterium tuberculosis. (2) GamTBvac is a recombinant vaccine made up of three Mycobacterium tuberculosis (MTB) antigens.	https://www.iavi.org/press-release/zendal-and-iavi-announce-expanded-agreement-to-partner-on-development-of-tb-vaccine-candidate-mtbvac/
		DRUGS	Tubivac: The world's first orally available therapeutic vaccine used as an immune adjunct for tuberculosis chemotherapy. Taken in conjunction with standard tuberculosis medications, it reduces treatment time for the condition to one month.	https://www.tbonline.info/posts/2020/2/1/immunitor-unveils-breakthrough-immunotherapy-treat/

TABLE A.25

Innovative candidate vaccines, therapeutics, and diagnostics that are likely to launch between 2025 and 2040

		DIAGNOSTICS	NA	
Malaria	The malaria pipeline contains over 100 candidates with 33 in preclinical phase, 31 in phase 1, 22 in phase 2, and 10 in phase 3 across the product range	VACCINES	R21/Matrix-M vaccine for malaria prevention has been approved by the WHO. The vaccine reduces symptomatic cases of malaria by 75% in the first year following a 3-dose series.	https://www.who.int/news/item/02-10-2023-who-recommends-r21-matrix-m-vaccine-for-malaria-prevention-in-updated-advice-on-immunization
		DRUGS	Artemether/Lumefantrine/Amodiaquine combination has been shown to be a viable alternative to Artemether/Lumefantrine alone for first-line treatment of multidrug-resistant P falciparum malaria.	https://www.thelancet.com/journals/laninf/article/PIIS1473-3099(21)00692-7/fulltext
		DIAGNOSTICS	NA	
Bacterial pneumonia & meningitis	There are over 30 products in the bacterial pneumonia & meningitis disease pipeline. 15 products in the preclinical phase and 8 in phase 3.	VACCINES	Six simple vaccines in phase 3 development.	
		DRUGS	NA	
		DIAGNOSTICS	MCDA-LFB: Ultra-efficient multiple cross displacement amplification-lateral flow biosensor for serogroup identification of Neisseria meningitidis. LEC-LAMP N: Loop-Primer Endonuclease Cleavage–Loop-Mediated Isothermal Amplification Technology for multiplex pathogen detection and SNP identification.	https://www.sciencedirect.com/science/article/pii/S1525157820300404, https://pubmed.ncbi.nlm.nih.gov/35623396/

FIGURE A.1

Global and frontier probability of premature death (PPD), 1950–2021

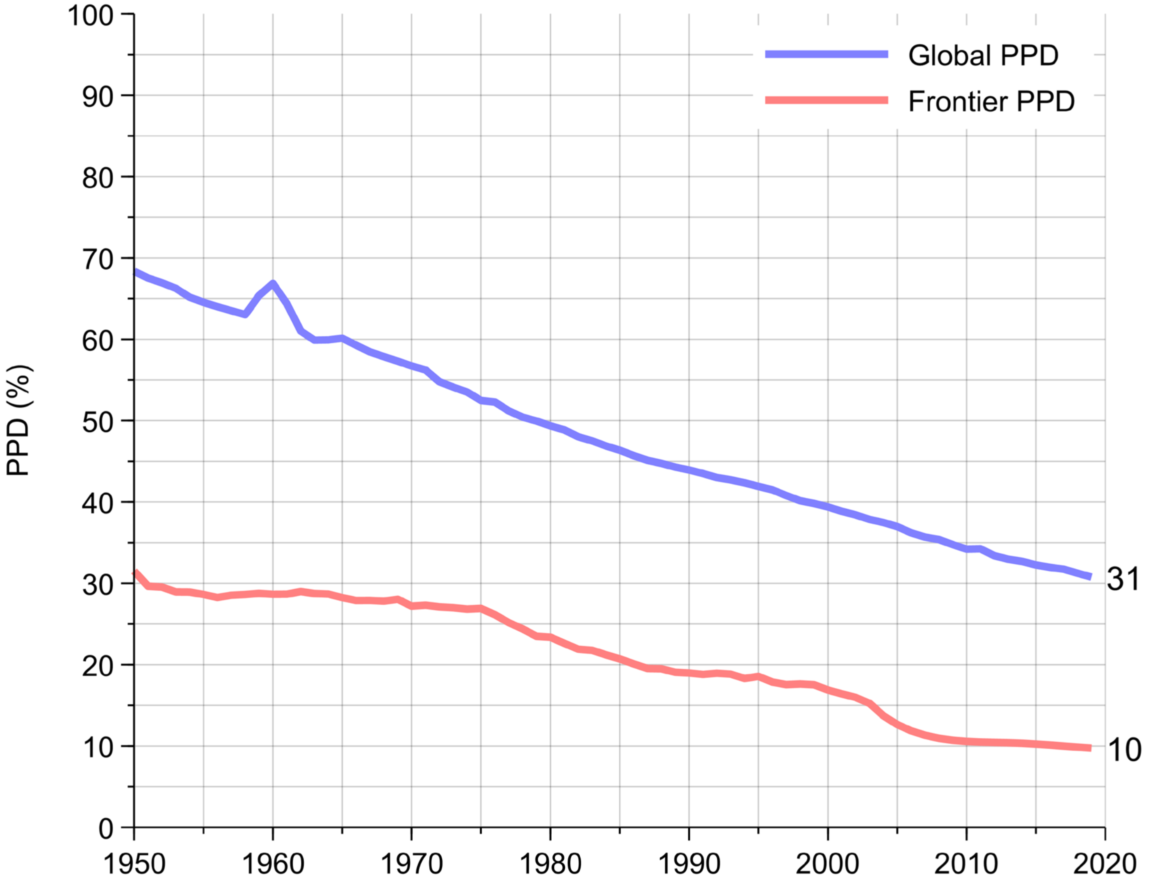

Probability of premature death (PPD) was defined as dying before age 70 years. The frontier is the lowest PPD observed each year. Countries with a population below 3 million in 2019 were not considered for being a frontier.

Adapted from CIH background paper Karlsson O, Jamison DT, Yamey G, Bolongaita S, Mao W, Chang AY, Norheim OF, Ogbuoji O, Verguet S. Probability of death before age 70: progress as years behind or ahead of the global average trend. Available from: https://www.uib.no/sites/w3.uib.no/files/attachments/karlsson_et_al._probability_of_death_before_70.pdf (accessed September 12, 2024).

Data from United Nations. World Population Prospects 2024, Online Edition. 2024.; Available from: https://population.un.org/wpp/Download/Standard/MostUsed/ (accessed July 14, 2024).

FIGURE A.2

Sex differences in the rate of decline in PPD, 30 most populous countries, 2010-19

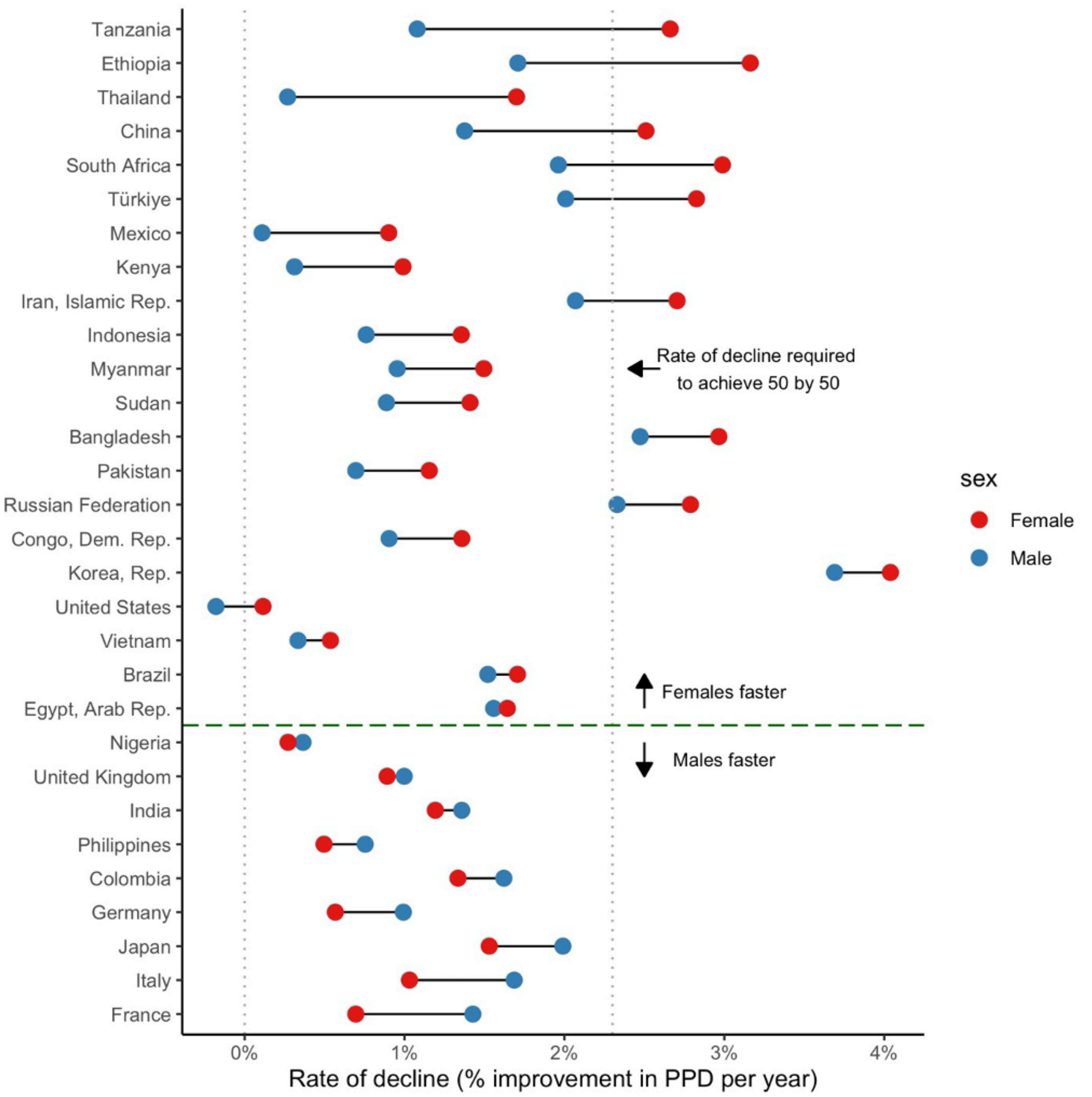

SOURCE:

United Nations. World Population Prospects 2024, Online Edition. 2024.; Available from: https://population.un.org/wpp/Download/Standard/MostUsed/ (accessed July 14, 2024).

FIGURE A.3

Current levels of government tax revenue and health spending, by country

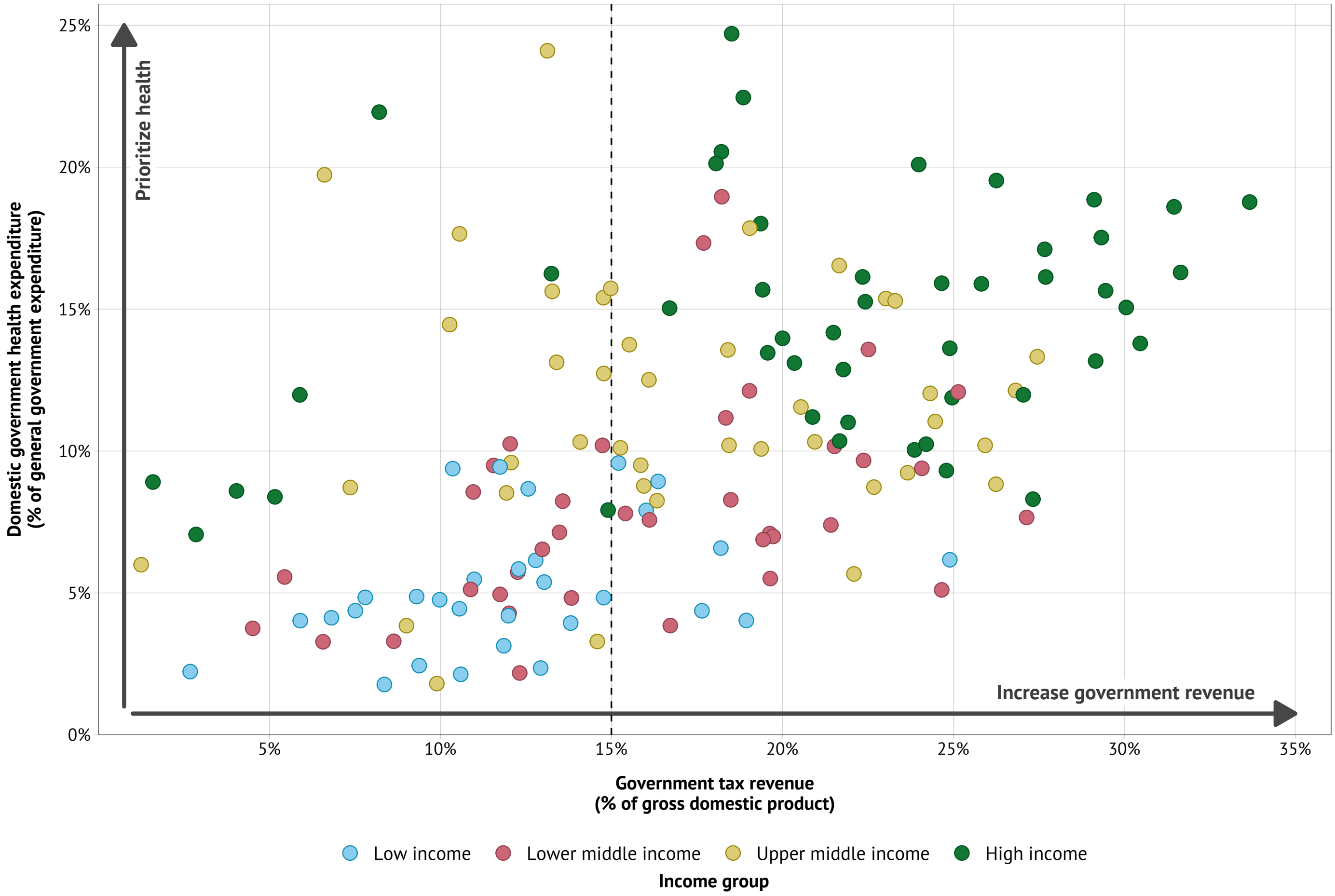

Note: Data from World Bank. World Development Indicators | DataBank [Internet]. Available from: https://databank.worldbank.org/source/world-development-indicators (accessed August 26, 2024). The most recent values for each country with complete data (n=126) are plotted here.

FIGURE A4

Out-of-pocket payments as share of current health expenditure (%)

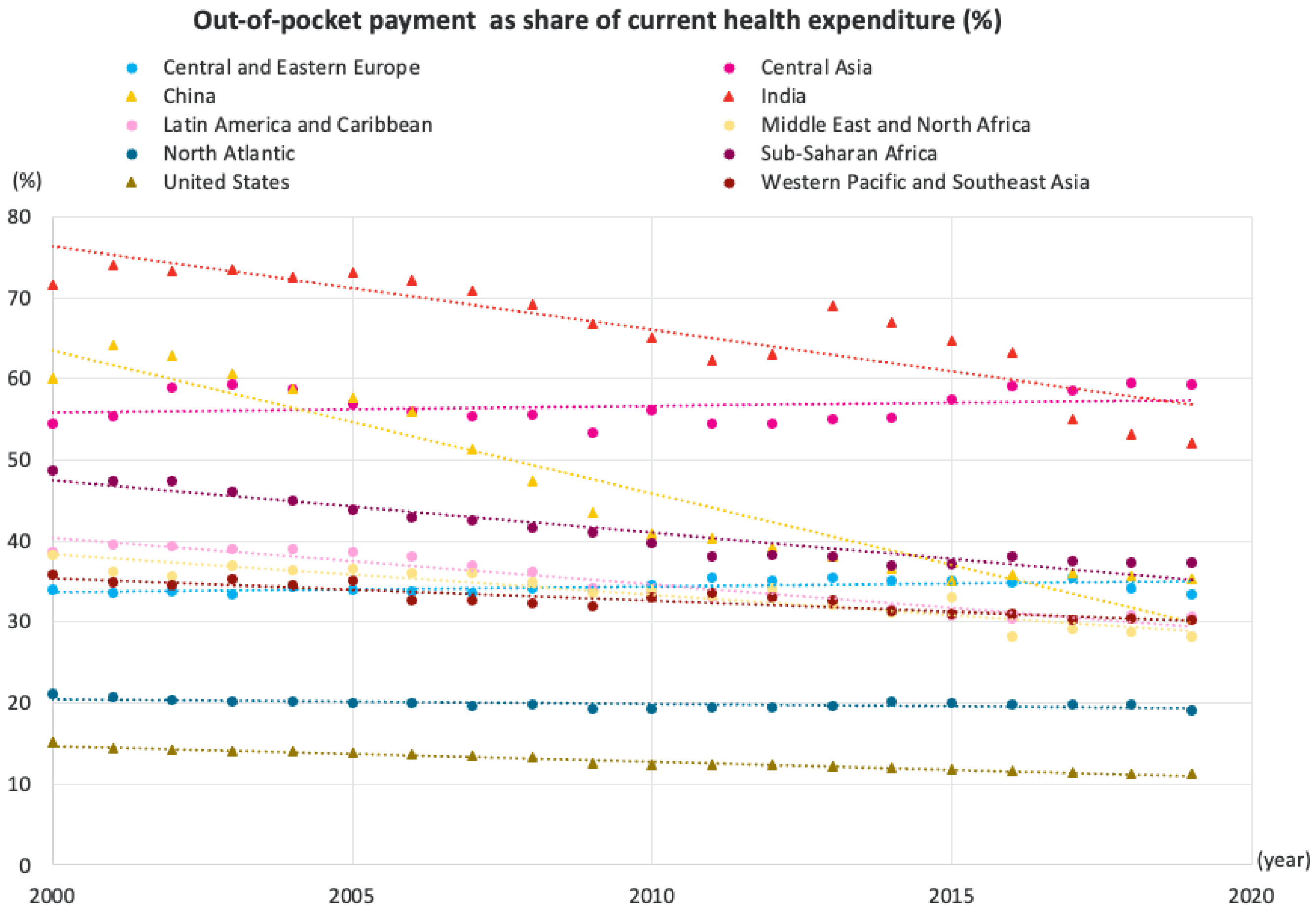

Data source: WHO. Global Health Expenditure Database [Internet]. [cited 2024 July 9]. Available from: https://apps.who.int/nha/database

FIGURE A5

Government health expenditure and out-of-pocket payment per person (%)

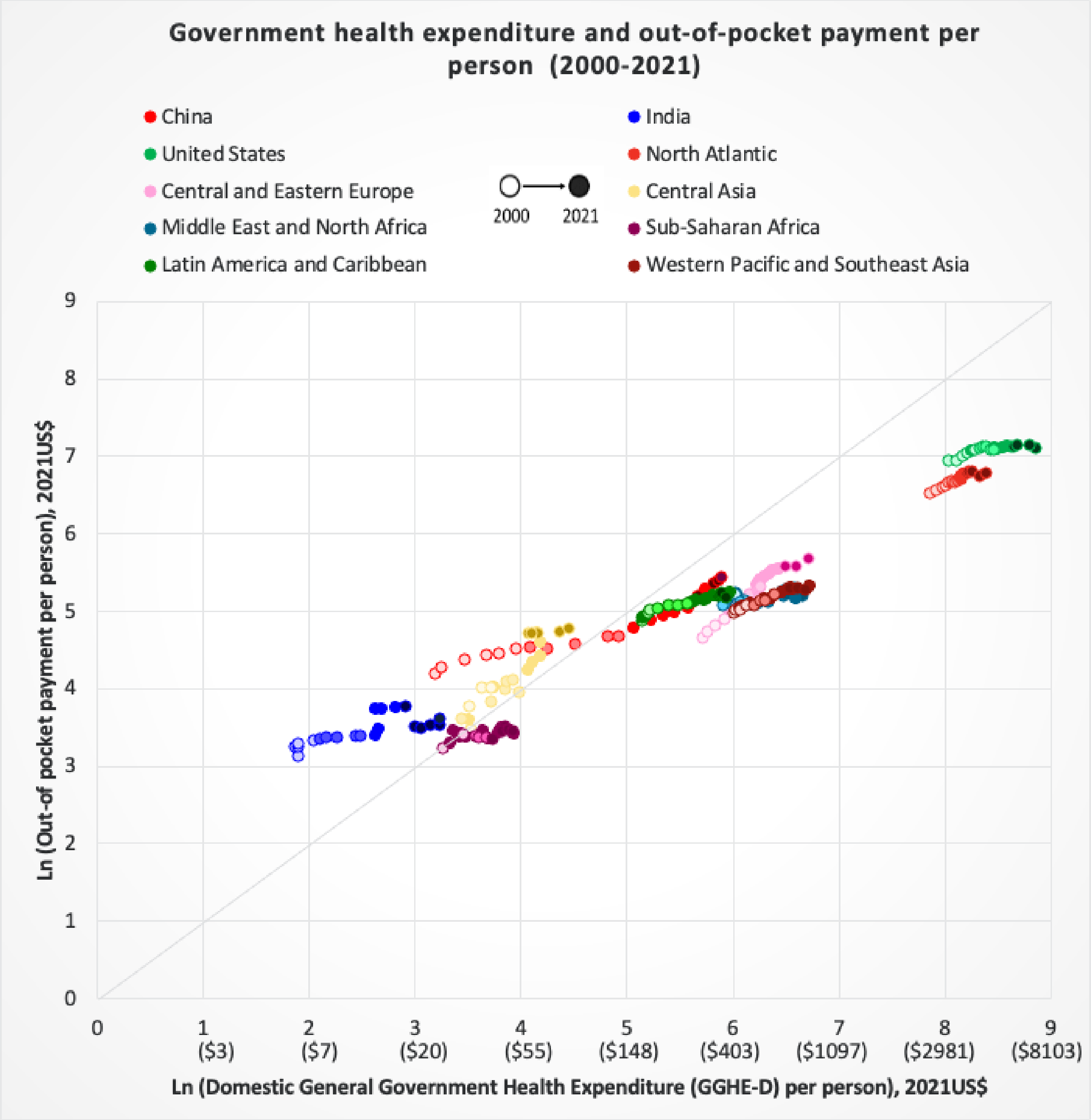

DATA SOURCE:

WHO. Global Health Expenditure Database [Internet]. [cited 2024 July 9]. Available from: https://apps.who.int/nha/database

FIGURE A6

P-scores by month: United States, Italy, Japan, and China – April 2020 to October 2023 (7-month moving averages)

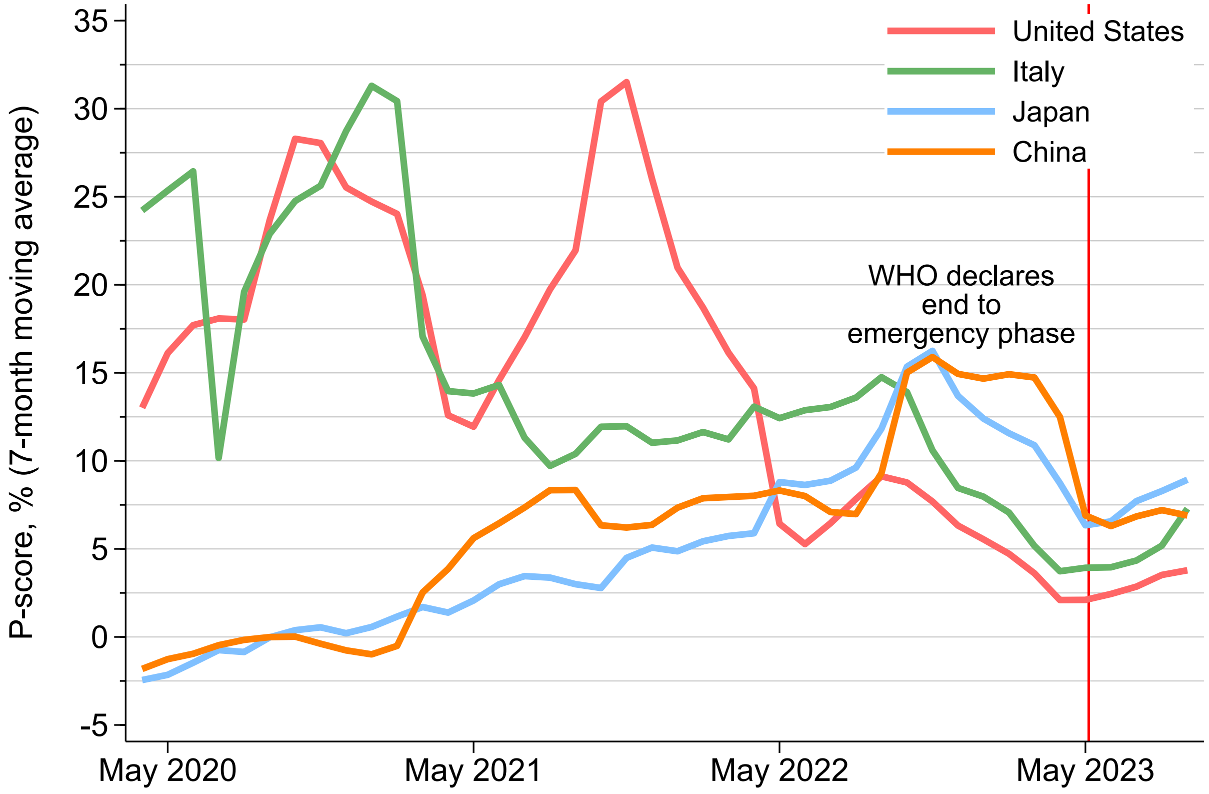

P-scores are excess deaths divided by expected deaths in the same period.

Estimates for excess deaths are from The Economist – processed by Our World in Data. "Central estimate" Estimated excess mortality from The Economist. 2023. https://ourworldindata.org/excess-mortality-covid#estimated-excess-mortality-from-the-economist (accessed July 18, 2024).

Estimates for total deaths are from United Nations, Department of Economic and Social Affairs, Population Division. World Population Prospects 2024, Online Edition. 2024. https://population.un.org/wpp/Download/Standard/MostUsed/ (accessed July 14, 2024)

We calculate the expected deaths used for the denominator as total deaths (divided by 12 as death are over a year) minus excess deaths (since The Economist – processed by Our World in Data. "Central estimate" Estimated excess mortality from The Economist. 2023. https://ourworldindata.org/excess-mortality-covid#estimated-excess-mortality-from-the-economist) did not include expected deaths for all countries.

FIGURE A7

Correlation between May 4 2023 cumulative p-scores and excess deaths per 1000 population for the 30 most populous countries, January 1, 2020, to May 4, 2023

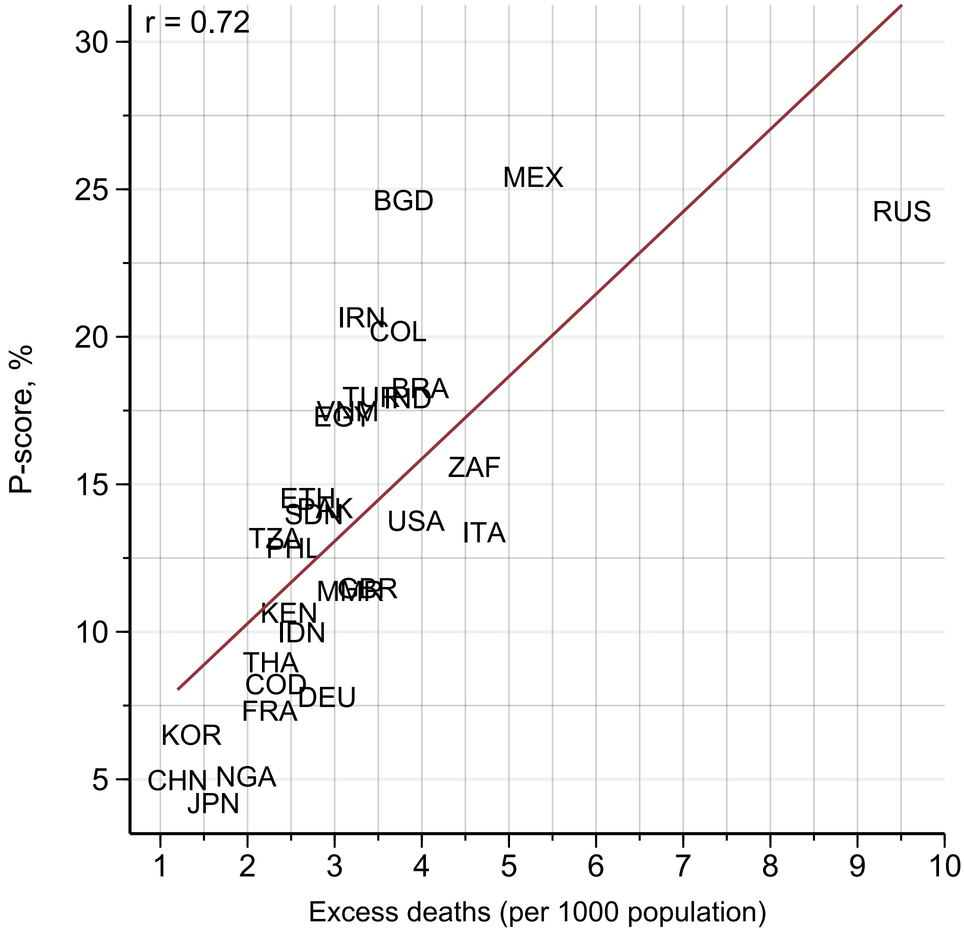

Pearson's correlation coefficient (r) is shown. Countries were equally weighted. Excess deaths are estimated for the period starting on January 1, 2020, and concluding on May 4, 2023. (The World Health Organization (WHO) declared the end of the COVID-19 emergency phase on May 5, 2023.) P-scores are excess deaths divided by expected deaths in the same period.

Estimates for excess deaths are fromThe Economist – processed by Our World in Data. "Central estimate" Estimated excess mortality from The Economist. 2023. https://ourworldindata.org/excess-mortality-covid#estimated-excess-mortality-from-the-economist (accessed July 18, 2024).

Estimates for total deaths are from United Nations, Department of Economic and Social Affairs, Population Division. World Population Prospects 2024, Online Edition. 2024. https://population.un.org/wpp/Download/Standard/MostUsed/ (accessed July 14, 2024).

We calculate the expected deaths used for the denominator for the p-scores as total deaths minus excess deaths (since The Economist – processed by Our World in Data. "Central estimate" Estimated excess mortality from The Economist. 2023. https://ourworldindata.org/excess-mortality-covid#estimated-excess-mortality-from-the-economist did not include expected deaths for all countries). Population data from United Nations, Department of Economic and Social Affairs, Population Division. World Population Prospects 2024, Online Edition. 2024. https://population.un.org/wpp/Download/Standard/MostUsed/ (accessed Jul 14, 2024).

PANEL A.1

Projecting temperature-related mortality impacts from climate change

Quantifying and projecting the health impacts of climate change is important both for planning climate adaptation and evaluating mitigation strategies.[1,2] However, generating accurate estimates of these impacts is difficult due to three major practical and conceptual challenges.

First, climate change can affect health through a complex web of causal pathways, making it challenging to comprehensively capture the full health burden. Second, there are uncertainties in extrapolating from current climate-health relationships to future ones, including the potential limits of individuals and societies to adaptation.[3] Third, there are difficulties in quantifying ambiguous risks where there is limited historical precedent and understanding of complex biophysical relationships. These risks include tipping points, defined by the Intergovernmental Panel on Climate Change (IPCC) as "critical thresholds in a system that, when exceeded, can lead to a significant change in the state of the system, often with an understanding that the change is irreversible,"[4] as well as the potential effects of climate change on war and mass migration.

Despite these difficulties, several multi-disciplinary consortia have recently attempted to robustly quantify the mortality impacts of climate change.[5–7] They aimed to update the social cost of carbon (SCC)—the net economic impact of one additional tonne of carbon dioxide emissions—which is widely used to evaluate climate policies.8 Mortality impacts, which have historically represented about 5% of the SCC,5 are an order of magnitude higher in these updated studies—for example, around 50% in Rennert and colleagues' study,[7] which was specific to the social cost of carbon dioxide.

The primary pathway that these recent studies evaluated is the effect of heat on mortality and, conversely, the benefit from reduced cold-induced mortality. Their focus on this particular pathway reflects its strong empirical grounding and perceived significance[6,7] – in contrast to pathways through extreme weather events that are more uncertain and, to a certain degree, unknowable.[9] Different pathways differ also in the extent to which compensatory behaviour can reduce mortality impact through that pathway.

These studies broadly involve two steps: first, assessing the current relationship between heat and mortality and, second, projecting this relationship in a hotter world.[5–7,10] Exposure-response relationships are projected at a national and, increasingly, sub-national scale using climate models and scenarios developed for the Intergovernmental Panel on Climate Change.[5–7,10] There is a "U-shaped" relationship, indicating an increased relative risk of mortality at low and high temperatures.[6,10] Notably, larger effect sizes are observed in poorer, more densely populated, and warmer regions.[6,10]

The IPCC laid out four scenarios for greenhouse gas emissions, called Representative Concentration Pathways: a stringent mitigation scenario (RCP2.6), two intermediate scenarios (RCP4.5 and RCP6.0),

and a scenario with very high emissions (RCP8.5). Despite using different datasets and methodological approaches, the projections generated by the new studies under RCP4.5, i.e., a global temperature rise of about 2.4°C by 2100, are similar, with annual excess deaths ranging between 1.0 and 1.7 million in 2100 (Figure A). Under RCP 8.5, i.e. a global temperature rise of around 4.3°C by 2100, projections in the different studies diverge, ranging between 2.4 and 7.3 million annual excess deaths in 2100. This divergence reflects differences in the studies' assumptions, including the shape of the relationship between mortality risk and temperature[6,7] and the inclusion of climate models accounting for low-likelihood, extreme climate outcomes.[6]

Panel A.1 Figure A:

Projected increase in annual heat-related deaths due to climate change compared to the 2015 baseline

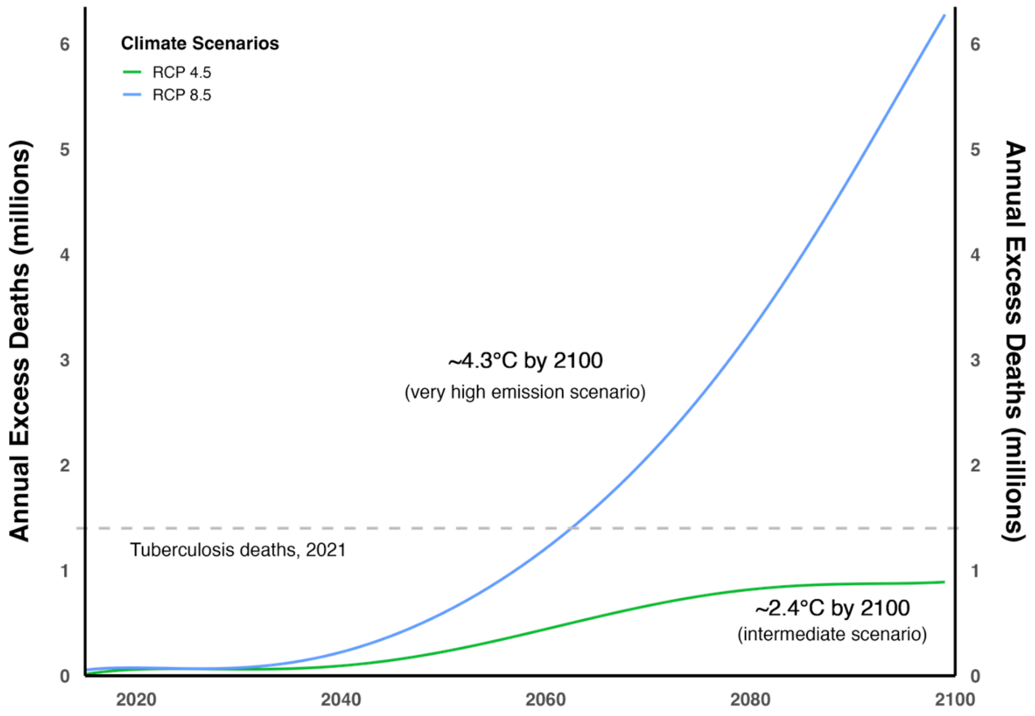

NOTE: The graph presents estimates under two Representative Concentration Pathways (RCPs): RCP 4.5, a ~2.4°C increase in global temperatures by 2100 compared to 1850-1900, and RCP 8.5, a ~4.3°C increase, as per the IPCC Fifth Assessment Report from Intergovernmental Panel on Climate Change (IPCC). Climate Change 2013 – The Physical Science Basis: Working Group I Contribution to the Fifth Assessment Report of the Intergovernmental Panel on Climate Change. Cambridge: Cambridge University Press; 2014. Available from: https://www.cambridge.org/core/books/climate-change-2013-the-physical-science-basis/BE9453E500DEF3640B383BADDC332C3E (accessed July 8, 2024).11 As of 2024, global temperatures have already risen between 1.25°C and 1.5°C from 1850-1900 (Hausfather, 2024).

Source from projections adapted from Carleton T, Jina A, Delgado M. Valuing the Global Mortality Consequences of Climate Change Accounting for Adaptation Costs and Benefits. Q J Econ. 2022;137:2037–1056

While there is significant uncertainty in the impacts, and relatively modest estimated impacts by 2050, these findings add to other arguments for limiting global temperature rise in line with the Paris Agreement—the international treaty to limit temperature rise to below 1.5-2°C, whose success will be critically shaped by decisions taken this decade.12 In the nearer term, i.e. up to 2050, many of the recommended measures to improve health systems and public health, such as investing in the health workforce, service delivery, and governance, and lifting people out of poverty, will help to mitigate the effects of heat.13 However, the broader impacts of climate change—including morbidity and mortality over the medium to long term—remain deeply uncertain, reflecting complex relationships between highly interdependent biophysical and social systems.9 It is therefore of value to continue research to characterise and identify ways of avoiding the remaining health risks, including the impacts of flooding, droughts, migration, and uncompensable heat stress ("the set of environmental conditions under which a healthy human being can no longer maintain a stable core temperature without the assistance of external cooling."14,15 It will also be valuable to identify rapid pathways to net zero emissions globally that impose no unfair burdens on lower-income and recently industrialized countries.

REFERENCES:

1. WHO. Health for All – transforming economies to deliver what matters: final report of the WHO Council on the Economics of Health for All [Internet]. Geneva: World Health Organization; 2023. Available from: https://www.ucl.ac.uk/bartlett/public-purpose/sites/bartlett_public_purpose/files/health_for_all_who_council_final_report.pdf
2. Scovronick N, Vasquez VN, Errickson F. Human Health and the Social Cost of Carbon: A Primer and Call to Action. Epidemiology. 2019;30:642–7.
3. I.P.C.C. Climate Change 2022: Impacts, Adaptation and Vulnerability. Contribution of Working Group II to the Sixth Assessment Report of the Intergovernmental Panel on Climate Change [Pörtner H-O. Roberts DC, Tignor M, editors. Cambridge, UK and New York, NY, USA: Cambridge University Press. Cambridge University Press; 2022.
4. IPCC. Special Report. Global Warming of 1.5 0C. Impacts of1.5 0C global warming on natural and human systems. Chapter [Internet]. 2018;3. Available from: https://www.ipcc.ch/sr15/chapter/chapter-3/
5. Bressler RD, Moore FC, Rennert K, Anthoff D. Estimates of country level temperature-related mortality damage functions. Sci Rep. 2021 Oct 13;11(1):20282.
6. Carleton T, Jina A, Delgado M. Valuing the Global Mortality Consequences of Climate Change Accounting for Adaptation Costs and Benefits. Q J Econ. 2022;137:2037–105.
7. Rennert K, Errickson F, Prest BC. Comprehensive evidence implies a higher social cost of CO2. Nature. 2022;610:687–92.
8. Nordhaus WD. Revisiting the social cost of carbon. Proc Natl Acad Sci U S A. 2017;114:1518–23.
9. Rising J, Tedesco M, Piontek F, Stainforth DA. The missing risks of climate change. Nature. 2022;
10. Gasparrini A, Guo Y, Sera F. Projections of temperature-related excess mortality under climate change scenarios. Lancet Planet Health. 2017;
11. Intergovernmental Panel on Climate Change (IPCC). Climate Change 2013 – The Physical Science Basis: Working Group I Contribution to the Fifth Assessment Report of the Intergovernmental Panel on Climate Change [Internet]. Cambridge: Cambridge University Press; 2014. Available from: https://www.cambridge.org/core/books/climate-change-2013-the-physical-science-basis/BE9453E500DEF3640B383BADDC332C3E (accessed July 8, 2024)
12. Stern N, Stiglitz Charlotte Taylor J, Taylor C. The economics of immense risk, urgent action and radical change: towards new approaches to the economics of climate change. J Econ Methodol. 2022;29:181–216.
13. Papworth A, Maslin M, Randalls S. Is climate change the greatest threat to global health? Geogr J. 2015;181:413–22.
14. Powis CM, Byrne D, Gassert KN. Observational and model evidence together support wide-spread exposure to noncompensable heat under continued global warming. Sci Adv. 2023;9:eadg9297.
15. Vecellio DJ, Kong Q, Kenney WL, Huber M. Greatly enhanced risk to humans as a consequence of empirically determined lower moist heat stress tolerance. Proc Natl Acad Sci. 2023;120:e2305427120.

PANEL A.2

The importance of the "next 7,000 days"

We have demonstrated that the 15 priority conditions account for most of the mortality differentials across regions. These 15 conditions are the most immediate causes of death but have many causal components and associated risk factors: Most premature deaths—from infections as well as NCDs—are determined by exposures occurring repeatedly or over an extended period involving many interlinked factors starting as early as the fetal stage.[1] Therefore, a life course perspective is needed to fully understand and prevent risk factors leading to premature death. The overwhelming focus of the life-course literature has been the "first 1,000 days" from conception; linking maternal, neonatal, and infant health and nutrition to mortality, infections, and chronic diseases at subsequent life stages, including adulthood and old age.[2,3] Therefore, preventing adverse exposures in early life—particularly diarrhea, respiratory infections, and neonatal conditions—will have compounding effects on premature mortality throughout the life course.

However, little attention is paid to human development between the first 1,000 days and adulthood— "the next 7,000 days." Using the number of global publications to measure research interest, 70% to 90% of research attention on the first 20 years of life has been expended before the subjects reach 5 years of age.[4,5] Yet the "next 7,000 days," from about 5 to 19 years, is increasingly recognized as a crucial period for human development. It is a period of dramatic growth and change, including the physical and emotional changes associated with puberty, the adolescent growth spurt, and the rewiring of the adolescent brain that establishes lifelong mental health and behavioural trajectories.6 For example, adolescence is when most smoking initiation occurs.[7]

Education and health later in life are heavily impacted by events and decisions during adolescence,[8] which in turn affects future health and mortality. Estimates suggest that completing 12 years of schooling reduces the risk of mortality in adulthood by 25%, or almost two percent for each additional year of education.[9] Suggested pathways are varied, including higher income,access to quality care, and improved health-related knowledge and healthy behaviours.[10]

A still weak, but rapidly improving evidence base points to enormous inequalities across countries in key aspects of development during adolescence.

Here we use two special data sets to explore two key aspects of this relationship. For physical growth, we use data compiled and harmonized by the Biomarkers Reflecting Inflammation and Nutritional Determinants of Anemia (BRINDA) Project to form one of the most comprehensive datasets of school-age child and adolescent nutrition biomarkers.

Young M, Luo H, Wang Y, Werner ER, Antoninis M, Gouëdard P, Schultz LS, Chang AY, Jamison DT and Bundy DAP. The importance of the "next 7,000 days." Panel in appendix to Global health 2050: the path to halving premature death by mid-century, Lancet 2024; Jamison DT, Summers LH, et al. published online Oct 14, 2024, https://doi.org/10.1016/

This multi-country data set shows that the prevalence of stunting, which correlates with educational underachievement, is in the range of 10% to 30% in children in low- and middle-income countries (figure A for females; figure B for males). Stunting persists not only during the first 1,000 days, but also throughout the age range 5-19 years when we invest most in children's education. The results suggest that in some countries nearly a third of 17-year-old girls remain at the height of a normally growing 12-year-old girl, while the survey results from high-income countries show that stunting can be largely avoidable at any age. Further, evidence suggests that although early stunting leads to a low growth trajectory if the social determinants remain unchanged,[12] early stunting is largely reversible through appropriate interventions.[13]

Panel A.2 Figure A:

Prevalence of stunting among females in selected countries

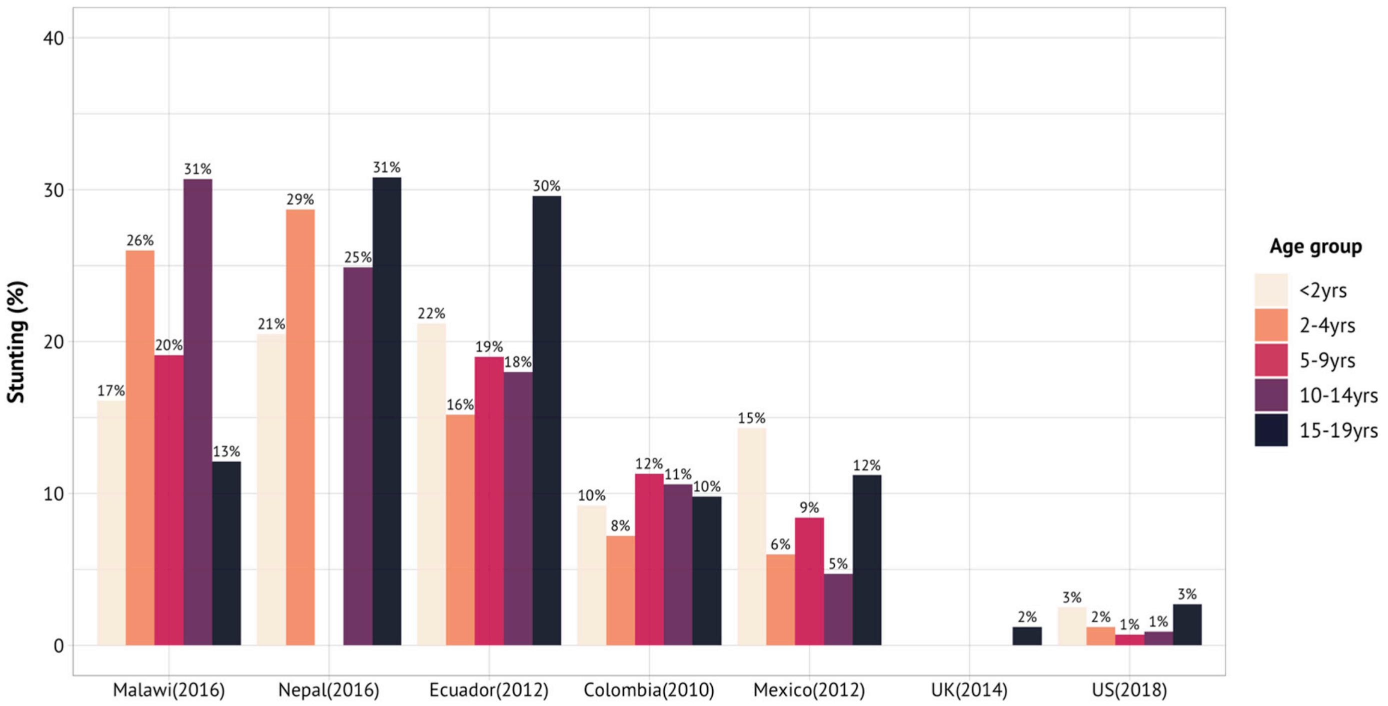

SOURCE:

Biomarkers Reflecting Inflammation and Nutritional Determinants of Anemia (BRINDA) Project

Panel A.2 Figure B:

Prevalence of stunting among males in selected countries

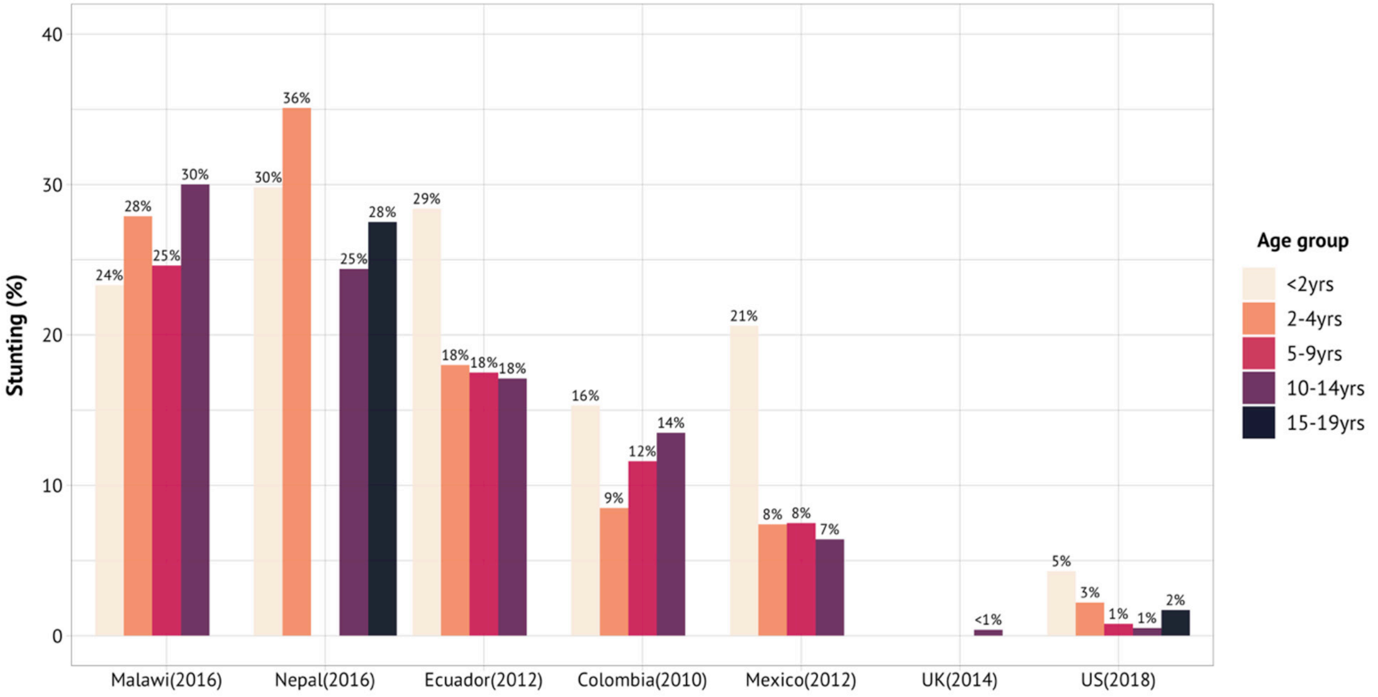

SOURCE:

Biomarkers Reflecting Inflammation and Nutritional Determinants of Anemia (BRINDA) Project

For cognitive development, we used data from the standardized mathematics test conducted by the OECD as part of their Programme for International Student Assessment (PISA).14 Comparing the 2022 test results with the achievable results in a "frontier" country, in this case Singapore, figure C shows huge cross-country gaps in math test performance, with very substantial and significant under-performance even in middle- and high-income countries. The level of achievement in low-income countries would be expected to be even further away from what is possible, but low-income countries are not currently included in any global comparative assessment of education achievement. The huge scale of this difference, comparable in scale to that seen with physical growth, argues for a link between under-achievement in physical growth and cognitive development.

Panel A.2 Figure C

Mathematics tests scores relative to Singapore, PISA 2022, selected countries

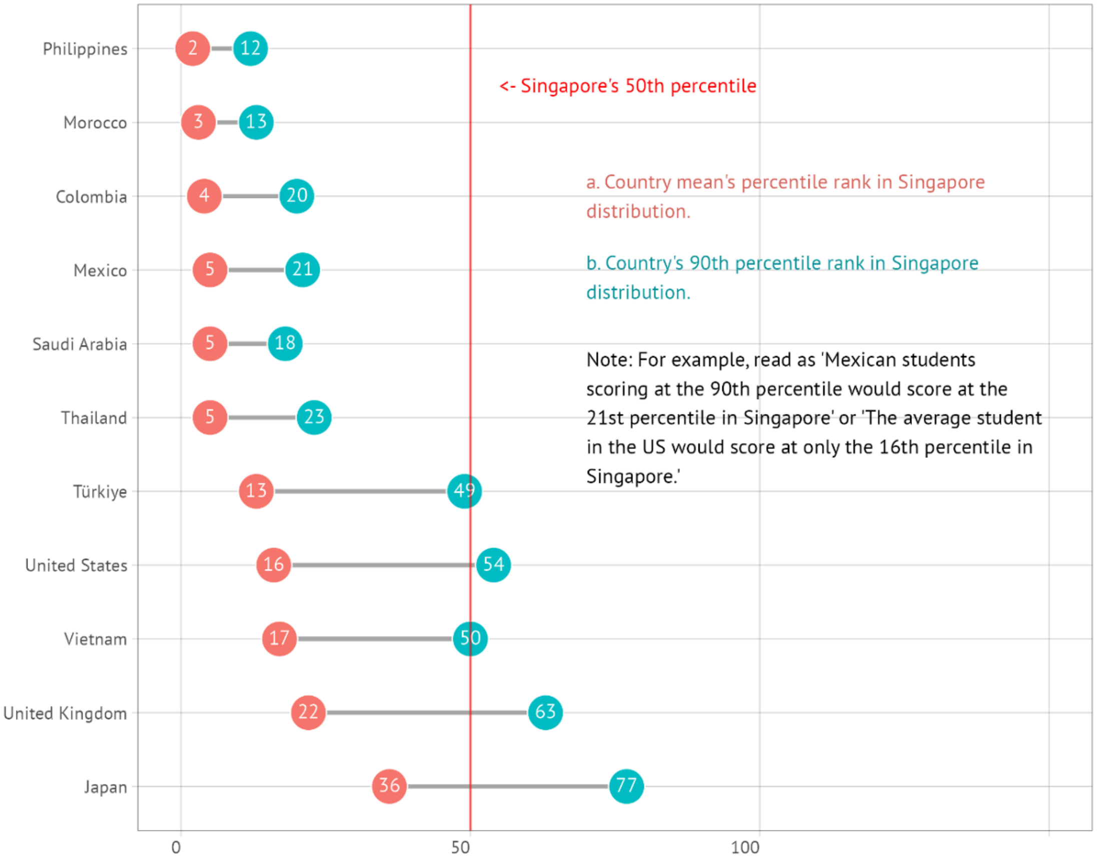

SOURCE: From Young M, Antoninis M, Addo Y, Jamison DT, Bundy DAP, BRINDA working group. Are global disparities in nutrition associated with scholastic performance of school-age children and adolescents? A Working Paper of the Research Consortium for School Health and Nutrition, an initiative of the School Meals Coalition. 2024 15

The world invests some USD 2.8 trillion globally (1 trillion in low- and middle-income countries) in the education of young people 5-19 years.16 The Disease Control Priorities, Third Edition (DCP3) estimates that in in low- and middle-income countries the investment in the health and wellbeing of this age group is just 2% of the education investment, and only 13% of the health investment in children under 5 years.[6]

Key interventions in the "next 7,000" days

There is an established literature on school health and school meals, which has used analysis of cost-effectiveness to identify a school health and nutrition package.13,17,18 The essential package for school-age children in DCP3 includes interventions that can be delivered in a school setting to improve cognition and learning, including: the provision of deworming tablets in endemic settings; vision screening and provision of spectacles;

promotion of oral health and insecticide-treated bed nets in malaria-endemic areas; and the delivery of dietary education and healthy school meals. In addition, the package protects against vaccine-preventable conditions in adulthood and among future offspring by including tetanus toxoid and HPV vaccinations.[18] Looking forward, there is growing recognition of the long-term value of early surgical intervention during school age and adolescence[19] and of the potential damage to mental health of inappropriate social media. The interventions and their benefits are summarized in panel A.2 table A.

There is growing global support for school-based interventions, in particular health services and school meals. School-based health services, such as deworming and vision screening, can improve school children’s health and well-being, supporting them to lead a well-educated and healthy life. Increasing coverage of school meals and school health services is now a global movement supported by the School Meals Coalition, with an emphasis on increasing coverage where it is needed the most. In 2022, estimates indicate that 420 million children were reached by school health and nutrition programmes, and that between 80 and 140 million of the most vulnerable children in low- and middle-income countries remained unreached. Expansion of national school health and nutrition programmes to reach those most in need should be a global goal.[20]

Panel A.2 Table A

Essential health services appropriate for children and adolescents as proposed by DCP3

	Primary health center	School	Benefit of intervention delivery in schools
**PHYSICAL HEALTH**			
Deworming	Deworming	Deworming	In endemic areas, regular deworming (following WHO guidelines) can be done inexpensively in schools since most deworming drugs are donated; benefits in school attendance has been reported as a result.
Insecticide-treated net promotion	Insecticide-treated net promotion	Insecticide-treated net promotion	Education about the use of insecticide treated nets in endemic areas is important because schoolchildren tend to use nets less often than mothers and small children.
Tetanus toxoid and HPV vaccination	Tetanus toxoid and HPV vaccination	Tetanus toxoid and HPV vaccination	Schools can be a good venue for administration of tetanus boosters, which benefit young people and babies born to those young women.
Oral health promotion	Oral health promotion and treatment	Oral health promotion	Education on oral health is important; poor households generally cannot afford dental treatment.
Correcting refractive error	Vision screening and provision of glasses	Vision screening and provision of glasses	Vision screening and provision of inexpensive ready-made glasses boost school performance.
**NUTRITION**			
Micronutrient supplementation	..	Micronutrient supplementation	Supports learning and the school is a good venue for administering micronutrient supplements which benefit young people and babies born to those young women.
Multifortified foods	..	Multifortified foods	Supports learning
Food provision	..	School feeding	School meals promote attendance and education outcomes

HPV=human papillomavirus. School-age children do not regularly contact the health system unless they seek treatment. With the remarkable success of the Millennium Development Goals in increasing enrolment and participation and the continuing focus on universal education with the Sustainable Development Goals, it makes sense to use schools to promote health in this age group and to deliver preventive and curative health interventions. These interventions are affordable and the highest priority because of their health and educational benefits. Table 5 presents the cost of components of the essential package of investments for school-age children. Data are from Fernandes and Aurino 18.

REFERENCES:

1. Gillman Matthew W. Developmental Origins of Health and Disease. New England Journal of Medicine. 2005;353(17):1848–50.
2. Almond D. Is the 1918 Influenza Pandemic Over? Long- Term Effects of In Utero Influenza Exposure in the Post- 1940 U.S. Population. Journal of Political Economy. 2006;114(4):672–712.
3. Victora CG, Adair L, Fall C, Hallal PC, Martorell R, Richter L, et al. Maternal and child undernutrition: consequences for adult health and human capital. The Lancet. 2008 Jan 26;371(9609):340–57.
4. Bundy DAP, Silva N, Horton S, Patton G, Schultz L, Jamison DT. Investment in child and adolescent health and development: key messages from Disease Control Priorities. Lancet. 3rd ed. 2018;391:687–99.
5. Schultz L, Bundy DAP. The lost children: what an analysis of publication effort tells us about the extraordinary age bias in health research on young people. Forthcoming; 2024.
6. Bundy DAP, Silva N, Horton S, Patton GC, Schultz L, Jamison DT. Child and Adolescent Health and Development: Realizing Neglected Potential. In: Bundy DAP, Silva N, Horton S, Jamison DT, Patton GC, editors. Disease Control Priorities, Third Edition (Volume 8): Child and Adolescent Health and Development. 3rd ed. Washington, DC: World Bank Group; 2017N. p. 1–24.
7. Reitsma MB, Flor LS, Mullany EC, Gupta V, Hay SI, Gakidou E. Spatial, temporal, and demographic patterns in prevalence of smoking tobacco use and initiation among young people in 204 countries and territories, 1990–2019. The Lancet Public Health. 2021 Jul 1;6(7):e472–81.
8. Cunha F, Heckman J. Human Capital Formation in Childhood and Adolescence. 2009; Available from: https://www.ifo.de/DocDL/dicereport409-forum5.pdf (accessed August 19, 2024)
9. Balaj M, Henson CA, Aronsson A, Aravkin A, Beck K, Degail C, et al. Effects of education on adult mortality: a global systematic review and meta-analysis. The Lancet Public Health. 2024 Mar 1;9(3):e155–65.
10. Baker DP, Smith WC, Muñoz IG, Jeon H, Fu T, Leon J, et al. The Population Education Transition Curve: Education Gradients Across Population Exposure to New Health Risks. Demography. 2017 Oct;54(5):1873–95.
11. UNESCO Institute of Statistics. UNESCO Institute of Statistics. Visualizing Indicators of Education for the World (VIEW [Internet]. Paris; 2023. Available from: https://education-estimates.org/out-of-school/data/ (accessed August 19, 2024)
12. Victora CG, Hartwig FP, Vidaletti LP, Martorell R, Osmond C, Richter LM, et al. Effects of early-life poverty on health and human capital in children and adolescents: analyses of national surveys and birth cohort studies in LMICs. The Lancet. 2022 Apr 30;399(10336):1741–52.
13. Watkins, Bundy DAP, Jamison DT, Fink G, Georgiadis A. Evidence of Impact of Interventions on Health and Development during Middle Childhood and School Age. In: Bundy DAP, Silva N, Horton S, Jamison DT, Patton GC, editors. Disease Control Priorities, Third Edition (Volume 8): Child and Adolescent Health and Development. 8th ed. Washington, DC: World Bank Group; 2017. p. 99–106.
14. Organisation for Economic Co-operation and Development. Programme for International Student Assessment Database [Internet]. Paris; 2023. Available from: https://www.oecd.org/pisa/data/ (accessed August 19, 2024)
15. Young M, Antoninis M, Addo Y, Jamison DT, Bundy DAP, BRINDA working group. Are global disparities in nutrition associated with scholastic performance of school-age children and adolescents? A Working Paper of the Research Consortium for School Health and Nutrition, an initiative of the School Meals Coalition. 2024;
16. UNESCO, World Bank. Education Finance Watch 2023 [Internet]. Washington, DC; Paris; 2023. Available from: https://www.unesco.org/gem-report/en/2023efw (accessed August 19, 2024)
17. Bundy DAP, Silva N, Horton S, Patton GC, Schultz L, Jamison DT. Child and adolescent health and development: realizing neglected potential. In: Bundy DAP, Silva N, Horton S, Jamison DT, Patton GC, eds. Disease control priorities (volume 8): child and adolescent health and development, 3rd edn. Washington, DC: World Bank Group, 2017: 1–24.
18. Fernandes M, Aurino E. Identifying an Essential Package for School-Age Child Health: Economic Analysis. In: Bundy DAP, Silva N, Horton S, Jamison DT, Patton GC, editors. Disease Control Priorities, Third Edition (Volume 8): Child and Adolescent Health and Development. 3rd ed. Washington, DC: World Bank; 2017. p. 355–68.
19. Seyi-Olajide J, Ali A, Francis William PJr, Samad L, Banu T, Abdelhafeez H, et al. Surgery and the First 8000 Days of Life [Internet]. Bergen; 2023. Available from: https://www.uib.no/sites/w3.uib.no/files/attachments/8000_days.pdf
20. Lu X, Bundy DAP, Burbano C, Antoninis M, Verguet S. Re-Estimating global school feeding program targets: how has the size of the global population of vulnerable school-age children changed post COVID Pandemic. A Working Paper of the Research Consortium for School Health and Nutrition of the School Meals Coalition. 2024.

PANEL A.3

New tools for prevention and treatment of tuberculosis

Globally, tuberculosis is now the number one infectious disease killer, causing about 1.3 million deaths in 2022;1 it is a major cause of premature mortality in high-burden nations. Last year, the Lancet Commission on Tuberculosis published its report "Scientific advances and the end of tuberculosis," which argued that a new set of tuberculosis control technologies provided grounds for optimism in reaching a tuberculosis-free world within a generation.2 Such tools, it argued, would need to be adopted wholesale, implemented at scale, and accompanied by sustained investment in development of new tools and in tuberculosis programmes for this vision to be achieved. These tools fall into three categories: diagnostics, therapeutics, and preventive interventions.

DIAGNOSTICS Diagnosis, say Pai and colleagues, is "the weakest aspect of TB care and control"—without diagnosis, the disease cannot be treated.3 Prior to the COVID-19 pandemic, around 2.9 million people annually had tuberculosis without being diagnosed, and the annual diagnostic gap grew to 4.2 million during the pandemic. Using new diagnostics along the case finding "cascade"—for those with subclinical tuberculosis, those with symptoms who have not sought care, and those who have sought care but are undiagnosed—could reap large benefits.2 New molecular diagnostics are now available that can be used at the point of care (POC) in decentralized settings and the WHO recommends molecular diagnosis over sputum smear microscopy as the preferred frontline testing option. Given the limitations of using sputum, especially in children and people with HIV, efforts are now underway to develop molecular diagnostics based on tongue swabs, urine, blood, and stool, and to develop multi-disease tests. "A simple, non-sputum sample," say Pai et al,3 "combined with an affordable, multi-disease POC molecular technology, deployed in decentralized settings would reach a much larger population, close the case detection gap, and curb TB transmission at the population level." Screening for tuberculosis and latent tuberculosis have been made more feasible with the advent of interferon-gamma release assays (IGRAs), which have many advantages over traditional tuberculin skin tests—for example, they are unaffected by prior BCG vaccination and have a higher specificity. However, IGRAs are labor intensive to use and are costly. The Serum Institute of India mass produces a newer skin test, C-tb, that has a similar sensitivity and specificity to IGRAs (the specificity is unaffected by prior BCG vaccination), while retaining the operational advantages of a skin test; C-tb is one tenth of the cost of IGRAs.4

THERAPEUTICS There have been striking advances over the last 5 years in the treatment of tuberculosis, including one-month regimens for tuberculosis prevention, a reduction in the duration of treatment of drug-susceptible tuberculosis down to 4 months, and the approval of 6-month treatment regimens for drug-resistant tuberculosis. These advances are now promoted by the 1/4/6x24 (one, four, six by 2024) campaign, launched at the AIDS 2022 conference in Montreal (one month regimen for prevention; four month regimen for sensitive disease; six month regimen for resistant disease). The WHO and the US Centers for Disease Control and Prevention recently approved the 4-month isoniazid–rifapentine–moxifloxacin–pyrazinamide (4HPMZ) regimen for eligible people with drug-susceptible tuberculosis, which "speaks to the momentum of the development of new treatments for drug-susceptible

tuberculosis."2 The 2023 TRUNCATE-TB trial suggests that even a two-month regimen for drug-susceptible tuberculosis may be feasible.5 For multidrug-resistant and rifampicin-resistant tuberculosis, we are in a "golden age of innovation."2 In particular, in 2022, the WHO codified a six month regimen of bedaquiline–pretomanid–linezolid augmented with moxifloxacin (6BPaLM) in its guidelines as the preferred regimen for adults and adolescents aged 14 years and older. Long-acting injectable drugs ("depot formulations") are currently being studied for both preventive therapy and treatment of active disease. Such formulations could improve adherence and significantly ease the burdens associated with long-term daily pill intake, both for individual patients and for public health systems.

PREVENTIVE INTERVENTIONS Reducing tuberculosis incidence will require aggressive scale-up of tuberculosis preventive treatment (TPT) to those at highest risk of latent tuberculosis infection, such as people living with HIV and household contacts younger than 5 years. An estimated 1.7 billion have tuberculosis infections that are "latent."6 While latent tuberculosis infection causes no actual harm and cannot be spread, there is a substantial risk that such latent infections can become active. TPT addresses this population. The advent of short-course TPT (e.g., 12 weeks of once-weekly isoniazid and rifapentine [3HP] or 1 month of daily isoniazid and rifapentine [1HP]) makes scale-up more feasible. Last year, India committed to expanding 3HP nationwide; for other countries to follow suit, licensing and cost barriers will need to be addressed. On the preventive vaccine front, progress has been greatly hindered by lack of funding. This lack is a key reason why it took over 19 years for one promising candidate, M72, a fusion protein of two M tuberculosis antigens administered with a potent adjuvant, to move from early clinical development studies to a phase 3 trial. Two other vaccine candidates are also now in late stage trials: (i) VPM 1002, a next-generation, genetically modified BCG vaccine, and (ii) MTBVAC, an M tuberculosis strain attenuated via two genetic mutations.

REFERENCES:

1. WHO. Health for All – transforming economies to deliver what matters: final report of the WHO Council on the Economics of Health for All [Internet]. Geneva: World Health Organization; 2023. Available from: https://www.ucl.ac.uk/bartlett/public-purpose/sites/bartlett_public_purpose/files/health_for_all_who_council_final_report.pdf
2. Reid M, Agbassi YJP, Arinaminpathy. Scientific advances and the end of tuberculosis: a report from the Lancet Commission on Tuberculosis. Lancet. 2023;402:1473–98.
3. Pai M, Dewan PK, Swaminathan S. Transforming tuberculosis diagnosis. Nature Microbiology. 2023;8:765–759.
4. Aggerbeck H, Giemza R, Joshi P, Tingskov PN, Hoff ST, Boyle J, et al. Randomised Clinical Trial Investigating the Specificity of a Novel Skin Test (C-Tb) for Diagnosis of M. tuberculosis Infection. PLOS ONE. 2013 May 14;8(5):e64215.
5. Paton NI, Cousins C, Suresh C. Treatment strategy for rifampin-susceptible tuberculosis. N Engl J Med. 2023;388:873–87.
6. Houben RMGJ, Dodd PJ. The Global Burden of Latent Tuberculosis Infection: A Re-estimation Using Mathematical Modelling. PLoS Med. 2016;13:e1002152.

PANEL A.4

Developing antibiotics in the age of AI

Antimicrobial resistance (AMR) continues to be a major threat. In 2019, nearly 1.3 million people died from resistant infections.1 By 2050, the death toll related to AMR could reach 10 million annually.2 For most of the 20th century, as resistance to specific antibiotics grew, physicians could rely on the discovery and development of new antibiotics to replace them. Now, however, weak incentives provided by the patent and limited investment in innovation have led to a decades-long discovery drought and a dwindling antibiotics pipeline.

Recent antibiotic development has focused on making small modifications to existing classes. Though these may create marginal improvements such as increased efficacy or decreased toxicity, in the short term, due to the structural similarities, these antibiotics are ultimately just as susceptible to resistance as the original. Today, some form of resistance has developed against every major class of antibiotics. The AMR crisis will not be solved unless we can quickly develop novel treatments against superbugs and ensure appropriate use. Here, the right use of AI could prove transformational.

AI is uniquely useful for antibiotic discovery for three reasons:

1. it offers unprecedented opportunities to search across vast and unknown chemical spaces for truly novel compounds;
2. the efficacy of AI-generated compounds can be quickly tested in a petri dish, creating a rapid and efficient feedback loop that enables ever-more targeted drugs to be developed; and
3. the speed and potential cost efficiencies of AI-based discovery can help overcome some of the funding challenges now stifling antibiotic development.

For example, Phare Bio is a social venture harnessing the power of AI to tackle the antibiotic pipeline crisis. In collaboration with Professor Jim Collins' lab at Massachusetts Institute of Technology, Phare Bio's mission is to use AI and a translational feedback loop to rapidly discover, design, and develop new antibiotic classes against the world's most urgent bacterial threats, as designated by the WHO and CDC. The Collins lab's first breakthrough discovery was published in Cell in 2020, detailing how the team used machine learning to identify a broad-spectrum antibiotic with potent activity across Gram-negative bacterial pathogens.3 Since then, the team has made significant enhancements to the AI platform, including the incorporation of generative AI that is moving beyond identifying antibiotic hits from existing compound libraries to de novo design of entirely new compounds, and "explainable" AI that elucidates how the model is learning and making its predictions.4 The use of these methods has produced new and promising candidates, including the discovery of a new structural class of compounds active against Gram-positive bacteria such as MRSA.5

Recognizing a need to move these discoveries out of academia and through the "valley of death" (the phases of development characterized by high failure rates and lack of financial investment), Phare Bio uses philanthropic and grant funding to build its pipeline of novel, de-risked preclinical candidates, and leverages commercial partnerships for more costly clinical development. With this approach, Phare Bio can file intellectual property for their novel candidates and leverage it for out-licensing agreements with

subsequent development partners – further bolstering the financial sustainability of the organization. This blended-finance model addresses the trade-off between profits for privately held intellectual property in these compounds, having prices that can ultimately reach the patients in greatest need, and the philanthropic finance required. The challenge will be for the philanthropists to ensure that their investments do, indeed, result in low prices and widely accessible products rather than high profits.

REFERENCES:

1. Antimicrobial Resistance Collaborators. Global burden of bacterial antimicrobial resistance in 2019: a systematic analysis. The Lancet. 2022;399(10325):629–55.
2. O'Neill J. Review on Antimicrobial Resistance. Tackling Drug-Resistant Infections Globally [Internet]. 2016. Available from: https://amr-review.org/sites/default/files/160525_Final%20paper_with%20cover.pdf (accessed August 19, 2024)
3. Stokes JM, Yang K, Swanson K. A deep learning approach to antibiotic discovery. Cell. 2020;180(4):688–702.
4. Wong F, Zheng EJ, Valeri JA. Discovery of a structural class of antibiotics with explainable deep learning. Nature. 2024;626:177–85.
5. Anderson M, Panteli D, Kessel R van, Ljungqvist G, Colombo F, Mossialos E. Challenges and opportunities for incentivising antibiotic research and development in Europe. The Lancet Regional Health – Europe [Internet]. 2023 Oct 1 [cited 2024 Jun ;33. Available from: https://www.thelancet.com/journals/lanepe/article/PIIS2666-7762(23)00124-2/fulltext
